# Systematics of the parasitic wasp genus ***Oxyscelio*** Kieffer (Hymenoptera, Platygastridae s.l.), Part I: Indo-Malayan and Palearctic fauna

**DOI:** 10.3897/zookeys.292.3867

**Published:** 2013-04-18

**Authors:** Roger A. Burks, Lubomír Masner, Norman F. Johnson, Andrew D. Austin

**Affiliations:** 1 Department of Evolution, Ecology, and Organismal Biology, The Ohio State University, 1315 Kinnear Road, Columbus, OH 43212, U.S.A.; 2 Agriculture and Agri-Food Canada, K.W. Neatby Building, Ottawa, ON K1A 0C6, Canada; 3 Australian Centre for Evolutionary Biology and Biodiversity, School of Earth and Environmental Sciences, The University of Adelaide, SA 5005, Australia

**Keywords:** Platygastroidea, Scelionidae, *Oxyscelio*, Scelioninae, key, revision, database, parasitoid

## Abstract

The Indo-Malayan and Palearctic species of *Oxyscelio* (Hymenoptera: Platygastridae s.l.) are revised. A total of 90 species are recognized as valid, 19 of which are redescribed - *Oxyscelio acutiventris* (Kieffer), *Oxyscelio brevinervis* (Kieffer), *Oxyscelio carinatus* (Kieffer), *Oxyscelio ceylonensis* (Dodd), *Oxyscelio consobrinus* (Kieffer), *Oxyscelio crassicornis* (Kieffer), *Oxyscelio cupularis* (Kieffer), *Oxyscelio dorsalis* (Kieffer), *Oxyscelio excavatus* (Kieffer), *Oxyscelio flavipennis* (Kieffer), *Oxyscelio florus* Kononova, *Oxyscelio foveatus* Kieffer, *Oxyscelio kiefferi* Dodd, *Oxyscelio magnus* (Kieffer), *Oxyscelio marginalis* (Kieffer), *Oxyscelio naraws* Kozlov & Lê, *Oxyscelio perpensus* Kononova, *Oxyscelio rugosus* (Kieffer) and *Oxyscelio spinosiceps* (Kieffer), and 71 which are described as new - *Oxyscelio aclavae* Burks, **sp. n.**, *Oxyscelio amrichae* Burks, **sp. n.**, *Oxyscelio anguli* Burks, **sp. n.**, *Oxyscelio angustifrons* Burks, **sp. n.**, *Oxyscelio angustinubbin* Burks, **sp. n.**, *Oxyscelio arcus* Burks, **sp. n.**, *Oxyscelio arvi* Burks, **sp. n.**, *Oxyscelio asperi* Burks, **sp. n.**, *Oxyscelio aureamediocritas* Burks, **sp. n.**, *Oxyscelio bipunctuum* Burks, **sp. n.**, *Oxyscelio brevidentis* Burks, **sp. n.**, *Oxyscelio caesitas* Burks, **sp. n.**, *Oxyscelio capilli* Burks, **sp. n.**, *Oxyscelio capitis* Burks, **sp. n.**, *Oxyscelio cavinetrion* Burks, **sp. n.**, *Oxyscelio chimaerae* Burks, **sp. n.**, *Oxyscelio codae* Burks, **sp. n.**, *Oxyscelio convergens* Burks, **sp. n.**, *Oxyscelio cordis* Burks, **sp. n.**, *Oxyscelio crateris* Burks, **sp. n.**, *Oxyscelio crebritas* Burks, **sp. n.**, *Oxyscelio crustum* Burks, **sp. n.**, *Oxyscelio cuculli* Burks, **sp. n.**, *Oxyscelio cyrtomesos* Burks, **sp. n.**, *Oxyscelio dasymesos* Burks, **sp. n.**, *Oxyscelio dasynoton* Burks, **sp. n.**, *Oxyscelio dermatoglyphes* Burks, **sp. n.**, *Oxyscelio doumao* Burks, **sp. n.**, *Oxyscelio fistulae* Burks, **sp. n.**, *Oxyscelio flabellae* Burks, **sp. n.**, *Oxyscelio flaviventris* Burks, **sp. n.**, *Oxyscelio fodiens* Burks, **sp. n.**, *Oxyscelio fossarum* Burks, **sp. n.**, *Oxyscelio fossularum* Burks, **sp. n.**, *Oxyscelio genae* Burks, **sp. n.**, *Oxyscelio granorum* Burks, **sp. n.**, *Oxyscelio granuli* Burks, **sp. n.**, *Oxyscelio greenacus* Burks, **sp. n.**, *Oxyscelio halmaherae* Burks, **sp. n.**, *Oxyscelio intermedietas* Burks, **sp. n.**, *Oxyscelio jaune* Burks, **sp. n.**, *Oxyscelio jugi* Burks, **sp. n.**, *Oxyscelio kramatos* Burks, **sp. n.**, *Oxyscelio labis* Burks, **sp. n.**, *Oxyscelio lacunae* Burks, **sp. n.**, *Oxyscelio latinubbin* Burks, **sp. n.**, *Oxyscelio latitudinis* Burks, **sp. n.**, *Oxyscelio limae* Burks, **sp. n.**, *Oxyscelio longiventris* Burks, **sp. n.**, *Oxyscelio mesiodentis* Burks, **sp. n.**, *Oxyscelio mollitia* Burks, **sp. n.**, *Oxyscelio nasolabii* Burks, **sp. n.**, *Oxyscelio nodorum* Burks, **sp. n.**, *Oxyscelio noduli* Burks, **sp. n.**, *Oxyscelio nubbin* Burks, **sp. n.**, *Oxyscelio obsidiani* Burks, **sp. n.**, *Oxyscelio ogive* Burks, **sp. n.**, *Oxyscelio operimenti* Burks, **sp. n.**, *Oxyscelio peludo* Burks, **sp. n.**, *Oxyscelio planocarinae* Burks, **sp. n.**, *Oxyscelio praecipitis* Burks, **sp. n.**, *Oxyscelio reflectens* Burks, **sp. n.**, *Oxyscelio regionis* Burks, **sp. n.**, *Oxyscelio sinuum* Burks, **sp. n.**, *Oxyscelio spinae* Burks, **sp. n.**, *Oxyscelio striarum* Burks, **sp. n.**, *Oxyscelio tecti* Burks, **sp. n.**, *Oxyscelio unguis* Burks, **sp. n.**, *Oxyscelio vadorum* Burks, **sp. n.**, *Oxyscelio vittae* Burks, **sp. n.** and *Oxyscelio zeuctomesos*. Neotypes are designated for nine species, including the type species *O*.* foveatus* Kieffer, *Oxyscelio brevinervis* (Kieffer), *Oxyscelio bifurcatus* (Kieffer), *Oxyscelio frontalis* (Kieffer), *Oxyscelio crassicornis* (Kieffer), *Oxyscelio cupularis* (Kieffer), *Oxyscelio foveatus* Kieffer, *Oxyscelio kiefferi* Dodd, *Oxyscelio magnus* (Kieffer) and *Oxyscelio marginalis* (Kieffer). *Oxyscelio bifurcatus* (Kieffer) **syn. n. **and *Oxyscelio frontalis* (Kieffer) **syn. n.** are synonymized under *Oxyscelio consobrinus* (Kieffer). The fauna is divided into 13 species groups, with six species unplaced to a group. A phylogenetic analysis employing 73 morphological characters did not find most of these groups to be monophyletic, but they are retained to aid in specimen identification. Potential biogeographical patterns are discussed, including regional variation in surface sculpture and a morphological link between Sri Lankan and Australian species.

## Introduction

The genus *Oxyscelio* Kiefer comprises relatively robust platygastoid wasps that occur across equatorial and east Africa, the south-eastern part of the Palearctic, the Indo-Malayian and Australasian regions. They are relatively easily identified by the fore wing submarginal vein being distant from wing margin, very short marginal vein, virtually absent postmarginal vein, posteriorly rounded vertex, and distinct metascutellum. In addition, many species can be recognised by the pronounced frontal depression on the head which is often rimmed by a carina.

The genus was first erected for a single species from Indonesia,* Oxyscelio foveatus*, by Kieffer (1907), which he later treated as a subgenus of *Chromoteleia* Ashmead (Kieffer 1910a) but later raised again to generic rank (Kieffer 1926). Following its original description the status of *Oxyscelio* and the genera that surround it was particularly confusing. This is clearly evident in the fact that, other than the type species, all taxa described prior to 1930 that are currently accommodated in *Oxyscelio* were described under other generic names. This situation was resolved by Dodd (1931) who recognized a range of species from the Oriental and Australian regions as being congeneric based on several key characters, the form of the fore wing venation (outlined above) and the structure of the metanotal plate. In so doing he treated *Dicroteleia* Kieffer, *Camptoteleia* Kieffer and *Xenoteleia* Kieffer as junior synomyms of *Oxyscelio*, and transferred 32 species to that genus including all Australian taxa that he had preciously described under *Sceliomorpha* Ashmead (*sensu* Kieffer 1926). This work by Dodd (1931) and his other studies on various scelionid genera around that time provided significant taxonomic stability and are testament to his thoughtful and perceptive approach to discriminating genera and species.

Since the descriptive work of Kieffer and Dodd prior to1920, only three additional species of *Oxyscelio* have been added to the world fauna (Kozlov and Lê 2000; Kononova 2007). Masner (1976) provided a diagnosis of the genus and key to separate putative related genera, and Johnson (1992) catalogued the world’s species.

The current study is the first of three papers that aim to fully revise the world species of *Oyscelio*, focusing on describing the large number of new taxa. This first paper deals with the Indo-Malayan and Palearctic species; the second one will treat the Australasian taxa, and the third one the African species. This work has arisen from our Platygastroidea Planetary Biodiversity Inventory (see below) which aims to revise all species on a worldwide basis for a number of important platygastroid genera.

The contributions of the individual authors are as follows; R.A. Burks: character definition, species concept development; key development, imaging, capture of specimen data, manuscript preparation, phylogenetic analysis and illustration; L. Masner: specimen acquisition, and generic overview; N.F. Johnson: generic concept development, software and database development and manuscript preparation; A.D. Austin: initial species concept development, manuscript preparation, and taxonomic overview.

## Materials and methods

Specimens examined were provided by the following collections: The American Entomological Institute, Gainesville, Florida, USA (AEIC)^1^; American Museum of Natural History, New York, NY (AMNH)^2^; Australian National Insect Collection, Canberra, Australia (ANIC)^3^; The Natural History Museum, London, United Kingdom (BMNH)^4^; Canadian National Collection of Insects, Arachnids and Nematodes, Ottawa, Canada (CNCI)^5^; Florida State Collection of Arthropods, Gainesville, FL (FSCA)^6^; Institut Royal des Sciences Naturelles de Belgique, Bruxelles, Belgium (ISNB)^7^; Museum of Comparative Zoology, Harvard University, Cambridge, Massachusetts, USA (MCZC)^8^; Muséum National d’Histoire Naturelle, Paris, France (MNHN)^9^; C.A. Triplehorn Insect Collection, Ohio State University, Columbus, Ohio (OSUC)^10^; Queensland Primary Industries Insect Collection, Brisbane, Australia (QDPC)^11^, Queensland Museum, Brisbane, Australia (QMBA)^12^, Royal Museum of Central Africa, Tervuren, Belgium (RMCA)^13^; Nationaal Natuurhistorisch Museum, Leiden, Netherlands (RMNH)^14^; Royal Ontario Museum, Toronto, Canada (ROME)^15^; South African National Collection of Insects, Pretoria, South Africa (SANC)^16^; Ukrainian Academy of Sciences, Kiev, Ukraine (UASK)^17^; National Museum of Natural History, Washington, DC (USNM)^18^; Waite Insect and Nematode Collection, Adelaide, Australia (WINC)^19^. Some specimens will be deposited in other collections where noted, depending on specimen collection agreements: Invertebrate Systematics and Diversity Facility (University of Peradeniya, Sri Lanka, ISDF)^20^; Museum Zoologicum Bogoriense (MBBJ)^21^; Queen Sirikit Botanic Garden (QSBG)^22^; Universiti Kebangsaan Malaysia, Selangor, Bangi (UKMB)^23^.

This revision is a product of the Platygastroidea Planetary Biodiversity Inventory, funded by the U.S. National Science Foundation (N.F. Johnson, Ohio State University; Andy Austin, University of Adelaide; Principal Investigators). An objective of this project is to use biodiversity informatics resources to accelerate taxonomic work, making real-time collaboration possible. Data associated with specimens examined in this study can be accessed at hol.osu.edu and entering the unique specimen identifier (e.g. OSUC 247918) in the search form. Life science identifiers (LSIDs) can be resolved at http://lsid.tdwg.org (i.e. urn:lsid:zoobank.org:act: 99E3E72E-DA88-4740-9ECB-2D03BCD1DACE).

**Terminology**. Morphological terminology follows Mikó et al. (2007) except where noted. Antennal terminology follows Bin (1981). Anteclypeus and postclypeus are used sensu Dangerfield et al. (2001). Dorsal epomial carina and vertical epomial carina were mentioned by Masner and Johnson (2007) and are here used as illustrated by Talamas et al. (2011). Ovipositor terminology is used as described by Austin and Field (1997). “Middle genal carina” is the largest carina subparallel to the eye but between the genal carina and the carina immediately encircling the eye; it has proven to be recognizable as homologous (when present) in *Oxyscelio*. T1 midlobe refers to the raised antero-medial area of T1 that is flanked by depressed lateral areas. This is usually flat and only weakly elevated in *Oxyscelio*, and therefore is not strictly the same as a T1 horn, but a T1 midlobe can be expressed as a T1 horn.

Surface sculpture terminology referring to repeated sculptural elements follows Eady (1968) when possible, with a novel set of designations and distinctions to increase specificity and descriptive value. Diminutive terms such as “foveolate” and “rugulose” were avoided because of a nearly total lack of criteria for separating them from non-diminutive alternatives. “Major” surface sculpture is here treated as repeated sculptural patterns that interact with seta placement. It does not include non-repeated elements or those which are repeated only once due to bilateral symmetry. Umbilicate-foveate sculpture refers to rounded crater-like sculptural elements, each surrounding a setiferous pit, with each fovea being much larger than its setiferous pit and spatially separated from that pit. Umbilicate-punctate sculpture indicates that no sculptural element accompanies the setiferous pit. Rugose sculpture refers to branching or wrinkling elevations that flank setiferous pits but do not fully surround them. Rugose sculpture can coexist with umbilicate sculpture in the same area of the sclerite, in which case the rugae occur on spaces between umbilicate sculptural elements. Note that “rugose” refers to a distribution of sculptural elements, and therefore can be “irregular” or “regular” even though rugae (the elements themselves) are by definition at least slightly irregular. Where both umbilicate-foveate and umbilicate-punctate sculpture are reported for the same sclerite, this should be interpreted as variable sculpture where some setiferous pits are surrounded by foveae while others are not. Under this scheme, “major” surface sculpture cannot occur in any part of the sclerite that lacks setae.

“Microsculpture” refers to repeated tiny sculptural elements that do not interact with seta placement. Microsculpture can occur on “major” sculptural elements, such as on rugae and on all surfaces of foveae. Punctate microsculpture refers to tiny round pits that do not bear setae. Granulate microsculpture refers to sculpture that is similar to that of leather or skin, with areas enclosed by tiny grooves (= sunken septa). Microsculpture can occur in areas that lack setae.

Sculptural terms for repeated sculpture that are not included in the above categories are 1) “carinae” which refers to elevations that are sharp and not branched or wrinkled, 2) “striae” which refers to repeated elevations that are not sharp and do not branch or exhibit wrinkling. These sculptural elements do not interact with setiferous pit placement, but umbilicate sculpture can occur between them. While alternative logic may suggest that rugose sculpture is better classed within this category, this choice was avoided because rugose sculptural patterns did apparently interact with umbilicate sculptural patterns. For the occipital carina, “crenulate” means that short carinae radiate from the occipital carina. For carinae in general: the carina may be described using the phrase “as a ruga” if it is expressed as a wrinkled and/or irregularly meandering elevation.

**Illustrations and data citations**.Photographs were taken using one of the following systems: 1) Visionary Digital BK+ Imaging System, November 2010 model, with either a K2 Long Distance Microscope or a 65 mm varifocal lens; 2) Synoptics, Ltd. system using a Leica Z16 APO microscope and a JVC KY-F75U 3-CCD camera; or 3) GT EntoVision Mobile Imaging System. Source photos were stacked using Zerene Stacker version 1.04 or Auto-Montage Pro version 5.01.0005, and enhanced using Adobe Photoshop CS5 or CS6.

**Phylogenetic analysis**.A New Technology Search at initial level 95 was performed using TNT (Tree analysis using New Technology) version 1.1 (Goloboff et al. 2003, 2008) on a subset of 28 characters that were deemed by the primary author to be phylogenetically valuable (see Appendix III for matrix and characters used). Implied weighting (K=2) was used to produce results with more resolution. Bootstrapping was performed with 1,000 replicates using the same settings. *Bracalba cuneata* Dodd was used as an outgroup for the analyses (specimens OSUC 238172, OSUC 238164), chosen because of morphological similarity between *Oxyscelio* and *Bracalba*.

## Taxonomy

### Oxyscelio

Kieffer

urn:lsid:zoobank.org:act:99E3E72E-DA88-4740-9ECB-2D03BCD1DACE

urn:lsid:biosci.ohio-state.edu:osuc_concepts:529

http://species-id.net/wiki/Oxyscelio

Oxyscelio Kieffer, 1907: 310. Original description. Type: *Oxyscelio foveatus* Kieffer, by monotypy; Brues 1908: 46 (diagnosis, list of species); Kieffer 1910b: 62, 68 (description, key to subgenera, keyed); Kieffer 1913a: 224 (description); Kieffer 1926: 261, 267 (description, keyed, key to species); Dodd 1931: 72 (diagnosis, synonymy, list of species, key to related groups); Mani 1941: 25 (catalog of species of India); Muesebeck and Walkley 1956: 377 (citation of type species); Masner 1976: 22, 25 (description, keys to separate *Baryconus* Foerster, *Bacalba* Dodd, *Chromoteleia* Ashmead, *Oxyscelio* Kieffer); De Santis 1980: 311 (catalog of species of Brazil); Galloway and Austin 1984: 7, 8, 13 (list of species described from Australia, keyed); Johnson 1992: 451 (catalog of world species); Austin and Field 1997: 18, 68 (structure of ovipositor system, discussion of phylogenetic relationships, genus misplaced in Baryconini); Lê 2000: 32, 39 (keyed, description); Rajmohana 2006: 116 (keyed); Kononova and Fursov 2007: 61 (description); Kononova and Fursov 2007: 103 (description); Kononova and Kozlov 2008: 21, 190 (description, keyed, key to species of Palearctic region).
Dicroteleia Kieffer, 1908: 92. Original description. Type: *Dicroteleia rugosa* Kieffer, by monotypy. Synonymized by Dodd (1931); Kieffer 1926: 267, 387 (description, keyed, key to species); Mani 1941: 25 (catalog of species of India); Muesebeck and Walkley 1956: 346 (citation of type species); Baltazar 1966: 179 (catalog of species of the Philippines).
Chromoteleia (*Oxyscelio*): Kieffer 1910a: 312 (key to species, keyed); Kieffer 1910b: 68, 69 (description, list of species, keyed).
Oxyscelio (*Dicroteleia*): Kieffer 1910b: 68 (description, list of species, keyed).
Camptoteleia Kieffer, 1913b: 387. Original description. Type: *Camptoteleia carinata* Kieffer, designated by Kieffer (1926) (key to species of the Philippines). Synonymized by Dodd (1931); Kieffer 1914: 296 (key to species of the Philippines); Kieffer 1916: 64, 171 (key to new species described from the Philippines); Kieffer 1926: 267, 379 (description, keyed, key to species); Baltazar 1966: 177 (catalog of species of the Philippines).
Xenoteleia Kieffer, 1913b: 390. Original description. Type: *Xenoteleia flavipennis* Kieffer, by monotypy and original designation. Synonymized by Dodd (1931); Kieffer 1926: 270, 427 (description, keyed); Muesebeck and Walkley 1956: 408 (citation of type species); Baltazar 1966: 181 (catalog of species of the Philippines).

#### Description.

Body length: 2.6–7.1 mm.

Head shape in dorsal view: weakly transverse, width approximately 1.5x greatest length; subquadrate. Hyperoccipital carina: absent; present. Occipital carina: present, complete medially; present, broadly interrupted medially. Occipital carina sculpture: crenulate. Ocular ocellar line (OOL): OOL < 0.5 ocellar diameter (OD). Dorsal area of frons: convex, without frontal shelf. Antennal scrobe shape: present, unmargined; scrobe margined by carina. Frons sculpture: umbilicate-punctate, with transverse carinae within scrobe; scrobe largely smooth, otherwise with transverse carinae. Submedian carina: absent. Orbital carina: absent. Inner orbits: diverging ventrally. Interocular space(IOS)/Eye height (EH): IOS distinctly less than EH. Interantennal process: triangular in lateral view. Central keel: absent. Antennal foramen opening: oriented laterally on interantennal process. Facial striae: present. Malar sulcus: present. Compound eye size: not significantly reduced. Compound eye setation: absent. Gena: weakly convex, receding behind posterior orbit; convex, distinctly produced behind eye. Clypeus shape: narrow, slightly convex medially, lateral corner not produced. Apical margin of clypeus: with small median point. Labrum: not visible. Mandibular teeth: apex with 2, acute, subequal teeth. Arrangement of mandibular teeth: transverse. Number of maxillary palpomeres: 4. Shape of maxillary palpomeres: cylindrical. Number of labial palpomeres: 2.

Number of antennomeres in female: 12. Number of antennomeres in male: 12. Insertion of radicle into A1: parallel to longitudinal axis of A1. Shape of A1: more or less cylindrical, not flattened. Length of A3 of female: subequal to length of A2; distinctly longer than A2. Number of clavomeres in female antenna: 7; 0. Claval formula of female antenna: A12-A7/1-2-2-2-2-1; A12-A6/1-2-2-2-2-2-2. Arrangement of doubled multiporous plate sensilla on female clava: in longitudinal pairs. Tyloid distribution on male antenna: A5 only. Shape of male flagellum: filiform.

Mesosoma shape in dorsal view: longer than wide. Mesosoma shape in lateral view: longer than high. Medial portion of transverse pronotal carina: weakly indicated laterally; absent. Posterior apex of pronotum in dorsal view: straight, bifid apically to articulate with tegula. Vertical epomial carina: present. Dorsal epomial carina (lateral portion of transverse pronotal carina of Vilhelmsen et al. 2010): present. Anterior face of pronotum: oblique, visible dorsally, short. Lateral face of pronotum: deeply concave below dorsal pronotal superhumeral sulcus. Netrion: present. Netrion shape: open ventrally. Anterior portion of mesoscutum: vertical, flexed ventrally to meet pronotum. Mesoscutum shape: pentagonal in dorsal view, posterolateral corner rounded. Skaphion: absent. Notaulus: present, percurrent. Parapsidal lines: present; absent. Anteroadmedial lines: present. Scutoscutellar sulcus: well-developed, narrow. Shape of mesoscutellum: quadrate to trapezoidal. Armature of mesoscutellum: absent. Surface of mesoscutellum: convex throughout. Median longitudinal furrow on mesoscutellum: absent. Shape of axillula: small, dorsal margin sinuate. Metascutellum: clearly differentiated. Metascutellar armature: produced medially into short, shallowly bidentate process; produced into broad flattened plate; produced into narrow, flat, apically blunt process. Metascutellar setation: absent; present dorsally and ventrally. Extent of metasomal depression of propodeum: percurrent, extending anteriorly to anterior margin of propodeum. Lateral propodeal projection: well-developed, extending clearly beyond anterior margin of T1. Mesopleural carina: present across sclerite; absent or strongly abbreviated, present only near mid coxa. Mesal course of acetabular carina: projecting as small spur anteriorly, not long enough to intercede between fore coxae. Mesopleural pit: absent. Sternaulus: absent. Posterodorsal corner of mesopleuron: rounded anteriorly.

Number of mid tibial spurs: 1. Number of hind tibial spurs: 1. Dorsal surface of hind coxa: smooth. Hind tibia shape: cylindrical, ecarinate. Trochantellus: present.

Wing size of female: macropterous. Wing size of male: macropterous. Tubular veins in fore wing: present. Bulla of fore wing R: absent. Extent of marginal venation of fore wing: R1 reaching and ending at costal margin; distinct marginal or postmarginal veins present. Origin of r-rs in fore wing: arising before (basad of) R/R1 attains costal margin. Structure of basal vein (Rs+M) in fore wing: spectral. Structure of R in hind wing: elongate, extending to costal margin; abbreviated, not attaining costal margin.

Number of externally visible terga in female: 6. Number of externally visible sterna in female: 6. Number of externally visible terga in male: 8. Number of externally visible sterna in male: 7. Shape of metasoma: acuminate, widest submedially. Laterotergites: present, narrow. Laterosternites: present. T1 of female: raised medially into low, rectangular or subelliptical platform, laterally depressed. Relative size of metasomal segments: T2 distinctly largest; T2 and T3 distinctly larger, subequal in size. Terga with basal crenulae: T1, T2. Sublateral carinae on tergites: present on T1. Median longitudinal carina on metasomal terga: absent. Shape of female T6: flattened. Shape of posterior margin of male T7: straight; incised medially. Anterior margin of S1: protruding anteriorly as short sharp extension of median longitudinal carina. Felt fields: absent. Ovipositor type: *Scelio*-type (Austin and Field 1997).

**Comments. ***Oxyscelio* is a very distinctive genus particularly because of the morphology of the face, vertex and fore wing venation. The genus is highly diverse and comprises in excess of 200 species. It has been collected from a large range of habitats from rainforest, open dry forest, grasslands to more open, dry environments including the malle and semi-arid zone of Australia. Species have been collected using a variety of standard collecting techniques for small parasitic Hymenoptera, but they can be particularly numerous in yellow pantraps, even in closed habitats, indicating that many species may be living close to the ground.

**Biology.** Although there are a large number of species known from Asia and Australia there are apparently no available rearing records from hosts identified beyond ordinal level (see Kononova and Fursov 2007, for photograph of egg of orthopteran host of *Oxyscelio perpensus* Kononova). However, given the large size of most species, the diversity of habitats in which they have been collected, and the structure of the ovipositor, we presume that all *Oxyscelio* species parasitise orthopteran eggs of some type.

**Distribution**. *Oxyscelio* has been recorded from Africa (Cameroon, Central African Republic, Democratic Republic of the Congo, Côte d’Ivoire, Gabon, Ghana, Guinea, Kenya, Malawi, Nigeria, Rwanda, Sierra Leone, Somalia, Tanzania, Togo, Uganda), the Indo-Malayan region (Brunei, Christmas Island, India, Indonesia, Laos, Malaysia, Philippines, Singapore, Sri Lanka, Taiwan, Thailand, Vietnam), the eastern Palearctic (mainland China, Japan, Nepal, South Korea), Australasia and the south-west Pacific (mainland Australia, Tasmania, Fiji, Lord Howe Is., New Britain, New Caledonia, Papua New Guinea, Solomon Islands, Vanuatu).

**Phylogenetic relationships.** Masner (1976) postulated that *Oxyscelio* is related to *Bracalba* Dodd, *Chromoteleia* Ashmead and *Baryconus* Foerster, and he provided a key to separate them. Based on the structure of the ovipositor system it is unlikely that *Baryconus* is related to this group of genera given it has a *Ceratobaeus*-type system, where the other genera all have a *Scelio* type ovipositor system (Austin and Fields 1997). The molecular phylogenetic study of Murphy et al. (2007) included three of these genera, *Oxyscelio*,* Chromoteleia* and *Baryconus*, and none showed a sister-group relationship to each other, although the support on the branches that linked these genera were far from robust. Two of us (NFJ and ADA) are currently coordinating a significantly expanded molecular analysis of the Platygastroidea involving additional sequence data and a trebling of taxa, and this should help resolved the relationships among these and other genera.

**Figure 1.**
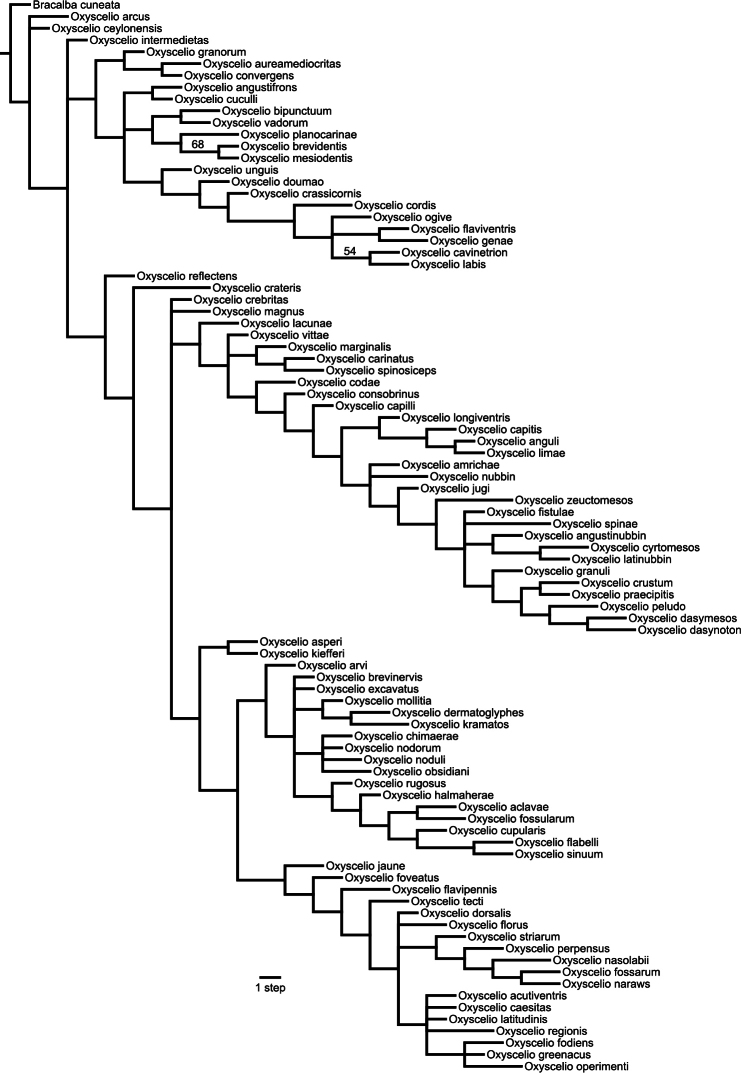
Strict consensus phylogram of four most parsimonious trees for Indo-Malayan and Palearctic species of *Oxyscelio* using TNT New Technology with Implied Weighting. Search with set initial level = 95, best score = 16.98. Bootstrap support values above 50% indicated above branches, found using TNT new technology search (set initial level = 95).

The species level phylogeny generated as a part of this study (Fig. 1) does not always uphold monophyly of the species groups described below. The authors do not see this as a major problem, as the species groups are informal groupings that are not necessarily meant to be strictly monophyletic. They are meant to be useful for species diagnostics, but can be seen as potentially valid alternatives to the included phylogeny.

##### Species Groups of Oxyscelio

For the Indo-Malayan and Palearctic fauna of *Oxyscelio* we recognize 13 species groups. These groups are discussed below to indicate intuitively our perception of the structure within the genus and to serve as an aid in specimen identification.

##### *Oxyscelio carinatus* Species Group

**Characteristics:** Frontal depression flat or nearly so. Hyperoccipital carina complete as a strong ruga, continuous with the anteriormost genal carina, laterally not connected with occipital carina. Occipital carina complete or incomplete, but without strong lateral corners. Metascutellum with a pair of subapical dorsal setae, concave dorsally, slightly emarginate apically with rounded posterolateral lobes. T7 in males with acuminate posterolateral corners.

**Comments**: The *carinatus*-group is very similar to the *cuculli*-group, but differs in that the hyperoccipital carina is defined by a ruga and in having a deeper frontal depression. The *Oxyscelio mesiodentis*-complex within the *cuculli*-group has a much more densely setose and differently shaped metascutellum than in the *carinatus*-group. The general trend towards sculptural reduction in Philippine species (a more pronounced reduction occurs in species from the Maluku Islands of Indonesia) leads one to consider the possibility that the *carinatus*-group could be weakly sculptured species of the *cuculli*-group. However, it does not seem proper to lump these groups without additional data supporting this hypothesis. Another possibility exists, that the *carinatus*-group could be closely related to the *dasymesos*-group, as both groups contain species with a setose metascutellum and nearly flat frontal depression.

Includes: *Oxyscelio carinatus*, *Oxyscelio praecipitis*, *Oxyscelio spinosiceps*, *Oxyscelio vittae*.

##### *Oxyscelio crateris* Species Group

**Characteristics:** Hyperoccipital carina complete, continuous with the anteriormost genal carina, laterally connected with occipital carina by a distinct longitudinal carina or elevation; area between hyperoccipital and occipital carinae slightly sunken and crater-like. Metascutellum about as long as broad, concave dorsally and with little or no median sculpture, rounded apically. T7 in males without posterolateral spines.

**Comments**:The *crateris*-group contains a few species with a crater-like area, between the occipital and hyperoccipital carinae, that is fully outlined by carinae. This area also has distinctive sculpture that is different from that of surrounding areas. Some members of the *latitudinis*-group may have a similarly weakly concave or partially outlined crater-like area as well, but these species have a very different, broad and strongly sculptured metascutellum.

Includes:*Oxyscelio cordis*, *Oxyscelio crateris*, *Oxyscelio spinae*.

##### *Oxyscelio crebritas* Species Group

**Characteristics:** Hyperoccipital carina absent or weakly indicated by rugae, laterally not connected with occipital carina. Occipital carina complete or incomplete, but without strong lateral corners. Frons without oblique flange; frontal depression without transverse carinae or grooves in ventral half. Metascutellum medially concave and smooth or with transverse carinae. T7 in males usually with sharp posterolateral corners, rarely with short spines or without spines.

**Comments**:The *crebritas*-group contains many very similar species differing in subtle ways. Most members of this group have a radicle that is darker than the scape, but this feature is variable in many species. The *florus*-group differs in having longitudinal metascutellar rugae, instead of transverse carinae. The *noduli*-group is similar but has a much more strongly sculptured frontal depression.

Includes:*Oxyscelio amrichae*, *Oxyscelio asperi*, *Oxyscelio brevinervis*, *Oxyscelio capilli*, *Oxyscelio capitis*, *Oxyscelio codae*, *Oxyscelio consobrinus*, *Oxyscelio crebritas*, *Oxyscelio excavatus*, *Oxyscelio genae*, *Oxyscelio granuli*, *Oxyscelio jugi*, *Oxyscelio kiefferi*, *Oxyscelio lacunae*, *Oxyscelio longiventris*, *Oxyscelio mollitia*, *Oxyscelio reflectens*.

##### *Oxyscelio cuculli* Species Group

**Characteristics:** Hyperoccipital carina complete and strong, continuous with the anteriormost genal carina, laterally not connected with occipital carina. Occipital carina complete or incomplete, but without strong lateral corners. Metascutellum concave dorsally and with little or no median sculpture, incised or slightly emarginate apically. T7 in males variable, some species with strong posterolateral spines.

**Comments**: The *cuculli*-group has several distinctive species with a sharp hyperoccipital carina and strongly concave frontal depression that is more or less hood-like. There are some other species that possess these traits in less-developed ways, but which can be linked with this group through transformation series. These species make distinction from the *crebritas*-group and *carinatus*-group especially difficult. Because of this, it is useful to divide this group into three species complexes that can be more consistently defined:

*Oxyscelio convergens* Species Complex: Metascutellum long, weakly emarginate, nearly flat, not setose. Anterior portion of metasomal depression long and exposed dorsally; with long and narrowly separated lateral propodeal carinae, often with a median carina between them. Includes:*Oxyscelio aureamediocritas*, *Oxyscelio bipunctuum*, *Oxyscelio convergens*, *Oxyscelio kramatos*, *Oxyscelio marginalis*, *Oxyscelio vadorum*.

*Oxyscelio cuculli* Species Complex: Metascutellum short, strongly emarginate with dorsally protruding posterolateral corners, not setose. Anterior portion of metasomal depression short and weakly developed, hidden from dorsal view; lateral propodeal carinae short and variably separated anteriorly. Includes:*Oxyscelio angustifrons*, *Oxyscelio cuculli*, *Oxyscelio granorum*, *Oxyscelio intermedietas*, *Oxyscelio nubbin*.

*Oxyscelio mesiodentis* Species Complex: Metascutellum setose dorsally, weakly emarginate and nearly flat. Anterior portion of metasomal depression short, variably sculptured. Includes:*Oxyscelio arcus*, *Oxyscelio brevidentis*, *Oxyscelio ceylonensis*, *Oxyscelio crassicornis*, *Oxyscelio crustum*, *Oxyscelio doumao*, *Oxyscelio mesiodentis*, *Oxyscelio unguis*.

##### *Oxyscelio dasymesos* Species Group

**Characteristics:** Hyperoccipital carina incomplete or indicated by poorly defined rugae, laterally not connected with occipital carina. Occipital carina complete or incomplete, but without strong lateral corners. Metasomal depression setose. T7 in males with acuminate posterolateral corners.

**Comments**:A setose metasomal depression does not occur in any other Asian species of *Oxyscelio*. The *dasymesos*-group is otherwise difficult to compare with other *Oxyscelio* species groups, but it bears some general resemblance to the *carinatus*-group and *crebritas*-group.

Includes:*Oxyscelio dasymesos*, *Oxyscelio dasynoton*.

##### *Oxyscelio florus* Species Group

**Characteristics:** Hyperoccipital carina absent or indicated by poorly defined rugae, laterally not connected with occipital carina. Occipital carina complete or incomplete, but without strong lateral corners. Frons without oblique flange. Metascutellum with longitudinal rugae and without any strong transverse carinae. T2 without longitudinal depressions or strong curved striae.

**Comments**:The *florus*-group contains species that are similar to the *crebritas*-group in having a dark radicle and uniformly curved occipital carina, but differ in having a rugose metascutellum and a generally longer metasoma. The *latitudinis*-group is also similar to this group based on most of the above-mentioned features, but differs in having an occipital carina with strong lateral corners.

Includes:*Oxyscelio arvi*, *Oxyscelio dermatoglyphes*, *Oxyscelio florus*, *Oxyscelio jaune*, *Oxyscelio regionis*.

##### *Oxyscelio fossarum* Species Group

**Characteristics:** Hyperoccipital carina incomplete or indicated by weak rugae. Occipital carina with sharp protruding lateral corners. T2 with long sublateral depressions.

**Comments**:The *fossarum*-group is similar to the *foveatus*-group, *latitudinis*-group, and *striarum*-group, but is distinguished by the T2 depressions that occur in females (and in males of some species). These groups differ in metascutellar form as well, with the *fossarum*-group having a generally narrower metascutellum. The defining feature of this group can be difficult to discern, but is best verified by finding the strong medial borders of the depressions.

Includes:*Oxyscelio aclavae*, *Oxyscelio acutiventris*, *Oxyscelio cyrtomesos*, *Oxyscelio fistulae*, *Oxyscelio fodiens*, *Oxyscelio fossarum*, *Oxyscelio fossularum*, *Oxyscelio rugosus*, *Oxyscelio zeuctomesos*.

##### *Oxyscelio foveatus* Species Group

**Characteristics:** Hyperoccipital carina incomplete or indicated by weak rugae. Occipital carina with strong lateral corners. Ventral frons with oblique flange. Metascutellum tiny and concave, or broad and convex, or elongate with a smooth channel. T2 without longitudinal depressions or strong curved striae.

**Comments**:The *foveatus*-group likely is a non-monophyletic group containing species with an oblique facial flange and an occipital carina with protruding lateral corners, but with none of the defining features of some other species groups. Species with longitudinal T2 depressions, but which would otherwise agree with this group, have been placed in the *fossarum*-group.Other species with an oblique facial flange occur in the *carinatus*-group, *crateris*-group,and *cuculli*-group, but differ strongly from these species.

Includes:*Oxyscelio angustinubbin*, *Oxyscelio cupularis*, *Oxyscelio foveatus*, *Oxyscelio greenacus*, *Oxyscelio latinubbin*, *Oxyscelio nasolabii*, *Oxyscelio operimenti*.

##### *Oxyscelio latitudinis* Species Group

**Characteristics:**Lower frons without oblique flange. Hyperoccipital carina incomplete or indicated by weak rugae. Occipital carina with sharp protruding lateral corners. Metascutellum broad, almost always rugose. T2 without sublateral depressions or strong curved striae.

**Comments**:The *latitudinis*-group is essentially negatively defined among *Oxyscelio* that have strong lateral corners of the occipital carina. The best distinctive feature of this group is the broad, rugose metascutellum of most species, but a few have a narrower metascutellum that more closely approaches that of the *fossarum*-group. Most members of this group have a metallic green luster, but this is lost in some specimens. Except where noted in species descriptions, color seems to be a highly unreliable character for identification of *Oxyscelio*.

Includes:*Oxyscelio dorsalis*, *Oxyscelio latitudinis*, *Oxyscelio naraws*, *Oxyscelio peludo*, *Oxyscelio perpensus*.

##### *Oxyscelio limae* Species Group

**Characteristics:** Hyperoccipital carina incomplete or weakly indicated by rugae. Occipital carina complete medially, without sharp protruding lateral corners. Mesoscutum anteriorly very steep. Postmarginal vein absent. Metascutellum variable but without dorsally protruding posterolateral corners.

**Comments**:The *limae*-group contains species from India and Sri Lanka, all with a strongly elevated and anteriorly steep mesoscutum. These species strongly resemble the *crebritas*-group, but differ in having very short fore wing venation with no sign of a postmarginal vein. Some Australian species, including *Oxyscelio montanus* (Dodd) strongly resemble this group, but differ in having a short metascutellum with dorsally protruding posterolateral corners. Tiny but sharp and slightly protruding posterolateral corners of T4 or T5 in females of *Oxyscelio limae* and *Oxyscelio anguli* indicate that the *limae*-group may be the closest relative of an otherwise Australian clade containing *Oxyscelio montanus* and *Oxyscelio mirellus* (Dodd).

Includes:*Oxyscelio anguli*, *Oxyscelio flaviventris*, *Oxyscelio limae*.

##### *Oxyscelio noduli* Species Group:

**Characteristics:** Hyperoccipital carina absent or weakly indicated by rugae, laterally not connected with occipital carina. Occipital carina complete, but without strong lateral corners. Frons without oblique flange; frontal depression crossed by many carinae. Metascutellum medially concave and smooth.

**Comments**:The *noduli*-group contains some species that are resemble the *latitudinis*-group in metasomal length and frontal depression sculpture, but which have a small and medially smooth metascutellum and an occipital carina without strong lateral corners. The latter features are similar to those in the *crebritas*-group, and therefore these species may be phylogenetically intermediate between that group and the *latitudinis*-group. Alternatively, they may be reduced apomorphic members of the *latitudinis*-group.

Includes:*Oxyscelio chimaerae*, *Oxyscelio nodorum*, *Oxyscelio noduli*.

##### *Oxyscelio ogive* Species Group

**Characteristics:** Hyperoccipital carina incomplete or indicated by rugae. Occipital carina complete medially, but with sharp lateral corners and concave medial sections that meet at a median peak.

**Comments**:Members of the *ogive*-group superficially resemble the *crebritas*-group, but differs in the sinuate occipital carina with sharp lateral corners. It differs from the *latitudinis* group in having a sharp and rounded submedian carina.

Includes:*Oxyscelio cavinetrion*, *Oxyscelio flabelli*, *Oxyscelio labis*, *Oxyscelio ogive*, *Oxyscelio sinuum*.

##### *Oxyscelio striarum* Species Group

**Characteristics:**Hyperoccipital carina incomplete or indicated by rugae. Occipital carina with sharp lateral corners. Metascutellum rugose. T2 (at least) in females with strong curved longitudinal striae submedially that flank a triangular area without striae.

**Comments**:The *striarum*-group is similar to the *latitudinis*-group, but differs in the strong curved striae of T2 and T3 in females. Males may be difficult to recognize, because those of *Oxyscelio caesitas* reveal that they do not possess these strong striae. They do have slightly more distinct sublateral striae of S2 and S3, but these striae are straight and do not distinctly differ from those of other species groups.

Includes:*Oxyscelio caesitas*, *Oxyscelio striarum*.

##### Species unplaced to group

*Oxyscelio flavipennis*, *Oxyscelio halmaherae*, *Oxyscelio magnus*, *Oxyscelio obsidiani*, *Oxyscelio planocarinae*, *Oxyscelio tecti*.

##### Key to Indo-Malayan and eastern Palearctic species of *Oxyscelio*

**Table:** 

1	Metasomal depression setose (Figs 163, 167): *Oxyscelio dasymesos* Species Group	2
–	Metasomal depression not setose (Fig. 141)	3
2	Metascutellum dorsally setose (Fig. 167)	*Oxyscelio dasynoton* Burks, sp. n.
–	Metascutellum not dorsally setose (Fig. 163)	*Oxyscelio dasymesos* Burks, sp. n.
3	Metascutellum setose dorsally (Figs 83, 128, 140, 364, 405)	4
–	Metascutellum not setose dorsally (Figs 146, 236, 255)	16
4	Metascutellum dorsally nearly flat, apically convex (Fig. 364)	*Oxyscelio peludo* Burks, sp. n.
–	Metascutellum dorsally concave, apically emarginate (Figs (Figs 83, 128, 140, 405)	5
5	Frontal depression flat or nearly so; submedian carina absent or indicated only dorsally by a weak elevation or short carina (Figs 84, 379, 406). Philippines: *Oxyscelio carinatus* Species Group	6
–	Frontal depression deeply concave; submedian carina strong, extending laterally to form a protruding hood-like structure (Figs 59, 142, 317) *Oxyscelio mesiodentis* Species Complex 9
6	Small and tooth-like oblique flange present between antennal foramina and eye (Fig. 406)	*Oxyscelio spinosiceps* (Kieffer)
–	Ventral frons without oblique flange (Figs 84, 379)	7
7	Mesoscutum anteriorly tall and very steep, almost at a right angle (Fig. 377). Metascutellum with many (>5) dorsal setae (Fig. 378)	*Oxyscelio praecipitis* Burks, sp. n.
–	Mesoscutum sometimes anteriorly weakly curved, not nearly at a right angle (Fig. 430). Metascutellum with 2-4 dorsal setae (Figs 83, 431)	8
8	Radicle darker than scape (Fig. 84). Female: fore wing long enough to reach to or beyond T6 (Fig. 85)	*Oxyscelio carinatus* (Kieffer)
–	Radicle same color as scape (Figs 430, 432) . Female: fore wing not long enough to reach T6 (Fig. 433)	*Oxyscelio vittae* Burks, sp. n.
9	Straight, vertical facial carina present extending dorsoventrally alongside antennal foramen (Fig. 317). Male: submedian carina with a small protrusion medially that intercedes between pedicels when antenna at rest (Figs 60, 320)	10
–	Vertical facial carina absent (Figs 142, 182). Male: submedian carina without median protrusion (Fig. 127)	11
10	1st metatarsomere over 1.1x as long as metatarsomeres 2–5 combined. Body long: females over 4 mm, males over 3.4 mm	*Oxyscelio mesiodentis* Burks, sp. n.
–	1st metatarsomere less than 1.1x as long as metatarsomeres 2–5 combined Body short: females less than 4 mm, males less than 3.3 mm	*Oxyscelio brevidentis* Burks, sp. n.
11	Female: T1 midlobe with 5 straight and anteriorly complete longitudinal carinae (as in Fig. 85). Male: A5 tyloid carina-like, not expanded (as in Fig. 136); T7 always with sharp and elongate posterolateral corners (Fig. 144)	12
–	Female: T1 midlobe with 4 anteriorly complete longitudinal carinae (Figs 37, 98), or with an irregular curved set of carinae that are difficult to discern medially (Figs 183, 422, 425). Male: A5 tyloid expanded, teardrop-shaped or sinuate (Figs 36, 99, 424); T7 with rounded or sharp posterior corners, but these not narrow and elongate (Figs 37, 425)	13
12	Occipital carina connected to hyperoccipital carina by a median carina (as in Fig. 356)	*Oxyscelio crassicornis* (Kieffer)
–	Occipital carina not connected to hyperoccipital carina (Fig. 140)	*Oxyscelio crustum* Burks, sp. n.
13	Postmarginal vein absent or very short: less than 1/3 stigmal vein length. Female: A4 longer than broad (Fig. 180)	*Oxyscelio doumao* Burks, sp. n.
–	Postmarginal vein long: more than 1/3 stigmal vein length. Female: A4 usually as broad or broader than long (Figs 35, 96; exception: *Oxyscelio unguis*, Figs 420, 423)	14
14	Medial mesoscutum with longitudinal rugae posteriorly (Fig. 97). Male: T1 midlobe with 3 longitudinal carinae	*Oxyscelio ceylonensis* (Dodd)
–	Medial mesoscutum without longitudinal rugae (Figs 34, 421). Male: T1 midlobe with 4 or 5 longitudinal carinae (Figs 37, 425)	15
15	Mesoscutum and mesoscutellum forming a strong arch in lateral view (Fig. 32). Female: T1 midlobe longitudinal carinae in pairs (with no median carina) but not broadly separated medially (as in Fig. 37)	*Oxyscelio arcus* Burks, sp. n.
–	Mesoscutum and mesoscutellum not forming such a steep arch (Fig. 420). Female: T1 midlobe longitudinal carinae in two broadly separated pairs with sometimes an irregular or split median carina between the pairs (Fig. 422)	*Oxyscelio unguis* Burks, sp. n.
16	Hyperoccipital carina indicated by a sharp carina but not connected to occipital carina laterally (Figs 58, 146, 318)	17
–	Hyperoccipital carina absent, indicated by one or more rounded rugae (Figs 11, 39), or connected to occipital carina laterally (Figs 132, 403)	27
17	Ventral frons with oblique flange between antennal foramen and eye (Fig. 347)	*Oxyscelio nubbin* Burks, sp. n.
–	Ventral frons without oblique flange between antennal foramen and eye (Fig. 237)	18
18	Netrion smooth centrally, with only two rows of foveae peripherally (Fig. 312). Frontal depression nearly flat (Fig. 314)	*Oxyscelio marginalis* (Kieffer)
–	Netrion with additional foveae centrally or crossed by rugae (Fig. 145, 235). Frontal depression deeply concave (Fig. 147, 274, 428)	19
19	Metascutellum narrowing posteriorly, truncate or very slightly emarginate apically (Figs 23, 273, 427)	20
–	Metascutellum deeply divided apically (Figs 49, 146)	22
20	Lateral propodeal carinae broadly separated anteriorly (as in Fig. 181). T1 midlobe with 6 longitudinal carinae. Gena roughly sculptured, without granulate sculpture (Fig. 22)	*Oxyscelio angustifrons* Burks, sp. n.
–	Lateral propodeal carinae narrowly separated anteriorly (Figs 275, 427). T1 midlobe with 4 or 5 longitudinal carinae. Gena with extensive granulate sculpture or nearly smooth (Fig. 272, 426)	21
21	Metasomal depression strongly sculptured (Fig. 427). Gena without middle carina or ruga (Fig. 427)	*Oxyscelio vadorum* Burks, sp. n.
–	Metasomal depression not sculptured (Fig. 275). Gena with middle carina or ruga (Fig. 272)	*Oxyscelio kramatos* Burks, sp. n.
22	T1 midlobe with 5 or more anteriorly complete longitudinal carinae (Fig. 49). Occipital carina with a sharply narrowed median peak (Fig. 49). Female: metasomal depression with complete median carina (Fig. 51); A3-A6 longer than broad	*Oxyscelio aureamediocritas* Burks, sp. n.
–	T1 midlobe with 4 anteriorly complete longitudinal carinae (Fig. 148). Occipital carina without median peak (Fig. 146). Female: metasomal depression often with sculpture, but not a complete median carina (Fig. 256); at least A6 broader than long (Fig. 253)	23
23	Metasomal depression with a pair of anterior areoles separated by a tiny median carina. Male: submedian carina with a small protrusion medially, separating pedicels when antenna at rest (as in Fig. 320)	*Oxyscelio bipunctuum* Burks, sp. n.
–	Metasomal depression smooth or with only a single median areole anteriorly (Fig. 256). Male: submedian carina without protrusion medially (as in Fig. 147)	24
24	Metascutellum narrowing apically, with posterolateral corners slightly convergent (Fig. 117)	*Oxyscelio convergens* Burks, sp. n.
–	Metascutellum not narrowing apically, posterolateral corners not convergent (Fig. 146)	25
25	Mesoscutellum without granulate sculpture (Fig. 146). Propodeal carinae anteromedially broadly separated (as in Fig. 97)	*Oxyscelio cuculli* Burks, sp. n.
–	Mesoscutellum posterolaterally with some granulate sculpture (Figs 236, 255–256). Propodeal carinae anteromedially narrowly separated (Fig. 256)	26
26	Mesoscutellum lacking granulate sculpture medially, but posterolaterally with granulate microsculpture (Figs 255–256). Propodeum anteriorly relatively long, its carinae anteromedially not expanded, proceeding for a long distance anteriorly before reaching anterior rim (Fig. 256)	*Oxyscelio intermedietas* Burks, sp. n.
–	Mesoscutellum uniformly sculptured, with strong granulate microsculpture (Figs 236). Propodeum anteriorly shorter, its carinae expanded (Fig. 238)	*Oxyscelio granorum* Burks, sp. n.
27	Ventral frons with oblique carina-like or tooth-like flange between antennal foramen and eye (Figs 7, 335, 361)	28
–	Ventral frons without flange or carina between antennal foramen and eye (Figs 134, 291)	37
28	Female: club not expanded or compact (Fig. 6). Males unknown	*Oxyscelio aclavae* Burks, sp. n.
–	Specimen male, or female with an expanded and compact antennal club (Fig. 284)	29
29	Metascutellum dorsally convex (Figs 246, 360)	30
–	Metascutellum dorsally flat or concave (Figs 152, 226, 400)	31
30	Metascutellum deeply triangularly emarginate apically (Fig. 360). Female: T5, T6 not nearly parallel-sided (Fig. 362)	*Oxyscelio operimenti* Burks, sp. n.
–	Metascutellum nearly truncate apically (Fig. 246). Female: T5, T6 very narrow, nearly parallel-sided (Fig. 248)	*Oxyscelio greenacus* Burks, sp. n.
31	T2 with sublateral longitudinal depressions (as in Fig. 217)	*Oxyscelio rugosus* (Kieffer)
–	T2 without sublateral depressions (Fig. 403)	32
32	Hyperoccipital carina connected laterally with occipital carina (Figs 400, 403). Female: T6 terminating in an elongate, sharp spine (Fig. 402). Male: T7 with rounded posterior corners (Fig. 403)	*Oxyscelio spinae* Burks, sp. n.
–	Hyperoccipital carina not distinctly connected with occipital carina laterally (Fig. 334). Female: T6 not terminating in elongate spine (Fig. 336). male: T7 with sharp posterior corners	33
33	Metascutellum longer than broad, with a narrow median channel (Fig. 285)	*Oxyscelio latinubbin* Burks, sp. n.
–	Metascutellum broader than long, with a median fovea or set of rugae (Figs 226, 334)	34
34	T1 midlobe with 3 anteriorly separate longitudinal carinae (Fig. 336)	*Oxyscelio nasolabii* Burks, sp. n.
–	T1 midlobe with 5 or more anteriorly separate longitudinal carinae, or T1 without separate longitudinal carinae (Figs 29, 152)	35
35	Metascutellum narrow and tiny, smooth medially (Fig. 152)	*Oxyscelio cupularis* (Kieffer)
–	Metascutellum large and broad, rugose medially (Figs 27, 226)	36
36	Frontal depression crossed by 1 (faint) transverse carina (Fig. 227). Female: T1 midlobe with strong anterior horn and no longitudinal carinae	*Oxyscelio foveatus* Kieffer
–	Frontal depression with 3 or more transverse carinae dorsally (Fig. 28). Female: T1 midlobe without anterior horn, with 6 strong and complete longitudinal carinae (Fig. 29)	*Oxyscelio angustinubbin* Burks, sp. n.
37	Occipital carina with sharp, protruding lateral corners (Figs 155–156)	38
–	Occipital carina rounded laterally, without lateral corners (Figs 381–382)	69
38	Mesoscutellar rim medially sharply incised (Fig. 123)	*Oxyscelio cordis* Burks, sp. n.
–	Mesoscutellum apically convex, straight, or broadly concave posteriorly, but rim not incised (Fig. 289)	39
39	Propodeum forming a complete, roughly sculptured arch protruding over the base of T1 (Figs 435, 438)	*Oxyscelio zeuctomesos* Burks, sp. n.
–	Propodeum with at most a slight arch anteriorly, not protruding over the base of T1 (Fig. 289)	40
40	Occipital carina medially flat, with sharp dorso-lateral corners, distinctly indicated throughout (Fig. 375)	*Oxyscelio planocarinae* Burks, sp. n.
–	Occipital carina absent or convex medially	41
41	Netrion anteriorly deeply concave (Fig. 196)	42
–	Netrion not concave (Fig. 351)	43
42	Occipital carina and submedian carina acuminate medially (Figs 89–90). Metascutellum extending beyond anterior margin of propodeum (Fig. 89)	*Oxyscelio cavinetrion* Burks, sp. n.
–	Occipital carina absent medially (Fig. 197), submedian carina rounded medially (Fig. 198). Metascutellum very tiny, hardly extending beyond anterior margin of propodeum (Fig. 197)	*Oxyscelio flavipennis* (Kieffer)
43	Mesoscutum and mesoscutellum smooth and shiny, head and mesosoma without granulate sculpture (Figs 352, 415). Seram	44
–	Mesoscutum and mesoscutellum, if nearly smooth, then strongly granulate (Fig. 328)	45
44	Mesoscutal median carina absent (Fig. 352)	*Oxyscelio obsidiani* Burks, sp. n.
–	Mesoscutal median carina present (Fig. 415)	*Oxyscelio tecti* Burks, sp. n.
45	Metascutellum elongate, extending past propodeal apex (Fig. 307)	*Oxyscelio magnus* (Kieffer)
–	Metascutellum not elongate, not extending past propodeal apex (Figs 289, 356)	45
46	Occipital carina submedially concave, medially acuminate or with a strongly convex middle section (Figs 356, 396). Submedian carina acuminate medially (Fig. 357). *Oxyscelio ogive* Species Group	47
–	Occipital carina and submedian carina absent medially or uniformly rounded medially (Figs 65, 130)	50
47	Metascutellum rugose medially, without transverse carinae (Figs 193, 396)	48
–	Metascutellum smooth medially, or with transverse carinae (Figs 279, 356)	49
48	Mesoscutellum with many strong longitudinal rugae (Fig. 396). Occipital carina with a strong median peak or arch (Fig. 396)	*Oxyscelio sinuum* Burks, sp. n.
–	Mesoscutellum without longitudinal rugae (Fig. 193). Occipital carina rounded medially (Fig. 193)	*Oxyscelio flabelli* Burks, sp. n.
49	Fore wing not long enough to reach beyond T5 in females (Fig. 358). Occipital carina with a sharp peak medially (Fig. 356)	*Oxyscelio ogive* Burks, sp. n.
–	Fore wing long enough to reach middle of T6 in females. Occipital carina weakly sinuate, with a weaker median peak (Fig. 281)	*Oxyscelio labis* Burks, sp. n.
50	Males (metasoma with 8 externally visible terga, the 8th small and directed posteriorly)	51
–	Females (metasoma with 6 externally visible terga on dorsal surface of metasoma, last pair of terga held internally unless ovipositor system extruded)	59
51	T1 midlobe with 6 or more anteriorly complete longitudinal carinae (Fig. 69)	52
–	T1 midlobe with 5 or fewer anteriorly complete longitudinal carinae (Fig. 224)	54
52	Head with depression extending from median ocellus to submedian carina (Fig. 251). Halmahera	*Oxyscelio halmaherae* Burks, sp. n.
–	Head without such a depression (Fig. 67)	53
53	Mesoscutellum with granulate sculpture (Fig. 179). Body brownish. the Philippines	*Oxyscelio dorsalis* (Kieffer)
–	Mesoscutellum without granulate sculpture (Fig. 65). Body metallic blue. Christmas Island	*Oxyscelio caesitas* Burks, sp. n.
54	T7 with bluntly rounded posterior corners that are not tapering (Fig. 132)	*Oxyscelio crateris* Burks, sp. n.
–	T7 with tapering posterior corners (Fig. 224)	55
55	Interantennal process elongate (Fig. 190)	*Oxyscelio fistulae* Burks, sp. n.
–	Interantennal process not elongate (Fig. 155)	6
56	A11 longer than broad (Fig. 224)	*Oxyscelio fossularum* Burks, sp. n.
–	A11 broader than long (Figs 292, 331)	57
57	Propodeum forming an interrupted arch over the base of T1, with metascutellum resting in the interruption (Fig. 159)	*Oxyscelio cyrtomesos* Burks, sp. n.
–	Propodeum not forming an arch over base of T1 (Fig. 332), metascutellum not so closely associated with propodeum	58
58	Metascutellum with a smooth median area or with branched rugae (Fig. 332)	*Oxyscelio naraws* Kozlov & Lê
–	Metascutellum with straight ruga (Fig. 293)	*Oxyscelio latitudinis* Burks, sp. n.
59	T2 with strong, curved and parallel sublateral striae, the medial ones without setae between them (Figs 66, 413); triangular anteromedian area present between the two sets of striae, setose and slightly raised (Fig. 413). *Oxyscelio striarum* Species Group	60
–	T2 with weaker longitudinal rugae or striae, with setae between all of them, and with no raised triangular area medially (Fig. 330)	61
60	Body metallic blue. Mesoscutellar apex concave (Fig. 65)	*Oxyscelio caesitas* Burks, sp. n.
–	Body brownish. Mesoscutellar apex straight or weakly convex (Fig. 411)	*Oxyscelio striarum* Burks, sp. n.
61	T2 and anterior part of T3 with sublateral longitudinal depressions, these medially bordered by a strong carina or unusually strong ruga (Fig. 217). *Oxyscelio fossarum* Species Group	62
–	T2 and T3 without sublateral longitudinal depressions (Fig. 330)	66
62	Mesoscutellum with granulate sculpture (Fig. 156)	63
–	Mesoscutellum without granulate sculpture (Fig. 211)	65
63	Metascutellum tongue-shaped; propodeum forming a long arch over base of T1, with a narrow median break (Fig. 156)	*Oxyscelio cyrtomesos* Burks, sp. n.
–	Metascutellum broader and subrectangular or apically emarginate; propodeum forming only a very short arch anteriorly, this not overhanging base of T1 and without median break (Figs 215, 221)	64
64	Fore wings long enough to reach middle of T4 (Fig. 217). T1 midlobe without longitudinal carinae anteriorly (Fig. 217)	*Oxyscelio fossarum* Burks, sp. n.
–	Fore wings long enough to reach middle of T5 or beyond. T1 midlobe with longitudinal carinae anteriorly (Fig. 222)	*Oxyscelio fossularum* Burks, sp. n.
*Note*:	Unknown females of *Oxyscelio fistulae* would key to this couplet, but should be distinguished in having an elongate interantennal process (as in Fig. 190).	
65	T5, T6 elongate and nearly parallel-sided (Fig. 213). Fore wings long enough to reach middle of T4	*Oxyscelio fodiens* Burks, sp. n.
–	T5, T6 moderately broad and tapering, not nearly parallel-sided (Fig. 9). Fore wings long enough to reach middle of T5	*Oxyscelio acutiventris* (Kieffer)
66	Metascutellum broad, medially rugose (Fig. 285). *Oxyscelio latitudinis* Species Group	67
–	Metascutellum narrow, medially smooth (Fig. 131)	*Oxyscelio crateris* Burks, sp. n.
67	T5, T6 elongate and nearly parallel-sided (Fig. 290)	*Oxyscelio latitudinis* Burks, sp. n.
–	T5, T6 moderately broad and tapering, broader and not nearly parallel-sided (Figs 330, 372)	68
68	Metascutellum very broad, with a median incision (Fig. 370)	*Oxyscelio perpensus* Kononova
–	Metascutellum about as long as broad, convex or truncate apically (Figs 328, 330)	*Oxyscelio naraws* Kozlov & Lê
69	Frontal depression crossed by many (>5) transverse carinae or grooves along its entire length, or with many dorsal transverse carinae that are discontinuous medially but reaching the midpoint from both sides (Figs 102, 343). (Fig. 342). *Oxyscelio noduli* Species Group	70
–	Frontal depression almost entirely smooth, with fewer than 5 carinae, these either complete or broadly interrupted (Fig. 134)	72
70	Fore wings long enough to reach middle of T6. Metascutellum completely smooth aside from peripheral carina (Fig. 342). T1 midlobe with a very slight, indistinct anterior horn (Fig. 344)	*Oxyscelio noduli* Burks, sp. n.
–	Fore wings long enough to reach middle of T5. Metascutellum with some central sculpture (Figs 101, 338). T1 midlobe with a strong anterior horn (Fig. 340)	71
71	Body yellowish. Metascutellar apex weakly emarginate (Fig. 101)	*Oxyscelio chimaerae* Burks, sp. n.
–	Body dark greenish-brown. Metascutellar apex convex (Fig. 338)	*Oxyscelio nodorum* Burks, sp. n.
72	Mesosoma anteriorly very tall and steep, descending at a right angle (Figs 16, 294). India, Sri Lanka. *Oxyscelio limae* Species Group	73
–	Mesosoma more weakly curved, not descending at a right angle. Widespread	75
73	Mesoscutum without extra carinae between median carina and notauli (Fig. 18)	*Oxyscelio anguli* Burks, sp. n.
–	Mesoscutum with extra set of carinae between median carina and notauli, anteriorly with 5 longitudinal carinae (Figs 201, 298)	74
74	Metasoma yellow; metascutellum tiny, not extending over base of T1; T1 midlobe with 5 separate longitudinal carinae in females and no smooth elevation(Fig. 201)	*Oxyscelio flaviventris* Burks, sp. n.
–	Metasoma black or dark brown; metascutellum large, extending partially over base of T1 (Fig. 298). T1 midlobe with smooth elevation anteriorly in females, this interrupting the carinae (Fig. 296)	*Oxyscelio limae* Burks, sp. n.
75	Metascutellum nearly flat and with some rugae or weak irregular sculpture centrally, without transverse carinae (Fig. 173). *Oxyscelio florus* Species Group	76
–	Metascutellum concave, smooth centrally or with one or more sharp transverse carinae (Fig. 137). *Oxyscelio crebritas* Species Group	80
76	Mesoscutellum with granulate sculpture (Fig. 39)	*Oxyscelio arvi* Burks, sp. n.
–	Mesoscutellum without granulate sculpture (Fig. 260)	77
77	Upper frons with one or more extra carinae parallel to submedian carina (Figs 174, 206)	78
–	Upper frons without additional carinae parallel to submedian carina (Figs 261, 389)	79
78	Fore wings long enough to reach middle of T4 or T5. Mesoscutellum with median carina (Fig. 205)	*Oxyscelio florus* Kononova
–	Fore wings long enough to reach apex of T5 or middle of T6. Mesoscutellum without median carina (Fig. 173)	*Oxyscelio dermatoglyphes* Burks, sp. n.
79	Mesosoma in known specimen yellow (Fig. 259); mesofemoral depression crossed by 3 carinae	*Oxyscelio jaune* Burks, sp. n.
–	Mesosoma in known specimens black (Fig. 387); mesofemoral depression crossed by more than 5 carinae	*Oxyscelio regionis* Burks, sp. n.
80	Mesoscutum and mesoscutellum entirely granulate (Fig. 242)	81
–	At least mesoscutum with areas that are not granulate (Figs 137, 264)	82
81	Gena granulate (Fig. 241)	*Oxyscelio granuli* Burks, sp. n.
–	Gena without granulate sculpture (Fig. 63)	*Oxyscelio brevinervis* (Kieffer)
82	Mesoscutellum with extensive granulate sculpture laterally (Figs 230, 264)	83
–	Mesoscutellum without granulate sculpture (Fig. 137)	85
83	Weak median carina present along occiput (Fig. 232). Gena with very strong middle carina (Fig. 229)	*Oxyscelio genae* Burks, sp. n.
–	Occiput without median carina (Fig, 264). Middle genal carina not very strong (Fig. 263)	84
84	Male (females unknown): T1 midlobe with 3 longitudinal carinae. Philippines	*Oxyscelio kiefferi* Dodd
–	T1 midlobe with 4 longitudinal carinae (Fig. 268).	*Oxyscelio jugi* Burks, sp. n.
85	Middle genal carina angled towards posterior genal carina (Fig. 381)	86
–	Middle genal carina parallel with eye margin (Fig. 133)	87
86	Female: T1 midlobe without anterior elevation, longitudinal carinae distinct (Fig. 383). Male: T7 with rounded posterior corners (Fig. 386)	*Oxyscelio reflectens* Burks, sp. n.
–	Female: T1 midlobe with smooth elevation rendering anterior carinae uncountable (Fig. 79). Male: T7 with sharp posterior corners (Fig. 81)	*Oxyscelio capitis* Burks, sp. n.
87	Metapleuron above ventral metapleural area rugose or with very short carinae that do not cross it, not crossed by long straight carinae (Figs 42, 282)	88
–	Metapleuron above ventral metapleural area crossed by long straight carinae, or smooth centrally (Figs 10, 133)	90
88	Submedian carina not defined (as in Fig. 251)	*Oxyscelio lacunae* Burks, sp. n.
–	Submedian carina sharp, well-defined (Fig. 134)	89
89	Male (females unknown): T1 midlobe with 4 anteriorly complete longitudinal carinae (Fig. 187). A5 tyloid carina-like, not expanded. Philippines	*Oxyscelio excavatus* (Kieffer)
–	Male (see Figs 42–45 for females): T1 midlobe with 3 anteriorly complete longitudinal carinae (as in Fig. 138). A5 tyloid expanded, sinuate or comma-shaped (as in Fig. 385)	*Oxyscelio asperi* Burks, sp. n.
90	Setae along anterior limit of femoral depression dense and very numerous, arising only from tiny pits that are not bordered by a carina (Fig. 70)	*Oxyscelio capilli* Burks, sp. n.
–	Setae along anterior limit of femoral depression more sparsely distributed, arising from foveae that are bordered by a carina dorsally (Fig. 133)	91
91	Medial mesoscutum and mesoscutellum with transverse rugae (Fig. 11). Female: T6 apex sharply acuminate (Fig. 13)	*Oxyscelio amrichae* Burks, sp. n.
–	Medial mesoscutum and mesoscutellum without transverse rugae (Fig. 137). Female: T6 apex rounded. (Fig. 135)	92
92	Male, T1 midlobe with 5 anteriorly complete longitudinal carinae (Fig. 305)	93
–	Female, or T1 midlobe with 4 or fewer anteriorly complete longitudinal carinae (Fig. 138)	94
93	Occipital carina absent medially; medial mesoscutum strongly sculptured, with strong longitudinal elevation between median carina and notaulus (Fig. 301)	*Oxyscelio longiventris* Burks, sp. n.
–	Occipital carina complete medially; medial mesoscutum weakly sculptured, without elevation between median carina and notaulus (Fig. 323)	*Oxyscelio mollitia* Burks, sp. n.
94	Female: Fore wings not long enough to reach past T4 (Fig. 302)	*Oxyscelio longiventris* Burks, sp. n.
–	Specimen male, or female with fore wings long enough to reach middle of T5 (Fig. 107)	95
95	Female: T1 midlobe with smooth elevation anteriorly, obscuring anterior carinae; T6 longer than broad (Figs 107, 325)	96
–	Female: T1 midlobe with 5 separate carinae anteriorly, or T6 broader than long (Fig. 135). (Males cannot be reliably keyed past this couplet)	97
96	Mesofemoral depression crossed by more than 5 carinae (Fig. 322). Female: A5 longer than broad	*Oxyscelio mollitia* Burks, sp. n.
–	Mesofemoral depression crossed by at most 5 carinae (Fig. 104). Female: A5 broader than long	*Oxyscelio codae* Burks, sp. n.
97	Mesoscutum and mesoscutellum shiny, with weak sculpture that appears melted; sculpture of posterior portion of medial mesoscutum umbilicate-punctate (Figs 111, 115). Philippines	*Oxyscelio consobrinus* (Kieffer)
–	Mesoscutum and mesoscutellum not shiny, with strong sculpture; sculpture of posterior portion of medial mesoscutum umbilicate-foveate, with no punctate areas (Fig. 137). Widespread, but absent from Philippines	*Oxyscelio crebritas* Burks, sp. n.

## Species descriptions

### Oxyscelio aclavae

Burks sp. n.

urn:lsid:zoobank.org:act:51993891-E31E-4C1C-B7CC-08E4C3FCFC2A

urn:lsid:biosci.ohio-state.edu:osuc_concepts:275554

http://species-id.net/wiki/Oxyscelio_aclavae

**Figures 2–7.**
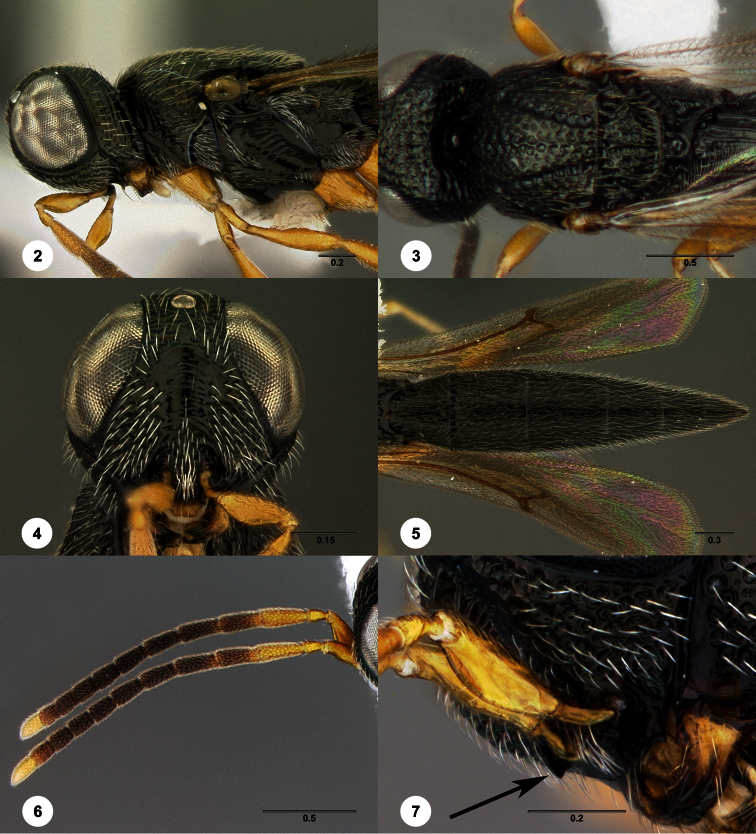
*Oxyscelio aclavae* sp. n., paratype female (OSUC 352476) **2** Head and mesosoma, lateral view. Paratype female (OSUC 335830) **3** Head and mesosoma, dorsal view. Paratype female (OSUC 247834) **4 **Head, anterior view. Paratype female (OSUC 369083) **5** Metasoma, dorsal view. Paratype female (OSUC 257059) **6** Antennae **7** Ventral frons, oblique view; arrow indicates oblique flange. Morphbank^24^

Figures 2–7 Morphbank^24^

#### Description.

*Female*. Body length 3.75–5.3 mm (n=20).

Radicle color: same color as scape. Scape color: Yellowish. A4: longer than broad. A5: longer than broad. Antennal club: not formed, segments not compact.

Interantennal process: not elongate. Median longitudinal elevation in frontal depression: absent. Frontal depression: concave. Frontal depression sculpture: crossed by many tiny furrows. Submedian carina: strong, formed by a sharp raised carina. Submedian carina medially: without peak. Concavity across dorsal part of frontal depression: absent. Depression extending ventrally from median ocellus: absent. Upper frons: not hood-like. Malar area near antennal foramen: with oblique tooth-like flange (facial nubbin). Malar area at mouth corner: with radiating striae. Smooth strip along posterior side of malar sulcus: present, broad throughout its length. Middle genal carina: present. Direction of middle genal carina dorsally: parallel to eye margin. Major sculpture of gena anteriorly: umbilicate-foveate; rugose. Major sculpture of gena posteriorly: rugose. Microsculpture of gena antero-ventrally: granulate. Microsculpture of gena postero-ventrally: granulate. Median carina extending posteriorly from hyperoccipital carina: absent. Hyperoccipital carina: not indicated medially. Lateral connection between hyperoccipital and occipital carinae: absent. Area between vertex and occipital carina: umbilicate-foveate. Occipital carina medially: sinuate, concave medial to corners, but without a median peak. Lateral corners of occipital carina: sharp and protruding.

Lateral pronotal area: without bulge projecting towards anterior pit. Epomial corner: strong. Netrion surface anteriorly: not inflexed. Mesoscutum anteriorly: not steep. Mesoscutal median carina: present and complete. Longitudinal carina between median carina and notauli: absent. Major sculpture of medial mesoscutum anteriorly: umbilicate-foveate. Major sculpture of medial mesoscutum posteriorly: umbilicate-foveate. Microsculpture of medial mesoscutum anteriorly: granulate. Microsculpture of medial mesoscutum posteriorly: absent. Major sculpture of mesoscutellum: umbilicate-foveate; irregularly rugose. Microsculpture of mesoscutellum medially: punctate. Microsculpture of mesoscutellum laterally: punctate. Mesoscutellar apex: convex or straight. Setae along anterior limit of femoral depression: arising from rows of foveae. Number of carinae crossing speculum above femoral depression: 2. Number of carinae crossing femoral depression: more than 5. Mesepimeral sulcus pits: more than 5. Metascutellum dorsally: concave. Metascutellar sculpture dorsally: smooth or with transverse carinae. Median carina of metascutellum: absent or branched. Metascutellar setae: absent. Metascutellar apex: convex or straight. Metapleuron above ventral metapleural area: smooth. Metasomal depression setae: absent. Lateral propodeal carinae antero-medially: strongly diverging. Anterior areoles of metasomal depression: absent. Anterior longitudinal carinae in metasomal depression: absent. Lateral propodeal areas: meeting for only a short distance medially. Postmarginal vein: present. Forewing apex: reaching middle of T5; reaching apex of T5.

T1 midlobe: obscured by other raised sculpture. T1: with small rounded anterior bulge, not reaching metascutellum. T2: with long sublateral depressions. T6: broader than long. Apical flange of T6: not exposed apically. Metasomal apex: rounded. Major sculpture of T6: umbilicate-punctate; longitudinally striate or rugose. Microsculpture of T6: granulate.

*Male*. Unknown.

#### Diagnosis.

Female: Antennal club not formed, flagellomeres widely separated. Face with oblique expanded flange between antennal foramen and eye. Metascutellum longer than broad, with central smooth channel.

#### Etymology.

Latin noun, genitive case, intended to mean “clubless.” Refers to the long and well-separated apical flagellomeres.

#### Link to distribution map.

[http://hol.osu.edu/map-full.html?id=275554]

#### Material examined.

Holotype, female: **THAILAND**: Chanthaburi Prov., inside youth camp, T3345, Khao Khitchakut National Park, 12°50.570'N, 102°07.220’E, 12m, 8.IX–15.IX.2008, malaise trap, Suthida & Charoenchai, OSUC 368762 (deposited in QSBG). *Paratypes*: (78 females) **BRUNEI**: 2 females, OSUC 376633, 376655 (BMNH). **INDONESIA**: 28 females, OSUC 257096, 376652-376654, 376658, 376661 (BMNH); OSUC 368943, 368955, 368957, 368963, 369074, 369083 (CNCI); OSUC 240914, 247845, 247854, 247865, 257074 (MBBJ); OSUC 228684-228686, 228697, 228700, 241815, 247834, 247839 (OSUC); OSUC 257059, 257061, 257070 (ROME). **MALAYSIA**: 20 females, OSUC 202717 (AEIC); OSUC 376580, 376587, 376589, 376592, 376594, 376599, 376603, 376606-376607, 376610, 376613 (BMNH); OSUC 369323, 369334 (CNCI); OSUC 376748-376749 (MCZC); OSUC 381324, 453782, 453787, 453794 (OSUC). **SRI LANKA**: 1 female, OSUC 268123 (USNM). **THAILAND**: 27 females, OSUC 335869 (BMNH); OSUC 368757-368758, 368768 (CNCI); OSUC 320372, 320407, 322089, 335911, 352472-352475 (OSUC); OSUC 335116, 335118-335119, 336027, 336045, 336119, 352476, 361337, 361340, 361349, 361364, 361366, 361374 (QSBG); OSUC 335144, 335830 (WINC).

#### Comments.

Females of *Oxyscelio aclavae* are frequently collected, but males are unknown. This species can be easily recognized by the lack of an antennal club in females, in which the apical flagellomere is at least partially white, and by the oblique flange near the antennal foramen. A12 in most specimens is entirely white, but it is only partially white in some smaller specimens. A long metascutellum and similar oblique facial flange also occurs in *Oxyscelio latinubbin*,which may be closely related to *Oxyscelio aclavae* if the T2 longitudinal depressions prove homoplastic.

### Oxyscelio acutiventris

(Kieffer)

urn:lsid:zoobank.org:act:F2981FE6-BACF-4F5B-960E-E3267A94F49D

urn:lsid:biosci.ohio-state.edu:osuc_concepts:5005

http://species-id.net/wiki/Oxyscelio_acutiventris

**Figures 8–9.**
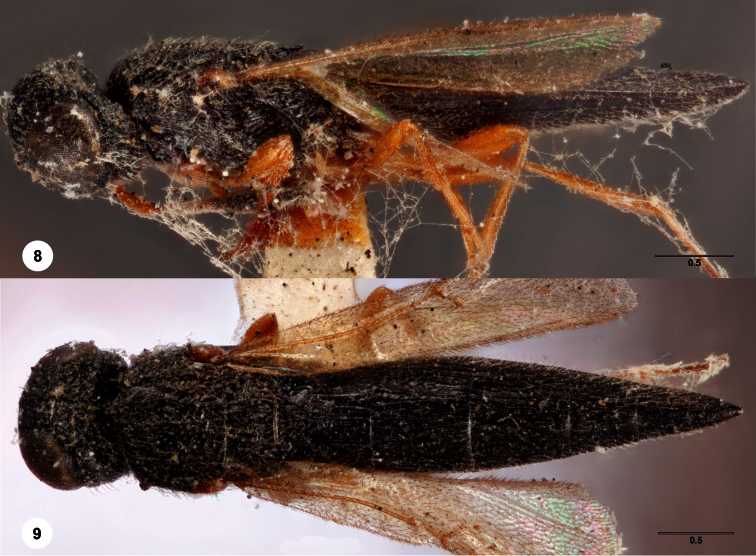
*Oxyscelio acutiventris* (Kieffer), holotype female (Museum Paris EY0000003996) **8** Body, lateral view **9** Body, dorsal view. Morphbank^25^

Figures 8–9 Morphbank^25^

Trichanteris acutiventris Kieffer, 1916: 176 (original description); Kelner-Pillault 1958: 152 (type information).
Dicroteleia acutiventris (Kieffer): Kieffer 1926: 387, 388 (generic transfer, description, keyed).
Oxyscelio acutiventris (Kieffer): Dodd 1931: 74 (generic transfer).

#### Description.

*Female*. Body length 4.25 mm (n=1).

Radicle color: same color as scape. Scape color: Yellowish. A4: longer than broad. A5: longer than broad. Antennal club: formed, segments compact.

Interantennal process: not elongate. Median longitudinal elevation in frontal depression: absent. Frontal depression: concave. Frontal depression sculpture: with 3-5 complete transverse carinae. Submedian carina: weak, shallow and rounded or formed by ledge. Submedian carina medially: without peak. Concavity across dorsal part of frontal depression: absent. Depression extending ventrally from median ocellus: absent. Upper frons: not hood-like. Malar area near antennal foramen: without carina or expansion. Malar area at mouth corner: with radiating striae. Smooth strip along posterior side of malar sulcus: present, broad throughout its length. Middle genal carina: present. Direction of middle genal carina dorsally: parallel to eye margin. Major sculpture of gena anteriorly: umbilicate-foveate. Major sculpture of gena posteriorly: umbilicate-foveate; rugose. Microsculpture of gena anteroventrally: granulate. Microsculpture of gena posteroventrally: granulate. Median carina extending posteriorly from hyperoccipital carina: absent. Hyperoccipital carina: indicated by rugae. Lateral connection between hyperoccipital and occipital carinae: absent. Area between vertex and occipital carina: umbilicate-foveate. Occipital carina medially: absent. Lateral corners of occipital carina: sharp and protruding.

Lateral pronotal area: without bulge projecting towards anterior pit. Epomial corner: weak. Netrion surface anteriorly: not inflexed. Mesoscutum anteriorly: not steep. Mesoscutal median carina: present and complete. Longitudinal carina between median carina and notauli: absent. Major sculpture of medial mesoscutum anteriorly: umbilicate-foveate. Major sculpture of medial mesoscutum posteriorly: umbilicate-foveate. Microsculpture of medial mesoscutum anteriorly: absent. Microsculpture of medial mesoscutum posteriorly: absent. Major sculpture of mesoscutellum: umbilicate-foveate. Microsculpture of mesoscutellum medially: absent. Microsculpture of mesoscutellum laterally: absent. Mesoscutellar apex: roundly concave. Setae along anterior limit of femoral depression: arising from rows of foveae. Number of carinae crossing speculum above femoral depression: 3. Number of carinae crossing femoral depression: more than 5. Mesepimeral sulcus pits: more than 5. Metascutellum dorsally: flat. Metascutellar sculpture dorsally: with scattered rugae. Median carina of metascutellum: absent or branched. Metascutellar setae: absent. Metascutellar apex: weakly emarginate. Metapleuron above ventral metapleural area: foveate or rugose. Metasomal depression setae: absent. Lateral propodeal carinae anteromedially: strongly diverging. Anterior areoles of metasomal depression: absent. Anterior longitudinal carinae in metasomal depression: absent. Lateral propodeal areas: meeting for only a short distance medially. Postmarginal vein: present. Fore wing apex: reaching middle of T5.

T1 midlobe: obscured by other raised sculpture. T1: with long anterior bulge, reaching metascutellum. T2: with long sublateral depressions. T6: longer than broad. Apical flange of T6: exposed apically. Metasomal apex: rounded. Major sculpture of T6: umbilicate-punctate. Microsculpture of T6: granulate.

*Male*. Unknown.

#### Diagnosis.

Female: Frontal depression crossed by a few carinae. Mesoscutellum without granulate sculpture. Metascutellum subrectangular, rugose. Fore wings long enough to reach middle of T5. T6 apically narrow but not sharply acuminate. T1 with a well-developed anterior horn with anteriorly obscure longitudinal carinae. T2 with long sublateral depressions bordered medially by strong carinae.

#### Link to distribution map.

[http://hol.osu.edu/map-full.html?id=5005]

#### Material examined.

Holotype, female, *Trichanteris acutiventris*: **PHILIPPINES**: Laguna Prov., Luzon Isl., Mount Makiling, no date, Baker, Museum Paris EY0000003996 (deposited in MNHN).

### Oxyscelio amrichae

Burks sp. n.

urn:lsid:zoobank.org:act:B4E57288-088C-49D1-866D-A2CAFC24448F

urn:lsid:biosci.ohio-state.edu:osuc_concepts:275570

http://species-id.net/wiki/Oxyscelio_amrichae

**Figures 10–15.**
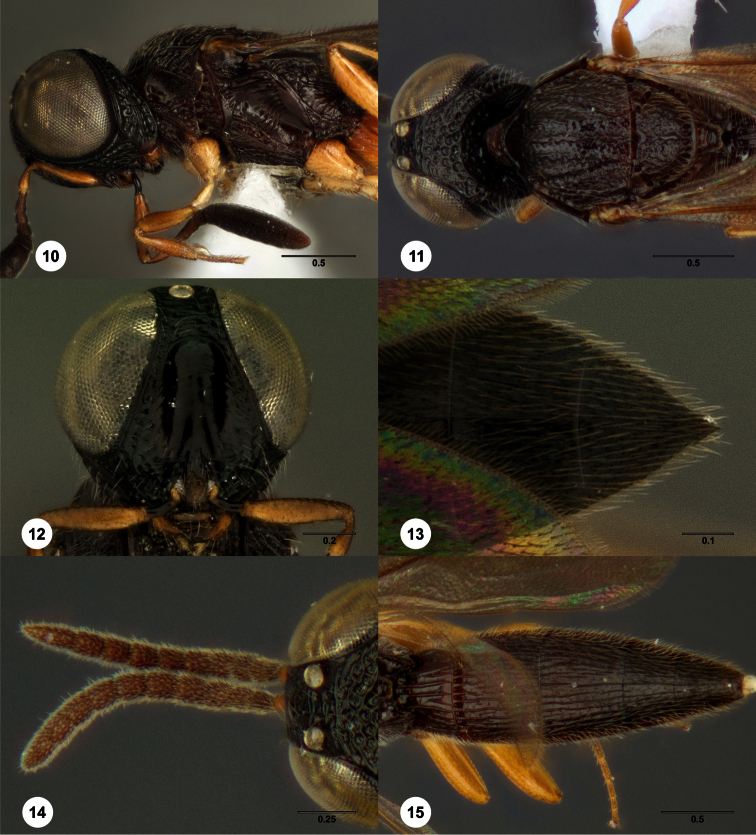
*Oxyscelio amrichae* sp. n., paratype female (OSUC 247855) **10** Head and mesosoma, lateral view **11** Head and mesosoma, dorsal view. Paratype female (OSUC 240927) **12** Head, anterior view. Paratype female (ROMEnt Spec. No. 112240) **13** Metasomal apex, dorsal view. Paratype male (OSUC 228746) **14** Antenna **15** Metasoma, dorsal view. Morphbank^26^

Figures 10–15 Morphbank^26^

#### Description.

*Female*. Body length 3.9–4.45 mm (n=17).

Radicle color: darker than scape. Scape color: Brown. A4: broader than long; as long as broad. A5: broader than long. Antennal club: formed, segments compact.

Interantennal process: not elongate. Median longitudinal elevation in frontal depression: absent. Frontal depression: concave. Frontal depression sculpture: with 3 or more broadly interrupted transverse carinae. Submedian carina: strong, formed by a sharp raised carina. Submedian carina medially: without peak. Concavity across dorsal part of frontal depression: absent. Depression extending ventrally from median ocellus: absent. Upper frons: not hood-like. Malar area near antennal foramen: without carina or expansion. Malar area at mouth corner: with radiating striae. Smooth strip along posterior side of malar sulcus: absent or not consistently broad. Middle genal carina: present. Direction of middle genal carina dorsally: parallel to eye margin. Major sculpture of gena anteriorly: umbilicate-foveate; rugose. Major sculpture of gena posteriorly: umbilicate-foveate; rugose. Microsculpture of gena anteroventrally: absent. Microsculpture of gena posteroventrally: absent. Median carina extending posteriorly from hyperoccipital carina: absent. Hyperoccipital carina: indicated by rugae. Lateral connection between hyperoccipital and occipital carinae: absent. Area between vertex and occipital carina: umbilicate-foveate. Occipital carina medially: absent. Lateral corners of occipital carina: not protruding.

Lateral pronotal area: without bulge projecting towards anterior pit. Epomial corner: weak. Netrion surface anteriorly: not inflexed. Mesoscutum anteriorly: not steep. Mesoscutal median carina: present and complete. Longitudinal carina between median carina and notauli: present. Major sculpture of medial mesoscutum anteriorly: umbilicate-foveate; transversely rugose. Major sculpture of medial mesoscutum posteriorly: umbilicate-punctate. Microsculpture of medial mesoscutum anteriorly: granulate. Microsculpture of medial mesoscutum posteriorly: absent. Major sculpture of mesoscutellum: umbilicate-foveate; irregularly rugose. Microsculpture of mesoscutellum medially: absent. Microsculpture of mesoscutellum laterally: absent. Mesoscutellar apex: convex or straight. Setae along anterior limit of femoral depression: arising from rows of foveae. Number of carinae crossing speculum above femoral depression: 2. Number of carinae crossing femoral depression: 3-5; more than 5. Mesepimeral sulcus pits: 3-5. Metascutellum dorsally: concave. Metascutellar sculpture dorsally: smooth or with transverse carinae. Median carina of metascutellum: straight, unbranched carina present. Metascutellar setae: absent. Metascutellar apex: weakly emarginate. Metapleuron above ventral metapleural area: crossed by carinae. Metasomal depression setae: absent. Lateral propodeal carinae anteromedially: weakly diverging. Anterior areoles of metasomal depression: absent. Anterior longitudinal carinae in metasomal depression: absent. Lateral propodeal areas: separated medially. Postmarginal vein: present. Fore wing apex: reaching apex of T5; reaching middle of T6.

T1 midlobe: with 5 longitudinal carinae. T1: without anterior bulge. T2: with straight longitudinal striae or rugae. T6: longer than broad; as long as broad. Apical flange of T6: not exposed apically. Metasomal apex: tapering to a sharp point. Major sculpture of T6: umbilicate-punctate; longitudinally striate or rugose. Microsculpture of T6: granulate.

*Male*. Body length 3.4–4.5 mm (n=20). A5 tyloid: carina-like, not expanded. A11: longer than broad. Median tooth of frontal depression: absent. Median lobe of T1: with 4 longitudinal carinae; with 5 longitudinal carinae. Metasomal apex: with acuminate lateral corners.

#### Diagnosis.

Both sexes: Middle genal carina subparallel with eye margin. Hyperoccipital carina indicated by rugae. Mesoscutellum without granulate sculpture. Metascutellum concave dorsally, smooth aside from some transverse carinae. Female: A4, A5 not longer than broad. T1 midlobe with 5 longitudinal carinae. T6 acuminate apically. Male: A11 slightly longer than broad. T1 midlobe with 4 or 5 longitudinal carinae. T7 with short, sharp and protruding posterolateral corners. *Oxyscelio amrichae* is very similar to *Oxyscelio jugi* in that both have an acuminate T6, but *Oxyscelio amrichae* differs in lacking granulate sculpture on the mesoscutellum. It can also be recognized by its usually reddish color.

#### Etymology.

Named in honor of Ruth Amrich, a dedicated and skilled entomologist.

#### Link to distribution map.

[http://hol.osu.edu/map-full.html?id=275570]

#### Material examined.

Holotype, female: **INDONESIA**: Aceh Auto. Prov., Sumatra Isl., Ketambe Research Station, 1° rainforest / young forest / terrace 3 closed canopy, IIS 900011, Gunung Leuser National Park, 03°41'N, 97°39’E, 350m, II-1990, malaise trap, C. Darling, OSUC 247976 (deposited in MBBJ). *Paratypes*: (16 females, 40 males) **BRUNEI**: 1 female, 8 males, OSUC 376630-376631, 376634-376636, 376638-376639, 376642, 376656 (BMNH).**INDONESIA**: 9 females, 32 males, OSUC 361276, ROMEnt Spec. No. 112234 (BMNH); OSUC 247842, 247973, 257050, 257077, ROMEnt Spec. No. 112236, ROMEnt Spec. No. 112237, ROMEnt Spec. No. 112238, ROMEnt Spec. No. 112241, ROMEnt Spec. No. 112242 (MBBJ); OSUC 228742, 228746, 240916, 240918, 240927, 247849, 247864, 248899, 251439, 257425, 361274 (OSUC); OSUC 228711, 228713, 240915, 240928, 240932, 247855, 247859-247860, 247966, 247968-247969, 247972, 257054-257055, 257422, 257424, 257429, ROMEnt Spec. No. 112240, ROMEnt Spec. No. 112249 (ROME). **MALAYSIA**: 7 females, 1 male, OSUC 376581, 376591, 376593, 376596-376598, 376604 (BMNH); OSUC 369063 (CNCI).

### Oxyscelio anguli

Burks sp. n.

urn:lsid:zoobank.org:act:DF07EB3C-5CDE-4434-BE42-F425444E4650

urn:lsid:biosci.ohio-state.edu:osuc_concepts:275509

http://species-id.net/wiki/Oxyscelio_anguli

**Figures 16–21.**
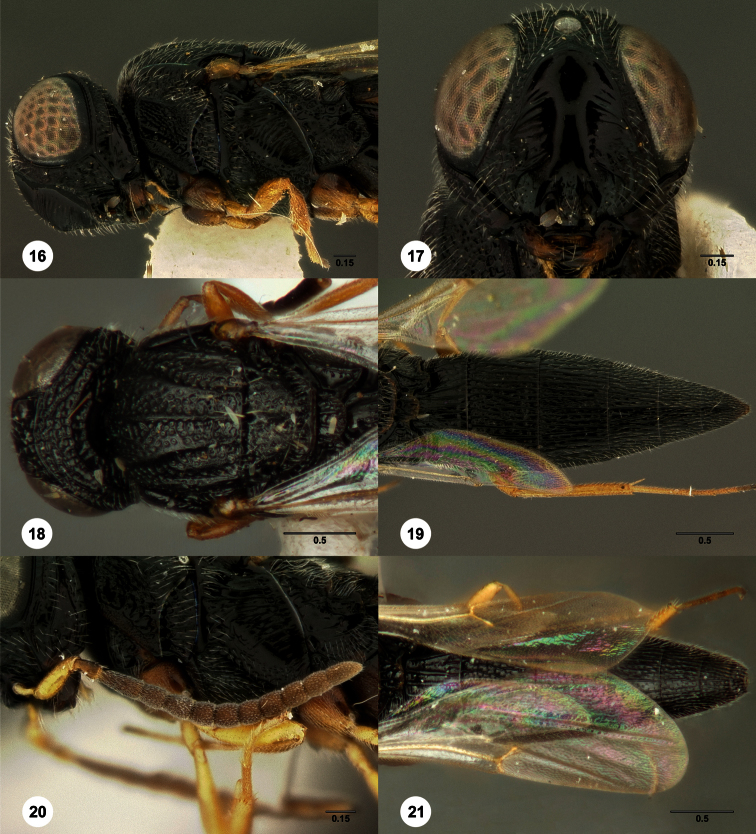
*Oxyscelio anguli* sp. n., paratype female (OSUC 268128) **16** Head and mesosoma, lateral view **17** Head, anterior view.Paratype female (OSUC 268142) **18** Head and mesosoma, dorsal view. Paratype female (OSUC 268130) **19** Metasoma, dorsal view. Paratype male (OSUC 268279) **20** Antenna **21** Metasoma, dorsal view. Morphbank^27^

Figures 16–21 Morphbank^27^

#### Description.

*Female*. Body length 4.75–5.75 mm (n=12).

Radicle color: same color as scape. Scape color: Yellowish. A4: broader than long; as long as broad. A5: broader than long. Antennal club: formed, segments compact.

Interantennal process: not elongate. Median longitudinal elevation in frontal depression: absent. Frontal depression: concave. Frontal depression sculpture: without transverse or oblique carinae below submedian carina. Submedian carina: strong, formed by a sharp raised carina. Submedian carina medially: without peak. Concavity across dorsal part of frontal depression: absent. Depression extending ventrally from median ocellus: absent. Upper frons: not hood-like. Malar area near antennal foramen: without carina or expansion. Malar area at mouth corner: with radiating striae. Smooth strip along posterior side of malar sulcus: absent or not consistently broad. Middle genal carina: present. Direction of middle genal carina dorsally: parallel to eye margin. Major sculpture of gena anteriorly: rugose; umbilicate-punctate. Major sculpture of gena posteriorly: umbilicate-foveate. Microsculpture of gena anteroventrally: absent. Microsculpture of gena posteroventrally: absent. Median carina extending posteriorly from hyperoccipital carina: absent. Hyperoccipital carina: indicated by rugae. Lateral connection between hyperoccipital and occipital carinae: absent. Area between vertex and occipital carina: umbilicate-foveate; with transverse carinae. Occipital carina medially: uniformly rounded. Lateral corners of occipital carina: not protruding.

Lateral pronotal area: without bulge projecting towards anterior pit. Epomial corner: strong. Netrion surface anteriorly: not inflexed. Mesoscutum anteriorly: steep. Mesoscutal median carina: present and complete. Longitudinal carina between median carina and notauli: present. Major sculpture of medial mesoscutum anteriorly: umbilicate-foveate; transversely rugose. Major sculpture of medial mesoscutum posteriorly: umbilicate-punctate; transversely rugose. Microsculpture of medial mesoscutum anteriorly: absent; granulate. Microsculpture of medial mesoscutum posteriorly: absent; granulate. Major sculpture of mesoscutellum: umbilicate-foveate; irregularly rugose. Microsculpture of mesoscutellum medially: absent. Microsculpture of mesoscutellum laterally: granulate. Mesoscutellar apex: convex or straight. Setae along anterior limit of femoral depression: arising from rows of foveae. Number of carinae crossing speculum above femoral depression: 3. Number of carinae crossing femoral depression: more than 5. Mesepimeral sulcus pits: more than 5. Metascutellum dorsally: flat. Metascutellar sculpture dorsally: with scattered rugae. Median carina of metascutellum: absent or branched. Metascutellar setae: absent. Metascutellar apex: convex or straight. Metapleuron above ventral metapleural area: crossed by carinae. Metasomal depression setae: absent. Lateral propodeal carinae anteromedially: strongly diverging. Anterior areoles of metasomal depression: absent. Anterior longitudinal carinae in metasomal depression: absent. Lateral propodeal areas: separated medially. Postmarginal vein: absent. Fore wing apex: reaching middle of T4.

T1 midlobe: obscured by other raised sculpture. T1: with small rounded anterior bulge, not reaching metascutellum. T2: with straight longitudinal striae or rugae. T6: longer than broad. Apical flange of T6: exposed apically. Metasomal apex: rounded. Major sculpture of T6: umbilicate-punctate; longitudinally striate or rugose. Microsculpture of T6: absent.

*Male*. Body length 4.65–4.7 mm (n=2).A5 tyloid: carina-like, not expanded. A11: longer than broad. Median tooth of frontal depression: absent. Median lobe of T1: with 5 longitudinal carinae. Metasomal apex: with acuminate lateral corners.

#### Diagnosis.

Both sexes: Hyperoccipital carina indicated by rugae; occipital carina complete. Mesosoma very tall and steep anteriorly, descending at a right angle. Medial mesoscutum without distinct carinae between notauli and median mesoscutal carina. Mesoscutellum with some granulate sculpture posterolaterally. Metascutellum extending over base of T1, with many longitudinal carinae or rugae. Female: Fore wing long enough to reach middle of T4. T1 midlobe with an elevation obscuring carinae anteriorly. T6 slightly longer than broad. Male: A11 longer than broad. T1 midlobe with 4 longitudinal carinae. T7 with sharp, protruding posterolateral corners. Males of *Oxyscelio anguli* are difficult to separate from those of *Oxyscelio limae*, but have a slightly longer flagellum, more granulate sculpture, and a slightly broader metascutellum.

#### Etymology.

Latin noun, genitive case, meaning “corner.” Refers to the slightly protruding corners of some metasomal terga.

#### Link to distribution map.

[http://hol.osu.edu/map-full.html?id=275509]

#### Material examined.

Holotype, female: **SRI LANKA**: Uva Prov., Moneragala Dist., 15km E Uda Walawe, 06°27'N, 80°28’E, 31.VII-3.VIII.1993, K. V. Krombein, J. W. Norden & B. B. Norden, OSUC 268177 (deposited in ISDF).*Paratypes*: **SRI LANKA**: 12 females, 2 males, OSUC 369090, 369160 (CNCI); OSUC 268098-268099, 268101, 268103, 268122, 268127-268128, 268130, 268142, 268147, 268164, 268279 (USNM).

### Oxyscelio angustifrons

Burks sp. n.

urn:lsid:zoobank.org:act:23809176-7451-4BA2-B580-161023F7B131

urn:lsid:biosci.ohio-state.edu:osuc_concepts:275487

http://species-id.net/wiki/Oxyscelio_angustifrons

**Figures 22–25.**
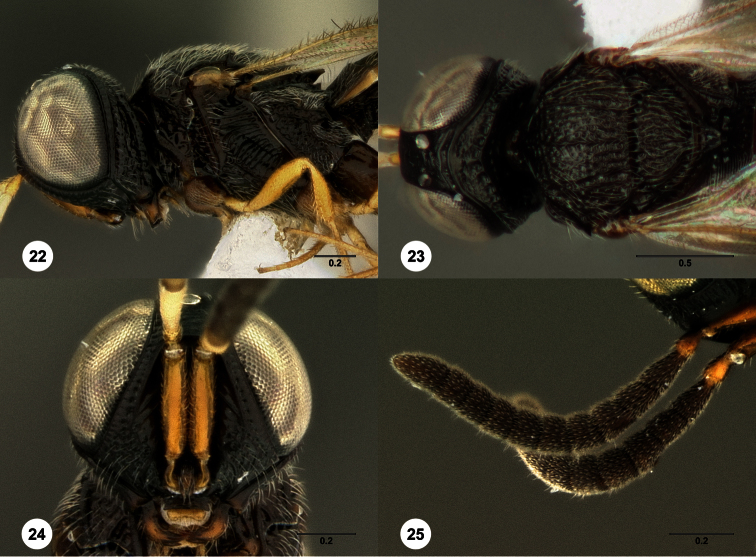
*Oxyscelio angustifrons* sp. n., holotype female (OSUC 247962) **22** Head and mesosoma, lateral view **23** Head and mesosoma, dorsal view **24** Head, anterior view. Paratype male (OSUC 247847) **25** Antenna. Morphbank^28^

Figures 22–25 Morphbank^28^

#### Description.

*Female*. Body length 2.95–3.1 mm (n=2).

Radicle color: same color as scape. Scape color: Yellowish. A4: broader than long. A5: broader than long. Antennal club: formed, segments compact.

Interantennal process: not elongate. Median longitudinal elevation in frontal depression: present. Frontal depression: concave. Frontal depression sculpture: with 3 or more broadly interrupted transverse carinae. Submedian carina: strong, formed by a sharp raised carina. Submedian carina medially: without peak. Concavity across dorsal part of frontal depression: absent. Depression extending ventrally from median ocellus: absent. Upper frons: hood-like, protruding over pedicel when antenna at rest. Malar area near antennal foramen: without carina or expansion. Malar area at mouth corner: with radiating striae. Smooth strip along posterior side of malar sulcus: absent or not consistently broad. Middle genal carina: absent. Direction of middle genal carina dorsally: parallel to eye margin. Major sculpture of gena anteriorly: umbilicate-foveate. Major sculpture of gena posteriorly: umbilicate-foveate. Microsculpture of gena anteroventrally: absent. Microsculpture of gena posteroventrally: absent. Median carina extending posteriorly from hyperoccipital carina: absent. Hyperoccipital carina: complete, continuous with anterior genal carina. Lateral connection between hyperoccipital and occipital carinae: absent. Area between vertex and occipital carina: irregularly rugose. Occipital carina medially: sinuate, concave medial to corners, but without a median peak. Lateral corners of occipital carina: not protruding.

Lateral pronotal area: without bulge projecting towards anterior pit. Epomial corner: strong. Netrion surface anteriorly: not inflexed. Mesoscutum anteriorly: not steep. Mesoscutal median carina: present and complete. Longitudinal carina between median carina and notauli: absent. Major sculpture of medial mesoscutum anteriorly: umbilicate-foveate. Major sculpture of medial mesoscutum posteriorly: umbilicate-foveate. Microsculpture of medial mesoscutum anteriorly: granulate. Microsculpture of medial mesoscutum posteriorly: absent. Major sculpture of mesoscutellum: umbilicate-foveate; longitudinally rugose. Microsculpture of mesoscutellum medially: absent. Microsculpture of mesoscutellum laterally: absent; granulate. Mesoscutellar apex: convex or straight. Setae along anterior limit of femoral depression: arising from rows of foveae. Number of carinae crossing speculum above femoral depression: 4. Number of carinae crossing femoral depression: more than 5. Mesepimeral sulcus pits: more than 5. Metascutellum dorsally: concave. Metascutellar sculpture dorsally: smooth or with transverse carinae. Median carina of metascutellum: absent or branched. Metascutellar setae: absent. Metascutellar apex: weakly emarginate. Metapleuron above ventral metapleural area: foveate or rugose. Metasomal depression setae: absent. Lateral propodeal carinae anteromedially: strongly diverging. Anterior areoles of metasomal depression: absent. Anterior longitudinal carinae in metasomal depression: absent. Lateral propodeal areas: separated medially. Postmarginal vein: present. Fore wing apex: reaching beyond T6.

T1 midlobe: with 6 or more longitudinal carinae. T1: without anterior bulge. T2: with straight longitudinal striae or rugae. T6: broader than long. Apical flange of T6: exposed apically. Metasomal apex: rounded. Major sculpture of T6: umbilicate-punctate. Microsculpture of T6: absent.

*Male*. Body length 2.95–3.35 mm (n=2). A5 tyloid: carina-like, not expanded. A11: broader than long. Median tooth of frontal depression: absent. Median lobe of T1: with 6 longitudinal carinae. Metasomal apex: with acuminate lateral corners.

#### Diagnosis.

Both sexes: Frons without elevation between antennal foramen and eye. Hyperoccipital carina present, continuous with anterior genal carina. Metascutellum narrowing posteriorly and only very slightly emarginate apically, without dorsal setae. Propodeum without median carina; lateral propodeal carinae broadly separated anteriorly. Female: T1 midlobe with 6 longitudinal carinae. T6 rounded apically. Male: A11 broader than long. T1 midlobe with 6 longitudinal carinae. T7 with acuminate posterolateral corners. The posteriorly narrowing metascutellum and large number of T1 midlobe carinae can be used to easily distinguish this rarely collected species from other members of the *cuculli*-group.

#### Etymology.

Latin noun in apposition to the generic name, meaning “narrow frons.” Refers to the very narrow upper frons.

#### Link to distribution map.

[http://hol.osu.edu/map-full.html?id=275487]

#### Material examined.

Holotype, female: **INDONESIA**: Kalimantan Barat Prov., Cabang Panti Research Station, 1° rainforest / alluvial light gap, IIS 910122, Gunung Palung National Park, 01°15'S, 110°05’E, 100–400m, 15.VI–15.VIII.1991, malaise trap, Darling & Rosichon, OSUC 247962 (deposited in MBBJ). *Paratypes*: (2 females, 2 males) **BRUNEI**: 1 female, OSUC 376627 (BMNH). **INDONESIA**: 1 female, 2 males, OSUC 464006 (CNCI); OSUC 257046 (MBBJ); OSUC 247847 (ROME).

#### Comments.

The gena in *Oxyscelio angustifrons* lacks any strong carina between the anterior one (continuous with the hyperoccipital carina) and the posterior genal carina. This state differs from that of most other species of *Oxyscelio*, but nearly all *Oxyscelio* have strong variation in the distinctness of carinae in this part of the gena.

### Oxyscelio angustinubbin

Burks sp. n.

urn:lsid:zoobank.org:act:A087A8B3-6ED2-499B-9205-95ED2CC7B795

urn:lsid:biosci.ohio-state.edu:osuc_concepts:275518

http://species-id.net/wiki/Oxyscelio_angustinubbin

**Figures 26–31.**
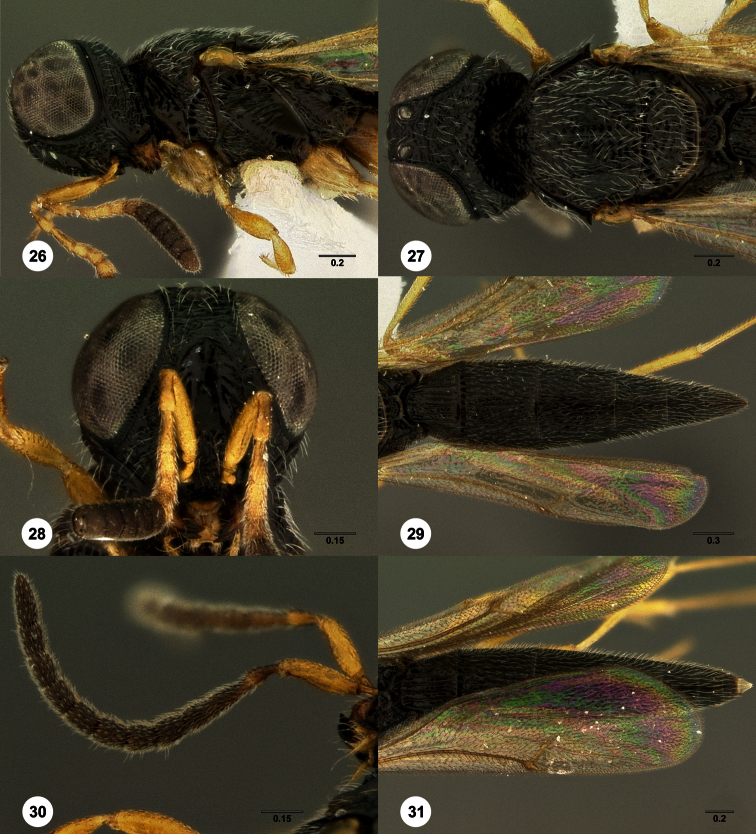
*Oxyscelio angustinubbin* sp. n., holotype female (OSUC 58667) **26** Head and mesosoma, lateral view **27** Head and mesosoma, dorsal view **28** Head, anterior view **29** Metasoma, dorsal view. Paratype male (OSUC 369233) **30** Antenna **31** Metasoma, dorsal view. Morphbank^29^

Figures 26–31 Morphbank^29^

#### Description.

*Female*. Body length 4.15–4.35 mm (n=4).

Radicle color: same color as scape. Scape color: Yellowish. A4: longer than broad. A5: longer than broad. Antennal club: formed, segments compact.

Interantennal process: not elongate. Median longitudinal elevation in frontal depression: absent. Frontal depression: concave. Frontal depression sculpture: with 3 or more broadly interrupted transverse carinae; with 3-5 complete transverse carinae. Submedian carina: strong, formed by a sharp raised carina. Submedian carina medially: without peak. Concavity across dorsal part of frontal depression: absent. Depression extending ventrally from median ocellus: absent. Upper frons: not hood-like. Malar area near antennal foramen: with oblique tooth-like flange (facial nubbin). Malar area at mouth corner: with one carina. Smooth strip along posterior side of malar sulcus: absent or not consistently broad. Middle genal carina: present. Direction of middle genal carina dorsally: parallel to eye margin. Major sculpture of gena anteriorly: umbilicate-foveate; rugose. Major sculpture of gena posteriorly: umbilicate-foveate; rugose. Microsculpture of gena anteroventrally: absent. Microsculpture of gena posteroventrally: absent. Median carina extending posteriorly from hyperoccipital carina: absent. Hyperoccipital carina: indicated by rugae. Lateral connection between hyperoccipital and occipital carinae: present as a weak elevation. Area between vertex and occipital carina: umbilicate-foveate. Occipital carina medially: absent. Lateral corners of occipital carina: sharp and protruding.

Lateral pronotal area: without bulge projecting towards anterior pit. Epomial corner: strong. Netrion surface anteriorly: not inflexed. Mesoscutum anteriorly: not steep. Mesoscutal median carina: present and complete. Longitudinal carina between median carina and notauli: absent. Major sculpture of medial mesoscutum anteriorly: umbilicate-foveate. Major sculpture of medial mesoscutum posteriorly: umbilicate-foveate. Microsculpture of medial mesoscutum anteriorly: granulate. Microsculpture of medial mesoscutum posteriorly: absent. Major sculpture of mesoscutellum: umbilicate-foveate; irregularly rugose. Microsculpture of mesoscutellum medially: absent. Microsculpture of mesoscutellum laterally: absent. Mesoscutellar apex: convex or straight. Setae along anterior limit of femoral depression: arising from rows of foveae.

Number of carinae crossing speculum above femoral depression: 3. Number of carinae crossing femoral depression: 3-5. Mesepimeral sulcus pits: more than 5. Metascutellum dorsally: concave. Metascutellar sculpture dorsally: smooth or with transverse carinae. Median carina of metascutellum: absent or branched. Metascutellar setae: absent. Metascutellar apex: weakly emarginate. Metapleuron above ventral metapleural area: crossed by carinae. Metasomal depression setae: absent. Lateral propodeal carinae anteromedially: strongly diverging. Anterior areoles of metasomal depression: absent. Anterior longitudinal carinae in metasomal depression: absent. Lateral propodeal areas: separated medially. Postmarginal vein: absent. Fore wing apex: reaching middle of T5.

T1 midlobe: with 6 or more longitudinal carinae. T1: without anterior bulge. T2: with straight longitudinal striae or rugae. T6: longer than broad. Apical flange of T6: not exposed apically. Metasomal apex: tapering to a sharp point. Major sculpture of T6: umbilicate-punctate. Microsculpture of T6: granulate.

*Male*.Body length 4.25 mm (n=1). A5 tyloid: carina-like, not expanded. A11: longer than broad. Median tooth of frontal depression: absent. Median lobe of T1: with 5 longitudinal carinae. Metasomal apex: with acuminate lateral corners.

#### Diagnosis.

Both sexes: Face with a narrow oblique flange between antennal foramen and eye. Metascutellum not convex, not emarginate posteriorly. Female: Antennal club formed. A4 longer than broad. Fore wing long enough to reach middle of T5. T1 without anterior horn, midlobe with 6 longitudinal carinae. Male: A11 longer than broad. T1 midlobe with 5 longitudinal carinae. T7 with weakly acuminate posterolateral corners. *Oxyscelio angustinubbin* is similar to *Oxyscelio foveatus* and some other species with a broad metascutellum and oblique flange between the antennal foramen and eye. It is distinctive in entirely lacking a T1 horn in females, and can be recognized through several additional sculptural features. Especially, the oblique facial flange is much narrower than that the broad sculptured flange of *Oxyscelio foveatus*.

#### Etymology.

Compound noun meaning “narrow nubbin.” Refers to the smaller than usual, smooth oblique flange between the antennal foramen and the eye.

#### Link to distribution map.

[http://hol.osu.edu/map-full.html?id=275518]

#### Material examined.

Holotype, female: **INDONESIA**: Sulawesi Utara Prov., “Edwards”, Toraut, Bogani Nani Wartabone (Dumoga-Bone) National Park, 680m, 26.IV-7.VI.1985, J. H. Martin, OSUC 58667 (deposited in BMNH).*Paratypes*: **INDONESIA**: 3 females, 1 male, OSUC 376617 (BMNH); OSUC 369233, 369291, 369298 (CNCI).

#### Comments.

The known male exhibits a narrower, more apically rounded metascutellum than in the females.

### Oxyscelio arcus

Burks sp. n.

urn:lsid:zoobank.org:act:B65A97D2-C8D7-46D9-9C4F-7B0EB305EBA2

urn:lsid:biosci.ohio-state.edu:osuc_concepts:275557

http://species-id.net/wiki/Oxyscelio_arcus

**Figures 32–37.**
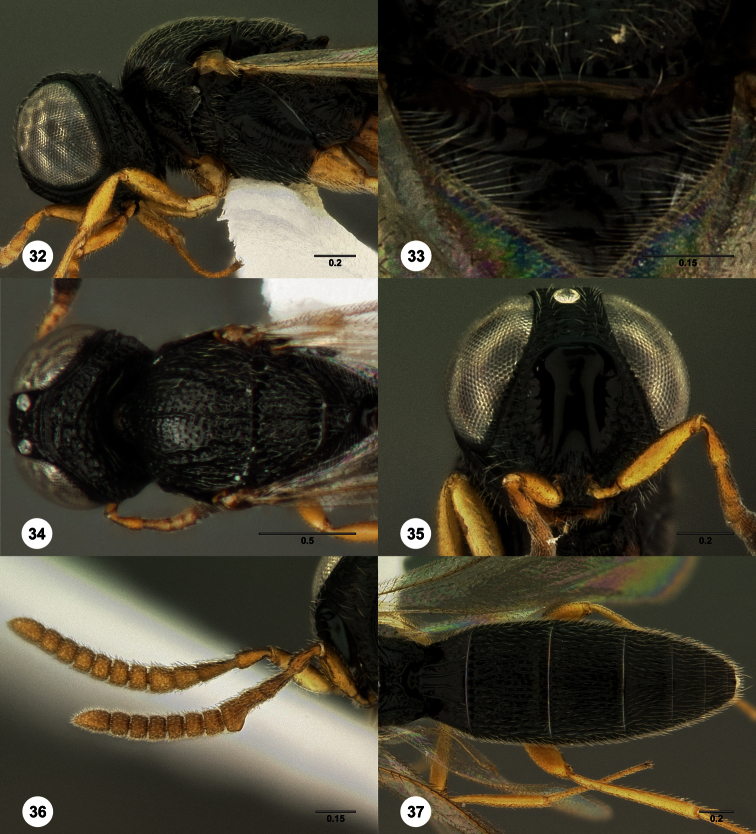
*Oxyscelio arcus* sp. n., paratype female (OSUC 368627) **32** Head and mesosoma, lateral view **33** Propodeum, posterior view. Paratype female (OSUC 285223) **34** Head and mesosoma, dorsal view. Paratype female (OSUC 369077) **35** Head, anterior view. Paratype male (OSUC 254743) **36** Antenna **37** Metasoma, dorsal view. Morphbank^30^

Figures 32–37 Morphbank^30^

#### Description.

*Female*. Body length 3.45–3.95 mm (n=14).

Radicle color: same color as scape. Scape color: Yellowish. A4: broader than long. A5: broader than long. Antennal club: formed, segments compact.

Interantennal process: not elongate. Median longitudinal elevation in frontal depression: absent. Frontal depression: concave. Frontal depression sculpture: with 3 or more broadly interrupted transverse carinae. Submedian carina: strong, formed by a sharp raised carina. Submedian carina medially: without peak. Concavity across dorsal part of frontal depression: absent. Depression extending ventrally from median ocellus: absent. Upper frons: not hood-like. Malar area near antennal foramen: without carina or expansion. Malar area at mouth corner: with radiating striae. Smooth strip along posterior side of malar sulcus: absent or not consistently broad. Middle genal carina: present. Direction of middle genal carina dorsally: parallel to eye margin. Major sculpture of gena anteriorly: umbilicate-foveate; rugose. Major sculpture of gena posteriorly: umbilicate-foveate; rugose. Microsculpture of gena anteroventrally: granulate. Microsculpture of gena posteroventrally: granulate. Median carina extending posteriorly from hyperoccipital carina: absent. Hyperoccipital carina: complete, continuous with anterior genal carina. Lateral connection between hyperoccipital and occipital carinae: absent. Area between vertex and occipital carina: with transverse carinae; irregularly rugose. Occipital carina medially: sinuate, concave medial to corners, but without a median peak. Lateral corners of occipital carina: sharp and protruding.

Lateral pronotal area: without bulge projecting towards anterior pit. Epomial corner: weak. Netrion surface anteriorly: not inflexed. Mesoscutum anteriorly: steep. Mesoscutal median carina: present and complete. Longitudinal carina between median carina and notauli: absent. Major sculpture of medial mesoscutum anteriorly: umbilicate-punctate. Major sculpture of medial mesoscutum posteriorly: umbilicate-punctate. Microsculpture of medial mesoscutum anteriorly: granulate. Microsculpture of medial mesoscutum posteriorly: absent. Major sculpture of mesoscutellum: umbilicate-foveate; longitudinally rugose. Microsculpture of mesoscutellum medially: absent. Microsculpture of mesoscutellum laterally: absent. Mesoscutellar apex: convex or straight. Setae along anterior limit of femoral depression: arising from rows of foveae. Number of carinae crossing speculum above femoral depression: 4. Number of carinae crossing femoral depression: more than 5. Mesepimeral sulcus pits: more than 5. Metascutellum dorsally: concave. Metascutellar sculpture dorsally: with scattered rugae. Median carina of metascutellum: absent or branched. Metascutellar setae: with many dorsal setae. Metascutellar apex: weakly emarginate. Metapleuron above ventral metapleural area: crossed by carinae. Metasomal depression setae: absent. Lateral propodeal carinae anteromedially: weakly diverging. Anterior areoles of metasomal depression: one or more areoles present. Anterior longitudinal carinae in metasomal depression: absent. Lateral propodeal areas: separated medially. Postmarginal vein: present. Fore wing apex: reaching middle of T6.

T1 midlobe: with 4 longitudinal carinae. T1: without anterior bulge. T2: with straight longitudinal striae or rugae. T6: broader than long. Apical flange of T6: exposed apically. Metasomal apex: rounded. Major sculpture of T6: umbilicate-punctate; longitudinally striate or rugose. Microsculpture of T6: absent.

*Male*. Body length 3.4–3.6 mm (n=5). A5 tyloid: carina-like, not expanded. A11: broader than long. Median tooth of frontal depression: absent. Median lobe of T1: with 4 longitudinal carinae. Metasomal apex: with no distinct corners.

#### Diagnosis.

Both sexes: Frons without elevation between antennal foramen and eye. Hyperoccipital carina present, continuous with anterior genal carina. Medial mesoscutum with strong sculpture, but without longitudinal rugae. Metascutellum with dorsal setae. Female: A4, A5 broader than long T1 with 4 longitudinal carinae. Male: A5 tyloid expanded. A11 broader than long. Frontal depression without tooth-like median protrusion dorsally. T1 midlobe with 4 longitudinal carinae. T7 without protruding posterolateral corners. *Oxyscelio arcus* is very similar to *Oxyscelio ceylonensis* and *Oxyscelio unguis*. It differs from them both in sculpture and body shape, and in other features such as T1 midlobe carinae and the apex of T7.

#### Etymology.

Latin noun, genitive case, meaning “arch.”

#### Link to distribution map.

[http://hol.osu.edu/map-full.html?id=275557]

#### Material examined.

Holotype, female: **THAILAND**: Phitsanulok Prov., mixed deciduous forest, T920, Thung Salaeng Luang National Park, 16°50.563'N, 100°51.757'E, 481m, 12.IX–13.IX.2006, pan trap, Pongpitak & Pranee, OSUC 361931 (deposited in QSBG). *Paratypes*: (12 females, 7 males) **INDONESIA**: 3 females, OSUC 369077, 369079 (CNCI); ROMEnt Spec. No. 112246 (ROME). **THAILAND**: 10 females, 7 males, OSUC 368627, 368690, 368745, 464056 (CNCI); OSUC 224350, 247657336161, 352486, 361962 (OSUC); OSUC 247624, 254743, 336708, 361285-361286, 368520 (QSBG); OSUC 285223, 336707 (WINC).

### Oxyscelio arvi

Burks sp. n.

urn:lsid:zoobank.org:act:491CF086-1501-4C6C-BB0E-1A9588FECF4F

urn:lsid:biosci.ohio-state.edu:osuc_concepts:275548

http://species-id.net/wiki/Oxyscelio_arvi

**Figures 38–41.**
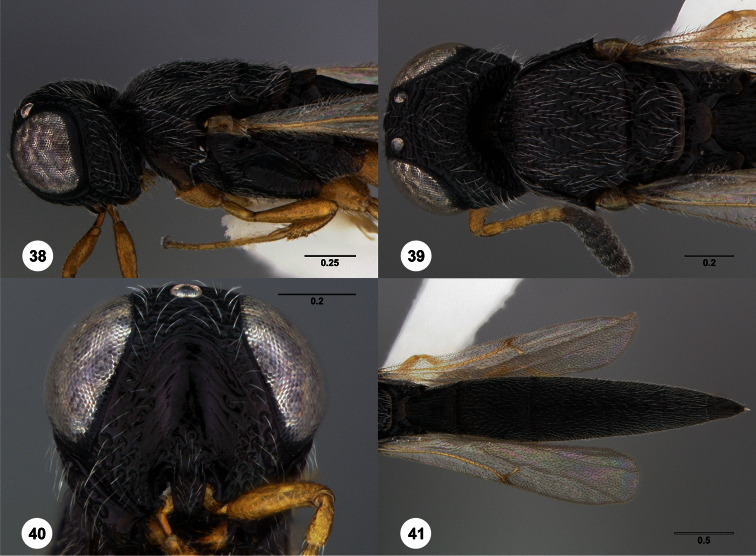
*Oxyscelio arvi* sp. n., paratype female (OSUC 368988) **38** Head and mesosoma, lateral view **39** Head and mesosoma, dorsal view **40** Head, anterior view **41** Metasoma, dorsal view. Morphbank^31^

Figures 38–41 Morphbank^31^

#### Description.

*Female*. Body length 4.35–4.45 mm (n=3).

Radicle color: same color as scape. Scape color: Yellowish. A4: longer than broad. A5: longer than broad. Antennal club: formed, segments compact.

Interantennal process: not elongate. Median longitudinal elevation in frontal depression: absent. Frontal depression: concave. Frontal depression sculpture: with 1 complete transverse carina. Submedian carina: strong, formed by a sharp raised carina. Submedian carina medially: without peak. Concavity across dorsal part of frontal depression: absent. Depression extending ventrally from median ocellus: absent. Upper frons: not hood-like. Malar area near antennal foramen: without carina or expansion. Malar area at mouth corner: with radiating striae. Smooth strip along posterior side of malar sulcus: absent or not consistently broad. Middle genal carina: present. Direction of middle genal carina dorsally: absent (replace with question mark). Major sculpture of gena anteriorly: umbilicate-foveate. Major sculpture of gena posteriorly: rugose; umbilicate-punctate. Microsculpture of gena anteroventrally: absent. Microsculpture of gena posteroventrally: absent. Median carina extending posteriorly from hyperoccipital carina: absent. Hyperoccipital carina: indicated by rugae. Lateral connection between hyperoccipital and occipital carinae: absent. Area between vertex and occipital carina: umbilicate-foveate. Occipital carina medially: uniformly rounded. Lateral corners of occipital carina: not protruding.

Lateral pronotal area: without bulge projecting towards anterior pit. Epomial corner: strong. Netrion surface anteriorly: not inflexed. Mesoscutum anteriorly: not steep. Mesoscutal median carina: present and complete. Longitudinal carina between median carina and notauli: absent. Major sculpture of medial mesoscutum anteriorly: umbilicate-foveate. Major sculpture of medial mesoscutum posteriorly: umbilicate-foveate. Microsculpture of medial mesoscutum anteriorly: granulate. Microsculpture of medial mesoscutum posteriorly: absent. Major sculpture of mesoscutellum: umbilicate-foveate. Microsculpture of mesoscutellum medially: granulate. Microsculpture of mesoscutellum laterally: granulate. Mesoscutellar apex: convex or straight. Setae along anterior limit of femoral depression: arising from rows of foveae. Number of carinae crossing speculum above femoral depression: 3. Number of carinae crossing femoral depression: 3-5. Mesepimeral sulcus pits: more than 5. Metascutellum dorsally: convex. Metascutellar sculpture dorsally: smooth or with transverse carinae. Median carina of metascutellum: absent or branched. Metascutellar setae: absent. Metascutellar apex: convex or straight. Metapleuron above ventral metapleural area: crossed by carinae. Metasomal depression setae: absent. Lateral propodeal carinae anteromedially: strongly diverging. Anterior areoles of metasomal depression: absent. Anterior longitudinal carinae in metasomal depression: absent. Lateral propodeal areas: separated medially. Postmarginal vein: present. Fore wing apex: reaching apex of T4.

T1 midlobe: obscured by other raised sculpture. T1: with long anterior bulge, reaching metascutellum. T2: with straight longitudinal striae or rugae. T6: longer than broad. Apical flange of T6: not exposed apically. Metasomal apex: rounded. Major sculpture of T6: umbilicate-punctate; longitudinally striate or rugose. Microsculpture of T6: granulate.

*Male*. Unknown.

#### Diagnosis.

Female: Upper frons without additional carinae dorsal to submedian carina, but frontal depression with sharply arched carinae ventral to submedian carina. Hyperoccipital carina indicated by rugae. Mesoscutellum with granulate sculpture posterolaterally. Mesofemoral depression crossed by more than 3 carinae below speculum. Mesopleuron along anteroventral edge of femoral depression with rows of weak pits or foveae that are separated by a broad smooth strip. Metascutellum subrectangular, with scattered rugae. T1 midlobe with strong anterior horn. T2 without sublateral depressions or curved striae. T6 longer than broad, tapering to a rounded apex. *Oxyscelio arvi* is very similar to *Oxyscelio florus* and the Taiwanese species *Oxyscelio dermatoglyphes*, but differs in mesosomal sculpture. It is unique in having a smooth strip interrupting the rows of setae along the anterior edge of the femoral depression.

#### Etymology.

Latin noun, genitive case, meaning “field.”

#### Link to distribution map.

[http://hol.osu.edu/map-full.html?id=275548]

#### Material examined.

Holotype, female: **JAPAN**: Tochigi Pref., Honshu, Nishi-nasuno, National Grassland Research Institute (NGRI), 500m, 10.VIII.1989, sweeping, M. J. Sharkey, OSUC 368989 (deposited in CNCI). *Paratypes*:**JAPAN**: 2 females, OSUC 368990 (CNCI); OSUC 368988 (OSUC).

### Oxyscelio asperi

Burks sp. n.

urn:lsid:zoobank.org:act:FC85267B-BCFC-42DB-B166-10DD2D98CEE5

urn:lsid:biosci.ohio-state.edu:osuc_concepts:275569

http://species-id.net/wiki/Oxyscelio_asperi

**Figures 42–47.**
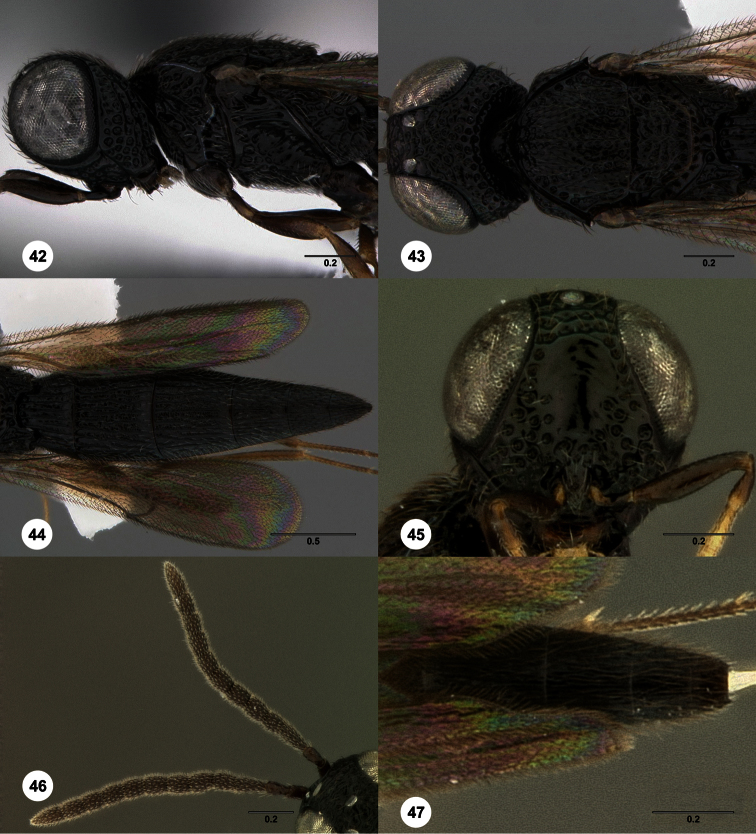
*Oxyscelio asperi* sp. n., holotype female (OSUC 368934) **42** Head and mesosoma, lateral view **43** Head and mesosoma, dorsal view **44** Metasoma, dorsal view. Paratype female (OSUC 368924) **45** Head, anterior view. Paratype male (OSUC 368933) **46** Antenna **47** Metasoma, dorsal view. Morphbank^32^

Figures 42–47 Morphbank^32^

#### Description.

*Female*. Body length 3.35–3.6 mm (n=6).

Radicle color: darker than scape. Scape color: Brown. A4: longer than broad. A5: broader than long; as long as broad. Antennal club: formed, segments compact.

Interantennal process: not elongate. Median longitudinal elevation in frontal depression: absent. Frontal depression: concave. Frontal depression sculpture: with 2 complete transverse carinae. Submedian carina: strong, formed by a sharp raised carina. Submedian carina medially: without peak. Concavity across dorsal part of frontal depression: absent. Depression extending ventrally from median ocellus: absent. Upper frons: not hood-like. Malar area near antennal foramen: without carina or expansion. Malar area at mouth corner: with one carina. Smooth strip along posterior side of malar sulcus: absent or not consistently broad. Middle genal carina: present. Direction of middle genal carina dorsally: parallel to eye margin. Major sculpture of gena anteriorly: umbilicate-foveate. Major sculpture of gena posteriorly: umbilicate-foveate; rugose. Microsculpture of gena anteroventrally: absent. Microsculpture of gena posteroventrally: absent. Median carina extending posteriorly from hyperoccipital carina: absent. Hyperoccipital carina: not indicated medially. Lateral connection between hyperoccipital and occipital carinae: absent. Area between vertex and occipital carina: umbilicate-foveate. Occipital carina medially: uniformly rounded. Lateral corners of occipital carina: not protruding.

Lateral pronotal area: without bulge projecting towards anterior pit. Epomial corner: strong. Netrion surface anteriorly: not inflexed. Mesoscutum anteriorly: not steep. Mesoscutal median carina: present and complete. Longitudinal carina between median carina and notauli: absent. Major sculpture of medial mesoscutum anteriorly: umbilicate-punctate; irregularly rugose. Major sculpture of medial mesoscutum posteriorly: umbilicate-foveate; irregularly rugose. Microsculpture of medial mesoscutum anteriorly: absent. Microsculpture of medial mesoscutum posteriorly: absent. Major sculpture of mesoscutellum: umbilicate-foveate; irregularly rugose. Microsculpture of mesoscutellum medially: absent. Microsculpture of mesoscutellum laterally: absent. Mesoscutellar apex: convex or straight. Setae along anterior limit of femoral depression: arising from rows of foveae. Number of carinae crossing speculum above femoral depression: 2. Number of carinae crossing femoral depression: more than 5. Mesepimeral sulcus pits: more than 5. Metascutellum dorsally: concave. Metascutellar sculpture dorsally: smooth or with transverse carinae. Median carina of metascutellum: absent or branched. Metascutellar setae: absent. Metascutellar apex: convex or straight. Metapleuron above ventral metapleural area: foveate or rugose. Metasomal depression setae: absent. Lateral propodeal carinae anteromedially: strongly diverging. Anterior areoles of metasomal depression: absent. Anterior longitudinal carinae in metasomal depression: absent. Lateral propodeal areas: separated medially. Postmarginal vein: present. Fore wing apex: reaching middle of T5.

T1 midlobe: with 5 longitudinal carinae. T1: without anterior bulge. T2: with straight longitudinal striae or rugae. T6: longer than broad. Apical flange of T6: exposed apically. Metasomal apex: rounded. Major sculpture of T6: umbilicate-punctate. Microsculpture of T6: granulate.

*Male*:Body length 3.15–3.5 mm (n=6). A5 tyloid: carina-like, not expanded. A11: longer than broad. Median tooth of frontal depression: absent. Median lobe of T1: with 3 longitudinal carinae. Metasomal apex: with acuminate lateral corners.

#### Diagnosis.

Both sexes: Middle genal carina weak, subparallel with eye margin. Hyperoccipital carina absent. Mesoscutellum without granulate sculpture. Metascutellum tiny, concave dorsally, smooth aside from some transverse carinae. Metapleuron not crossed by carinae above lower metapleural area, instead with rough irregular sculpture. Female: A4, A5 longer than broad. T1 midlobe with 5 longitudinal carinae. T6 longer than broad. Male: A11 longer than broad. A5 tyloid expanded, sinuate or teardrop-shaped. T7 with short, sharp and protruding posterolateral corners. *Oxyscelio asperi* is very similar to *Oxyscelio crebritas*, but is entirely dark brown in color, lacks straight carinae on the metapleuron, and has at most one straight carina crossing the mesofemoral depression. It is also distinctive within the *crebritas*-group in having relatively long A4 and A5.

#### Etymology.

Latin noun (2nd declension: asperum, -i), genitive case, meaning “uneven landscape.” Refers to the unusual and irregular metapleural sculpture.

**Link to distribution map**. [http://hol.osu.edu/map-full.html?id=275569]

**Associations.** Unspecified association Uncaria Schreber: [Rubiales: Rubiaceae]

#### Material examined.

Holotype, female: **INDONESIA**: Maluku Prov., Ceram (Seram) Isl., Solea, VIII-1987, malaise trap, M. C. Day, OSUC 368934 (deposited in BMNH). *Paratypes*: **INDONESIA**: 6 females, 6 males, OSUC 368924-368928, 368932, 368935, 368937 (CNCI); OSUC 368929, 368931, 368933, 368936 (OSUC).

#### Comments.

*Oxyscelio asperi* is known only from Seram, and was initially assessed as just a regionally dark form of *Oxyscelio crebritas*. It is recognized as distinct on strength of the metapleural and mesopleural sculpture.

### Oxyscelio aureamediocritas

Burks sp. n.

urn:lsid:zoobank.org:act:EDAA6F52-41B2-4C11-870F-568EB113307F

urn:lsid:biosci.ohio-state.edu:osuc_concepts:275486

http://species-id.net/wiki/Oxyscelio_aureamediocritas

**Figures 48–51.**
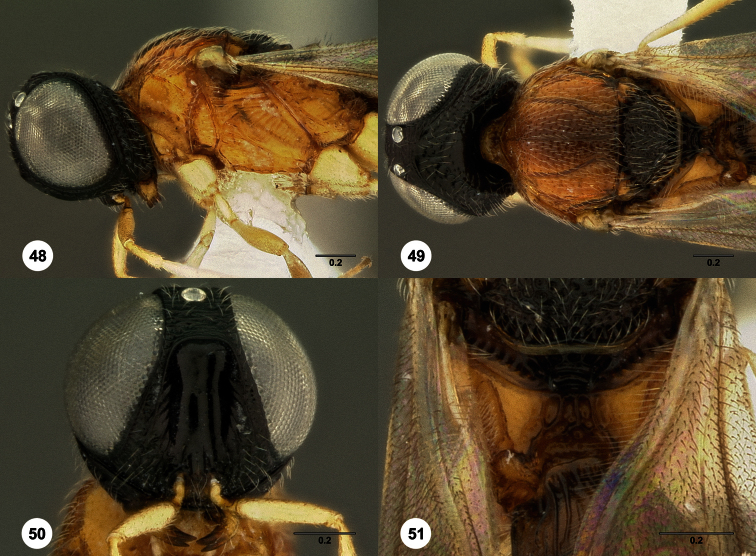
*Oxyscelio aureamediocritas* sp. n., holotype female (OSUC 280518) **48** Head and mesosoma, lateral view **49** Head and mesosoma, dorsal view **50** Head, anterior view **51** Propodeum, posterior view. Morphbank^33^

Figures 48–51 Morphbank^33^

#### Description.

*Female*. Body length 3.45 mm (n=1).

Radicle color: darker than scape. Scape color: Yellowish. A4: longer than broad. A5: longer than broad. Antennal club: formed, segments compact.

Interantennal process: not elongate. Median longitudinal elevation in frontal depression: absent. Frontal depression: concave. Frontal depression sculpture: with 3 or more broadly interrupted transverse carinae. Submedian carina: strong, formed by a sharp raised carina. Submedian carina medially: without peak. Concavity across dorsal part of frontal depression: absent. Depression extending ventrally from median ocellus: absent. Upper frons: hood-like, protruding over pedicel when antenna at rest. Malar area near antennal foramen: without carina or expansion. Malar area at mouth corner: with radiating striae. Smooth strip along posterior side of malar sulcus: absent or not consistently broad. Middle genal carina: present. Direction of middle genal carina dorsally: parallel to eye margin. Major sculpture of gena anteriorly: umbilicate-foveate; rugose. Major sculpture of gena posteriorly: umbilicate-foveate; rugose. Microsculpture of gena anteroventrally: absent. Microsculpture of gena posteroventrally: absent. Median carina extending posteriorly from hyperoccipital carina: absent. Hyperoccipital carina: complete, continuous with anterior genal carina. Lateral connection between hyperoccipital and occipital carinae: absent. Area between vertex and occipital carina: crenulate; umbilicate-punctate. Occipital carina medially: convex, with a sharp median peak. Lateral corners of occipital carina: not protruding.

Lateral pronotal area: without bulge projecting towards anterior pit. Epomial corner: weak. Netrion surface anteriorly: not inflexed. Mesoscutum anteriorly: not steep. Mesoscutal median carina: present and complete. Longitudinal carina between median carina and notauli: absent. Major sculpture of medial mesoscutum anteriorly: umbilicate-foveate. Major sculpture of medial mesoscutum posteriorly: umbilicate-punctate; longitudinally rugose. Microsculpture of medial mesoscutum anteriorly: granulate. Microsculpture of medial mesoscutum posteriorly: absent. Major sculpture of mesoscutellum: umbilicate-foveate; irregularly rugose. Microsculpture of mesoscutellum medially: absent. Microsculpture of mesoscutellum laterally: absent. Mesoscutellar apex: convex or straight. Setae along anterior limit of femoral depression: arising from tiny pits. Number of carinae crossing speculum above femoral depression: 3. Number of carinae crossing femoral depression: more than 5. Mesepimeral sulcus pits: more than 5. Metascutellum dorsally: concave. Metascutellar sculpture dorsally: smooth or with transverse carinae. Median carina of metascutellum: absent or branched. Metascutellar setae: absent. Metascutellar apex: deeply emarginate. Metapleuron above ventral metapleural area: smooth. Metasomal depression setae: absent. Lateral propodeal carinae anteromedially: weakly diverging. Anterior areoles of metasomal depression: one or more areoles present. Anterior longitudinal carinae in metasomal depression: median carina present. Lateral propodeal areas: separated medially. Postmarginal vein: present. Fore wing apex: reaching beyond T6.

T1 midlobe: with 5 longitudinal carinae. T1: without anterior bulge. T2: with straight longitudinal striae or rugae. T6: broader than long. Apical flange of T6: exposed apically. Metasomal apex: rounded. Major sculpture of T6: umbilicate-punctate. Microsculpture of T6: absent.

*Male*. Unknown.

#### Diagnosis.

Female: A4, A5 longer than broad. Frons without elevation between antennal foramen and eye. Hyperoccipital carina present, continuous with anterior genal carina. Metascutellum deeply emarginate. Metasomal depression elongate, with median carina; lateral propodeal carinae narrowly separated anteriorly. T1 midlobe with 5 longitudinal carinae. T6 rounded apically. *Oxyscelio aureamediocritas* is similar to *Oxyscelio convergens* and other species with a sculptured and conspicuous metasomal depression, and with the flagellum in females having an elongate A4 and A5 and weakly developed club. It differs in having a propodeal median carina. Although males of this species are unknown, patterns of variation in other species of *Oxyscelio* suggest that males may lack the median propodeal carina.

#### Etymology. 

Latin noun in apposition to the generic name, based on “The Golden Mean” coined by Horace. Refers to the median propodeal carina and general golden color of the holotype.

#### Link to distribution map.

[http://hol.osu.edu/map-full.html?id=275486]

#### Material examined.

Holotype, female: **THAILAND**: Phetchabun Prov., Hell, evergreen forest, T1329, Nam Nao National Park, 16°44.402'N, 101°34.560'E, 883m, 27.XI-4.XII.2006, malaise trap, N. Hongyothi, OSUC 280518 (deposited in QSBG).

### Oxyscelio bipunctuum

Burks sp. n.

urn:lsid:zoobank.org:act:714F9E81-51B3-4696-9B30-B6B05CBF4CDB

urn:lsid:biosci.ohio-state.edu:osuc_concepts:275497

http://species-id.net/wiki/Oxyscelio_bipunctuum

**Figures 52–55.**
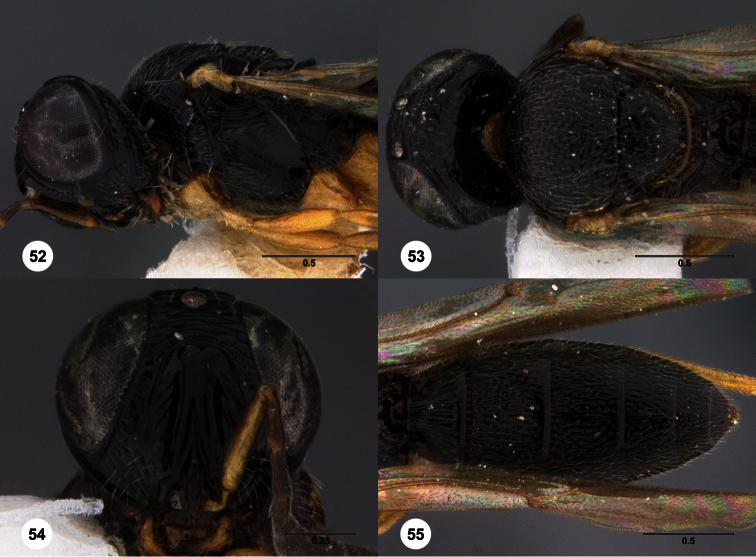
*Oxyscelio bipunctuum* sp. n., paratype female (OSUC 376572) **52** Head and mesosoma, lateral view. Holotype female (OSUC 369048) **53** Head and mesosoma, dorsal view **54** Head, anterior view **55** Metasoma, dorsal view. Morphbank^34^

Figures 52–55 Morphbank^34^

#### Description.

*Female*. Body length 3.75–3.8 mm (n=2).

Radicle color: darker than scape. Scape color: Yellowish. A4: broader than long. A5: broader than long. Antennal club: formed, segments compact.

Interantennal process: not elongate. Median longitudinal elevation in frontal depression: present. Frontal depression: concave. Frontal depression sculpture: with 3 or more broadly interrupted transverse carinae. Submedian carina: strong, formed by a sharp raised carina. Submedian carina medially: without peak. Concavity across dorsal part of frontal depression: absent. Depression extending ventrally from median ocellus: absent. Upper frons: not hood-like. Malar area near antennal foramen: without carina or expansion. Malar area at mouth corner: with radiating striae. Smooth strip along posterior side of malar sulcus: absent or not consistently broad. Middle genal carina: absent. Direction of middle genal carina dorsally: absent (replace with question mark). Major sculpture of gena anteriorly: umbilicate-punctate. Major sculpture of gena posteriorly: umbilicate-punctate. Microsculpture of gena anteroventrally: absent. Microsculpture of gena posteroventrally: absent. Median carina extending posteriorly from hyperoccipital carina: absent. Hyperoccipital carina: complete, continuous with anterior genal carina. Lateral connection between hyperoccipital and occipital carinae: absent. Area between vertex and occipital carina: umbilicate-punctate. Occipital carina medially: uniformly rounded; absent. Lateral corners of occipital carina: not protruding.

Lateral pronotal area: without bulge projecting towards anterior pit. Epomial corner: weak. Netrion surface anteriorly: not inflexed. Mesoscutum anteriorly: not steep. Mesoscutal median carina: present and complete. Longitudinal carina between median carina and notauli: absent. Major sculpture of medial mesoscutum anteriorly: umbilicate-foveate. Major sculpture of medial mesoscutum posteriorly: umbilicate-foveate. Microsculpture of medial mesoscutum anteriorly: granulate. Microsculpture of medial mesoscutum posteriorly: absent. Major sculpture of mesoscutellum: umbilicate-punctate. Microsculpture of mesoscutellum medially: absent; granulate. Microsculpture of mesoscutellum laterally: granulate. Mesoscutellar apex: convex or straight. Setae along anterior limit of femoral depression: arising from tiny pits. Number of carinae crossing speculum above femoral depression: 3; 4. Number of carinae crossing femoral depression: 3-5. Mesepimeral sulcus pits: 3-5. Metascutellum dorsally: concave. Metascutellar sculpture dorsally: smooth or with transverse carinae. Median carina of metascutellum: absent or branched. Metascutellar setae: absent. Metascutellar apex: deeply emarginate. Metapleuron above ventral metapleural area: smooth. Metasomal depression setae: absent. Lateral propodeal carinae anteromedially: weakly diverging. Anterior areoles of metasomal depression: absent. Anterior longitudinal carinae in metasomal depression: median carina present. Lateral propodeal areas: separated medially. Postmarginal vein: present. Fore wing apex: reaching beyond T6.

T1 midlobe: with 4 longitudinal carinae. T1: without anterior bulge. T2: with straight longitudinal striae or rugae. T6: broader than long. Apical flange of T6: exposed apically. Metasomal apex: rounded. Major sculpture of T6: umbilicate-punctate. Microsculpture of T6: absent.

*Male*. Body length 3.55–3.65 mm (n=2). A5 tyloid: carina-like, not expanded. A11: longer than broad. Median tooth of frontal depression: present. Median lobe of T1: with 4 longitudinal carinae. Metasomal apex: with no distinct corners.

#### Diagnosis.

Both sexes: Frons without elevation between antennal foramen and eye. Hyperoccipital carina present, continuous with anterior genal carina. Metascutellum deeply emarginate. Metasomal depression elongate, with a pair of areoles separated by a short median carina; lateral propodeal carinae narrowly separated anteriorly. Female: A4, A5 broader than long. T1 midlobe with 4 longitudinal carinae. T6 rounded apically. Male: Frontal depression with tooth-like median protrusion dorsally. T1 midlobe with 4 longitudinal carinae. T7 without distinct posterolateral corners. *Oxyscelio bipunctuum* differs from other species of the *Oxyscelio convergens*-complex in having a median protrusion dorsomedially from the frontal depression in males. This character also occurs in some species of the *Oxyscelio mesiodentis*-complex, but these species do not otherwise strongly resemble *Oxyscelio bipunctuum*.

#### Etymology.

Latin noun, 4th declension, plural genitive case. Refers to the two small areoles on the metasomal depression.

#### Link to distribution map.

[http://hol.osu.edu/map-full.html?id=275497]

#### Material examined.

Holotype, female: **INDIA**: Tamil Nadu St., Palni (Pulney) Hills, Kodaikanal, 6500ft, V-1953, P. S. Nathan, OSUC 369048 (deposited in CNCI). *Paratypes*: **INDIA**: 2 females, 2 males, OSUC 376572-376575 (BMNH).

### Oxyscelio brevidentis

Burks sp. n.

urn:lsid:zoobank.org:act:7BC1A5B2-D4A0-40FA-8AF4-D30DA93A1929

urn:lsid:biosci.ohio-state.edu:osuc_concepts:275563

http://species-id.net/wiki/Oxyscelio_brevidentis

**Figures 56–61.**
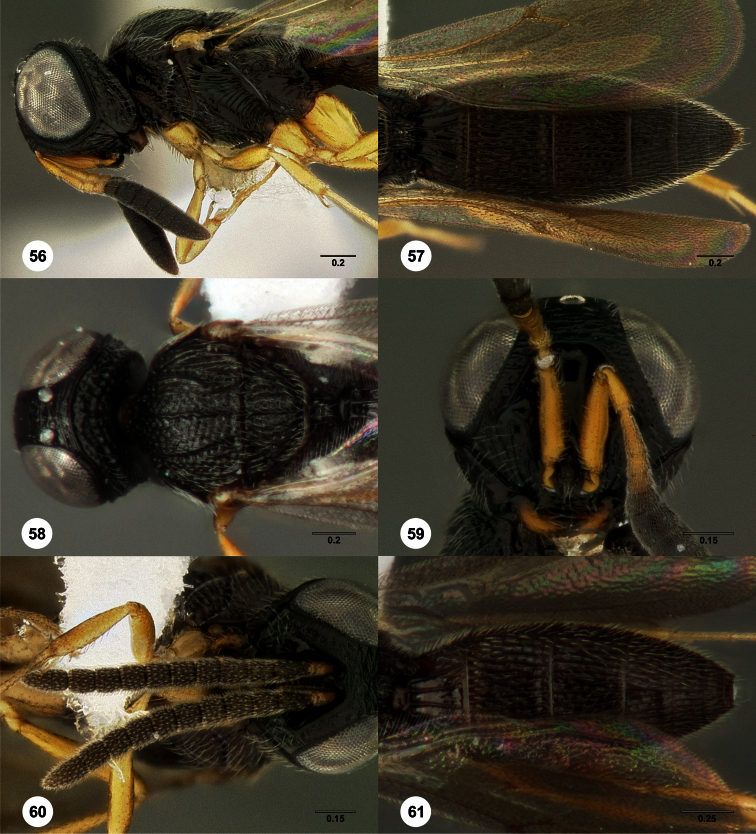
*Oxyscelio brevidentis* sp. n., paratype female (OSUC 317861) **56** Head and mesosoma, lateral view **57** Metasoma, dorsal view. Paratype female (OSUC 352487) **58** Head and mesosoma, dorsal view. Paratype female (OSUC 257385) **59** Head, anterior view Paratype male (OSUC 352455) **60** Antenna. Paratype male (OSUC 247910) **61** Metasoma, dorsal view. Morphbank^35^

Figures 56–61 Morphbank^35^

#### Description.

*Female*. Body length 2.7–3.75 mm (n=18).

Radicle color: same color as scape. Scape color: Yellowish. A4: broader than long. A5: broader than long. Antennal club: formed, segments compact.

Interantennal process: not elongate. Median longitudinal elevation in frontal depression: absent. Frontal depression: concave. Frontal depression sculpture: without transverse or oblique carinae below submedian carina. Submedian carina: strong, formed by a sharp raised carina. Submedian carina medially: without peak. Concavity across dorsal part of frontal depression: absent. Depression extending ventrally from median ocellus: absent. Upper frons: hood-like, protruding over pedicel when antenna at rest. Malar area near antennal foramen: without carina or expansion. Malar area at mouth corner: with radiating striae. Smooth strip along posterior side of malar sulcus: absent or not consistently broad. Middle genal carina: present. Direction of middle genal carina dorsally: parallel to eye margin. Major sculpture of gena anteriorly: rugose; umbilicate-punctate. Major sculpture of gena posteriorly: umbilicate-foveate; rugose. Microsculpture of gena anteroventrally: absent. Microsculpture of gena posteroventrally: absent. Median carina extending posteriorly from hyperoccipital carina: absent. Hyperoccipital carina: complete, continuous with anterior genal carina. Lateral connection between hyperoccipital and occipital carinae: absent. Area between vertex and occipital carina: irregularly rugose. Occipital carina medially: uniformly rounded. Lateral corners of occipital carina: sharp and protruding.

Lateral pronotal area: without bulge projecting towards anterior pit. Epomial corner: weak. Netrion surface anteriorly: not inflexed. Mesoscutum anteriorly: not steep. Mesoscutal median carina: present and complete. Longitudinal carina between median carina and notauli: absent. Major sculpture of medial mesoscutum anteriorly: umbilicate-foveate. Major sculpture of medial mesoscutum posteriorly: umbilicate-foveate. Microsculpture of medial mesoscutum anteriorly: granulate. Microsculpture of medial mesoscutum posteriorly: absent. Major sculpture of mesoscutellum: umbilicate-foveate; longitudinally rugose. Microsculpture of mesoscutellum medially: absent. Microsculpture of mesoscutellum laterally: absent. Mesoscutellar apex: convex or straight. Setae along anterior limit of femoral depression: arising from rows of foveae. Number of carinae crossing speculum above femoral depression: 3; 4. Number of carinae crossing femoral depression: more than 5. Mesepimeral sulcus pits: more than 5. Metascutellum dorsally: concave. Metascutellar sculpture dorsally: foveate. Median carina of metascutellum: absent or branched. Metascutellar setae: with many dorsal setae. Metascutellar apex: weakly emarginate. Metapleuron above ventral metapleural area: crossed by carinae. Metasomal depression setae: absent. Lateral propodeal carinae anteromedially: strongly diverging. Anterior areoles of metasomal depression: one or more areoles present. Anterior longitudinal carinae in metasomal depression: absent. Lateral propodeal areas: separated medially. Postmarginal vein: present. Fore wing apex: reaching apex of T6; reaching beyond T6. 1st metatarsomere less than 1.1x as long as metatarsomeres 2–5 combined.

T1 midlobe: with 4 longitudinal carinae. T1: without anterior bulge. T2: with straight longitudinal striae or rugae. T6: broader than long. Apical flange of T6: exposed apically. Metasomal apex: rounded. Major sculpture of T6: umbilicate-punctate; longitudinally striate or rugose. Microsculpture of T6: absent.

*Male*. Body length 2.7–3.25 mm (n=18). A5 tyloid: carina-like, not expanded. A11: broader than long; as long as broad. Median tooth of frontal depression: present. Median lobe of T1: with 4 longitudinal carinae. Metasomal apex: with acuminate lateral corners.

#### Diagnosis.

Both sexes: Face with vertical elevation between antennal foramen and eye. Hyperoccipital carina present, continuous with anterior genal carina. Medial mesoscutum and mesoscutellum with many strong longitudinal rugae. Metascutellum with dorsal setae. Lateral propodeal carinae strongly diverging. Female: A4, A5 broader than long. T1 with 4 longitudinal carinae. Male: A5 tyloid expanded. Frontal depression with tooth-like median protrusion dorsally. T1 midlobe with 3 longitudinal carinae. T7 without distinct posterolateral corners. *Oxyscelio brevidentis* and *Oxyscelio mesiodentis* are very similar, but differ mainly in the lateral propodeal carinae and size-related features, with metatarsomere length being the best diagnostic feature.

#### Etymology.

Latin noun, genitive case, meaning “short tooth.”

#### Link to distribution map.

[http://hol.osu.edu/map-full.html?id=275563]

#### Material examined.

Holotype, female: **THAILAND**: Ubon Ratchathani Prov., Rong Khi Noi (Rong Hi Noy), T1476, Pha Tam National Park, 15°40.021'N, 105°30.448'E, 240m, 1.I-7.I.2007, malaise trap, Thongkam & Pakdee, OSUC 285222 (deposited in QSBG). *Paratypes*: **THAILAND**: 20 females, 22 males, OSUC 247882, 247908 (BMNH); OSUC 317879, 320406, 368721, 368764, 464019, 464024, 464026, 464045, 464059-464060 (CNCI); OSUC 247598, 247899, 247909, 247921, 257385, 285228, 335631 (OSUC); OSUC 247649, 257401, 317861, 317869, 317873, 320384, 322083, 335547, 335986, 352453, 352455, 352487, 361186, 361200-361204, 361206, 361960 (QSBG); OSUC 247910, 285227, 320393 (WINC).

#### Comments.

The strong similarity between *Oxyscelio brevidentis* and *Oxyscelio mesiodentis* may indicate that they are really one species attacking a wide variety of hosts, but it seems best to verify this possibility before combining these two species.

### Oxyscelio brevinervis

(Kieffer)

urn:lsid:zoobank.org:act:9AF3CD19-A976-4478-B3E9-B54D93D3AA9B

urn:lsid:biosci.ohio-state.edu:osuc_concepts:5008

http://species-id.net/wiki/Oxyscelio_brevinervis

**Figures 62–63.**
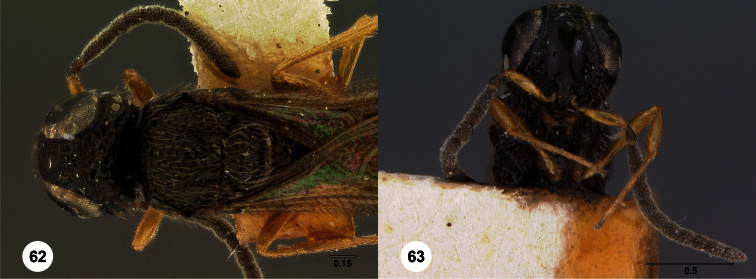
*Oxyscelio brevinervis* sp. n., neotype male (OSUC 268270) **62** Head and mesosoma, dorsal view **63** Head, anterior view. Morphbank^36^

Figures 62–63 Morphbank^36^

Camptoteleia brevinervis Kieffer, 1916: 171, 175 (original description, keyed); Kieffer 1926: 380, 384 (description, keyed).
Oxyscelio brevinervis (Kieffer): Dodd 1931: 74 (generic transfer).

#### Description.

*Female*. Unknown.

*Male*. Body length 3.4–3.45 mm (n=3).

Radicle color: same color as scape. Scape color: Yellowish. A5 tyloid: carina-like, not expanded. A11: longer than broad.

Interantennal process: not elongate. Median longitudinal elevation in frontal depression: absent. Frontal depression: concave. Frontal depression sculpture: with 3-5 complete transverse carinae. Submedian carina: strong, formed by a sharp raised carina. Submedian carina medially: without peak. Median tooth of frontal depression: absent. Concavity across dorsal part of frontal depression: absent. Depression extending ventrally from median ocellus: absent. Upper frons: not hood-like. Malar area near antennal foramen: without carina or expansion. Malar area at mouth corner: with radiating striae. Smooth strip along posterior side of malar sulcus: absent or not consistently broad. Middle genal carina: present. Direction of middle genal carina dorsally: parallel to eye margin. Major sculpture of gena anteriorly: umbilicate-foveate. Major sculpture of gena posteriorly: umbilicate-foveate; rugose. Microsculpture of gena antero-ventrally: absent. Microsculpture of gena postero-ventrally: absent. Median carina extending posteriorly from hyperoccipital carina: absent. Hyperoccipital carina: indicated by rugae. Lateral connection between hyperoccipital and occipital carinae: absent. Area between vertex and occipital carina: umbilicate-foveate; irregularly rugose. Occipital carina medially: uniformly rounded. Lateral corners of occipital carina: not protruding.

Lateral pronotal area: without bulge projecting towards anterior pit. Epomial corner: strong. Netrion surface anteriorly: not inflexed. Mesoscutum anteriorly: not steep. Mesoscutal median carina: present and complete. Longitudinal carina between median carina and notauli: absent. Major sculpture of medial mesoscutum anteriorly: umbilicate-foveate; irregularly rugose. Major sculpture of medial mesoscutum posteriorly: umbilicate-punctate. Microsculpture of medial mesoscutum anteriorly: granulate. Microsculpture of medial mesoscutum posteriorly: granulate. Major sculpture of mesoscutellum: umbilicate-foveate. Microsculpture of mesoscutellum medially: granulate. Microsculpture of mesoscutellum laterally: granulate. Mesoscutellar apex: convex or straight. Setae along anterior limit of femoral depression: arising from rows of foveae. Number of carinae crossing speculum above femoral depression: 2. Number of carinae crossing femoral depression: 3-5; more than 5. Mesepimeral sulcus pits: more than 5. Metascutellum dorsally: concave. Metascutellar sculpture dorsally: smooth or with transverse carinae. Median carina of metascutellum: absent or branched. Metascutellar setae: absent. Metascutellar apex: convex or straight. Metapleuron above ventral metapleural area: crossed by carinae; smooth. Metasomal depression setae: absent. Anterior areoles of metasomal depression: absent. Anterior longitudinal carinae in metasomal depression: absent. Lateral propodeal areas: separated medially. Postmarginal vein: present.

Median lobe of T1: with 4 longitudinal carinae. Metasomal apex: with acuminate lateral corners.

#### Diagnosis.

Male: A11 longer than broad. Mesoscutum and mesoscutellum granulate. Metascutellum concave dorsally, smooth medially. Postmarginal vein present. T1 midlobe with 4 longitudinal carinae. T7 with short, sharp and protruding posterolateral corners.

#### Link to distribution map.

[http://hol.osu.edu/map-full.html?id=5008]

#### Material examined.

Neotype, male: **PHILIPPINES**: Laguna Prov., Mount Makiling (Maquiling), no date, Baker, OSUC 268270 (deposited in USNM). *Other material*: **PHILIPPINES**: 2 males, OSUC 436884 (BMNH); OSUC 268251 (USNM).

#### Comments.

The type material of *Camptoteleia brevinervis* Kieffer, collected from Mindanao (Butuan) in the Philippines,could not be found after an extensive search of collections known to house Kieffer type material. The neotype of *Camptoteleia brevinervis* is presently designated to clarify the taxonomic status of the species. It was selected because of its collection locality, its short stigmal vein relative to the postmarginal vein, and for its long flagellomeres.

### Oxyscelio caesitas

Burks sp. n.

urn:lsid:zoobank.org:act:BD154B1E-B716-43DE-9FAA-05E937EAD38D

urn:lsid:biosci.ohio-state.edu:osuc_concepts:305770

http://species-id.net/wiki/Oxyscelio_caesitas

**Figures 64–69.**
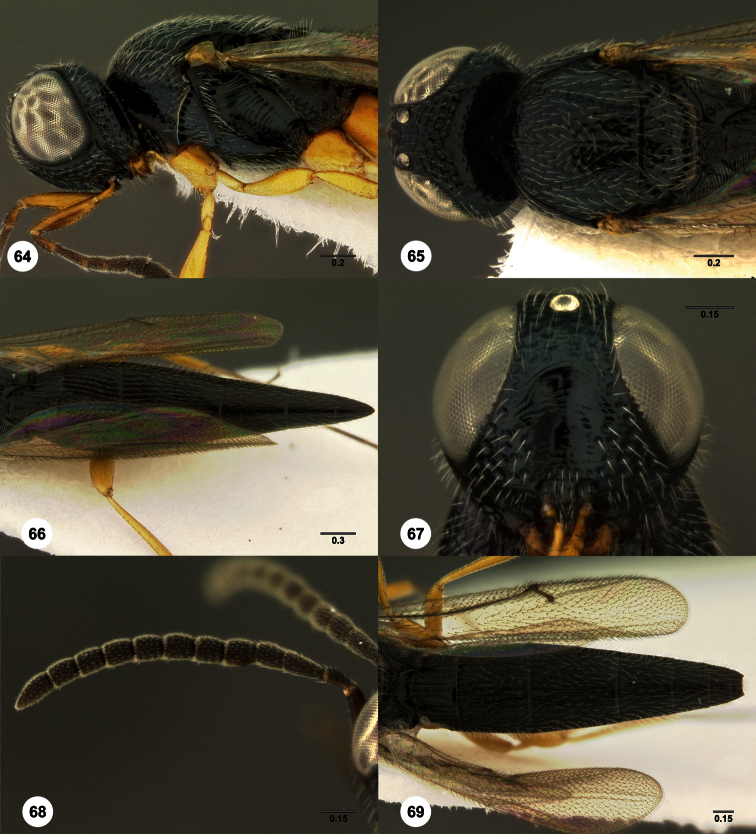
*Oxyscelio caesitas* sp. n., holotype female (ANIC Database no. 32 021000) **64** Head and mesosoma, lateral view **65** Head and mesosoma, dorsal view **66** Metasoma, dorsal view.Paratype female (OSUC 442266) **67** Head, anterior view. Paratype male (OSUC 442269) **68** Antenna **69** Metasoma, dorsal view. Morphbank^37^

Figures 64–69 Morphbank^37^

#### Description.

*Female*. Body length 4.6–4.85 mm (n=2).

Radicle color: same color as scape. Scape color: Yellowish. A4: longer than broad. A5: longer than broad; as long as broad. Antennal club: formed, segments compact.

Interantennal process: not elongate. Median longitudinal elevation in frontal depression: absent. Frontal depression: concave. Frontal depression sculpture: with 3 or more broadly interrupted transverse carinae. Submedian carina: weak, shallow and rounded or formed by ledge. Submedian carina medially: without peak. Concavity across dorsal part of frontal depression: absent. Depression extending ventrally from median ocellus: absent. Upper frons: not hood-like. Malar area near antennal foramen: without carina or expansion. Malar area at mouth corner: with radiating striae. Smooth strip along posterior side of malar sulcus: present, broad throughout its length. Middle genal carina: absent. Direction of middle genal carina dorsally: absent (replace with question mark). Major sculpture of gena anteriorly: umbilicate-foveate. Major sculpture of gena posteriorly: absent; umbilicate-foveate. Microsculpture of gena anteroventrally: absent. Microsculpture of gena posteroventrally: absent. Median carina extending posteriorly from hyperoccipital carina: absent. Hyperoccipital carina: indicated by rugae. Lateral connection between hyperoccipital and occipital carinae: absent. Area between vertex and occipital carina: umbilicate-foveate. Occipital carina medially: absent. Lateral corners of occipital carina: sharp and protruding.

Lateral pronotal area: without bulge projecting towards anterior pit. Epomial corner: weak. Netrion surface anteriorly: not inflexed. Mesoscutum anteriorly: not steep. Mesoscutal median carina: present and complete. Longitudinal carina between median carina and notauli: absent. Major sculpture of medial mesoscutum anteriorly: umbilicate-foveate. Major sculpture of medial mesoscutum posteriorly: umbilicate-foveate. Microsculpture of medial mesoscutum anteriorly: granulate. Microsculpture of medial mesoscutum posteriorly: absent. Major sculpture of mesoscutellum: umbilicate-foveate. Microsculpture of mesoscutellum medially: absent. Microsculpture of mesoscutellum laterally: absent. Mesoscutellar apex: roundly concave. Setae along anterior limit of femoral depression: arising from tiny pits. Number of carinae crossing speculum above femoral depression: 4. Number of carinae crossing femoral depression: more than 5. Mesepimeral sulcus pits: more than 5. Metascutellum dorsally: flat. Metascutellar sculpture dorsally: with scattered rugae. Median carina of metascutellum: absent or branched; straight, unbranched carina present. Metascutellar setae: absent. Metascutellar apex: weakly emarginate. Metapleuron above ventral metapleural area: crossed by carinae. Metasomal depression setae: absent. Lateral propodeal carinae anteromedially: strongly diverging. Anterior areoles of metasomal depression: absent. Anterior longitudinal carinae in metasomal depression: absent. Lateral propodeal areas: separated medially. Postmarginal vein: present. Fore wing apex: reaching middle of T4; reaching apex of T4.

T1 midlobe: obscured by other raised sculpture. T1: with long anterior bulge, reaching metascutellum. T2: with strong set of curved striae. T6: longer than broad. Apical flange of T6: not exposed apically. Metasomal apex: rounded. Major sculpture of T6: umbilicate-punctate. Microsculpture of T6: granulate.

*Male*. Body length 4.3–4.35 mm (n=3). A5 tyloid: carina-like, not expanded. A11: longer than broad. Median tooth of frontal depression: absent. Median lobe of T1: with 5 longitudinal carinae. Metasomal apex: with acuminate lateral corners.

#### Diagnosis.

Both sexes: Mesoscutellum without granulate areas. Metascutellum nearly square, rugose. Female: T1 with a strong anterior horn. T2 and T3 with long, approximated curved striate that for much of their length are not separated by setal pits. Fore wings long enough to reach middle or nearly to apex of T4. Male: A11 longer than broad. T1 midlobe with 6 or more longitudinal carinae. T7 with sharp, protruding posterolateral corners.

#### Etymology.

Latin noun in apposition, meaning “blueness.”

#### Link to distribution map.

[http://hol.osu.edu/map-full.html?id=305770]

**Material Examined**. Holotype, female: **CHRISTMAS ISLAND**: East-West Park Track, 10°30'S, 105°35'E, 13.IV–28.IV.1989, malaise trap, J. C. Cardale, ANIC DB 32-021000 (deposited in ANIC). *Paratypes*: **CHRISTMAS ISLAND**: 1 female, 3 males, OSUC 442266-442269 (ANIC).

#### Comments.

*Oxyscelio caesitas* is the only known species of *Oxyscelio* with any metallic blue luster. It is also the only member of the *striarum*-group in which males are definitively known. These male specimens do not exhibit the distinctive curved T2 and T3 striae found in females.

### Oxyscelio capilli

Burks sp. n.

urn:lsid:zoobank.org:act:7461903F-08CD-43DE-8DD4-988FD7A6DABE

urn:lsid:biosci.ohio-state.edu:osuc_concepts:305707

http://species-id.net/wiki/Oxyscelio_capilli

**Figures 70–75.**
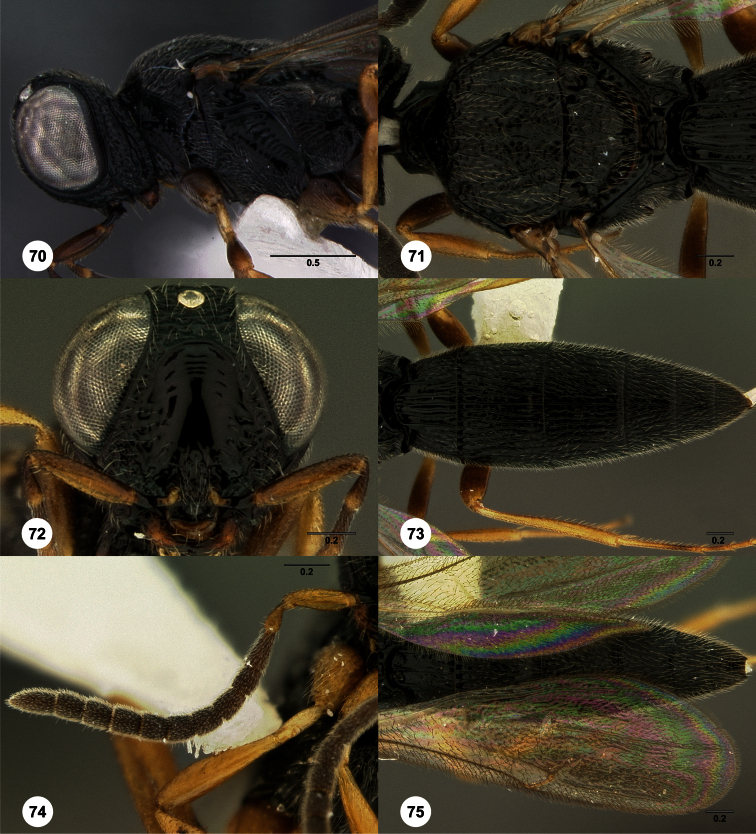
*Oxyscelio capilli* sp. n., paratype female (OSUC 368951) **70** Head and mesosoma, lateral view **71** Mesosoma, dorsal view **72** Head, anterior view **73** Metasoma, dorsal view. Paratype male (OSUC 58670) **74** Antenna **75** Metasoma, dorsal view. Morphbank^38^

Figures 70–75 Morphbank^38^

#### Description.

*Female*. Body length 3.6–4.95 mm (n=20).

Radicle color: darker than scape. Scape color: Yellowish; Brown. A4: broader than long. A5: broader than long. Antennal club: formed, segments compact.

Interantennal process: not elongate. Median longitudinal elevation in frontal depression: absent. Frontal depression: concave. Frontal depression sculpture: with 2 complete transverse carinae. Submedian carina: strong, formed by a sharp raised carina. Submedian carina medially: without peak. Concavity across dorsal part of frontal depression: absent. Depression extending ventrally from median ocellus: absent. Upper frons: not hood-like. Malar area near antennal foramen: without carina or expansion. Malar area at mouth corner: with radiating striae. Smooth strip along posterior side of malar sulcus: absent or not consistently broad. Middle genal carina: present. Direction of middle genal carina dorsally: parallel to eye margin. Major sculpture of gena anteriorly: umbilicate-foveate. Major sculpture of gena posteriorly: umbilicate-foveate; rugose. Microsculpture of gena anteroventrally: absent. Microsculpture of gena posteroventrally: absent. Median carina extending posteriorly from hyperoccipital carina: absent. Hyperoccipital carina: indicated by rugae. Lateral connection between hyperoccipital and occipital carinae: absent. Area between vertex and occipital carina: umbilicate-foveate. Occipital carina medially: uniformly rounded. Lateral corners of occipital carina: not protruding.

Lateral pronotal area: without bulge projecting towards anterior pit. Epomial corner: strong. Netrion surface anteriorly: not inflexed. Mesoscutum anteriorly: not steep. Mesoscutal median carina: present and complete. Longitudinal carina between median carina and notauli: present. Major sculpture of medial mesoscutum anteriorly: umbilicate-foveate; irregularly rugose. Major sculpture of medial mesoscutum posteriorly: umbilicate-foveate; umbilicate-punctate. Microsculpture of medial mesoscutum anteriorly: granulate. Microsculpture of medial mesoscutum posteriorly: absent. Major sculpture of mesoscutellum: umbilicate-foveate; irregularly rugose. Microsculpture of mesoscutellum medially: absent. Microsculpture of mesoscutellum laterally: absent. Mesoscutellar apex: convex or straight. Setae along anterior limit of femoral depression: arising from tiny pits. Number of carinae crossing speculum above femoral depression: 2. Number of carinae crossing femoral depression: 3-5. Mesepimeral sulcus pits: more than 5. Metascutellum dorsally: concave. Metascutellar sculpture dorsally: smooth or with transverse carinae. Median carina of metascutellum: absent or branched. Metascutellar setae: absent. Metascutellar apex: weakly emarginate. Metapleuron above ventral metapleural area: smooth. Metasomal depression setae: absent. Lateral propodeal carinae anteromedially: strongly diverging. Anterior areoles of metasomal depression: absent. Anterior longitudinal carinae in metasomal depression: absent. Lateral propodeal areas: separated medially. Postmarginal vein: present. Fore wing apex: reaching middle of T6.

T1 midlobe: with 5 longitudinal carinae. T1: without anterior bulge. T2: with straight longitudinal striae or rugae. T6: broader than long. Apical flange of T6: exposed apically. Metasomal apex: rounded. Major sculpture of T6: umbilicate-punctate. Microsculpture of T6: absent.

*Male*. Body length 3.5–4.6 mm (n=20). A5 tyloid: carina-like, not expanded. A11: longer than broad; as long as broad. Median tooth of frontal depression: absent. Median lobe of T1: with 3 longitudinal carinae. Metasomal apex: with acuminate lateral corners.

#### Diagnosis.

Both sexes: Middle genal carina subparallel with eye margin. Hyperoccipital carina indicated by rugae. Mesoscutellum without granulate sculpture. Metascutellum concave dorsally, smooth aside from some transverse carinae. Female: A5 broader than long. T1 midlobe with 5 longitudinal carinae. T6 rounded apically. Mesopleuron, along ventral margin of femoral depression, with many fine setae arising from tiny pits, some of these setae arising from the femoral depression itself. Male: A11 longer than broad. T1 midlobe with 3 longitudinal carinae. T7 with short, sharp and protruding posterolateral corners that are very widely separated. *Oxyscelio capilli* is very similar to *Oxyscelio crebritas*, and males of these two species (plus some others) are very difficult to separate due to variation in what constitute reliable diagnostic features for other species. Females of *Oxyscelio capilli* can be recognized by the extensive setation along the ventral edge of the femoral depression, which also occurs in *Oxyscelio reflectens* and some other species of *Oxyscelio*.

#### Etymology.

Latin noun, genitive case, meaning “hair.” Refers to the unusually extensive setation of the mesopleuron ventrally.

#### Link to distribution map.

[http://hol.osu.edu/map-full.html?id=305707]

#### Material examined.

Holotype, female: **INDONESIA**: Sulawesi Utara Prov., Toraut, Bogani Nani Wartabone (Dumoga-Bone) National Park, 1000m, 9.V-16.V.1985, J. S. Noyes, OSUC 369179 (deposited in BMNH). *Paratypes*:**INDONESIA**: 82 females, 26 males, OSUC 368916-368922, 368948-368953, 368959, 368962, 369180-369181, 369183-369195, 369197-369209, 369212-369216, 369220, 369222-369224, 369226, 369229-369232, 369235, 369237-369239, 369242, 369245, 369247-369250, 369252, 369255-369256, 369263-369264, 369267-369269, 369273, 369277, 369282, 369295, 369299, 369301, 369303-369305 (CNCI); OSUC 436897-436901, 436903, 58669-58670 (OSUC); OSUC 436885-436890, 436892-436896, 436902, 436904-436907 (WINC).

#### Comments.

There are two distinct size fractions of specimens included in this species, but these variants exhibit no other apparent differences.

### Oxyscelio capitis

Burks sp. n.

urn:lsid:zoobank.org:act:5579A896-20DA-49F9-A652-6835FFBCC177

urn:lsid:biosci.ohio-state.edu:osuc_concepts:275566

http://species-id.net/wiki/Oxyscelio_capitis

**Figures 76–81.**
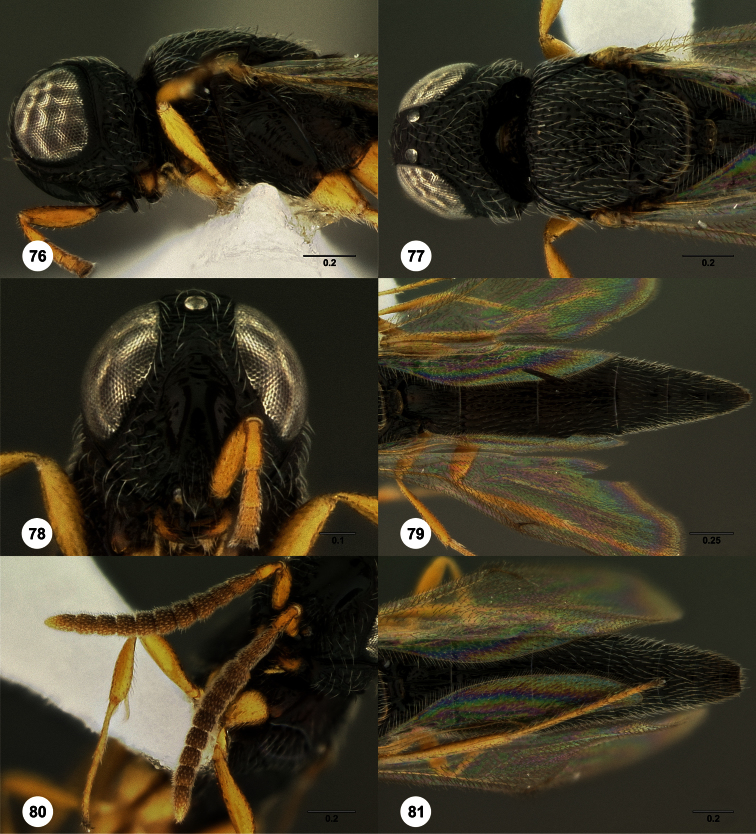
*Oxyscelio capitis* sp. n., holotype female (OSUC 335910) **76** Head and mesosoma, lateral view **77** Head and mesosoma, dorsal view **78** Head, anterior view. Paratype female (OSUC 335922) **79 **Metasoma, dorsal view. Paratype male (OSUC 247958) **80** Antenna **81** Metasoma, dorsal view. Morphbank^39^

Figures 76–81 Morphbank^39^

#### Description.

*Female*. Body length 3.05–3.25 mm (n=3).

Radicle color: darker than scape. Scape color: Yellowish. A4: broader than long. A5: broader than long. Antennal club: formed, segments compact.

Interantennal process: not elongate. Median longitudinal elevation in frontal depression: absent. Frontal depression: concave. Frontal depression sculpture: with 2 complete transverse carinae. Submedian carina: strong, formed by a sharp raised carina. Submedian carina medially: without peak. Concavity across dorsal part of frontal depression: absent. Depression extending ventrally from median ocellus: absent. Upper frons: not hood-like. Malar area near antennal foramen: without carina or expansion. Malar area at mouth corner: without striae. Smooth strip along posterior side of malar sulcus: absent or not consistently broad. Middle genal carina: present. Direction of middle genal carina dorsally: curving towards genal carina dorsally. Major sculpture of gena anteriorly: umbilicate-foveate. Major sculpture of gena posteriorly: umbilicate-foveate. Microsculpture of gena anteroventrally: absent. Microsculpture of gena posteroventrally: absent. Median carina extending posteriorly from hyperoccipital carina: absent. Hyperoccipital carina: indicated by rugae. Lateral connection between hyperoccipital and occipital carinae: absent. Area between vertex and occipital carina: umbilicate-foveate. Occipital carina medially: uniformly rounded. Lateral corners of occipital carina: not protruding.

Lateral pronotal area: with slight bulge projecting anteriorly towards anterior pit. Epomial corner: strong. Netrion surface anteriorly: not inflexed. Mesoscutum anteriorly: not steep. Mesoscutal median carina: present and complete. Longitudinal carina between median carina and notauli: present. Major sculpture of medial mesoscutum anteriorly: umbilicate-foveate. Major sculpture of medial mesoscutum posteriorly: umbilicate-foveate; umbilicate-punctate. Microsculpture of medial mesoscutum anteriorly: granulate. Microsculpture of medial mesoscutum posteriorly: absent. Major sculpture of mesoscutellum: umbilicate-foveate; longitudinally rugose. Microsculpture of mesoscutellum medially: absent. Microsculpture of mesoscutellum laterally: absent. Mesoscutellar apex: convex or straight. Setae along anterior limit of femoral depression: arising from rows of foveae. Number of carinae crossing speculum above femoral depression: 2. Number of carinae crossing femoral depression: more than 5. Mesepimeral sulcus pits: more than 5. Metascutellum dorsally: flat. Metascutellar sculpture dorsally: with scattered rugae. Median carina of metascutellum: absent or branched. Metascutellar setae: absent. Metascutellar apex: convex or straight. Metapleuron above ventral metapleural area: crossed by carinae. Metasomal depression setae: absent. Lateral propodeal carinae anteromedially: strongly diverging. Anterior areoles of metasomal depression: absent. Anterior longitudinal carinae in metasomal depression: absent. Lateral propodeal areas: separated medially. Postmarginal vein: present. Fore wing apex: reaching apex of T5.

T1 midlobe: obscured by other raised sculpture. T1: with long anterior bulge, reaching metascutellum. T2: with straight longitudinal striae or rugae. T6: longer than broad; as long as broad. Apical flange of T6: exposed apically. Metasomal apex: rounded. Major sculpture of T6: umbilicate-punctate; longitudinally striate or rugose. Microsculpture of T6: absent.

*Male*.Body length 2.9–3.05 mm (n=2). A5 tyloid: expanded, teardrop-shaped or sinuate. A11: broader than long. Median tooth of frontal depression: absent. Median lobe of T1: with 4 longitudinal carinae. Metasomal apex: with acuminate lateral corners.

#### Diagnosis.

Both sexes: Middle genal carina angled towards genal carina dorsally. Metascutellum flat but with one or more transverse carinae. Female: A4, A5 broader than long. T1 midlobe with well-developed anterior horn. Male: A11 broader than long. A5 tyloid expanded, sinuate or teardrop-shaped. T7 with short, sharp and protruding posterolateral corners. *Oxyscelio capitis* is very similar to *Oxyscelio reflectens*, but is smaller-bodied, with a relatively larger head, a flat metascutellum, a T1 horn in females, and acuminate posterolateral corners on T7 in males.

#### Etymology.

Latin noun, genitive case, meaning “head.” Emphasizes the large head of this species.

#### Link to distribution map.

[http://hol.osu.edu/map-full.html?id=275566]

#### Material examined.

Holotype, female: **THAILAND**: Kanchanaburi Prov., Khong Kraborg, T3431, Khuean Srinagarindra National Park, 14°29.972'N, 98°53.035'E, 210m, 4.IX–11.IX.2008, malaise trap, Boonnam & Phumarin, OSUC 335910 (deposited in QSBG). *Paratypes*: **THAILAND**: 2 females, 2 males, OSUC 247918, 335919 (OSUC); OSUC 247958, 335922 (QSBG).

### Oxyscelio carinatus

(Kieffer)

urn:lsid:zoobank.org:act:C91A9F90-F2C4-4391-ABB9-DCBE67993121

urn:lsid:biosci.ohio-state.edu:osuc_concepts:5009

http://species-id.net/wiki/Oxyscelio_carinatus

**Figures 82–87.**
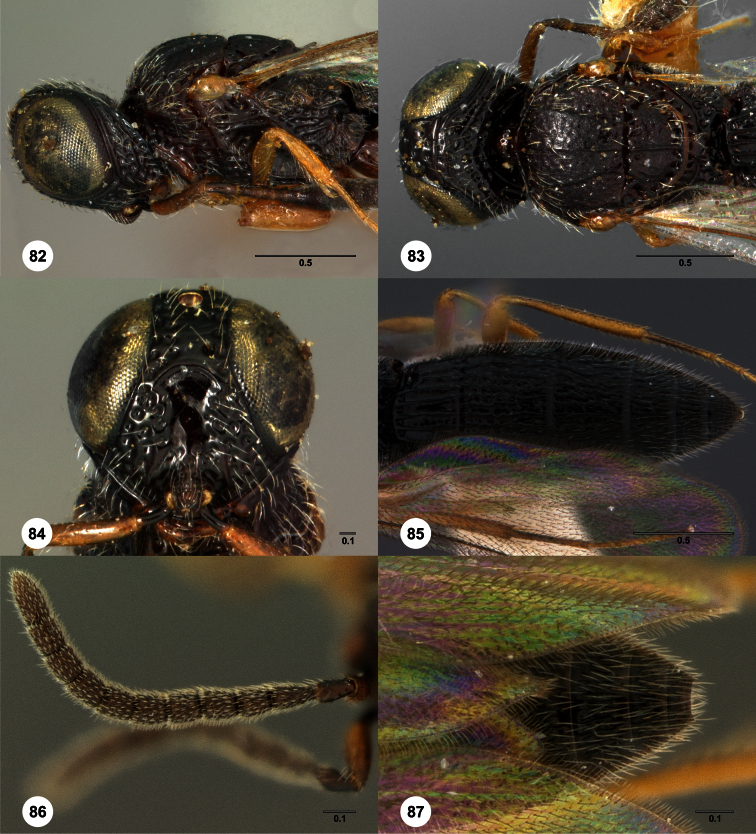
*Oxyscelio carinatus* (Kieffer), holotype female (USNM Type No. 70472) **82** Head and mesosoma, lateral view **83** Head and mesosoma, dorsal view **84** Head, anterior view. Female (OSUC 369057) **85** Metasoma, dorsal view. Male (ROMEnt Spec. No. 112220) **86** Antenna **87** Metasoma, dorsal view. Morphbank^40^

Figures 82–87 Morphbank^40^

Camptoteleia carinata Kieffer, 1913b: 387 (original description, keyed); Kieffer 1914: 296 (keyed); Kieffer 1916: 171 (keyed); Kieffer 1926: 380 (description, keyed).
Oxyscelio carinatus (Kieffer): Dodd 1931: 74 (generic transfer); Masner 1976: 23 (type information).
Camptoteleia kiefferi Benoit: Kelner-Pillault 1958: 150 (unnecessarily proposed replacement name, rejected by Baltazar (1966)).

#### Description.

*Female*. Body length 6.3–7.1 mm (n=8).

Radicle color: darker than scape. Scape color: Brown. A4: broader than long; as long as broad. A5: broader than long. Antennal club: formed, segments compact.

Interantennal process: not elongate. Median longitudinal elevation in frontal depression: absent. Frontal depression: flat. Frontal depression sculpture: without transverse or oblique carinae below submedian carina. Submedian carina: weak, shallow and rounded or formed by ledge. Submedian carina medially: without peak. Concavity across dorsal part of frontal depression: absent. Depression extending ventrally from median ocellus: absent. Upper frons: not hood-like. Malar area near antennal foramen: without carina or expansion. Malar area at mouth corner: without striae; with radiating striae. Smooth strip along posterior side of malar sulcus: absent or not consistently broad. Middle genal carina: absent. Direction of middle genal carina dorsally: absent (replace with question mark). Major sculpture of gena anteriorly: umbilicate-foveate. Major sculpture of gena posteriorly: umbilicate-foveate. Microsculpture of gena anteroventrally: absent. Microsculpture of gena posteroventrally: absent. Median carina extending posteriorly from hyperoccipital carina: absent. Hyperoccipital carina: indicated by rugae. Lateral connection between hyperoccipital and occipital carinae: absent. Area between vertex and occipital carina: umbilicate-foveate. Occipital carina medially: absent. Lateral corners of occipital carina: not protruding.

Lateral pronotal area: without bulge projecting towards anterior pit. Epomial corner: strong. Netrion surface anteriorly: not inflexed. Mesoscutum anteriorly: steep. Mesoscutal median carina: present and complete. Longitudinal carina between median carina and notauli: absent. Major sculpture of medial mesoscutum anteriorly: umbilicate-foveate. Major sculpture of medial mesoscutum posteriorly: umbilicate-foveate. Microsculpture of medial mesoscutum anteriorly: absent. Microsculpture of medial mesoscutum posteriorly: absent. Major sculpture of mesoscutellum: obliquely rugose. Microsculpture of mesoscutellum medially: absent. Microsculpture of mesoscutellum laterally: absent. Mesoscutellar apex: convex or straight. Setae along anterior limit of femoral depression: arising from rows of foveae. Number of carinae crossing speculum above femoral depression: 2; 3. Number of carinae crossing femoral depression: 3-5. Mesepimeral sulcus pits: more than 5. Metascutellum dorsally: concave. Metascutellar sculpture dorsally: smooth or with transverse carinae. Median carina of metascutellum: absent or branched. Metascutellar setae: with many dorsal setae. Metascutellar apex: deeply emarginate. Metapleuron above ventral metapleural area: crossed by carinae. Metasomal depression setae: absent. Lateral propodeal carinae anteromedially: strongly diverging. Anterior areoles of metasomal depression: one or more areoles present. Anterior longitudinal carinae in metasomal depression: median carina present. Lateral propodeal areas: separated medially. Postmarginal vein: present. Fore wing apex: reaching apex of T6; reaching beyond T6.

T1 midlobe: with 5 longitudinal carinae. T1: without anterior bulge. T2: irregularly areolate. T6: broader than long. Apical flange of T6: exposed apically. Metasomal apex: rounded. Major sculpture of T6: umbilicate-punctate. Microsculpture of T6: absent.

*Male*. Body length 3.15–3.25 mm (n=3). A5 tyloid: expanded, teardrop-shaped or sinuate. A11: longer than broad. Median tooth of frontal depression: absent. Median lobe of T1: with 4 longitudinal carinae. Metasomal apex: with acuminate lateral corners.

#### Diagnosis.

Both sexes: Frons without elevation between antennal foramen and eye; frontal depression flat. Hyperoccipital carina defined by ruga, but continuous with anterior genal carina. Metascutellum with dorsal setae. Metasomal depression extensively sculptured; lateral propodeal carinae broadly separated anteriorly. Female: A4, A5 broader than long. T1 midlobe with 5 longitudinal carinae. Male: T1 midlobe with 4 longitudinal carinae. T7 with sharp posterolateral corners. *Oxyscelio carinatus* is very similar to *Oxyscelio spinosiceps*, but differs in having weaker mesoscutal and scutellar sculpture, and in lacking a flange between the antennal foramen and eye.

#### Link to distribution map.

[http://hol.osu.edu/map-full.html?id=5009]

#### Material examined.

Holotype, female, *Camptoteleia carinata*: **PHILIPPINES**: Laguna Prov., Los Baños, no date, Baker, USNM Type No. 70472 (deposited in USNM). *Other material*: **PHILIPPINES**: 8 females, 3 males, OSUC 149521 (AEIC); OSUC 369057 (CNCI); OSUC 240936, ROMEnt Spec. No. 112206, ROMEnt Spec. No. 112212, ROMEnt Spec. No. 112218, ROMEnt Spec. No. 112220, ROMEnt Spec. No. 112225, ROMEnt Spec. No. 112684, ROMEnt Spec. No. 112685 (ROME); OSUC 268272 (USNM).

### Oxyscelio cavinetrion

Burks sp. n.

urn:lsid:zoobank.org:act:9C701DA3-7561-45CB-AA2F-AF66CE34B854

urn:lsid:biosci.ohio-state.edu:osuc_concepts:275527

http://species-id.net/wiki/Oxyscelio_cavinetrion

**Figures 88–93.**
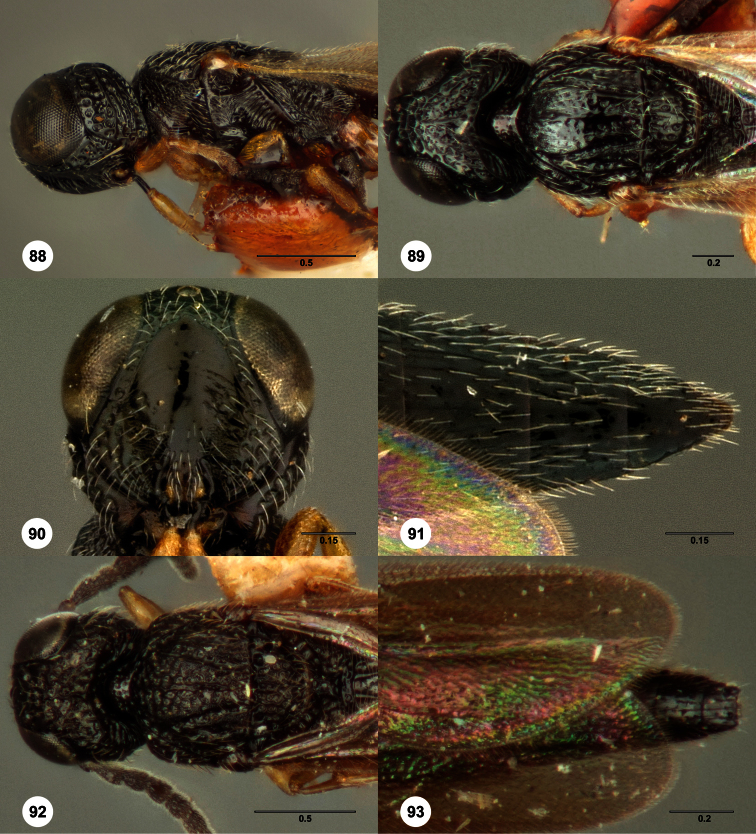
*Oxyscelio cavinetrion* sp. n., paratype female (OSUC 268262) **88** Head and mesosoma, lateral view **89** Head and mesosoma, dorsal view. Holotype female (OSUC 268260) **90** Head, anterior view **91** Metasoma, dorsal view. Paratype male (OSUC 268271) **92** Mesosoma, dorsal view **93** Metasomal apex, dorsal view. Morphbank^41^

Figures 88–93 Morphbank^41^

#### Description.

*Female*. Body length 3.5 mm (n=1).

Radicle color: darker than scape. Scape color: Yellowish. A4: longer than broad. A5: broader than long. Antennal club: formed, segments compact.

Interantennal process: not elongate. Median longitudinal elevation in frontal depression: absent. Frontal depression: concave. Frontal depression sculpture: with 3-5 complete transverse carinae. Submedian carina: weak, shallow and rounded or formed by ledge. Submedian carina medially: with sharp peak. Concavity across dorsal part of frontal depression: absent. Depression extending ventrally from median ocellus: absent. Upper frons: not hood-like. Malar area near antennal foramen: without carina or expansion. Malar area at mouth corner: without striae. Smooth strip along posterior side of malar sulcus: present, broad throughout its length. Middle genal carina: present. Direction of middle genal carina dorsally: parallel to eye margin. Major sculpture of gena anteriorly: umbilicate-foveate. Major sculpture of gena posteriorly: rugose. Microsculpture of gena anteroventrally: absent. Microsculpture of gena posteroventrally: absent. Median carina extending posteriorly from hyperoccipital carina: present. Hyperoccipital carina: not indicated medially. Lateral connection between hyperoccipital and occipital carinae: absent. Area between vertex and occipital carina: with transverse carinae. Occipital carina medially: divided into concave halves, meeting at median peak. Lateral corners of occipital carina: sharp and protruding.

Lateral pronotal area: without bulge projecting towards anterior pit. Epomial corner: strong. Netrion surface anteriorly: inflexed. Mesoscutum anteriorly: not steep. Mesoscutal median carina: present and complete. Longitudinal carina between median carina and notauli: absent. Major sculpture of medial mesoscutum anteriorly: umbilicate-punctate. Major sculpture of medial mesoscutum posteriorly: umbilicate-foveate. Microsculpture of medial mesoscutum anteriorly: granulate. Microsculpture of medial mesoscutum posteriorly: absent. Major sculpture of mesoscutellum: umbilicate-foveate; longitudinally rugose. Microsculpture of mesoscutellum medially: absent. Microsculpture of mesoscutellum laterally: absent. Mesoscutellar apex: convex or straight. Setae along anterior limit of femoral depression: arising from rows of foveae. Number of carinae crossing speculum above femoral depression: 2. Number of carinae crossing femoral depression: more than 5. Mesepimeral sulcus pits: more than 5. Metascutellum dorsally: concave. Metascutellar sculpture dorsally: smooth or with transverse carinae. Median carina of metascutellum: absent or branched. Metascutellar setae: absent. Metascutellar apex: weakly emarginate. Metapleuron above ventral metapleural area: crossed by carinae. Metasomal depression setae: absent. Lateral propodeal carinae anteromedially: weakly diverging. Anterior areoles of metasomal depression: one or more areoles present. Anterior longitudinal carinae in metasomal depression: absent. Lateral propodeal areas: separated medially. Postmarginal vein: present. Fore wing apex: reaching middle of T5; reaching apex of T5.

T1 midlobe: with 4 longitudinal carinae. T1: without anterior bulge. T2: with straight longitudinal striae or rugae. T6: broader than long. Apical flange of T6: not exposed apically. Metasomal apex: rounded. Major sculpture of T6: umbilicate-punctate. Microsculpture of T6: granulate.

*Male*. Body length 3.3–3.55 mm (n=8). A5 tyloid: carina-like, not expanded. A11: longer than broad. Median tooth of frontal depression: absent. Median lobe of T1: with 4 longitudinal carinae. Metasomal apex: with acuminate lateral corners.

#### Diagnosis.

Both sexes: Occipital carina complete as a distinct carina, but medial portions concave and meeting at a peak. Mesoscutellum with a few flattened longitudinal carinae. Netrion concave anteriorly. Metascutellum tiny, dorsally concave. Female: A4, A5 broader than long. Fore wings long enough to reach middle or apex of T5. T1 midlobe without anterior horn. Male: A11 longer than broad. Fore wings long enough to reach middle of T5. T7 with short, sharp and protruding posterolateral corners. *Oxyscelio cavinetrion* is very similar to *Oxyscelio flavipennis*, but has a shorter metasoma which lacks the anterior T1 horn in females, and a differently shaped metascutellum.

#### Etymology.

Compound noun intended to mean “concave netrion.”

#### Link to distribution map.

[http://hol.osu.edu/map-full.html?id=275527]

#### Material examined.

Holotype, female: **PHILIPPINES**: Basilan Prov., Basilan Island, no date, Baker, OSUC 268260 (deposited in USNM). *Paratypes*: **PHILIPPINES**: 1 female, 10 males, ROMEnt Spec. No. 112682 (ROME); OSUC 268224, 268234-268235, 268238, 268240, 268246, 268258, 268262, 268266, 268271 (USNM).

### Oxyscelio ceylonensis

(Dodd)

urn:lsid:zoobank.org:act:FA70AF9F-816F-497E-BD85-674997D2543B

urn:lsid:biosci.ohio-state.edu:osuc_concepts:5010

http://species-id.net/wiki/Oxyscelio_ceylonensis

**Figures 94–99.**
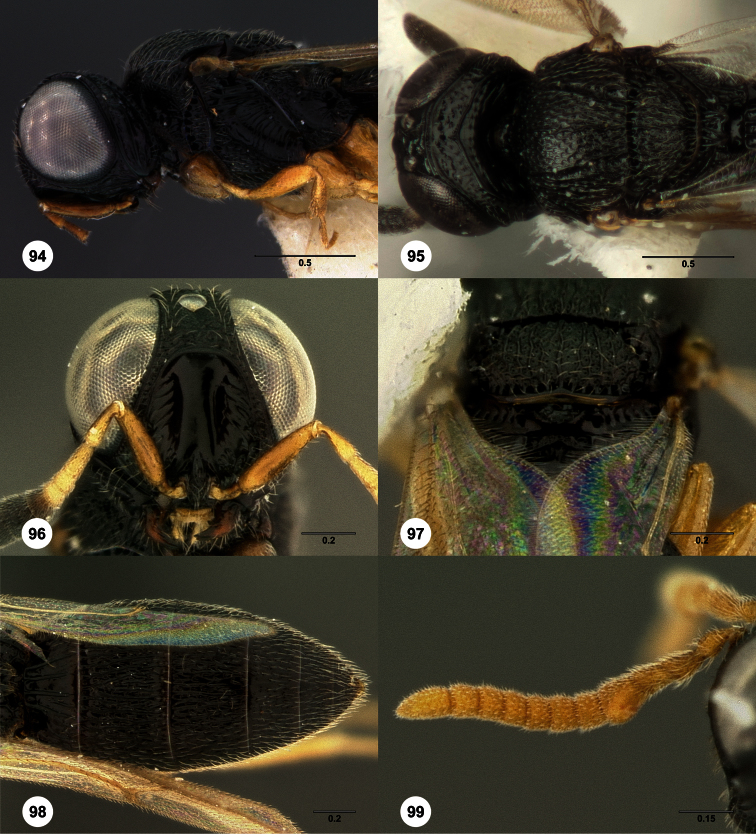
*Oxyscelio ceylonensis* (Dodd), female (OSUC 268172) **94** Head and mesosoma, lateral view **95** Head and mesosoma, dorsal view. Female (OSUC 369091) **96** Head, anterior view. Female (OSUC 268174) **97** Propodeum, posterior view. Female (OSUC 369174) **98** Metasoma, dorsal view. Male (OSUC 369134) **99** Antenna. Morphbank^42^

Figures 94–99 Morphbank^42^

Sceliomorpha ceylonensis Dodd, 1920: 349 (original description); Masner 1965: 96 (type information).
Oxyscelio ceylonensis (Dodd): Dodd 1931: 75 (generic transfer); Masner 1976: 24 (description).

#### Description.

*Female*. Body length 2.9–3.65 mm (n=20).

Radicle color: same color as scape. Scape color: Yellowish. A4: broader than long; as long as broad. A5: broader than long. Antennal club: formed, segments compact.

Interantennal process: not elongate. Median longitudinal elevation in frontal depression: absent. Frontal depression: concave. Frontal depression sculpture: with 3 or more broadly interrupted transverse carinae. Submedian carina: strong, formed by a sharp raised carina. Submedian carina medially: without peak. Concavity across dorsal part of frontal depression: absent. Depression extending ventrally from median ocellus: absent. Upper frons: not hood-like. Malar area near antennal foramen: without carina or expansion. Malar area at mouth corner: with radiating striae. Smooth strip along posterior side of malar sulcus: absent or not consistently broad. Middle genal carina: present. Direction of middle genal carina dorsally: parallel to eye margin. Major sculpture of gena anteriorly: rugose; umbilicate-punctate. Major sculpture of gena posteriorly: absent; umbilicate-punctate. Microsculpture of gena anteroventrally: absent. Microsculpture of gena posteroventrally: granulate. Median carina extending posteriorly from hyperoccipital carina: absent. Hyperoccipital carina: complete, continuous with anterior genal carina. Lateral connection between hyperoccipital and occipital carinae: absent. Area between vertex and occipital carina: with transverse carinae; irregularly rugose. Occipital carina medially: uniformly rounded; sinuate, concave medial to corners, but without a median peak. Lateral corners of occipital carina: not protruding.

Lateral pronotal area: without bulge projecting towards anterior pit. Epomial corner: strong. Netrion surface anteriorly: not inflexed. Mesoscutum anteriorly: steep. Mesoscutal median carina: present and complete. Longitudinal carina between median carina and notauli: absent. Major sculpture of medial mesoscutum anteriorly: umbilicate-foveate. Major sculpture of medial mesoscutum posteriorly: umbilicate-foveate; longitudinally rugose. Microsculpture of medial mesoscutum anteriorly: granulate. Microsculpture of medial mesoscutum posteriorly: absent. Major sculpture of mesoscutellum: umbilicate-foveate; longitudinally rugose. Microsculpture of mesoscutellum medially: granulate. Microsculpture of mesoscutellum laterally: granulate. Mesoscutellar apex: convex or straight. Setae along anterior limit of femoral depression: arising from rows of foveae. Number of carinae crossing speculum above femoral depression: 3. Number of carinae crossing femoral depression: more than 5. Mesepimeral sulcus pits: more than 5. Metascutellum dorsally: concave. Metascutellar sculpture dorsally: with scattered rugae. Median carina of metascutellum: absent or branched. Metascutellar setae: with many dorsal setae. Metascutellar apex: weakly emarginate. Metapleuron above ventral metapleural area: crossed by carinae. Metasomal depression setae: absent. Lateral propodeal carinae anteromedially: strongly diverging; weakly diverging. Anterior areoles of metasomal depression: absent; one or more areoles present. Anterior longitudinal carinae in metasomal depression: absent. Lateral propodeal areas: separated medially. Postmarginal vein: present. Fore wing apex: reaching apex of T5; reaching beyond T6; reaching middle of T6. T1 midlobe: with 4 longitudinal carinae. T1: without anterior bulge. T2: with straight longitudinal striae or rugae. T6: broader than long. Apical flange of T6: exposed apically. Metasomal apex: rounded. Major sculpture of T6: umbilicate-punctate; longitudinally striate or rugose. Microsculpture of T6: absent.

*Male*. Body length 3.1–3.55 mm (n=8). A5 tyloid: expanded, teardrop-shaped or sinuate. A11: broader than long; as long as broad. Median tooth of frontal depression: absent. Median lobe of T1: with 3 longitudinal carinae. Metasomal apex: with no distinct corners.

#### Diagnosis.

Both sexes: Frons without elevation between antennal foramen and eye. Hyperoccipital carina present, continuous with anterior genal carina. Medial mesoscutum and mesoscutellum with many strong longitudinal rugae. Metascutellum with dorsal setae. Female: A4, A5 broader than long. T1 with 4 longitudinal carinae. Male: A5 tyloid expanded. Frontal depression without tooth-like median protrusion dorsally. T1 midlobe with 3 longitudinal carinae. T7 without distinct posterolateral corners. *Oxyscelio ceylonensis* is very similar to *Oxyscelio unguis*, but differs chiefly in the longitudinal rugae mentioned here.

#### Link to distribution map.

[http://hol.osu.edu/map-full.html?id=5010]

#### Material examined.

Holotype, female, *Sceliomorpha ceylonensis*: **SRI LANKA**: 67-25, no date, Thwaites, B.M. TYPE HYM. 9.509 (deposited in BMNH). *Paratypes*: **SRI LANKA**: 3 females, OSUC 376673-376675 (BMNH). *Other material*: (33 females, 10 males) **CHINA**: 1 female, OSUC 268209 (USNM). **INDIA**: 3 females, OSUC 376565-376566, 376577 (BMNH). **MALAYSIA**: 2 females, OSUC 376746, 376751 (MCZC). **NEPAL**: 18 females, 9 males, OSUC 369129-369138, 369140-369145, 369147, 369150-369157, 369159, 369174 (CNCI). **SRI LANKA**: 5 females, ANIC DB 32-020126 (ANIC); OSUC 442262 (BMNH); OSUC 369091 (CNCI); OSUC 268172, 268174 (USNM). **THAILAND**: 1 female, OSUC 247605 (OSUC). **VIETNAM**: 3 females, 1 male, OSUC 119941 (OSUC); OSUC 277390, 277411, 281583 (RMNH).

#### Comments.

The lateral propodeal carinae exhibit strong variation in *Oxyscelio ceylonensis*, being narrowly separated and subparallel in some specimens (especially those from Nepal) and strongly divergent in others. This variation did not prove consistent enough to serve as a convincing feature for species separation.

### Oxyscelio chimaerae

Burks sp. n.

urn:lsid:zoobank.org:act:D8EA6ACB-8C3D-40BE-8B8F-2FD185B2D5B8

urn:lsid:biosci.ohio-state.edu:osuc_concepts:275551

http://species-id.net/wiki/Oxyscelio_chimaerae

**Figures 100–103.**
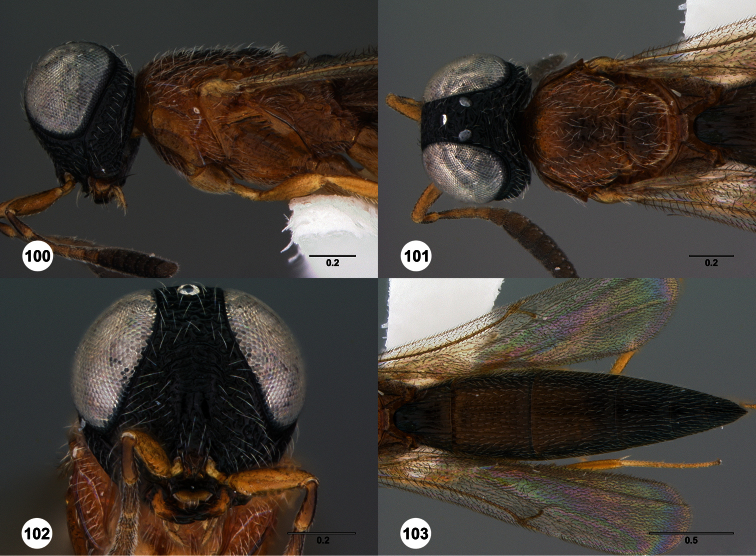
*Oxyscelio chimaerae* sp. n., holotype female (OSUC 369320) **100** Head and mesosoma, lateral view **101** Head and mesosoma, dorsal view **102** Head, anterior view **103** Metasoma, dorsal view. Morphbank^43^

Figures 100–103 Morphbank^43^

#### Description.

*Female*. Body length 3.4 mm (n=1).

Radicle color: same color as scape. Scape color: Yellowish. A4: longer than broad. A5: longer than broad. Antennal club: formed, segments compact.

Interantennal process: not elongate. Median longitudinal elevation in frontal depression: present. Frontal depression: concave. Frontal depression sculpture: crossed by many tiny furrows. Submedian carina: weak, shallow and rounded or formed by ledge. Submedian carina medially: without peak. Concavity across dorsal part of frontal depression: absent. Depression extending ventrally from median ocellus: absent. Upper frons: not hood-like. Malar area near antennal foramen: without carina or expansion. Malar area at mouth corner: with radiating striae. Smooth strip along posterior side of malar sulcus: absent or not consistently broad. Middle genal carina: present. Direction of middle genal carina dorsally: parallel to eye margin. Major sculpture of gena anteriorly: umbilicate-foveate; rugose. Major sculpture of gena posteriorly: umbilicate-foveate; rugose. Microsculpture of gena anteroventrally: absent. Microsculpture of gena posteroventrally: absent. Median carina extending posteriorly from hyperoccipital carina: absent. Hyperoccipital carina: indicated by rugae. Lateral connection between hyperoccipital and occipital carinae: absent. Area between vertex and occipital carina: umbilicate-foveate. Occipital carina medially: uniformly rounded. Lateral corners of occipital carina: not protruding.

Lateral pronotal area: without bulge projecting towards anterior pit. Epomial corner: strong. Netrion surface anteriorly: not inflexed. Mesoscutum anteriorly: not steep. Mesoscutal median carina: present and complete. Longitudinal carina between median carina and notauli: absent. Major sculpture of medial mesoscutum anteriorly: umbilicate-punctate. Major sculpture of medial mesoscutum posteriorly: umbilicate-punctate; irregularly rugose. Microsculpture of medial mesoscutum anteriorly: granulate. Microsculpture of medial mesoscutum posteriorly: absent. Major sculpture of mesoscutellum: umbilicate-foveate. Microsculpture of mesoscutellum medially: absent. Microsculpture of mesoscutellum laterally: granulate. Mesoscutellar apex: convex or straight. Setae along anterior limit of femoral depression: arising from rows of foveae. Number of carinae crossing speculum above femoral depression: 3. Number of carinae crossing femoral depression: 3-5. Mesepimeral sulcus pits: more than 5. Metascutellum dorsally: concave. Metascutellar sculpture dorsally: smooth or with transverse carinae. Median carina of metascutellum: absent or branched. Metascutellar setae: absent. Metascutellar apex: weakly emarginate. Metapleuron above ventral metapleural area: crossed by carinae. Metasomal depression setae: absent. Lateral propodeal carinae anteromedially: strongly diverging. Anterior areoles of metasomal depression: absent. Anterior longitudinal carinae in metasomal depression: absent. Lateral propodeal areas: separated medially. Postmarginal vein: present. Fore wing apex: reaching middle of T5.

T1 midlobe: obscured by other raised sculpture. T1: with small rounded anterior bulge, not reaching metascutellum. T2: with straight longitudinal striae or rugae. T6: longer than broad. Apical flange of T6: not exposed apically. Metasomal apex: rounded. Major sculpture of T6: umbilicate-punctate; longitudinally striate or rugose. Microsculpture of T6: granulate.

*Male*. Unknown.

#### Diagnosis.

Female: A4 longer than broad. Frontal depression crossed by many carinae that are interrupted medially. Submedian carina weak, not accompanied by extra carinae dorsally. Hyperoccipital carina indicated by rugae; occipital carina without distinct lateral corners. Mesoscutellum with granulate sculpture. Mesofemoral depression crossed by few (not more than 5) carinae below speculum. Metascutellum subrectangular, smooth centrally. T1 midlobe with very strong anterior horn. T2 without sublateral depressions or curved striae. Fore wings long enough to reach middle of T5. T6 slightly longer than broad.

#### Etymology.

Latinized noun, genitive case. Refers to the mix of distinctive features in this species.

#### Link to distribution map.

[http://hol.osu.edu/map-full.html?id=275551]

#### Material examined.

Holotype, female: **MALAYSIA**: Sabah St., rainforest edge, Danum Valley Protection Forest Reserve, 25.X–11.XII.1986, malaise trap, P. J. Eggleton, OSUC 369320 (deposited in BMNH).

#### Comments.

*Oxyscelio chimaerae* exhibits an unusual mix of features that agree with the *noduli*-group; it is the only known member of that group from outside Sulawesi.

### Oxyscelio codae

Burks sp. n.

urn:lsid:zoobank.org:act:BDF7E587-400A-4EC9-B711-A1EB50BE09D2

urn:lsid:biosci.ohio-state.edu:osuc_concepts:305708

http://species-id.net/wiki/Oxyscelio_codae

**Figures 104–109.**
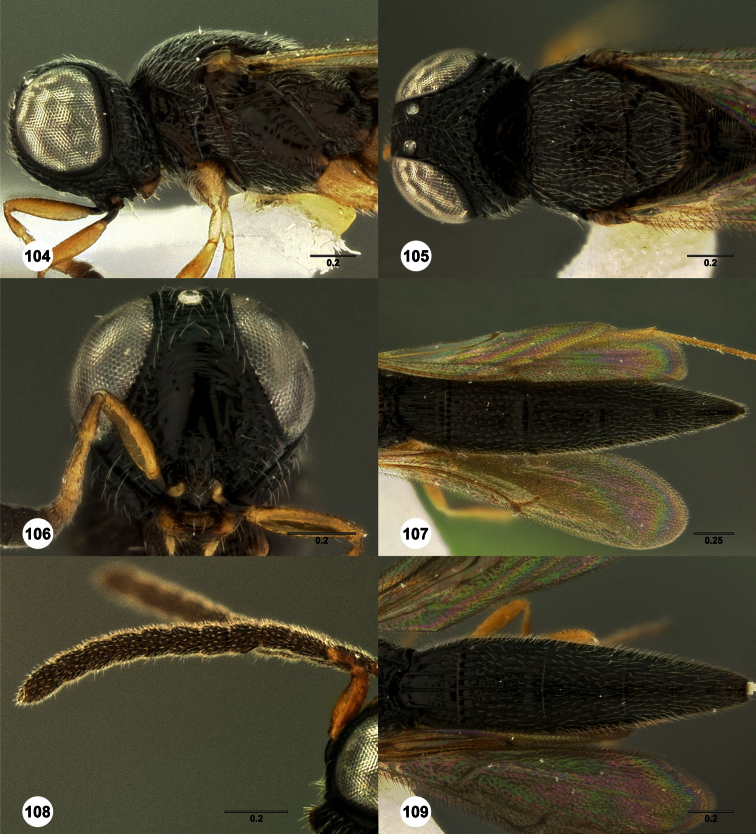
*Oxyscelio codae* sp. n., paratype female (OSUC 369292) **104** Head and mesosoma, lateral view **105** Head and mesosoma, dorsal view. Paratype female (OSUC 369286) **106** Head, anterior view. Paratype female (OSUC 369293) **107** Metasoma, dorsal view. Paratype male (OSUC 369284) **108 **Antenna. Paratype male (OSUC 369259) **109** Metasoma, dorsal view. Morphbank^44^

Figures 104–109 Morphbank^44^

#### Description.

*Female*.Body length 3.6–3.9 mm (n=20).

Radicle color: darker than scape. Scape color: Yellowish. A4: broader than long; as long as broad. A5: broader than long. Antennal club: formed, segments compact.

Interantennal process: not elongate. Median longitudinal elevation in frontal depression: absent. Frontal depression: concave. Frontal depression sculpture: with 3-5 complete transverse carinae. Submedian carina: weak, shallow and rounded or formed by ledge. Submedian carina medially: without peak. Concavity across dorsal part of frontal depression: absent. Depression extending ventrally from median ocellus: absent. Upper frons: not hood-like. Malar area near antennal foramen: without carina or expansion. Malar area at mouth corner: with radiating striae. Smooth strip along posterior side of malar sulcus: absent or not consistently broad. Middle genal carina: present. Direction of middle genal carina dorsally: parallel to eye margin. Major sculpture of gena anteriorly: umbilicate-foveate. Major sculpture of gena posteriorly: umbilicate-foveate; rugose. Microsculpture of gena anteroventrally: absent. Microsculpture of gena posteroventrally: absent. Median carina extending posteriorly from hyperoccipital carina: absent. Hyperoccipital carina: indicated by rugae. Lateral connection between hyperoccipital and occipital carinae: absent. Area between vertex and occipital carina: umbilicate-foveate. Occipital carina medially: absent. Lateral corners of occipital carina: not protruding.

Lateral pronotal area: without bulge projecting towards anterior pit. Epomial corner: strong. Netrion surface anteriorly: not inflexed. Mesoscutum anteriorly: not steep. Mesoscutal median carina: present and complete. Longitudinal carina between median carina and notauli: present. Major sculpture of medial mesoscutum anteriorly: umbilicate-foveate; irregularly rugose. Major sculpture of medial mesoscutum posteriorly: umbilicate-foveate; transversely rugose. Microsculpture of medial mesoscutum anteriorly: absent; granulate. Microsculpture of medial mesoscutum posteriorly: absent. Major sculpture of mesoscutellum: umbilicate-foveate; irregularly rugose. Microsculpture of mesoscutellum medially: absent. Microsculpture of mesoscutellum laterally: absent. Mesoscutellar apex: convex or straight. Setae along anterior limit of femoral depression: arising from rows of foveae. Number of carinae crossing speculum above femoral depression: 2. Number of carinae crossing femoral depression: 3-5. Mesepimeral sulcus pits: more than 5. Metascutellum dorsally: concave. Metascutellar sculpture dorsally: smooth or with transverse carinae. Median carina of metascutellum: absent or branched. Metascutellar setae: absent. Metascutellar apex: convex or straight. Metapleuron above ventral metapleural area: crossed by carinae. Metasomal depression setae: absent. Lateral propodeal carinae anteromedially: strongly diverging. Anterior areoles of metasomal depression: absent. Anterior longitudinal carinae in metasomal depression: absent. Lateral propodeal areas: separated medially. Postmarginal vein: present. Fore wing apex: reaching middle of T5; reaching apex of T5.

T1 midlobe: obscured by other raised sculpture. T1: with small rounded anterior bulge, not reaching metascutellum. T2: with straight longitudinal striae or rugae. T6: longer than broad. Apical flange of T6: exposed apically. Metasomal apex: rounded. Major sculpture of T6: umbilicate-punctate. Microsculpture of T6: absent.

*Male*. Body length 3.25–3.5 mm (n=6). A5 tyloid: carina-like, not expanded. A11: longer than broad; as long as broad. Median tooth of frontal depression: absent. Median lobe of T1: with 3 longitudinal carinae. Metasomal apex: with acuminate lateral corners.

#### Diagnosis.

Both sexes: Middle genal carina subparallel with eye margin. Hyperoccipital carina indicated by rugae. Mesoscutellum without granulate sculpture. Metascutellum concave dorsally, smooth aside from some transverse carinae. Female: A5 broader than long. T1 midlobe with a small anterior horn obscuring the longitudinal carinae. T6 rounded apically but longer than broad. Male: A11 longer than broad. T1 midlobe with 3 longitudinal carinae. T7 with short, sharp and protruding posterolateral corners that are curved and not widely separated. *Oxyscelio codae* is distinguished from most members of the *crebritas*-group in having a long T6 in females. Males of *Oxyscelio codae* are especially difficult to distinguish from those of *Oxyscelio capilli*, another species from Sulawesi. They differ in that *Oxyscelio codae* has a more elongate, tapering metasoma in which T7 is rounded apically, with acuminate apical projections angled slightly towards one another. In *Oxyscelio capilli*, the metasoma is usually shorter and broader, with T7 more truncate apically, and with the inner margins of the acuminate apical projections being at right angles and not angled towards one another.

#### Etymology.

Vulgar Latin noun, genitive case, meaning “tail.” Refers to the mildly elongate metasoma.

#### Link to distribution map.

[http://hol.osu.edu/map-full.html?id=305708]

#### Material examined.

Holotype, female: **INDONESIA**: Sulawesi Utara Prov., Toraut, forest, Bogani Nani Wartabone (Dumoga-Bone) National Park, 9.V–16.V.1985, malaise trap, J. S. Noyes, OSUC 58664 (deposited in BMNH).*Paratypes*: **INDONESIA**: 22 females, 6 males, OSUC 369218-369219, 369225, 369228, 369236, 369240-369241, 369243-369244, 369253-369254, 369259, 369261, 369266, 369270, 369272, 369274, 369286, 369289-369290, 369293, 369296, 369302 (CNCI); OSUC 368947, 369258, 369265, 369284, 369292 (OSUC).

### Oxyscelio consobrinus

(Kieffer)

urn:lsid:zoobank.org:act:649DD4F0-A071-49BA-8D8B-1812733C6105

urn:lsid:biosci.ohio-state.edu:osuc_concepts:5012

http://species-id.net/wiki/Oxyscelio_consobrinus

**Figures 110–115.**
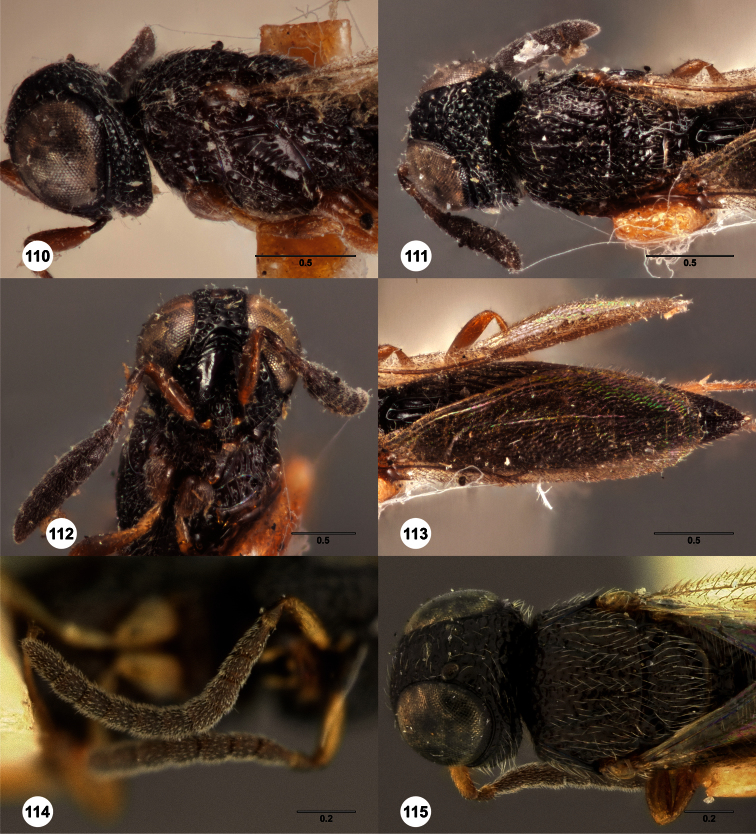
*Oxyscelio consobrinus* (Kieffer), holotype female (Museum Paris EY0000003995) **110 **Head and mesosoma, lateral view **111** Head and mesosoma, dorsal view **112** Head, anterior view **113** Metasoma, dorsal view. Paratype male (OSUC 268233) **114** Antennae **115** Mesosoma, dorsal view. Morphbank^45^

Figures 110–115 Morphbank^45^

Camptoteleia consobrina Kieffer, 1916: 171, 173 (original description, keyed); Kieffer 1926: 380, 382 (description, keyed); Kelner-Pillault 1958: 150 (type information).
Camptoteleia bifurcata Kieffer, 1916: 64, 172 (original description, keyed); Kieffer 1926: 380, 381 (description, keyed); Muesebeck and Walkley 1956: 339 (citation of type species). **syn. n.**
Camptoteleia frontalis Kieffer, 1916: 171, 175 (original description, keyed); Kieffer 1926: 380, 384 (description, keyed). **syn. n.**
Oxyscelio consobrinus (Kieffer): Dodd 1931: 75 (generic transfer).
Oxyscelio bifurcatus (Kieffer): Dodd 1931: 74 (generic transfer).
Oxyscelio frontalis (Kieffer): Dodd 1931: 75 (generic transfer).

#### Description.

*Female*. Body length 3.4–4.1 mm (n=14).

Radicle color: darker than scape. Scape color: Yellowish. A4: broader than long. A5: broader than long. Antennal club: formed, segments compact.

Interantennal process: not elongate. Median longitudinal elevation in frontal depression: absent. Frontal depression: concave. Frontal depression sculpture: with 2 complete transverse carinae. Submedian carina: weak, shallow and rounded or formed by ledge. Submedian carina medially: without peak. Concavity across dorsal part of frontal depression: absent. Depression extending ventrally from median ocellus: absent. Upper frons: not hood-like. Malar area near antennal foramen: without carina or expansion. Malar area at mouth corner: with radiating striae. Smooth strip along posterior side of malar sulcus: absent or not consistently broad. Middle genal carina: present. Direction of middle genal carina dorsally: parallel to eye margin. Major sculpture of gena anteriorly: umbilicate-foveate. Major sculpture of gena posteriorly: rugose. Microsculpture of gena anteroventrally: absent. Microsculpture of gena posteroventrally: absent. Median carina extending posteriorly from hyperoccipital carina: absent. Hyperoccipital carina: indicated by rugae. Lateral connection between hyperoccipital and occipital carinae: absent. Area between vertex and occipital carina: umbilicate-foveate. Occipital carina medially: uniformly rounded. Lateral corners of occipital carina: sharp and protruding.

Lateral pronotal area: without bulge projecting towards anterior pit. Epomial corner: strong. Netrion surface anteriorly: not inflexed. Mesoscutum anteriorly: not steep. Mesoscutal median carina: present and complete. Longitudinal carina between median carina and notauli: present. Major sculpture of medial mesoscutum anteriorly: umbilicate-foveate. Major sculpture of medial mesoscutum posteriorly: umbilicate-punctate. Microsculpture of medial mesoscutum anteriorly: granulate. Microsculpture of medial mesoscutum posteriorly: absent. Major sculpture of mesoscutellum: umbilicate-foveate; longitudinally rugose. Microsculpture of mesoscutellum medially: absent. Microsculpture of mesoscutellum laterally: absent. Mesoscutellar apex: convex or straight. Setae along anterior limit of femoral depression: arising from rows of foveae. Number of carinae crossing speculum above femoral depression: 2. Number of carinae crossing femoral depression: 3-5. Mesepimeral sulcus pits: more than 5. Metascutellum dorsally: concave. Metascutellar sculpture dorsally: smooth or with transverse carinae. Median carina of metascutellum: absent or branched. Metascutellar setae: absent. Metascutellar apex: weakly emarginate. Metapleuron above ventral metapleural area: crossed by carinae. Metasomal depression setae: absent. Lateral propodeal carinae anteromedially: strongly diverging. Anterior areoles of metasomal depression: absent. Anterior longitudinal carinae in metasomal depression: absent. Lateral propodeal areas: separated medially. Postmarginal vein: present. Fore wing apex: reaching middle of T5.

T1 midlobe: with 5 longitudinal carinae. T1: without anterior bulge. T2: with straight longitudinal striae or rugae. T6: broader than long. Apical flange of T6: not exposed apically. Metasomal apex: rounded. Major sculpture of T6: umbilicate-punctate; longitudinally striate or rugose. Microsculpture of T6: granulate.

*Male*. Body length 3.3–3.6 mm (n=13). A5 tyloid: carina-like, not expanded. A11: longer than broad; as long as broad. Median tooth of frontal depression: absent. Median lobe of T1: with 3 longitudinal carinae. Metasomal apex: with acuminate lateral corners.

**Diagnosis**. Both sexes: Middle genal carina subparallel with eye margin. Hyperoccipital carina indicated by rugae. Mesoscutum and mesoscutellum with very weak sculpture, giving them a melted appearance. Metascutellum concave dorsally, smooth aside from some transverse carinae. Female: A4, A5 not longer than broad. Antennal club very large. T1 midlobe with 5 longitudinal carinae. T6 rounded apically. Male: A11 slightly longer than broad. T1 midlobe with 3 longitudinal carinae. T7 with short, sharp and protruding posterolateral corners. *Oxyscelio consobrinus* is very similar to *Oxyscelio crebritas*, but differs in having much weaker sculpture on the mesoscutum and mesoscutellum.

#### Link to distribution map.

[http://hol.osu.edu/map-full.html?id=5012]

#### Material examined. 

Holotype, female, *Camptoteleia consobrina*: **PHILIPPINES**: Mindanao Isl., Butuan Chartered City, no date, Baker, Museum Paris EY0000003995 (deposited in MNHN). Neotype, female, *Oxyscelio bifurcatus*: **PHILIPPINES**: Negros Oriental Prov., 7km W Valencia, Cuernos de Negros Mountain, 700m, 17.V–25.V.1987, D. C. Darling, OSUC 369051 (deposited in CNCI). Neotype, male, *Oxyscelio frontalis*: **PHILIPPINES**: Negros Oriental Prov., 7km W Valencia, 1° forest edge, ROM 873068, Cuernos de Negros Mountain, 09°17'N, 123°15'E, 700m, 22.VIII-31.VIII.1987, malaise trap/pan trap, D. C. Darling & E. Mayordo, ROMEnt Spec. No. 112696 (deposited in ROME). *Other material*: **PHILIPPINES**: 13 females, 19 males, OSUC 448562 (BMNH); OSUC 369050 (CNCI); OSUC 228722, 251435, ROMEnt Spec. No. 112211, ROMEnt Spec. No. 112213, ROMEnt Spec. No. 112215, ROMEnt Spec. No. 112221, ROMEnt Spec. No. 112222, ROMEnt Spec. No. 112223, ROMEnt Spec. No. 112224, ROMEnt Spec. No. 112226, ROMEnt Spec. No. 112231, ROMEnt Spec. No. 112232, ROMEnt Spec. No. 112680, ROMEnt Spec. No. 112689, ROMEnt Spec. No. 112691, ROMEnt Spec. No. 112693, ROMEnt Spec. No. 112695 (ROME); OSUC 268225-268226, 268230, 268233, 268237, 268242-268244, 268250, 268254-268255, 268265, 268273 (USNM).

#### Comments.

The weak sculpture of *Oxyscelio consobrinus*, which can resemble melted plastic, is a distinctive trait common to many Philippine species. Recently collected specimens of this species have indicated that this was not an artefact of any unusual collecting or preservation methods.

The type material of *Camptoteleia bifurcata* Kieffer, collected from Mindanao (Butuan) in the Philippines, could not be found after an extensive search of collections known to house Kieffer type material. The neotype of *Camptoteleia bifurcata* is presently designated to clarify the taxonomic status of the species. It was selected because of its collection locality, and because it resembles Kieffer’s (1916) description in having a shiny mesosoma. In assigning a neotype for *Camptoteleia bifurcata*, we presumed that Kieffer (1916) was mistaken in his description of the fore wing venation. The “forked submarginal vein” seems to refer to the strongly tilted venation in which only the postmarginal vein closely approaches the anterior wing margin (the marginal vein is distant from the wing margin). This state is variable in many species of *Oxyscelio*, and therefore likely only indicates that a postmarginal vein is present. Females of *Oxyscelio consobrinus* can have a variably emarginate metascutellum, with extreme cases seeming bifurcate. This, and the description of the thorax as shiny, leads us to conclude that the lost type series of *Camptoteleia bifurcata* represented specimens of *Oxyscelio consobrinus* corresponding to the above criteria, which proved to fit within intraspecifc variation.

*Camptoteleia frontalis* Kieffer was described from the same locality as *Oxyscelio consobrinus*. Kieffer (1916) did mention a male specimen of *Oxyscelio bifurcatus*, but he did not discuss its mesoscutal surface sculpture. The type material of *Camptoteleia frontalis* could not be found after an extensive search of collections known to house Kieffer type material. The neotype of *Camptoteleia frontalis* is presently designated to clarify the taxonomic status of the species. It was selected because of its collection locality, and the relatively rough sculpture of the specimen (relative to other Philippine specimens, which are not roughly sculptured compared with most mainland Asian specimens). Mesoscutal surface sculpture in male *Oxyscelio consobrinus* is variable. Kieffer also described the metascutellum of *Oxyscelio frontalis* as bilobed, and therefore in assigning a neotype we conclude that *Oxyscelio frontalis* was a male *Oxyscelio consobrinus* with relatively rough surface sculpture, a broad metascutellum having an emarginate apex, and with the marginal vein in close contact with the anterior wing margin.

### Oxyscelio convergens

Burks sp. n.

urn:lsid:zoobank.org:act:E03A3DFC-3859-4097-9D95-508F16CF1C04

urn:lsid:biosci.ohio-state.edu:osuc_concepts:275500

http://species-id.net/wiki/Oxyscelio_convergens

**Figures 116–121.**
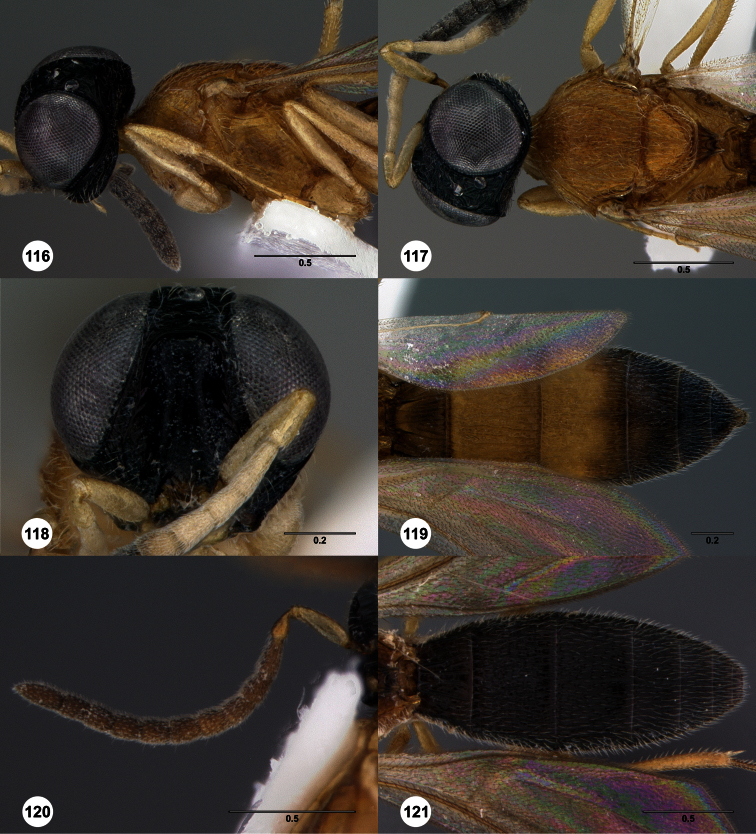
*Oxyscelio convergens* sp. n., holotype female (OSUC 368789) **116** Head and mesosoma, lateral view **117** Head and mesosoma, dorsal view **118** Head, anterior view **119** Metasoma, dorsal view. Paratype male (OSUC 368781) **120** Antenna **121** Metasoma, dorsal view. Morphbank^46^

Figures 116–121 Morphbank^46^

#### Description.

*Female*. Body length 3.5–3.75 mm (n=19).

Radicle color: same color as scape; darker than scape. Scape color: Yellowish. A4: longer than broad. A5: longer than broad. Antennal club: formed, segments compact.

Interantennal process: not elongate. Median longitudinal elevation in frontal depression: absent; present. Frontal depression: concave. Frontal depression sculpture: with 3 or more broadly interrupted transverse carinae. Submedian carina: strong, formed by a sharp raised carina. Submedian carina medially: without peak. Concavity across dorsal part of frontal depression: absent. Depression extending ventrally from median ocellus: absent. Upper frons: hood-like, protruding over pedicel when antenna at rest. Malar area near antennal foramen: without carina or expansion. Malar area at mouth corner: with radiating striae. Smooth strip along posterior side of malar sulcus: absent or not consistently broad. Middle genal carina: present. Direction of middle genal carina dorsally: parallel to eye margin. Major sculpture of gena anteriorly: umbilicate-foveate; rugose. Major sculpture of gena posteriorly: umbilicate-foveate; rugose. Microsculpture of gena anteroventrally: absent. Microsculpture of gena posteroventrally: absent. Median carina extending posteriorly from hyperoccipital carina: absent; present. Hyperoccipital carina: complete, continuous with anterior genal carina. Lateral connection between hyperoccipital and occipital carinae: absent. Area between vertex and occipital carina: with transverse carinae; irregularly rugose. Occipital carina medially: convex, with a sharp median peak. Lateral corners of occipital carina: not protruding.

Lateral pronotal area: without bulge projecting towards anterior pit. Epomial corner: weak. Netrion surface anteriorly: not inflexed. Mesoscutum anteriorly: not steep. Mesoscutal median carina: present and complete. Longitudinal carina between median carina and notauli: absent. Major sculpture of medial mesoscutum anteriorly: umbilicate-foveate. Major sculpture of medial mesoscutum posteriorly: umbilicate-foveate; longitudinally rugose. Microsculpture of medial mesoscutum anteriorly: granulate. Microsculpture of medial mesoscutum posteriorly: absent. Major sculpture of mesoscutellum: umbilicate-foveate; longitudinally rugose. Microsculpture of mesoscutellum medially: absent. Microsculpture of mesoscutellum laterally: absent. Mesoscutellar apex: convex or straight. Setae along anterior limit of femoral depression: arising from rows of foveae. Number of carinae crossing speculum above femoral depression: 3; 4. Number of carinae crossing femoral depression: more than 5. Mesepimeral sulcus pits: more than 5. Metascutellum dorsally: concave. Metascutellar sculpture dorsally: smooth or with transverse carinae. Median carina of metascutellum: absent or branched. Metascutellar setae: absent. Metascutellar apex: weakly emarginate. Metapleuron above ventral metapleural area: crossed by carinae. Metasomal depression setae: absent. Lateral propodeal carinae anteromedially: weakly diverging. Anterior areoles of metasomal depression: one or more areoles present. Anterior longitudinal carinae in metasomal depression: absent. Lateral propodeal areas: separated medially. Postmarginal vein: present. Fore wing apex: reaching apex of T5; reaching apex of T6; reaching beyond T6.

T1 midlobe: with 4 longitudinal carinae. T1: without anterior bulge. T2: with straight longitudinal striae or rugae. T6: broader than long. Apical flange of T6: exposed apically. Metasomal apex: rounded. Major sculpture of T6: umbilicate-punctate; longitudinally striate or rugose. Microsculpture of T6: absent.

*Male*. Body length 3.35–3.65 mm (n=20). A5 tyloid: carina-like, not expanded. A11: longer than broad. Median tooth of frontal depression: absent. Median lobe of T1: with 4 longitudinal carinae. Metasomal apex: with rounded but projecting lobe-like corners.

#### Diagnosis.

Both sexes: Frons without elevation between antennal foramen and eye. Hyperoccipital carina present, continuous with anterior genal carina. Metascutellum narrowing posteriorly, but deeply incised. Metasomal depression elongate, with extensive sculpture; lateral propodeal carinae narrowly separated anteriorly. Female: A4, A5 longer than broad. T1 midlobe with 4 longitudinal carinae. T6 rounded apically. Male: All flagellomeres longer than broad. T1 midlobe with 4 longitudinal carinae. T7 with rounded posterolateral corners.

#### Etymology.

Latin participle, meaning “converging.” Does not change spelling under different genders. Refers to the posteriorly convergent mesoscutellar sculpture.

#### Link to distribution map.

[http://hol.osu.edu/map-full.html?id=275500]

#### Material examined.

Holotype, female: **TAIWAN**: Taiwan Prov., Pingtung Co., Kenting National Park, 230m, V-1991, pan trap, Starr & Wu, OSUC 368789 (deposited in CNCI). *Paratypes*: **TAIWAN**: 18 females, 38 males, OSUC 368776, 368778-368780, 368782-368783, 368785-368786, 368788, 368790, 368792-368794, 368796-368799, 368801, 368803-368804, 368806-368807, 368814-368824, 368826-368827, 368830-368831, 368833, 368836-368837, 368839, 368842-368844, 368846 (CNCI); OSUC 199585-199586 (FSCA); OSUC 368781, 368800, 368809, 368811, 368841, 368845 (OSUC); OSUC 439690, 439692, 439954 (TARI).

#### Comments.

*Oxyscelio convergens* belongs to a set of species with an elongate metasomal depression (the median portion of the propodeum anterior to the propodeal foramen), and with an elongate, posteriorly narrowing but deeply incised metascutellum. The metasomal depression is extensively sculptured in these species. The anterior portion of the lateral propodeal carina, laterally bordering the metasomal depression, is also conspicuously long in these species.

### Oxyscelio cordis

Burks sp. n.

urn:lsid:zoobank.org:act:ED24C4B5-414C-4D6A-8713-14766B7DDB19

urn:lsid:biosci.ohio-state.edu:osuc_concepts:275553

http://species-id.net/wiki/Oxyscelio_cordis

**Figures 122–125.**
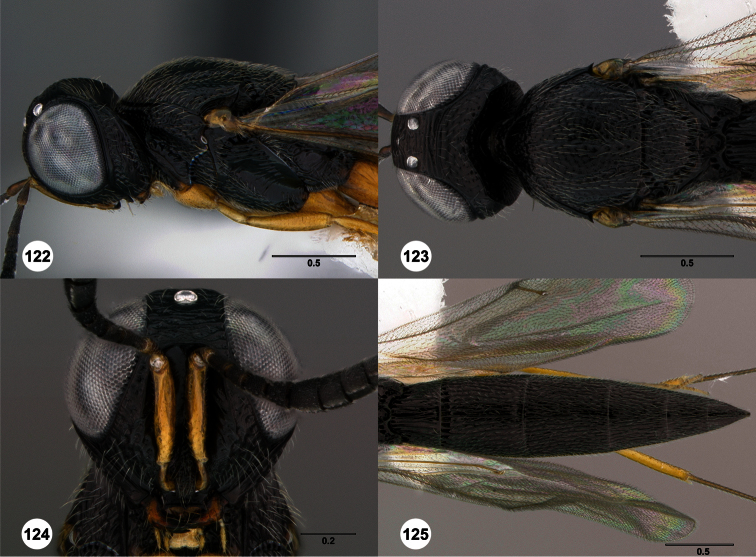
*Oxyscelio cordis* sp. n., holotype female (OSUC 368750) **122** Head and mesosoma, lateral view **123** Head and mesosoma, dorsal view **124** Head, anterior view **125** Metasoma, dorsal view. Morphbank^47^

Figures 122–125 Morphbank^47^

#### Description.

*Female*. Body length 4.5 mm (n=1).

Radicle color: same color as scape. Scape color: Yellowish. A4: longer than broad. A5: longer than broad. Antennal club: formed, segments compact.

Interantennal process: not elongate. Median longitudinal elevation in frontal depression: absent. Frontal depression: concave. Frontal depression sculpture: with 3 or more broadly interrupted transverse carinae. Submedian carina: strong, formed by a sharp raised carina. Submedian carina medially: without peak. Concavity across dorsal part of frontal depression: absent. Depression extending ventrally from median ocellus: absent. Upper frons: not hood-like. Malar area near antennal foramen: without carina or expansion. Malar area at mouth corner: with radiating striae. Smooth strip along posterior side of malar sulcus: absent or not consistently broad. Middle genal carina: present. Direction of middle genal carina dorsally: parallel to eye margin. Major sculpture of gena anteriorly: umbilicate-foveate. Major sculpture of gena posteriorly: umbilicate-punctate. Microsculpture of gena anteroventrally: absent. Microsculpture of gena posteroventrally: granulate. Median carina extending posteriorly from hyperoccipital carina: present. Hyperoccipital carina: indicated by rugae. Lateral connection between hyperoccipital and occipital carinae: present as a distinct carina. Area between vertex and occipital carina: umbilicate-foveate. Occipital carina medially: divided into concave halves, meeting at median peak. Lateral corners of occipital carina: sharp and protruding.

Lateral pronotal area: without bulge projecting towards anterior pit. Epomial corner: weak. Netrion surface anteriorly: not inflexed. Mesoscutum anteriorly: not steep. Mesoscutal median carina: present and complete. Longitudinal carina between median carina and notauli: absent. Major sculpture of medial mesoscutum anteriorly: umbilicate-foveate. Major sculpture of medial mesoscutum posteriorly: umbilicate-foveate. Microsculpture of medial mesoscutum anteriorly: granulate. Microsculpture of medial mesoscutum posteriorly: granulate. Major sculpture of mesoscutellum: umbilicate-foveate. Microsculpture of mesoscutellum medially: absent. Microsculpture of mesoscutellum laterally: absent. Mesoscutellar apex: incised. Setae along anterior limit of femoral depression: arising from rows of foveae. Number of carinae crossing speculum above femoral depression: 3. Number of carinae crossing femoral depression: more than 5. Mesepimeral sulcus pits: more than 5. Metascutellum dorsally: concave. Metascutellar sculpture dorsally: with scattered rugae. Median carina of metascutellum: absent or branched. Metascutellar setae: absent. Metascutellar apex: convex or straight. Metapleuron above ventral metapleural area: crossed by carinae. Metasomal depression setae: absent. Lateral propodeal carinae anteromedially: strongly diverging. Anterior areoles of metasomal depression: absent. Anterior longitudinal carinae in metasomal depression: absent. Lateral propodeal areas: meeting for only a short distance medially. Postmarginal vein: present. Fore wing apex: reaching middle of T6.

T1 midlobe: with 6 or more longitudinal carinae. T1: without anterior bulge. T2: with straight longitudinal striae or rugae. T6: longer than broad. Apical flange of T6: not exposed apically. Metasomal apex: rounded. Major sculpture of T6: umbilicate-punctate; longitudinally striate or rugose. Microsculpture of T6: granulate.

*Male*. Unknown.

#### Diagnosis.

Female: A4, A5 longer than broad. Frons without flange between antennal foramen and eye. Hyperoccipital carina present, continuous with an anterior genal carina, connected with occipital carina by a distinct longitudinal carina. Mesoscutellum without granulate sculpture. T1 midlobe with 6 or more longitudinal carinae. T6 strongly narrowed by not sharply pointed. The overall body shape recalls *Oxyscelio crateris*, which otherwise differs in having granulate mesoscutellar sculpture.

#### Etymology.

Latin noun, genitive case, meaning “heart.” Refers to mesoscutellar shape.

#### Link to distribution map.

[http://hol.osu.edu/map-full.html?id=275553]

#### Material examined.

Holotype, female: **THAILAND**: Nan Prov., office 9, T3239, Doi Phu Kha National Park, 19°12.252'N, 101°04.697'E, 1350m, 1.X–8.X.2007, malaise trap, Charoen & Nikom, OSUC 368750 (deposited in QSBG).

#### Comments.

*Oxyscelio cordis* is very unusual in having a posteriorly incised mesoscutellum, a feature that is otherwise found in some Australian species.

### Oxyscelio crassicornis

(Kieffer)

urn:lsid:zoobank.org:act:995E95E5-E6E4-4D0B-88B6-5BD6084F49A3

urn:lsid:biosci.ohio-state.edu:osuc_concepts:5013

http://species-id.net/wiki/Oxyscelio_crassicornis

**Figures 126–128.**
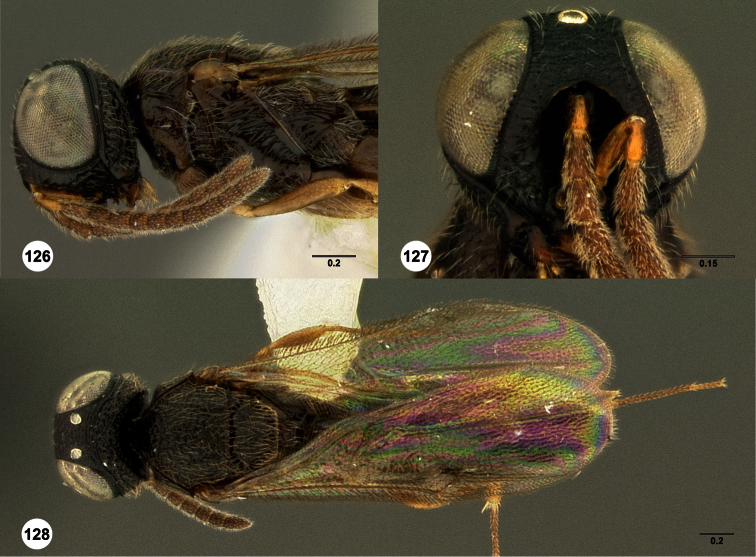
*Oxyscelio crassicornis* (Kieffer), neotype male (ROMEnt Spec. No. 112210) **126 **Head and mesosoma, lateral view **127** Head anterior view **128** Body, dorsal view. Morphbank^48^

Figures 126–128 Morphbank^48^

Camptoteleia crassicornis Kieffer, 1916: 171, 174 (original description, keyed); Kieffer 1926: 380, 385 (description, keyed).
Oxyscelio crassicornis (Kieffer): Dodd 1931: 75 (generic transfer).

#### Description.

*Female*. Unknown.

*Male*. Body length 3.35 mm (n=1).

Radicle color: darker than scape. Scape color: Brown. A5 tyloid: carina-like, not expanded. A11: longer than broad; as long as broad.

Interantennal process: not elongate. Median longitudinal elevation in frontal depression: absent. Frontal depression: concave. Frontal depression sculpture: without transverse or oblique carinae below submedian carina. Submedian carina: strong, formed by a sharp raised carina. Submedian carina medially: without peak. Median tooth of frontal depression: absent. Concavity across dorsal part of frontal depression: absent. Depression extending ventrally from median ocellus: absent. Upper frons: hood-like, protruding over pedicel when antenna at rest. Malar area near antennal foramen: without carina or expansion. Malar area at mouth corner: with radiating striae. Smooth strip along posterior side of malar sulcus: absent or not consistently broad. Middle genal carina: present. Direction of middle genal carina dorsally: parallel to eye margin. Major sculpture of gena anteriorly: umbilicate-foveate. Major sculpture of gena posteriorly: umbilicate-foveate. Microsculpture of gena anteroventrally: absent. Microsculpture of gena posteroventrally: absent. Median carina extending posteriorly from hyperoccipital carina: present. Hyperoccipital carina: complete, continuous with anterior genal carina. Lateral connection between hyperoccipital and occipital carinae: absent. Area between vertex and occipital carina: umbilicate-foveate. Occipital carina medially: convex, with a sharp median peak. Lateral corners of occipital carina: not protruding.

Lateral pronotal area: without bulge projecting towards anterior pit. Epomial corner: strong. Netrion surface anteriorly: not inflexed. Mesoscutum anteriorly: not steep. Mesoscutal median carina: present and complete. Longitudinal carina between median carina and notauli: absent. Major sculpture of medial mesoscutum anteriorly: umbilicate-foveate; irregularly rugose. Major sculpture of medial mesoscutum posteriorly: umbilicate-foveate; irregularly rugose. Microsculpture of medial mesoscutum anteriorly: granulate. Microsculpture of medial mesoscutum posteriorly: absent. Major sculpture of mesoscutellum: umbilicate-foveate. Microsculpture of mesoscutellum medially: absent. Microsculpture of mesoscutellum laterally: absent. Mesoscutellar apex: convex or straight. Setae along anterior limit of femoral depression: arising from rows of foveae. Number of carinae crossing speculum above femoral depression: 3. Number of carinae crossing femoral depression: more than 5. Mesepimeral sulcus pits: more than 5. Metascutellum dorsally: concave. Metascutellar sculpture dorsally: with scattered rugae. Median carina of metascutellum: absent or branched. Metascutellar setae: with many dorsal setae. Metascutellar apex: weakly emarginate. Metapleuron above ventral metapleural area: crossed by carinae. Metasomal depression setae: absent. Anterior areoles of metasomal depression: one or more areoles present. Anterior longitudinal carinae in metasomal depression: absent. Lateral propodeal areas: separated medially. Postmarginal vein: present.

Median lobe of T1: with 5 longitudinal carinae. Metasomal apex: with acuminate lateral corners.

#### Diagnosis.

Male: Frons without elevation between antennal foramen and eye. Frontal depression without tooth-like median protrusion dorsally. Hyperoccipital carina present, continuous with anterior genal carina; occipital carina complete medially and connected to hyperoccipital carina by a weak median carina. Metascutellum with dorsal setae. Metasomal depression elongate, with a single areole strongly defined by a posterior carina; lateral propodeal carinae narrowly separated anteriorly. T1 midlobe with 5 longitudinal carinae. T7 with sharp, narrowly protruding posterolateral corners.

#### Link to distribution map.

[http://hol.osu.edu/map-full.html?id=5013]

#### Material examined.

Neotype, male: **PHILIPPINES**: Negros Oriental Prov., 7km W Valencia, 1° forest edge, ROM 873058, Cuernos de Negros Mountain, 09°17'N, 123°15'E, 700m, 13.VI–19.VI.1987, malaise trap/pan trap, D. C. Darling & E. Mayordo, ROMEnt Spec. No. 112210 (deposited in ROME). *Other material*: **PHILIPPINES**: 1 male, OSUC 149520 (AEIC).

#### Comments.

The type material of *Camptoteleia crassicornis* Kieffer, collected from Mount Makiling, Luzon, in the Philippines, could not be found after an extensive search of collections known to house Kieffer type material. The neotype of *Camptoteleia crassicornis* is presently designated to clarify the taxonomic status of the species. It was selected because it possesses a convex mesosoma and discernably posteriorly carinate head as specified by Kieffer (1916). This choice presumes that Kieffer (1916) overlooked the metascutellar setae and misinterpreted the postmarginal vein. However, no other examined Philippine specimens bear close resemblance to Kieffer’s description. Our concept of *Oxyscelio crassicornis* indicates that it is very close to *Oxyscelio crustum*.

### Oxyscelio crateris

Burks sp. n.

urn:lsid:zoobank.org:act:EB1E6D51-A930-4C70-A5E0-A07ABC1816AB

urn:lsid:biosci.ohio-state.edu:osuc_concepts:275506

http://species-id.net/wiki/Oxyscelio_crateris

**Figures 129–132.**
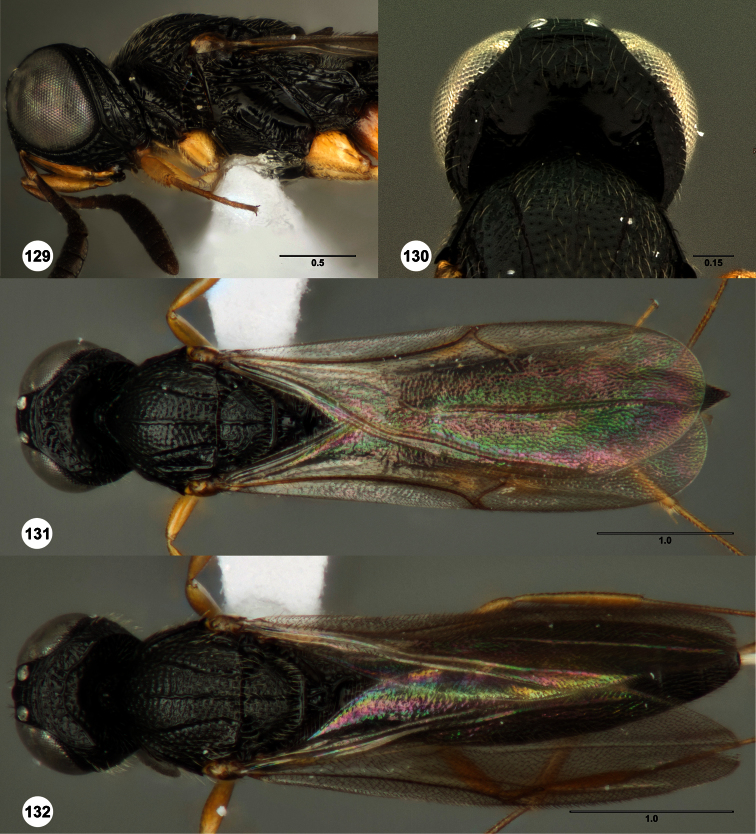
*Oxyscelio crateris* sp. n., holotype female (OSUC 336014) **129** Head and mesosoma, lateral view **130** Head, posterodorsal view **131** Body, dorsal view. Paratype male (OSUC 322129) **132 **Body, dorsal view. Morphbank^49^

Figures 129–132 Morphbank^49^

#### Description.

*Female*. Body length 4.65 mm (n=1).

Radicle color: same color as scape. Scape color: Yellowish. A4: longer than broad. A5: longer than broad. Antennal club: formed, segments compact.

Interantennal process: not elongate. Median longitudinal elevation in frontal depression: absent. Frontal depression: concave. Frontal depression sculpture: with 3 or more broadly interrupted transverse carinae. Submedian carina: strong, formed by a sharp raised carina. Submedian carina medially: without peak. Concavity across dorsal part of frontal depression: absent. Depression extending ventrally from median ocellus: absent. Upper frons: not hood-like. Malar area near antennal foramen: without carina or expansion. Malar area at mouth corner: with radiating striae. Smooth strip along posterior side of malar sulcus: present, broad throughout its length. Middle genal carina: present. Direction of middle genal carina dorsally: parallel to eye margin. Major sculpture of gena anteriorly: umbilicate-foveate; rugose. Major sculpture of gena posteriorly: umbilicate-foveate; rugose. Microsculpture of gena anteroventrally: absent. Microsculpture of gena posteroventrally: granulate. Median carina extending posteriorly from hyperoccipital carina: absent. Hyperoccipital carina: complete, continuous with anterior genal carina. Lateral connection between hyperoccipital and occipital carinae: present as a distinct carina. Area between vertex and occipital carina: irregularly rugose. Occipital carina medially: convex, with a sharp median peak. Lateral corners of occipital carina: sharp and protruding.

Lateral pronotal area: without bulge projecting towards anterior pit. Epomial corner: weak. Netrion surface anteriorly: not inflexed. Mesoscutum anteriorly: not steep. Mesoscutal median carina: present and complete. Longitudinal carina between median carina and notauli: absent. Major sculpture of medial mesoscutum anteriorly: umbilicate-foveate. Major sculpture of medial mesoscutum posteriorly: umbilicate-foveate; transversely rugose. Microsculpture of medial mesoscutum anteriorly: granulate. Microsculpture of medial mesoscutum posteriorly: granulate. Major sculpture of mesoscutellum: umbilicate-foveate; transversely rugose. Microsculpture of mesoscutellum medially: granulate. Microsculpture of mesoscutellum laterally: granulate. Mesoscutellar apex: convex or straight. Setae along anterior limit of femoral depression: arising from tiny pits. Number of carinae crossing speculum above femoral depression: 3. Number of carinae crossing femoral depression: 3-5. Mesepimeral sulcus pits: more than 5. Metascutellum dorsally: concave. Metascutellar sculpture dorsally: smooth or with transverse carinae. Median carina of metascutellum: absent or branched. Metascutellar setae: absent. Metascutellar apex: deeply emarginate. Metapleuron above ventral metapleural area: crossed by carinae. Metasomal depression setae: absent. Lateral propodeal carinae anteromedially: weakly diverging. Anterior areoles of metasomal depression: absent. Anterior longitudinal carinae in metasomal depression: absent. Lateral propodeal areas: separated medially. Postmarginal vein: present. Fore wing apex: reaching middle of T6.

T1 midlobe: with 5 longitudinal carinae. T1: without anterior bulge. T2: with straight longitudinal striae or rugae. T6: longer than broad. Apical flange of T6: exposed apically. Metasomal apex: rounded. Major sculpture of T6: umbilicate-punctate; longitudinally striate or rugose. Microsculpture of T6: absent.

*Male*. Body length 3.8–4.5 mm (n=10). A5 tyloid: carina-like, not expanded. A11: longer than broad. Median tooth of frontal depression: absent. Median lobe of T1: with 5 longitudinal carinae. Metasomal apex: with no distinct corners.

#### Diagnosis.

Both sexes: Frons without flange between antennal foramen and eye. Hyperoccipital carina present, continuous with an anterior genal carina, connected with occipital carina by a distinct longitudinal carina. Mesoscutellum with granulate sculpture. Metascutellum weakly emarginate or apically incised, posterior corners narrow. Female: A4, A5 longer than broad. T6 strongly narrowed but not sharply pointed. Male: A11 not longer than broad. T7 with weakly rounded lobes posterolaterally.

#### Etymology.

Latin noun, genitive case, meaning “crater.” Refers to the outlined concave area on the dorsal part of the occiput.

#### Link to distribution map.

[http://hol.osu.edu/map-full.html?id=275506]

#### Material examined.

Holotype, female: **THAILAND**: Chiang Mai Prov., Doi Phaluang, T2846, Doi Phahompok National Park, 20°00.966'N, 99°09.579'E, 1449m, 13.VII–20.VII.2007, malaise trap, P. Wongchai, OSUC 336014 (deposited in QSBG). *Paratypes*: (12 males) **MALAYSIA**: 2 males, OSUC 369012-369013 (CNCI). **THAILAND**: 10 males, OSUC 368749, 464036-464037 (CNCI); OSUC 322088, 322115 (OSUC); OSUC 322130, 352921, 368545, 368548 (QSBG); OSUC 322129 (WINC).

### Oxyscelio crebritas

Burks sp. n.

urn:lsid:zoobank.org:act:82C94912-5D20-4C31-8447-1E6F470BFA53

urn:lsid:biosci.ohio-state.edu:osuc_concepts:275371

http://species-id.net/wiki/Oxyscelio_crebritas

**Figures 133–138.**
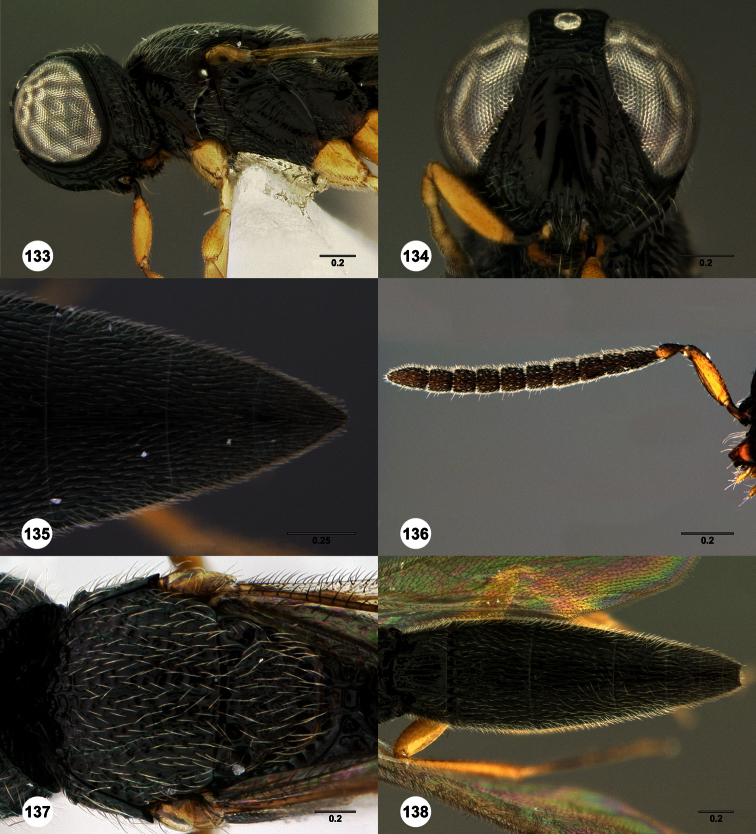
*Oxyscelio crebritas* sp. n., paratype female (OSUC 335200) **133** Head and mesosoma, lateral view **134** Head, anterior view. Paratype female (OSUC 320403) **135** Metasoma, dorsal view. Paratype male (OSUC 320416) **136** Antenna **137** Mesosoma, dorsal view. Paratype male (OSUC 322096) **138** Metasoma, dorsal view. Morphbank^50^

Figures 133–138 Morphbank^50^

#### Description.

*Female*. Body length 3.25–5.6 mm (n=20).

Radicle color: darker than scape. Scape color: Yellowish. A4: broader than long. A5: broader than long. Antennal club: formed, segments compact.

Interantennal process: not elongate. Median longitudinal elevation in frontal depression: absent. Frontal depression: concave. Frontal depression sculpture: with 3-5 complete transverse carinae. Submedian carina: strong, formed by a sharp raised carina. Submedian carina medially: without peak. Concavity across dorsal part of frontal depression: absent. Depression extending ventrally from median ocellus: absent. Upper frons: not hood-like. Malar area near antennal foramen: without carina or expansion. Malar area at mouth corner: with radiating striae. Smooth strip along posterior side of malar sulcus: absent or not consistently broad. Middle genal carina: present. Direction of middle genal carina dorsally: parallel to eye margin. Major sculpture of gena anteriorly: umbilicate-foveate. Major sculpture of gena posteriorly: umbilicate-foveate; rugose. Microsculpture of gena anteroventrally: absent. Microsculpture of gena posteroventrally: absent. Median carina extending posteriorly from hyperoccipital carina: absent. Hyperoccipital carina: not indicated medially; indicated by rugae. Lateral connection between hyperoccipital and occipital carinae: absent. Area between vertex and occipital carina: umbilicate-foveate. Occipital carina medially: uniformly rounded. Lateral corners of occipital carina: not protruding.

Lateral pronotal area: without bulge projecting towards anterior pit. Epomial corner: strong. Netrion surface anteriorly: not inflexed. Mesoscutum anteriorly: steep; not steep. Mesoscutal median carina: present and complete. Longitudinal carina between median carina and notauli: absent; present. Major sculpture of medial mesoscutum anteriorly: umbilicate-foveate. Major sculpture of medial mesoscutum posteriorly: umbilicate-foveate; umbilicate-punctate. Microsculpture of medial mesoscutum anteriorly: granulate. Microsculpture of medial mesoscutum posteriorly: absent. Major sculpture of mesoscutellum: umbilicate-foveate; longitudinally rugose. Microsculpture of mesoscutellum medially: absent. Microsculpture of mesoscutellum laterally: absent. Mesoscutellar apex: convex or straight. Setae along anterior limit of femoral depression: arising from rows of foveae. Number of carinae crossing speculum above femoral depression: 2. Number of carinae crossing femoral depression: 3-5. Mesepimeral sulcus pits: more than 5. Metascutellum dorsally: concave. Metascutellar sculpture dorsally: smooth or with transverse carinae. Median carina of metascutellum: absent or branched. Metascutellar setae: absent. Metascutellar apex: convex or straight. Metapleuron above ventral metapleural area: crossed by carinae. Metasomal depression setae: absent. Lateral propodeal carinae anteromedially: strongly diverging; weakly diverging. Anterior areoles of metasomal depression: absent. Anterior longitudinal carinae in metasomal depression: absent. Lateral propodeal areas: separated medially. Postmarginal vein: present. Fore wing apex: reaching middle of T5; reaching apex of T5; reaching apex of T6; reaching beyond T6; reaching middle of T6.

T1 midlobe: with 5 longitudinal carinae. T1: without anterior bulge. T2: with straight longitudinal striae or rugae. T6: broader than long. Apical flange of T6: exposed apically. Metasomal apex: rounded. Major sculpture of T6: umbilicate-punctate; longitudinally striate or rugose. Microsculpture of T6: absent; granulate.

*Male*. Body length 2.95–4.75 mm (n=20). A5 tyloid: carina-like, not expanded. A11: broader than long; as long as broad. Median tooth of frontal depression: absent. Median lobe of T1: with 3 longitudinal carinae. Metasomal apex: with acuminate lateral corners.

#### Diagnosis.

Both sexes: Middle genal carina subparallel with eye margin. Hyperoccipital carina absent or indicated by rugae. Mesoscutellum strongly umbilicate-foveolate, without granulate sculpture. Metascutellum concave dorsally, smooth aside from some transverse carinae. Female: A5 broader than long. T1 midlobe with 5 longitudinal carinae or a slight anterior bulge. T6 rounded apically and not longer than broad. Mesopleuron, along ventral margin of femoral depression, with only a few setae, these arising from foveae. Male: T1 midlobe with 3 longitudinal carinae. T7 with short, sharp and protruding posterolateral corners.

#### Etymology.

Latin noun in apposition to the generic name, meaning “common.”

#### Link to distribution map.

[http://hol.osu.edu/map-full.html?id=275371]

#### Material examined.

Holotype, female: **THAILAND**: Sakon Nakhon Prov., nr. office, dry evergreen, T2494, Phu Phan National Park, 16°48.618'N, 103°53.476'E, 526m, 4.VI-10.VI.2007, malaise trap, W. Kongnara, OSUC 336709 (deposited in QSBG). *Paratypes*: (134 females, 187 males, 1 unknown) **INDONESIA**: 15 females, 51 males, OSUC 376615 (BMNH); OSUC 368960, 369082, 369182, 369196, 369221, 369251, 369281, 369288, 369297, 369300 (CNCI); OSUC 228687, 228706, 228741, 228743-228745, 247961, 251438, 257045, 257047-257048, 257057, 257087, 257426, 464003-464004 (MBBJ); OSUC 228690, 228717, 228725, 228734-228735, 241816, 247814, 247817, 247819, 247833, 248891, 257419, 453947-453948, 464002, 58663 (OSUC); OSUC 228693-228694, 228707, 228718-228719, 240917, 247835, 247940, 257033, 257049, 257078, 257086, 257088, 464001, ROMEnt Spec. No. 112243, ROMEnt Spec. No. 112247, ROMEnt Spec. No. 112248, ROMEnt Spec. No. 112260 (ROME); OSUC 448565-448566, 448591, 448593, 453946 (WINC). **LAOS**: 8 females, 5 males, OSUC 368865-368866, 368871, 368879, 368892-368893, 368895, 368900, 368902-368904, 368907, 464009 (CNCI). **MALAYSIA**: 24 females, 37 males, 1 unknown, OSUC 376584-376585, 376600-376601, 376665-376666 (BMNH); OSUC 369011, 369014-369016, 369018, 369020, 369023-369025, 369028, 369066, 369307-369314, 369316-369319, 369321-369322, 369326, 369328, 369330-369332, 463991, 463993, 463995 (CNCI); OSUC 376743, 376745 (MCZC); OSUC 398961, 453761, 453770, 453780, 453790-453792 (OSUC); OSUC 436908-436921 (WINC).**NEPAL**: 7 females, 2 males, OSUC 238924 (BMNH); OSUC 369139, 369149, 369166, 369169, 369172-369173, 369177-369178 (CNCI). **SINGAPORE**: 1 female, 1 male, OSUC 376758-376759 (MCZC). **TAIWAN**: 1 female, 10 males, OSUC 368775, 368777, 368784, 368787, 368795, 368802, 368808, 368812, 368828-368829, 368849 (CNCI). **THAILAND**: 78 females, 73 males, OSUC 320403, 335200, 352496, 361368, 361370 (BMNH); OSUC 368609, 368619, 368678, 368692, 368694, 368702, 368706, 368708-368711, 368716-368717, 368719, 368727-368728, 368734, 368738, 368755-368756, 368759-368760, 462829-462830, 464014, 464016-464017, 464031-464033, 464035, 464038-464039, 464041, 464043, 464053 (CNCI); OSUC 251434, 280509, 280517, 285203, 320394, 320414, 322096, 335169, 335199, 335202, 335217, 335632, 336088-336090, 336164, 336780, 352495, 352497-352498, 352500, 352517, 352520-352522, 352526-352527, 352911, 352914-352915, 361293-361295, 361348, 361353, 361360, 361367, 361928, 361937, 368522 (OSUC); OSUC 224373, 237455, 247623, 252042, 257383, 257387, 280499, 280514-280515, 285200, 285218, 309713, 317856, 317867, 317878, 317887-317890, 320392, 320395, 320409, 320415-320417, 322072, 322097, 322131, 335072, 335146-335148, 335213-335216, 335937, 335984-335985, 336023, 336120, 336124, 336126-336127, 336761, 336782, 352499, 352501, 352516, 352518-352519, 352525, 352528, 352913, 361214, 361217, 361277, 361332, 361335, 361344, 361347, 361359, 361371-361372, 361934, 361936, 361965, 368504, 368515 (QSBG); UCRC ENT 135263 (UCRC). **VIETNAM**: 8 males, OSUC 369102, 369104-369105, 369110, 369124-369125 (CNCI); OSUC 240935, 352920 (ROME).

#### Comments.

*Oxyscelio crebritas* is one of the most commonly collected species of *Oxyscelio*. It exhibits some partially geographically correlated variation across its broad range. This includes variation in strength and number of carinae along the gena, metasomal length, wing color, and strength of sculpture. While it is possible to separate most females into three main variants in Thailand, Vietnam, and Borneo based on T1 midlobe sculpture, genal sculpture, and wing color, many males could not be definitively assigned to any of these forms. Nearly identical specimens collected from other areas were also difficult to distinguish from these forms. It is therefore possible that *Oxyscelio crebritas* represents a complex of sibling species. Regional variants should be more closely studied to test this. For the purposes of this revision, they were combined into a single species to avoid presenting a large number of named species that could hardly be identified.

### Oxyscelio crustum

Burks sp. n.

urn:lsid:zoobank.org:act:53098202-56D3-4738-9254-1E83B2A20C55

urn:lsid:biosci.ohio-state.edu:osuc_concepts:275555

http://species-id.net/wiki/Oxyscelio_crustum

**Figures 139–144.**
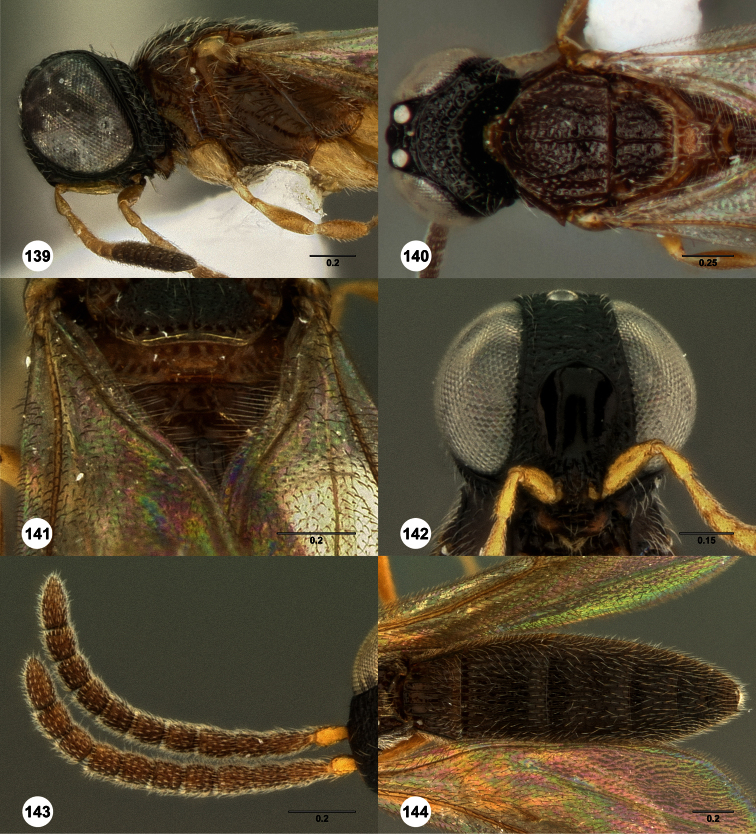
*Oxyscelio crustum* sp. n., paratype female (OSUC 352529) **139** Head and mesosoma, lateral view. Paratype female (OSUC 240929) **140** Head and mesosoma, dorsal view **141** Propodeum, posterior view. Holotype female (OSUC 369329) **142** Head, anterior view. Paratype male (OSUC 273320) **143** Antenna **144** Metasoma, dorsal view. Morphbank^51^

Figures 139–144 Morphbank^51^

#### Description.

*Female*. Body length 3.25–3.4 mm (n=4).

Radicle color: darker than scape. Scape color: Yellowish. A4: longer than broad. A5: longer than broad. Antennal club: formed, segments compact.

Interantennal process: not elongate. Median longitudinal elevation in frontal depression: absent. Frontal depression: concave. Frontal depression sculpture: without transverse or oblique carinae below submedian carina. Submedian carina: strong, formed by a sharp raised carina. Submedian carina medially: without peak. Concavity across dorsal part of frontal depression: absent. Depression extending ventrally from median ocellus: absent. Upper frons: not hood-like. Malar area near antennal foramen: without carina or expansion. Malar area at mouth corner: without striae. Smooth strip along posterior side of malar sulcus: absent or not consistently broad. Middle genal carina: present. Direction of middle genal carina dorsally: parallel to eye margin. Major sculpture of gena anteriorly: umbilicate-foveate. Major sculpture of gena posteriorly: umbilicate-foveate; rugose. Microsculpture of gena anteroventrally: absent. Microsculpture of gena posteroventrally: absent. Median carina extending posteriorly from hyperoccipital carina: absent. Hyperoccipital carina: complete, continuous with anterior genal carina. Lateral connection between hyperoccipital and occipital carinae: absent. Area between vertex and occipital carina: umbilicate-foveate; irregularly rugose. Occipital carina medially: uniformly rounded. Lateral corners of occipital carina: not protruding.

Lateral pronotal area: without bulge projecting towards anterior pit. Epomial corner: strong. Netrion surface anteriorly: not inflexed. Mesoscutum anteriorly: not steep. Mesoscutal median carina: present and complete. Longitudinal carina between median carina and notauli: absent. Major sculpture of medial mesoscutum anteriorly: umbilicate-foveate. Major sculpture of medial mesoscutum posteriorly: umbilicate-foveate. Microsculpture of medial mesoscutum anteriorly: granulate. Microsculpture of medial mesoscutum posteriorly: absent. Major sculpture of mesoscutellum: umbilicate-foveate; transversely rugose. Microsculpture of mesoscutellum medially: absent. Microsculpture of mesoscutellum laterally: absent. Mesoscutellar apex: convex or straight. Setae along anterior limit of femoral depression: arising from rows of foveae. Number of carinae crossing speculum above femoral depression: 3. Number of carinae crossing femoral depression: more than 5. Mesepimeral sulcus pits: more than 5. Metascutellum dorsally: concave. Metascutellar sculpture dorsally: with scattered rugae. Median carina of metascutellum: absent or branched. Metascutellar setae: with many dorsal setae. Metascutellar apex: weakly emarginate. Metapleuron above ventral metapleural area: crossed by carinae. Metasomal depression setae: absent. Lateral propodeal carinae anteromedially: weakly diverging. Anterior areoles of metasomal depression: one or more areoles present. Anterior longitudinal carinae in metasomal depression: absent. Lateral propodeal areas: separated medially. Postmarginal vein: present. Fore wing apex: reaching apex of T5; reaching middle of T6.

T1 midlobe: with 5 longitudinal carinae; obscured by other raised sculpture. T1: without anterior bulge; with small rounded anterior bulge, not reaching metascutellum. T2: with straight longitudinal striae or rugae. T6: broader than long; as long as broad. Apical flange of T6: exposed apically. Metasomal apex: rounded; tapering to a sharp point. Major sculpture of T6: umbilicate-punctate; longitudinally striate or rugose. Microsculpture of T6: absent.

*Male*. Body length 3.1–3.35 mm (n=). A5 tyloid: carina-like, not expanded. A11: broader than long; as long as broad. Median tooth of frontal depression: absent. Median lobe of T1: with 5 longitudinal carinae. Metasomal apex: with acuminate lateral corners.

#### Diagnosis.

Both sexes: Frons without elevation between antennal foramen and eye. Hyperoccipital carina present, continuous with anterior genal carina; occipital carina incomplete medially. Metascutellum with dorsal setae. Metasomal depression elongate, with a single areole strongly defined by a posterior carina; lateral propodeal carinae narrowly separated anteriorly. Female: A4, A5 longer than broad. T1 midlobe with 5 longitudinal carinae. Male: Frontal depression without tooth-like median protrusion dorsally. T1 midlobe with 5 longitudinal carinae. T7 with sharp, narrowly protruding posterolateral corners. Among members of the *Oxyscelio mesiodentis*-complex, *Oxyscelio crustum* is distinctive in having relatively weak surface sculpture. The propodeal areole and usually amber color can also help in distinguishing this species. It is very similar to the Philippine species *Oxyscelio crassicornis*, but lacks the median carina present between the hyperoccipital and occipital carinae in that species

#### Etymology.

Latin noun in apposition to the generic name, meaning “pie.” Suggested by the crinkly appearance of the surface sculpture.

#### Link to distribution map.

[http://hol.osu.edu/map-full.html?id=275555]

#### Material examined.

Holotype, female: **MALAYSIA**: Sabah St., rainforest edge, Danum Valley Protection Forest Reserve, 25.X–11.XII.1986, malaise trap, P. J. Eggleton, OSUC 369329 (deposited in BMNH). *Paratypes*: (3 females, 86 males) **BRUNEI**: 1 male, OSUC 376632 (BMNH). **INDONESIA**: 1 female, 37 males, OSUC 376659, 376662 (BMNH); OSUC 369081, 369084 (CNCI); OSUC 247843, 273320, 352906, ROMEnt Spec. No. 112252, ROMEnt Spec. No. 112259 (MBBJ); OSUC 228689, 247841, 247960, 247963, 257034, 257036, 257052-257053, 257420-257421, 257423, 257428 (OSUC); OSUC 228740, 240925, 240929, 247848, 247851-247852, 247861, 247863, 247967, 248898, 251432, 251436-251437, 251440, 257080, 361272, 361720 (ROME). **MALAYSIA**: 46 males, OSUC 203136 (AEIC); OSUC 376582 (BMNH); OSUC 368956, 369021-369022 (CNCI); OSUC 376750 (MCZC); OSUC 381323, 453754-453760, 453762, 453765-453766, 453769, 453773-453775, 453777, 453779, 453781, 453783-453786, 453793, 453796-453798, 453800-453802, 453804-453805, 453807-453809, 453811-453815 (OSUC); OSUC 448590 (WINC). **THAILAND**: 2 females, 4 males, OSUC 368491 (BMNH); OSUC 309264, 352529 (OSUC); OSUC 352459, 361363 (QSBG); OSUC 361215 (WINC).

### Oxyscelio cuculli

Burks sp. n.

urn:lsid:zoobank.org:act:D09D8C28-2F8A-4E65-A1B6-D442FB123BF9

urn:lsid:biosci.ohio-state.edu:osuc_concepts:275485

http://species-id.net/wiki/Oxyscelio_cuculli

**Figures 145–150.**
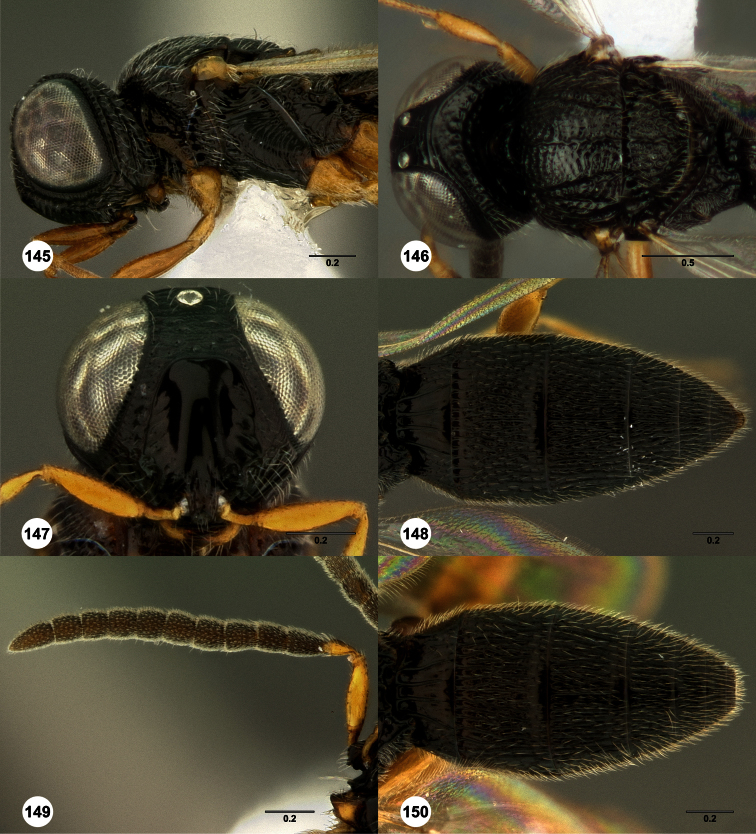
*Oxyscelio cuculli* sp. n., paratype female (OSUC 352910) **145** Head and mesosoma, lateral view **146** Head and mesosoma, dorsal view. Paratype female (OSUC 247614) **147** Head, anterior view. Paratype female (OSUC 352510) **148** Metasoma, dorsal view. Paratype male (OSUC 247647) **149 **Antenna. Paratype male (OSUC 228733) **150** Metasoma, dorsal view. Morphbank^52^

Figures 145–150 Morphbank^52^

#### Description.

*Female*. Body length 2.6–3.75 mm (n=20).

Radicle color: same color as scape; darker than scape. Scape color: Yellowish. A4: broader than long. A5: broader than long. Antennal club: formed, segments compact.

Interantennal process: not elongate. Median longitudinal elevation in frontal depression: absent; present. Frontal depression: concave. Frontal depression sculpture: with 3 or more broadly interrupted transverse carinae. Submedian carina: strong, formed by a sharp raised carina. Submedian carina medially: without peak. Concavity across dorsal part of frontal depression: absent. Depression extending ventrally from median ocellus: absent. Upper frons: hood-like, protruding over pedicel when antenna at rest. Malar area near antennal foramen: without carina or expansion. Malar area at mouth corner: with radiating striae. Smooth strip along posterior side of malar sulcus: absent or not consistently broad. Middle genal carina: present. Direction of middle genal carina dorsally: parallel to eye margin. Major sculpture of gena anteriorly: umbilicate-foveate. Major sculpture of gena posteriorly: umbilicate-foveate; rugose. Microsculpture of gena anteroventrally: absent. Microsculpture of gena posteroventrally: absent. Median carina extending posteriorly from hyperoccipital carina: absent. Hyperoccipital carina: complete, continuous with anterior genal carina. Lateral connection between hyperoccipital and occipital carinae: absent. Area between vertex and occipital carina: irregularly rugose. Occipital carina medially: sinuate, concave medial to corners, but without a median peak. Lateral corners of occipital carina: not protruding.

Lateral pronotal area: with slight bulge projecting anteriorly towards anterior pit. Epomial corner: weak. Netrion surface anteriorly: not inflexed. Mesoscutum anteriorly: steep. Mesoscutal median carina: present and complete. Longitudinal carina between median carina and notauli: absent. Major sculpture of medial mesoscutum anteriorly: umbilicate-foveate; transversely rugose. Major sculpture of medial mesoscutum posteriorly: umbilicate-foveate; transversely rugose. Microsculpture of medial mesoscutum anteriorly: granulate. Microsculpture of medial mesoscutum posteriorly: absent. Major sculpture of mesoscutellum: umbilicate-foveate; longitudinally rugose. Microsculpture of mesoscutellum medially: absent. Microsculpture of mesoscutellum laterally: absent. Mesoscutellar apex: convex or straight. Setae along anterior limit of femoral depression: arising from rows of foveae. Number of carinae crossing speculum above femoral depression: 3; 4. Number of carinae crossing femoral depression: more than 5. Mesepimeral sulcus pits: more than 5. Metascutellum dorsally: concave. Metascutellar sculpture dorsally: smooth or with transverse carinae. Median carina of metascutellum: absent or branched. Metascutellar setae: absent. Metascutellar apex: weakly emarginate. Metapleuron above ventral metapleural area: crossed by carinae. Metasomal depression setae: absent. Lateral propodeal carinae anteromedially: weakly diverging. Anterior areoles of metasomal depression: one or more areoles present. Anterior longitudinal carinae in metasomal depression: absent. Lateral propodeal areas: separated medially. Postmarginal vein: present. Fore wing apex: reaching apex of T6; reaching middle of T6.

T1 midlobe: with 4 longitudinal carinae. T1: without anterior bulge. T2: with straight longitudinal striae or rugae. T6: broader than long. Apical flange of T6: exposed apically. Metasomal apex: rounded. Major sculpture of T6: umbilicate-punctate; longitudinally striate or rugose. Microsculpture of T6: absent.

*Male*. Body length 2.95–3.8 mm (n=20). A5 tyloid: carina-like, not expanded. A11: broader than long. Median tooth of frontal depression: absent. Median lobe of T1: with 4 longitudinal carinae. Metasomal apex: with acuminate lateral corners.

#### Diagnosis.

Both sexes: Frons without elevation between antennal foramen and eye. Hyperoccipital carina present and sharp, continuous with anterior genal carina. Mesoscutellum without granulate sculpture. Metascutellum without dorsal setae. Propodeum without median carina; lateral propodeal carinae narrowly separated anteriorly. Female: T1 midlobe with 4 longitudinal carinae. T6 rounded apically. Male: A11 broader than long. T1 midlobe with 4 longitudinal carinae. T7 with acuminate posterolateral corners. *Oxyscelio cuculli* is similar to *Oxyscelio granorum*, but is usually smaller and has strongly foveate surface sculpture. Additionally, these species differ in A11 length and in shape of the T7 apex in males.

#### Etymology.

Latin noun, genitive case, meaning “hood.”

#### Link to distribution map.

[http://hol.osu.edu/map-full.html?id=275485]

#### Material examined.

Holotype, female: **THAILAND**: Trang Prov., Nam Tok Ton Yai, Khao Chong Mountain, 07°32'50"N, 99°47'20"E, 65m, 10.II.2005, malaise trap/pan trap, D. Yanega, UCRC ENT 149528 (deposited in UCRC).*Paratypes*: (77 females, 92 males) **INDIA**: 1 female, OSUC 376567 (BMNH). **INDONESIA**: 11 males, OSUC 228679, 240912, 247846, 248892, 257032 (MBBJ); OSUC 228678, 228680, 228682, 228692, 228733, 257062 (ROME). **LAOS**: 1 male, OSUC 368889 (CNCI). **MALAYSIA**: 1 female, 2 males, OSUC 369032, 369041 (CNCI); OSUC 381322 (QSBG). **NEPAL**: 2 females, OSUC 369167, 369176 (CNCI). **SRI LANKA**: 3 females, 1 male, OSUC 369085, 369094-369095 (CNCI); OSUC 442265 (QMBA). **TAIWAN**: 3 females, 7 males, OSUC 368791, 368805, 368810, 368825, 368847 (CNCI); OSUC 439689, 439691, 439693, 439697, 439699 (TARI). **THAILAND**: 65 females, 69 males, OSUC 335813, 335828, 361912-361913 (BMNH); OSUC 335812, 361917, 361919, 361964, 368597, 368611-368614, 368623, 368628, 368633, 368638, 368677, 368679, 368688, 368704, 368707, 368722-368723, 368725, 368730-368732, 368739-368741, 368769-368772, 368840, 464025 (CNCI); OSUC 237453, 247614, 247647, 247651-247652, 247655, 247892, 257391, 257393, 267440-267441, 280516, 285202, 285221, 285232-285236, 317877, 320376, 320383, 320410, 322118, 322125, 335090, 335800-335801, 335913, 352485, 352507-352508, 352510, 352910, 352916, 361211, 361213, 361219, 361297, 361358, 361907-361909, 361920, 361948 (OSUC); OSUC 237460, 247919, 251433, 257390, 257397, 257402, 280507, 280510-280511, 285220, 317863, 317866, 317870, 317876, 317886, 320386-320387, 320390, 320418, 322121, 322128, 335516, 335815, 335833, 335837, 335916, 336631, 336712, 336715, 336735, 361187, 361341, 361362, 361914-361916, 361933, 361939, 361941, 361955-361956, 368490, 368514, 368523-368524, 368526, 368536, 368544 (QSBG); OSUC 335806, 335811, 361910-361911 (WINC). **VIETNAM**: 2 females, 1 male, OSUC 277461, 277531 (RMNH); OSUC 352918 (ROME).

### Oxyscelio cupularis

(Kieffer)

urn:lsid:zoobank.org:act:319CCC31-561F-4C7C-8BFB-7794BAC207E9

urn:lsid:biosci.ohio-state.edu:osuc_concepts:5014

http://species-id.net/wiki/Oxyscelio_cupularis

**Figures 151–154.**
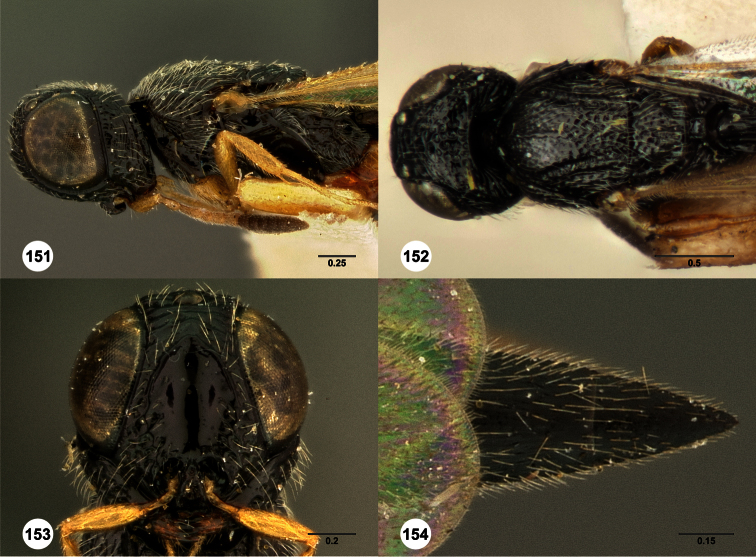
*Oxyscelio cupularis* (Kieffer), holotype female (OSUC 268223) **151** Head and mesosoma, dorsal view. Female (OSUC 268259) **152** Head and mesosoma, lateral view **153** Head, anterior view **154** Metasoma, dorsal view. Morphbank^53^

Figures 151–154 Morphbank^53^

Camptoteleia cupularis Kieffer, 1914: 296, 298 (original description, keyed); Kieffer 1916: 171 (keyed); Kieffer 1926: 380, 384 (description, keyed).
Oxyscelio cupularis (Kieffer): Dodd 1931: 75 (generic transfer).

#### Description.

*Female*. Body length 5.15–5.3 mm (n=2).

Radicle color: same color as scape. Scape color: Yellowish. A4: longer than broad. A5: longer than broad. Antennal club: formed, segments compact.

Interantennal process: not elongate. Median longitudinal elevation in frontal depression: absent. Frontal depression: concave. Frontal depression sculpture: with 2 oblique interrupted carinae. Submedian carina: strong, formed by a sharp raised carina. Submedian carina medially: with sharp peak. Concavity across dorsal part of frontal depression: absent. Depression extending ventrally from median ocellus: absent. Upper frons: not hood-like. Malar area near antennal foramen: with oblique tooth-like flange (facial nubbin). Malar area at mouth corner: without striae. Smooth strip along posterior side of malar sulcus: present, broad throughout its length. Middle genal carina: absent. Direction of middle genal carina dorsally: parallel to eye margin. Major sculpture of gena anteriorly: umbilicate-foveate. Major sculpture of gena posteriorly: umbilicate-foveate; rugose. Microsculpture of gena anteroventrally: absent. Microsculpture of gena posteroventrally: absent. Median carina extending posteriorly from hyperoccipital carina: absent. Hyperoccipital carina: not indicated medially. Lateral connection between hyperoccipital and occipital carinae: absent. Area between vertex and occipital carina: umbilicate-foveate. Occipital carina medially: sinuate, concave medial to corners, but without a median peak. Lateral corners of occipital carina: sharp and protruding.

Lateral pronotal area: without bulge projecting towards anterior pit. Epomial corner: strong. Netrion surface anteriorly: not inflexed. Mesoscutum anteriorly: not steep. Mesoscutal median carina: present and complete. Longitudinal carina between median carina and notauli: absent. Major sculpture of medial mesoscutum anteriorly: umbilicate-foveate. Major sculpture of medial mesoscutum posteriorly: umbilicate-foveate. Microsculpture of medial mesoscutum anteriorly: granulate. Microsculpture of medial mesoscutum posteriorly: absent. Major sculpture of mesoscutellum: umbilicate-foveate. Microsculpture of mesoscutellum medially: absent. Microsculpture of mesoscutellum laterally: granulate. Mesoscutellar apex: convex or straight. Setae along anterior limit of femoral depression: arising from rows of foveae. Number of carinae crossing speculum above femoral depression: 2. Number of carinae crossing femoral depression: 3-5. Mesepimeral sulcus pits: more than 5. Metascutellum dorsally: concave. Metascutellar sculpture dorsally: smooth or with transverse carinae. Median carina of metascutellum: absent or branched. Metascutellar setae: absent. Metascutellar apex: convex or straight; weakly emarginate. Metapleuron above ventral metapleural area: crossed by carinae. Metasomal depression setae: absent. Lateral propodeal carinae anteromedially: strongly diverging. Anterior areoles of metasomal depression: absent. Anterior longitudinal carinae in metasomal depression: absent. Lateral propodeal areas: separated medially. Postmarginal vein: present. Fore wing apex: reaching apex of T4.

T1 midlobe: obscured by other raised sculpture. T1: with small rounded anterior bulge, not reaching metascutellum. T2: with straight longitudinal striae or rugae. T6: longer than broad. Apical flange of T6: not exposed apically. Metasomal apex: rounded. Major sculpture of T6: umbilicate-punctate; longitudinally striate or rugose. Microsculpture of T6: granulate.

*Male*. Unknown.

#### Diagnosis.

Female: Antennal club formed. A4, A5 longer than broad. Face with oblique expanded flange between antennal foramen and eye. Metascutellum tiny, subrectangular.

#### Link to distribution map.

[http://hol.osu.edu/map-full.html?id=5014]

#### Material examined.

Neotype, female: **PHILIPPINES**: Laguna Prov., Mount Makiling (Maquiling), no date, Baker, OSUC 268223 (deposited in USNM). *Other material*: **PHILIPPINES**: 1 female, OSUC 268259 (USNM).

#### Comments.

The type material of *Camptoteleia cupularis* Kieffer, collected from Mount Makling, Luzon, in the Philippines, could not be found after an extensive search of collections known to house Kieffer type material. The neotype of *Camptoteleia cupularis* is presently designated to clarify the taxonomic status of the species. It was selected because of its collection locality and because it agrees with Kieffer’s (1914) description in having a long metasoma, poorly sculptured and shiny mesoscutum, and cupuliform metascutellum.

### Oxyscelio cyrtomesos

Burks sp. n.

urn:lsid:zoobank.org:act:4E5CB234-E34D-4E0A-96EE-BC1DD7BA4373

urn:lsid:biosci.ohio-state.edu:osuc_concepts:275531

http://species-id.net/wiki/Oxyscelio_cyrtomesos

Figures 155–169 Morphbank^54^

#### Description.

*Female*. Body length 4.05–4.4 mm (n=4).

Radicle color: same color as scape. Scape color: Yellowish. A4: broader than long; as long as broad. A5: broader than long. Antennal club: formed, segments compact.

Interantennal process: not elongate. Median longitudinal elevation in frontal depression: absent. Frontal depression: concave. Frontal depression sculpture: with 3-5 complete transverse carinae. Submedian carina: strong, formed by a sharp raised carina. Submedian carina medially: with sharp peak. Concavity across dorsal part of frontal depression: absent. Depression extending ventrally from median ocellus: absent. Upper frons: not hood-like. Malar area near antennal foramen: without carina or expansion. Malar area at mouth corner: without striae. Smooth strip along posterior side of malar sulcus: absent or not consistently broad. Middle genal carina: present. Direction of middle genal carina dorsally: parallel to eye margin. Major sculpture of gena anteriorly: umbilicate-foveate. Major sculpture of gena posteriorly: umbilicate-foveate; rugose. Microsculpture of gena anteroventrally: absent. Microsculpture of gena posteroventrally: absent. Median carina extending posteriorly from hyperoccipital carina: absent. Hyperoccipital carina: indicated by rugae. Lateral connection between hyperoccipital and occipital carinae: present as a weak elevation. Area between vertex and occipital carina: umbilicate-foveate. Occipital carina medially: sinuate, concave medial to corners, but without a median peak. Lateral corners of occipital carina: sharp and protruding.

Lateral pronotal area: without bulge projecting towards anterior pit. Epomial corner: weak. Netrion surface anteriorly: not inflexed. Mesoscutum anteriorly: not steep. Mesoscutal median carina: present and complete. Longitudinal carina between median carina and notauli: absent. Major sculpture of medial mesoscutum anteriorly: umbilicate-foveate. Major sculpture of medial mesoscutum posteriorly: umbilicate-foveate. Microsculpture of medial mesoscutum anteriorly: absent; granulate. Microsculpture of medial mesoscutum posteriorly: granulate. Major sculpture of mesoscutellum: umbilicate-foveate; umbilicate-punctate. Microsculpture of mesoscutellum medially: absent. Microsculpture of mesoscutellum laterally: granulate. Mesoscutellar apex: convex or straight. Setae along anterior limit of femoral depression: arising from rows of foveae. Number of carinae crossing speculum above femoral depression: 3. Number of carinae crossing femoral depression: more than 5. Mesepimeral sulcus pits: 3-5. Metascutellum dorsally: concave. Metascutellar sculpture dorsally: smooth or with transverse carinae. Median carina of metascutellum: absent or branched. Metascutellar setae: absent. Metascutellar apex: convex or straight. Metapleuron above ventral metapleural area: crossed by carinae. Metasomal depression setae: absent. Lateral propodeal carinae anteromedially: strongly diverging. Anterior areoles of metasomal depression: absent. Anterior longitudinal carinae in metasomal depression: absent. Lateral propodeal areas: meeting for only a short distance medially. Postmarginal vein: absent. Fore wing apex: reaching middle of T5; reaching apex of T5.

T1 midlobe: with 6 or more longitudinal carinae. T1: without anterior bulge. T2: with long sublateral depressions. T6: longer than broad. Apical flange of T6: not exposed apically. Metasomal apex: tapering to a sharp point. Major sculpture of T6: umbilicate-punctate. Microsculpture of T6: granulate.

*Male*. Body length 3.5–4.2 mm (n=8). A5 tyloid: carina-like, not expanded. A11: longer than broad. Median tooth of frontal depression: absent. Median lobe of T1: with 6 longitudinal carinae. Metasomal apex: with rounded but projecting lobe-like corners.

#### Diagnosis.

Both sexes: Mesoscutellum laterally granulate. Metascutellum long and tongue-shaped. Propodeum forming a nearly complete arch over the base of T1, but with a narrow break along middle of the arch. Female: A4, A5 broader than long. T1 midlobe with 6-7 longitudinal carinae. T2 with sublateral depressions. T6 strongly tapering to a narrow point. Male: A11 slightly broader than long. T1 midlobe with 5 longitudinal carinae. T7 with sharp, protruding posterolateral corners. *Oxyscelio cyrtomesos* is very similar to *Oxyscelio zeuctomesos*, but differs in development of the propodeal arch.

#### Etymology.

Compound noun based on Greek, meaning “convex middle.” Refers to the way that the propodeum arches medially over the base of T1.

#### Link to distribution map.

[http://hol.osu.edu/map-full.html?id=275531]

#### Material examined.

Holotype, female: **INDONESIA**: Kalimantan Barat Prov., Cabang Panti Research Station, 1° rainforest / alluvial light gap, IIS 910122, Gunung Palung National Park, 01°15'S, 110°05'E, 100–400m, 15.VI–15.VIII.1991, malaise trap, Darling & Rosichon, OSUC 247938 (deposited in MBBJ). *Paratypes*: (5 females, 10 males) **INDONESIA**: 2 females, 8 males, OSUC 228695, 247818, 247824, 247955-247956 (MBBJ); OSUC 247825, 247829, 247937, 247964, 257060 (ROME). **MALAYSIA**: 3 females, 2 males, OSUC 376583 (BMNH); OSUC 463990 (CNCI); OSUC 453799, 453803, 453806 (OSUC).

**Figures 155–159.**
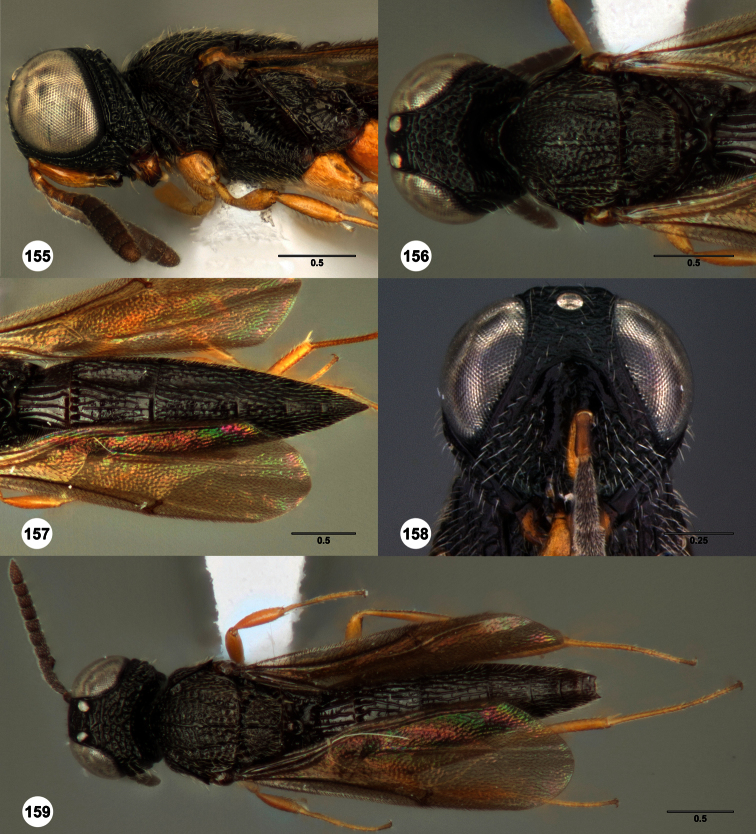
*Oxyscelio cyrtomesos* sp. n., holotype female (OSUC 247938) **155** Head and mesosoma, lateral view **156** Head and mesosoma, dorsal view **157** Metasoma, dorsal view. Paratype female (OSUC 247829) **158** Head, anterior view. Paratype male (OSUC 247964) **159** Body, dorsal view. Morphbank^54^

### Oxyscelio dasymesos

Burks sp. n.

urn:lsid:zoobank.org:act:58BBE82B-3696-4540-AE2E-3387CB5B1326

http://species-id.net/wiki/Oxyscelio_dasymesos

urn:lsid:biosci.ohio-state.edu:osuc_concepts:275536

**Figures 160–165.**
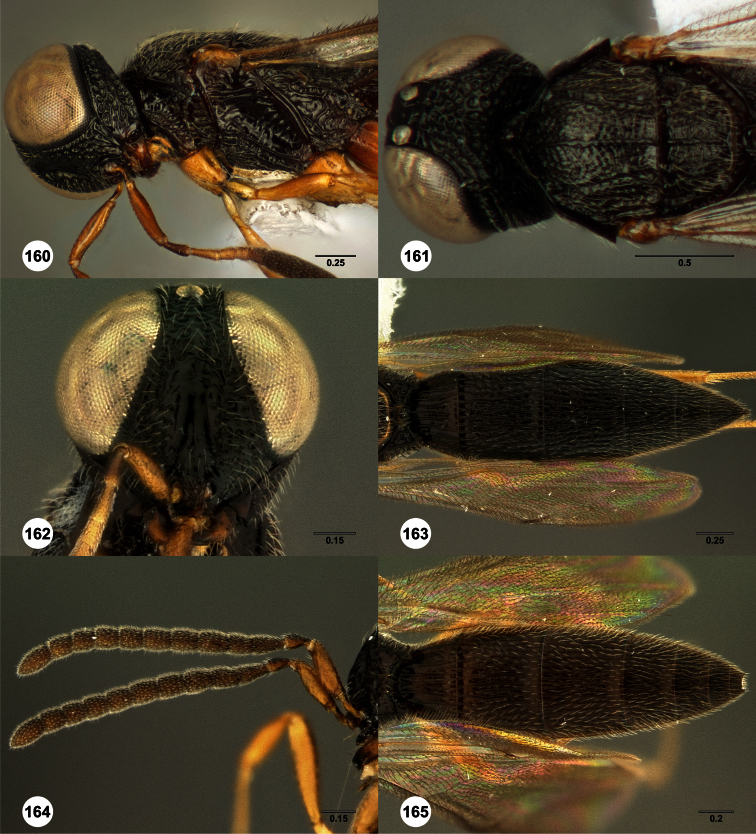
*Oxyscelio dasymesos* sp. n., paratype female (OSUC 247933) **160** Head and mesosoma, lateral view **161** Head and mesosoma, dorsal view **162** Head, anterior view. Paratype female (OSUC 376648) **163** Metasoma, dorsal view. Paratype male (OSUC 228732) **164** Antenna **165** Metasoma, dorsal view. Morphbank^55^

Figures 160–165 Morphbank^55^

#### Description.

*Female*. Body length 3.7–4.3 mm (n=9).

Radicle color: same color as scape. Scape color: Yellowish; Brown. A4: longer than broad. A5: longer than broad; as long as broad. Antennal club: formed, segments compact.

Interantennal process: not elongate. Median longitudinal elevation in frontal depression: present. Frontal depression: concave. Frontal depression sculpture: with 2 complete transverse carinae. Submedian carina: weak, shallow and rounded or formed by ledge. Submedian carina medially: without peak. Concavity across dorsal part of frontal depression: absent. Depression extending ventrally from median ocellus: absent. Upper frons: not hood-like. Malar area near antennal foramen: without carina or expansion. Malar area at mouth corner: with radiating striae. Smooth strip along posterior side of malar sulcus: absent or not consistently broad. Middle genal carina: present. Direction of middle genal carina dorsally: parallel to eye margin. Major sculpture of gena anteriorly: umbilicate-foveate; rugose. Major sculpture of gena posteriorly: umbilicate-foveate; rugose. Microsculpture of gena anteroventrally: absent. Microsculpture of gena posteroventrally: absent. Median carina extending posteriorly from hyperoccipital carina: absent. Hyperoccipital carina: indicated by rugae. Lateral connection between hyperoccipital and occipital carinae: present as a weak elevation. Area between vertex and occipital carina: umbilicate-foveate; irregularly rugose. Occipital carina medially: absent. Lateral corners of occipital carina: sharp and protruding.

Lateral pronotal area: without bulge projecting towards anterior pit. Epomial corner: weak. Netrion surface anteriorly: not inflexed. Mesoscutum anteriorly: not steep. Mesoscutal median carina: present and complete. Longitudinal carina between median carina and notauli: absent. Major sculpture of medial mesoscutum anteriorly: umbilicate-punctate; irregularly rugose. Major sculpture of medial mesoscutum posteriorly: umbilicate-punctate; transversely rugose. Microsculpture of medial mesoscutum anteriorly: absent. Microsculpture of medial mesoscutum posteriorly: absent. Major sculpture of mesoscutellum: umbilicate-foveate; transversely rugose. Microsculpture of mesoscutellum medially: absent. Microsculpture of mesoscutellum laterally: absent. Mesoscutellar apex: convex or straight. Setae along anterior limit of femoral depression: arising from rows of foveae. Number of carinae crossing speculum above femoral depression: 3. Number of carinae crossing femoral depression: 3-5. Mesepimeral sulcus pits: more than 5. Metascutellum dorsally: concave. Metascutellar sculpture dorsally: smooth or with transverse carinae. Median carina of metascutellum: absent or branched. Metascutellar setae: absent; with many dorsal setae. Metascutellar apex: deeply emarginate. Metapleuron above ventral metapleural area: crossed by carinae. Metasomal depression setae: present. Lateral propodeal carinae anteromedially: weakly diverging. Anterior areoles of metasomal depression: one or more areoles present. Anterior longitudinal carinae in metasomal depression: absent. Lateral propodeal areas: separated medially. Postmarginal vein: present. Fore wing apex: reaching middle of T6.

T1 midlobe: with 6 or more longitudinal carinae. T1: without anterior bulge. T2: with straight longitudinal striae or rugae. T6: broader than long. Apical flange of T6: not exposed apically. Metasomal apex: tapering to a sharp point. Major sculpture of T6: umbilicate-punctate; longitudinally striate or rugose. Microsculpture of T6: absent.

*Male*.Body length 3.1–4.15 mm (n=20). A5 tyloid: carina-like, not expanded. A11: longer than broad. Median tooth of frontal depression: absent. Median lobe of T1: with 5 longitudinal carinae; with 6 longitudinal carinae. Metasomal apex: with acuminate lateral corners.

#### Diagnosis.

Both sexes: Metascutellum deeply emarginate with rounded apical margin, not dorsally setose. Propodeum setose in metasomal depression, with an anterior subrectangular areole. Female: T1 midlobe with 6 longitudinal carinae. T6 apically tapering to a sharp point. Male: A11 longer than broad. T1 midlobe with 5-6 longitudinal carinae. T7 with sharp, protruding posterolateral corners.

#### Etymology.

Greek noun meaning “medial setae.” Refers to the setose metasomal depression.

#### Link to distribution map.

[http://hol.osu.edu/map-full.html?id=275536]

#### Material examined.

Holotype, female: **INDONESIA**: Kalimantan Barat Prov., Cabang Panti Research Station, RR6, 1° rainforest / sandstone closed canopy, IIS 910136, Gunung Palung National Park, 01°15'S, 110°05'E, 100m, 17.VI-29.VI.1991, canopy malaise trap, Darling, Rosichon & Sutrisno, OSUC 257091 (deposited in MBBJ). *Paratypes*: (8 females, 28 males) **BRUNEI**: 1 female, OSUC 376648 (BMNH). **INDONESIA**: 5 females, 24 males, OSUC 376651 (BMNH); OSUC 228703, 228731, 228747, 241813, 247840, 251429-251430 (MBBJ); OSUC 228691, 228701-228702, 228704, 228732, 228739, 240920, 247815-247816, 248922 (OSUC); OSUC 228699, 247837, 247933, 247957, 257039, 257041, 257064, 257066, 257068, 257071, 257079 (ROME). **MALAYSIA**: 2 females, 4 males, OSUC 376578 (BMNH); OSUC 369315, 369324-369325, 463994 (CNCI); OSUC 453767 (OSUC).

### Oxyscelio dasynoton

Burks sp. n.

urn:lsid:zoobank.org:act:1248D8C8-B3A7-4B1A-A8D2-C17E7A29D79F

urn:lsid:biosci.ohio-state.edu:osuc_concepts:275538

http://species-id.net/wiki/Oxyscelio_dasynoton

**Figures 166–171.**
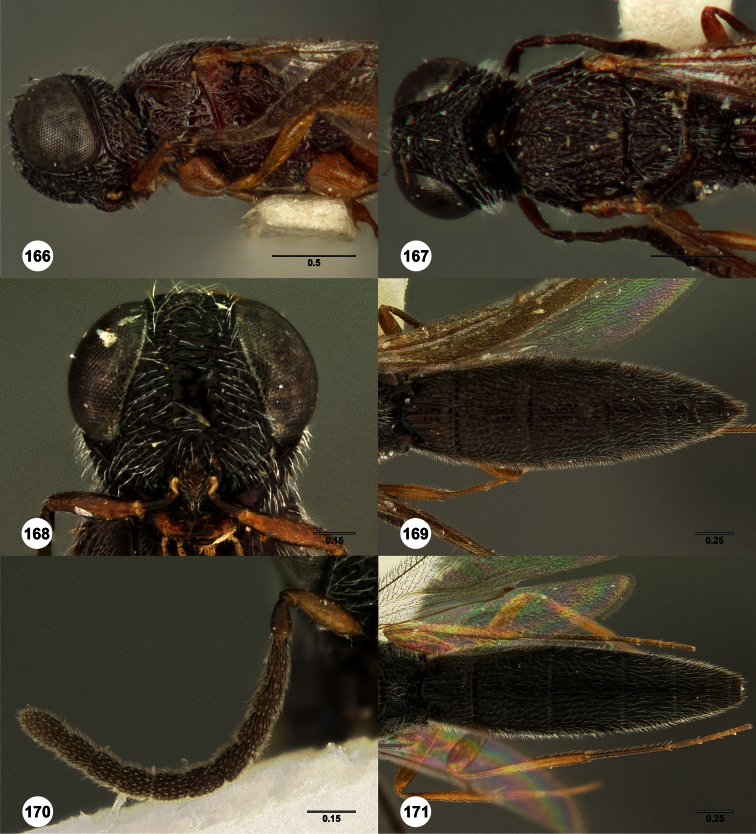
*Oxyscelio dasynoton* sp. n., holotype female (OSUC 268276) **166** Head and mesosoma, lateral view **167** Head and mesosoma, dorsal view **168** Head, anterior view **169** Metasoma, dorsal view. Paratype male (OSUC 369056) **170** Antenna **171** Metasoma, dorsal view. Morphbank^56^

Figures 166–171 Morphbank^56^

#### Description.

*Female*. Body length 4.1 mm (n=2).

Radicle color: darker than scape. Scape color: Brown. A4: longer than broad. A5: longer than broad; as long as broad. Antennal club: formed, segments compact.

Interantennal process: not elongate. Median longitudinal elevation in frontal depression: absent. Frontal depression: flat. Frontal depression sculpture: without transverse or oblique carinae below submedian carina. Submedian carina: weak, shallow and rounded or formed by ledge. Submedian carina medially: without peak. Concavity across dorsal part of frontal depression: absent. Depression extending ventrally from median ocellus: absent. Upper frons: not hood-like. Malar area near antennal foramen: without carina or expansion. Malar area at mouth corner: with radiating striae. Smooth strip along posterior side of malar sulcus: absent or not consistently broad. Middle genal carina: absent. Direction of middle genal carina dorsally: absent (replace with question mark). Major sculpture of gena anteriorly: umbilicate-foveate; rugose. Major sculpture of gena posteriorly: umbilicate-foveate; rugose. Microsculpture of gena anteroventrally: granulate. Microsculpture of gena posteroventrally: granulate. Median carina extending posteriorly from hyperoccipital carina: absent. Hyperoccipital carina: indicated by rugae. Lateral connection between hyperoccipital and occipital carinae: absent. Area between vertex and occipital carina: umbilicate-foveate. Occipital carina medially: uniformly rounded. Lateral corners of occipital carina: not protruding.

Lateral pronotal area: without bulge projecting towards anterior pit. Epomial corner: weak. Netrion surface anteriorly: not inflexed. Mesoscutum anteriorly: not steep. Mesoscutal median carina: present and complete. Longitudinal carina between median carina and notauli: absent. Major sculpture of medial mesoscutum anteriorly: umbilicate-foveate. Major sculpture of medial mesoscutum posteriorly: umbilicate-foveate. Microsculpture of medial mesoscutum anteriorly: absent. Microsculpture of medial mesoscutum posteriorly: granulate. Major sculpture of mesoscutellum: umbilicate-foveate. Microsculpture of mesoscutellum medially: granulate. Microsculpture of mesoscutellum laterally: granulate. Mesoscutellar apex: convex or straight. Setae along anterior limit of femoral depression: arising from rows of foveae. Number of carinae crossing speculum above femoral depression: 3. Number of carinae crossing femoral depression: 3-5. Mesepimeral sulcus pits: more than 5. Metascutellum dorsally: concave. Metascutellar sculpture dorsally: with scattered rugae. Median carina of metascutellum: absent or branched. Metascutellar setae: with many dorsal setae. Metascutellar apex: deeply emarginate. Metapleuron above ventral metapleural area: crossed by carinae. Metasomal depression setae: present. Lateral propodeal carinae anteromedially: weakly diverging. Anterior areoles of metasomal depression: one or more areoles present. Anterior longitudinal carinae in metasomal depression: absent. Lateral propodeal areas: separated medially. Postmarginal vein: present. Fore wing apex: reaching apex of T6.

T1 midlobe: with 5 longitudinal carinae. T1: without anterior bulge. T2: irregularly areolate. T6: broader than long. Apical flange of T6: exposed apically. Metasomal apex: tapering to a sharp point. Major sculpture of T6: umbilicate-punctate. Microsculpture of T6: granulate.

*Male*. Body length 3.75–3.8 mm (n=3). A5 tyloid: carina-like, not expanded. A11: longer than broad. Median tooth of frontal depression: absent. Median lobe of T1: with 6 longitudinal carinae. Metasomal apex: with acuminate lateral corners.

#### Diagnosis.

Both sexes: Metascutellum deeply emarginate with rounded apical margin, dorsally setose. Propodeum setose in metasomal depression, with an anterior subrectangular areole. Female: T1 midlobe with 5 longitudinal carinae. T6 apically tapering to a sharp point. Male: A11 longer than broad. T1 midlobe with 5 longitudinal carinae. T7 with sharp, protruding posterolateral corners. *Oxyscelio dasynoton* is similar to *Oxyscelio dasymesos* but has a broader, setose metascutellum and differs in surface sculpture.

#### Etymology.

Compound noun based on Greek, intended to mean “hairy back.” Refers to the setose metascutellum and metasomal depression.

#### Link to distribution map.

[http://hol.osu.edu/map-full.html?id=275538]

#### Material examined.

Holotype, female: **PHILIPPINES**: Laguna Prov., Luzon Isl., Mount Makiling, 1927, OSUC 268276 (deposited in USNM). *Paratypes*: **PHILIPPINES**: 1 female, 3 males, OSUC 369054, 369056 (CNCI); OSUC 268208, 268268 (USNM).

### Oxyscelio dermatoglyphes

Burks sp. n.

urn:lsid:zoobank.org:act:BF3D2E8D-3434-4B3B-88AA-70122522496D

urn:lsid:biosci.ohio-state.edu:osuc_concepts:275502

http://species-id.net/wiki/Oxyscelio_dermatoglyphes

**Figures 172–177.**
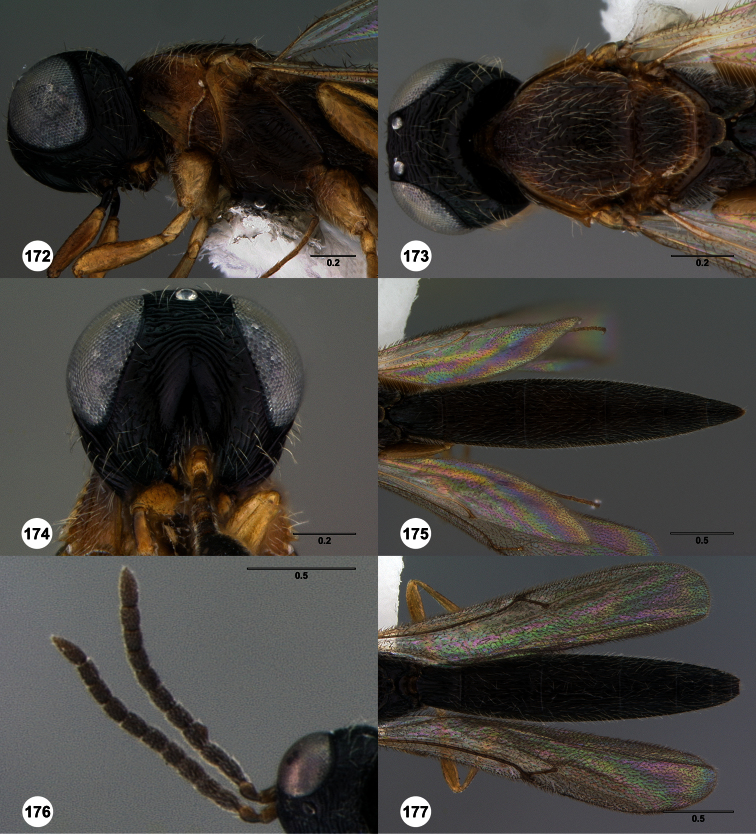
*Oxyscelio dermatoglyphes* sp. n., holotype female (OSUC 368835) **172** Head and mesosoma, lateral view **173** Head and mesosoma, dorsal view **174** Head, anterior view **175** Metasoma, dorsal view. Paratype male (OSUC 368838) **176** Antenna **177** Metasoma, dorsal view. Morphbank^57^

Figures 172–177 Morphbank^57^

#### Description.

*Female*. Body length 4.25–4.7 mm (n=12).

Radicle color: darker than scape. Scape color: Yellowish. A4: longer than broad. A5: longer than broad; as long as broad. Antennal club: formed, segments compact.

Interantennal process: not elongate. Median longitudinal elevation in frontal depression: absent. Frontal depression: concave. Frontal depression sculpture: without transverse or oblique carinae below submedian carina. Submedian carina: indicated by multiple weak carinae. Submedian carina medially: without peak. Concavity across dorsal part of frontal depression: absent. Depression extending ventrally from median ocellus: absent. Upper frons: not hood-like. Malar area near antennal foramen: without carina or expansion. Malar area at mouth corner: with radiating striae. Smooth strip along posterior side of malar sulcus: absent or not consistently broad. Middle genal carina: present. Direction of middle genal carina dorsally: parallel to eye margin. Major sculpture of gena anteriorly: rugose; umbilicate-punctate. Major sculpture of gena posteriorly: rugose; umbilicate-punctate. Microsculpture of gena anteroventrally: absent. Microsculpture of gena posteroventrally: absent. Median carina extending posteriorly from hyperoccipital carina: absent. Hyperoccipital carina: indicated by rugae. Lateral connection between hyperoccipital and occipital carinae: absent. Area between vertex and occipital carina: umbilicate-foveate. Occipital carina medially: uniformly rounded. Lateral corners of occipital carina: not protruding.

Lateral pronotal area: without bulge projecting towards anterior pit. Epomial corner: weak. Netrion surface anteriorly: not inflexed. Mesoscutum anteriorly: not steep. Mesoscutal median carina: present and complete. Longitudinal carina between median carina and notauli: absent. Major sculpture of medial mesoscutum anteriorly: umbilicate-foveate. Major sculpture of medial mesoscutum posteriorly: umbilicate-foveate. Microsculpture of medial mesoscutum anteriorly: granulate. Microsculpture of medial mesoscutum posteriorly: absent. Major sculpture of mesoscutellum: umbilicate-foveate; longitudinally rugose. Microsculpture of mesoscutellum medially: absent. Microsculpture of mesoscutellum laterally: absent. Mesoscutellar apex: convex or straight. Setae along anterior limit of femoral depression: arising from tiny pits. Number of carinae crossing speculum above femoral depression: 4. Number of carinae crossing femoral depression: more than 5. Mesepimeral sulcus pits: more than 5. Metascutellum dorsally: concave. Metascutellar sculpture dorsally: with scattered rugae. Median carina of metascutellum: absent or branched. Metascutellar setae: absent. Metascutellar apex: convex or straight. Metapleuron above ventral metapleural area: crossed by carinae. Metasomal depression setae: absent. Lateral propodeal carinae anteromedially: strongly diverging. Anterior areoles of metasomal depression: absent. Anterior longitudinal carinae in metasomal depression: absent. Lateral propodeal areas: separated medially. Postmarginal vein: present. Fore wing apex: reaching middle of T5.

T1 midlobe: obscured by other raised sculpture. T1: with small rounded anterior bulge, not reaching metascutellum. T2: with straight longitudinal striae or rugae. T6: longer than broad. Apical flange of T6: exposed apically. Metasomal apex: rounded. Major sculpture of T6: umbilicate-punctate; longitudinally striate or rugose. Microsculpture of T6: absent.

*Male*.Body length 3.55–4.5 mm (n=20). A5 tyloid: carina-like, not expanded. A11: longer than broad. Median tooth of frontal depression: absent. Median lobe of T1: with 6 longitudinal carinae. Metasomal apex: with tiny rounded tubercles.

#### Diagnosis.

Both sexes: Upper frons with extra carinae present parallel to submedian carina, all these carinae of about equal height. Hyperoccipital carina indicated by rugae. Mesoscutellum without granulate sculpture. Mesopleuron along anteroventral edge of femoral depression without row of foveae, setae arising from tiny pits. Female: A4 longer than broad. Metascutellum subrectangular, with scattered weak rugae. T1 midlobe with long anterior bulge. T6 longer than broad, tapering to a rounded apex. Male: A11 longer than broad. T1 midlobe with 4 longitudinal carinae. T7 with sharp and protruding posterolateral corners.

#### Etymology.

Compound noun based on Greek, intended to mean “fingerprint.” Refers to the pattern formed on the frons near the submedian carina.

#### Link to distribution map.

[http://hol.osu.edu/map-full.html?id=275502]

#### Material examined.

Holotype, female: **TAIWAN**: Taiwan Prov., Nantou Co., Lugu (Luku) Twp., Hsi-t’ou (Chito) Experimental Forest, 1100m, 20.IX.1997, B. J. Sinclair, OSUC 368835 (deposited in CNCI). *Paratypes*: **TAIWAN**: 13 females, 22 males, OSUC 368834, 368838 (CNCI); OSUC 368832 (OSUC); OSUC 439680-439681, 439683, 439687-439688, 439700-439715, 439717-439718, 439720-439727 (TARI); OSUC 442263 (WINC). *Other material*: **TAIWAN**: 1 unknown [consists only of mesosoma], OSUC 439686 (TARI).

#### Comments.

*Oxyscelio dermatoglyphes* is part of a species complex occurring also in Japan and Korea. This complex can be characterized by the elongate body, dark antennal radicle, weak occipital carina without protruding lateral corners, subrectangular flat metascutellum (reduced in males), and very strong T1 horn in females.

### Oxyscelio dorsalis

(Kieffer)

urn:lsid:zoobank.org:act:F419F35E-66BF-4A60-9AD0-5D800C2DB259

urn:lsid:biosci.ohio-state.edu:osuc_concepts:5015

http://species-id.net/wiki/Oxyscelio_dorsalis

**Figures 178–179.**
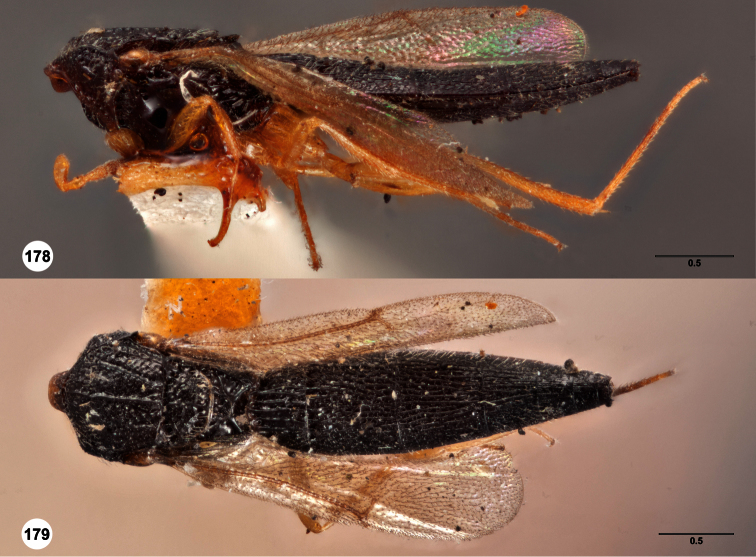
*Oxyscelio dorsalis* (Kieffer), holotype male (Museum Paris EY0000003994) **178 **Body, lateral view **179** Body, dorsal view. Morphbank^58^

Figures 178–179 Morphbank^58^

Camptoteleia dorsalis Kieffer, 1916: 64, 173 (original description, keyed); Kieffer 1926: 380, 381 (description, keyed); Kelner-Pillault 1958: 150 (type information).
Oxyscelio dorsalis (Kieffer): Dodd 1931: 75 (generic transfer).

#### Description.

*Female*. Unknown.

*Male*.Length of mesosoma plus metasoma (head of holotype missing): 3.5 mm (n=1). Epomial corner: weak. Netrion surface anteriorly: not inflexed. Mesoscutum anteriorly: not steep. Mesoscutal median carina: present and complete. Longitudinal carina between median carina and notauli: absent. Major sculpture of medial mesoscutum anteriorly: umbilicate-foveate. Major sculpture of medial mesoscutum posteriorly: umbilicate-foveate. Microsculpture of medial mesoscutum anteriorly: granulate. Microsculpture of medial mesoscutum posteriorly: absent. Major sculpture of mesoscutellum: umbilicate-foveate. Microsculpture of mesoscutellum medially: absent. Microsculpture of mesoscutellum laterally: granulate. Mesoscutellar apex: convex or straight. Number of carinae crossing speculum above femoral depression: 2. Metascutellum dorsally: flat. Metascutellar sculpture dorsally: with scattered rugae. Median carina of metascutellum: absent or branched. Metascutellar setae: absent. Metascutellar apex: convex or straight. Metapleuron above ventral metapleural area: crossed by carinae. Metasomal depression setae: absent. Anterior areoles of metasomal depression: absent. Anterior longitudinal carinae in metasomal depression: absent. Lateral propodeal areas: separated medially. Postmarginal vein: present.

Median lobe of T1: with 6 longitudinal carinae. Metasomal apex: with acuminate lateral corners.

#### Diagnosis.

Male: Mesoscutellum with granulate sculpture. T1 midlobe with 6 longitudinal carinae. T7 with sharp, protruding posterolateral corners.

#### Link to distribution map.

[http://hol.osu.edu/map-full.html?id=5015]

#### Material examined.

Holotype, male, *Camptoteleia dorsalis*: **PHILIPPINES**: Mindanao Isl., Butuan Chartered City, no date, Baker, Museum Paris EY0000003994 (deposited in MNHN).

#### Comments.

The damaged holotype of *Oxyscelio dorsalis* lacks a head. Nevertheless, the metascutellum and T1 midlobe indicate that this is a male of the *latitudinis*-group. Females are expected to have a broader, more strongly sculptured metascutellum and an elongate metasoma.

### Oxyscelio doumao

Burks sp. n.

urn:lsid:zoobank.org:act:FB6BAA50-D0F3-4F7B-B04E-7AF33F722FBC

urn:lsid:biosci.ohio-state.edu:osuc_concepts:305771

http://species-id.net/wiki/Oxyscelio_doumao

**Figures 180–183.**
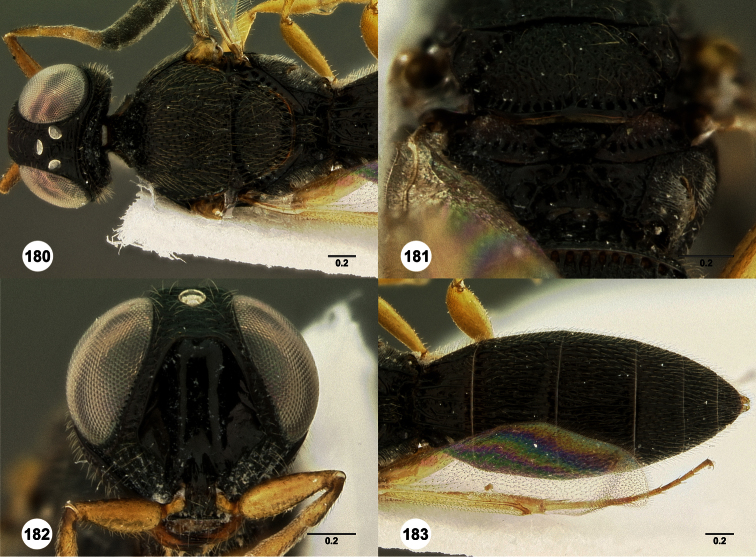
*Oxyscelio doumao* sp. n., holotype female (OSUC 448570) **180** Head and mesosoma, dorsal view **181** Propodeum, posterodorsal view **182** Head, anterior view **183** Metasoma, dorsal view. Morphbank^59^

Figures 180–183 Morphbank^59^

#### Description.

*Female*. Body length 4.55–4.7 mm (n=2).

Radicle color: same color as scape. Scape color: Yellowish. A4: longer than broad. A5: broader than long. Antennal club: formed, segments compact.

Interantennal process: not elongate. Median longitudinal elevation in frontal depression: absent. Frontal depression: concave. Frontal depression sculpture: without transverse or oblique carinae below submedian carina. Submedian carina: strong, formed by a sharp raised carina. Submedian carina medially: without peak. Concavity across dorsal part of frontal depression: absent. Depression extending ventrally from median ocellus: absent. Upper frons: hood-like, protruding over pedicel when antenna at rest. Malar area near antennal foramen: without carina or expansion. Malar area at mouth corner: with radiating striae. Smooth strip along posterior side of malar sulcus: absent or not consistently broad. Middle genal carina: absent. Direction of middle genal carina dorsally: parallel to eye margin. Major sculpture of gena anteriorly: umbilicate-foveate; rugose. Major sculpture of gena posteriorly: rugose; umbilicate-punctate. Microsculpture of gena anteroventrally: absent. Microsculpture of gena posteroventrally: absent. Median carina extending posteriorly from hyperoccipital carina: absent. Hyperoccipital carina: complete, continuous with anterior genal carina. Lateral connection between hyperoccipital and occipital carinae: absent. Area between vertex and occipital carina: irregularly rugose. Occipital carina medially: convex, with a sharp median peak. Lateral corners of occipital carina: not protruding.

Lateral pronotal area: with slight bulge projecting anteriorly towards anterior pit. Epomial corner: weak. Netrion surface anteriorly: not inflexed. Mesoscutum anteriorly: steep. Mesoscutal median carina: present and complete. Longitudinal carina between median carina and notauli: absent. Major sculpture of medial mesoscutum anteriorly: umbilicate-foveate. Major sculpture of medial mesoscutum posteriorly: umbilicate-foveate. Microsculpture of medial mesoscutum anteriorly: granulate. Microsculpture of medial mesoscutum posteriorly: absent. Major sculpture of mesoscutellum: umbilicate-foveate; longitudinally rugose. Microsculpture of mesoscutellum medially: granulate. Microsculpture of mesoscutellum laterally: absent. Mesoscutellar apex: convex or straight. Setae along anterior limit of femoral depression: arising from rows of foveae. Number of carinae crossing speculum above femoral depression: 4. Number of carinae crossing femoral depression: more than 5. Mesepimeral sulcus pits: more than 5. Metascutellum dorsally: concave. Metascutellar sculpture dorsally: with scattered rugae. Median carina of metascutellum: absent or branched. Metascutellar setae: with many dorsal setae. Metascutellar apex: deeply emarginate. Metapleuron above ventral metapleural area: crossed by carinae. Metasomal depression setae: absent. Lateral propodeal carinae anteromedially: weakly diverging. Anterior areoles of metasomal depression: absent. Anterior longitudinal carinae in metasomal depression: pair of submedian carinae present. Lateral propodeal areas: separated medially. Postmarginal vein: present; absent. Fore wing apex: reaching beyond T6.

T1 midlobe: with 4 longitudinal carinae. T1: without anterior bulge. T2: with straight longitudinal striae or rugae. T6: broader than long. Apical flange of T6: exposed apically. Metasomal apex: rounded. Major sculpture of T6: umbilicate-punctate. Microsculpture of T6: absent.

*Male*. Unknown.

#### Diagnosis.

Female: A4 longer than broad. Frons without elevation between antennal foramen and eye. Hyperoccipital carina present, continuous with anterior genal carina. Medial mesoscutum weakly sculptured, without longitudinal rugae. Metascutellum with dorsal setae. Metasomal depression short; lateral propodeal carinae narrowly separated anteriorly. Postmarginal vein very short or absent: less than 1/3 stigmal vein length, marginal vein very short but extending for a long distance from anterior wing margin. T1 with 4 longitudinal carinae, medial pair bending outward. *Oxyscelio doumao* is very similar to *Oxyscelio unguis* from Borneo, but differs in fore wing venation and in sculpture of the T1 midlobe. In *Oxyscelio unguis*, the medial pair of T1 midlobe carinae is broadly separated, with an incomplete median carina between them. In *Oxyscelio doumao*, these carinae are not broadly separated anteriorly, but one or both of them arch laterally in the posterior part of the T1.

**Etymology**. Mandarin noun, 兜帽 (dōumào), indicating the hood of a coat. Refers to the strongly hood-like submedian carina.

#### Link to distribution map.

[http://hol.osu.edu/map-full.html?id=305771]

#### Material examined.

Holotype, female: **CHINA**: Sichuan Prov., 29km N Muping (Baoxing), 1400m, 16.VIII-20.VIII.1995, malaise trap, R. E. Roughley, OSUC 448570 (deposited in ANIC). *Paratype*: **CHINA**: 1 female, OSUC 448571 (ANIC).

### Oxyscelio excavatus

(Kieffer)

urn:lsid:zoobank.org:act:7C8A7B0A-E175-4DB6-AE6C-13045066DF01

urn:lsid:biosci.ohio-state.edu:osuc_concepts:5016

http://species-id.net/wiki/Oxyscelio_excavatus

**Figures 184–187.**
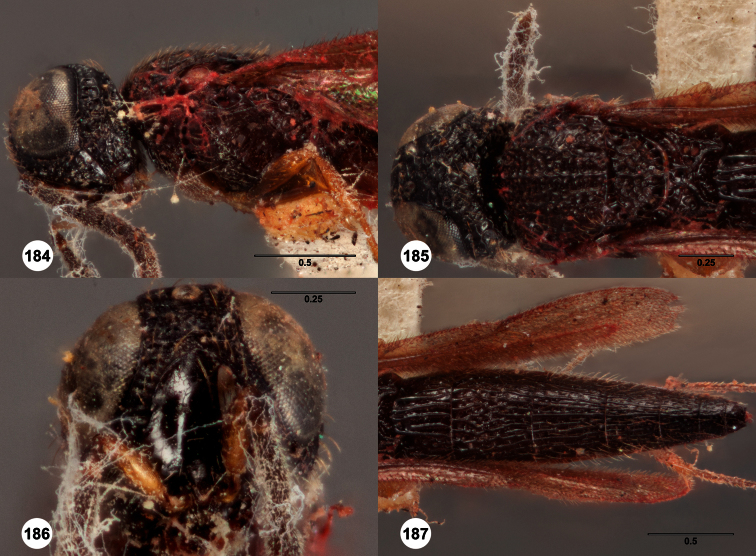
*Oxyscelio excavatus* (Kieffer), holotype male (Museum Paris EY0000003993) **184 **Head and mesosoma, lateral view **185** Head and mesosoma, dorsal view **186** Head, anterior view **187** Metasoma, dorsal view. Morphbank^60^

Figures 184–187 Morphbank^60^

Camptoteleia excavata Kieffer, 1913b: 387, 388 (original description., keyed); Kieffer 1914: 296 (keyed); Kieffer 1916: 171 (keyed); Kieffer 1926: 380, 383 (description, keyed); Kelner-Pillault 1958: 150 (type information).
Oxyscelio excavatus (Kieffer): Dodd 1931: 75 (generic transfer).

#### Description.

*Female*. Unknown.

*Male*. Body length 3.45 mm (n=1).

Radicle color: darker than scape. Scape color: Yellowish. A5 tyloid: carina-like, not expanded. A11: longer than broad.

Interantennal process: not elongate. Median longitudinal elevation in frontal depression: absent. Frontal depression: concave. Frontal depression sculpture: without transverse or oblique carinae below submedian carina. Submedian carina: strong, formed by a sharp raised carina. Submedian carina medially: without peak. Median tooth of frontal depression: absent. Concavity across dorsal part of frontal depression: absent. Depression extending ventrally from median ocellus: absent. Upper frons: not hood-like. Malar area near antennal foramen: without carina or expansion. Malar area at mouth corner: with radiating striae. Smooth strip along posterior side of malar sulcus: absent or not consistently broad. Middle genal carina: present. Direction of middle genal carina dorsally: parallel to eye margin. Major sculpture of gena anteriorly: umbilicate-foveate. Major sculpture of gena posteriorly: umbilicate-foveate; rugose. Microsculpture of gena anteroventrally: absent. Microsculpture of gena posteroventrally: absent. Median carina extending posteriorly from hyperoccipital carina: absent. Hyperoccipital carina: indicated by rugae. Lateral connection between hyperoccipital and occipital carinae: absent. Area between vertex and occipital carina: umbilicate-foveate. Occipital carina medially: uniformly rounded. Lateral corners of occipital carina: not protruding.

Lateral pronotal area: without bulge projecting towards anterior pit. Epomial corner: strong. Netrion surface anteriorly: not inflexed. Mesoscutum anteriorly: not steep. Mesoscutal median carina: present and complete. Longitudinal carina between median carina and notauli: absent. Major sculpture of medial mesoscutum anteriorly: umbilicate-foveate. Major sculpture of medial mesoscutum posteriorly: umbilicate-foveate. Microsculpture of medial mesoscutum anteriorly: absent. Microsculpture of medial mesoscutum posteriorly: absent. Major sculpture of mesoscutellum: umbilicate-foveate. Microsculpture of mesoscutellum medially: absent. Microsculpture of mesoscutellum laterally: absent. Mesoscutellar apex: convex or straight. Setae along anterior limit of femoral depression: arising from rows of foveae. Number of carinae crossing speculum above femoral depression: 2. Number of carinae crossing femoral depression: 3-5. Mesepimeral sulcus pits: more than 5. Metascutellum dorsally: concave. Metascutellar sculpture dorsally: smooth or with transverse carinae. Median carina of metascutellum: absent or branched. Metascutellar setae: absent. Metascutellar apex: convex or straight. Metapleuron above ventral metapleural area: foveate or rugose. Metasomal depression setae: absent. Anterior areoles of metasomal depression: absent. Anterior longitudinal carinae in metasomal depression: absent. Lateral propodeal areas: separated medially. Postmarginal vein: present.

Median lobe of T1: with 4 longitudinal carinae. Metasomal apex: with acuminate lateral corners.

#### Diagnosis.

Male: A11 longer than broad. Fore wing long enough to reach apex of T5. T1 midlobe with 4 longitudinal carinae. T7 with short, sharp and protruding posterolateral corners. Female specimens should be similar to those of *Oxyscelio consobrinus*, but are expected to differ in having a longer metasoma and more T1 midlobe carinae, which are the chief differences between the males of these species.

#### Link to distribution map.

[http://hol.osu.edu/map-full.html?id=5016]

#### Material examined.

Holotype, male, *Camptoteleia excavata*: **PHILIPPINES**: Laguna Prov., Los Baños, no date, Baker, Museum Paris EY0000003993 (deposited in MNHN).

### Oxyscelio fistulae

Burks sp. n.

urn:lsid:zoobank.org:act:6DD43E64-2F87-4451-ABDC-FC0A00A4EF0D

urn:lsid:biosci.ohio-state.edu:osuc_concepts:275543

http://species-id.net/wiki/Oxyscelio_fistulae

**Figures 188–191.**
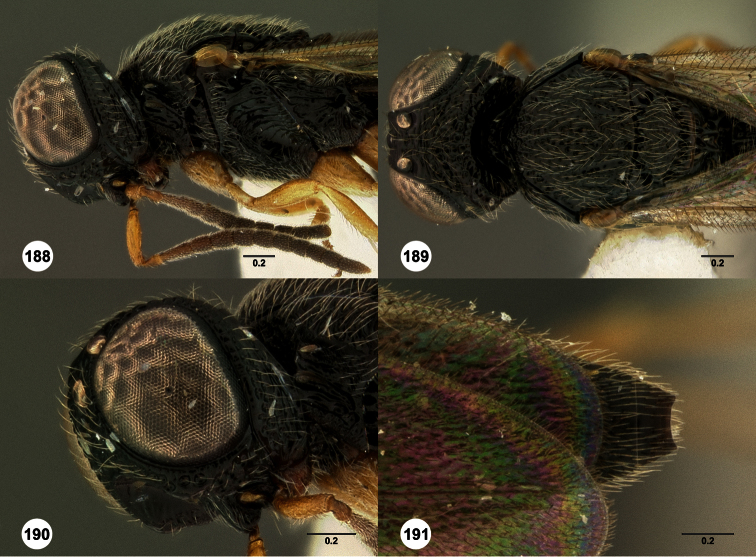
*Oxyscelio fistulae* sp. n., paratype male (OSUC 376621) **188** Head and mesosoma, lateral view **189** Head and mesosoma, dorsal view **190** Head, oblique view. Paratype male (OSUC 376616) **191** Metasoma, dorsal view. Morphbank^61^

Figures 188–191 Morphbank^61^

#### Description.

*Female*. Unknown.

*Male*.Body length 4.7–4.9 mm (n=3).

Radicle color: darker than scape. Scape color: Yellowish. Antennal club: formed, segments compact. A5 tyloid: carina-like, not expanded. A11: longer than broad.

Interantennal process: elongate. Median longitudinal elevation in frontal depression: absent. Frontal depression: concave. Frontal depression sculpture: with 3-5 complete transverse carinae. Submedian carina: strong, formed by a sharp raised carina. Submedian carina medially: without peak. Median tooth of frontal depression: absent. Concavity across dorsal part of frontal depression: absent. Depression extending ventrally from median ocellus: absent. Upper frons: not hood-like. Malar area near antennal foramen: without carina or expansion. Malar area at mouth corner: with radiating striae. Smooth strip along posterior side of malar sulcus: absent or not consistently broad. Middle genal carina: present. Direction of middle genal carina dorsally: parallel to eye margin. Major sculpture of gena anteriorly: umbilicate-foveate. Major sculpture of gena posteriorly: rugose. Microsculpture of gena anteroventrally: absent. Microsculpture of gena posteroventrally: absent. Median carina extending posteriorly from hyperoccipital carina: absent. Hyperoccipital carina: indicated by rugae. Lateral connection between hyperoccipital and occipital carinae: absent. Area between vertex and occipital carina: umbilicate-foveate; irregularly rugose. Occipital carina medially: absent. Lateral corners of occipital carina: sharp and protruding.

Lateral pronotal area: without bulge projecting towards anterior pit. Epomial corner: weak. Netrion surface anteriorly: not inflexed. Mesoscutum anteriorly: not steep. Mesoscutal median carina: present and complete. Longitudinal carina between median carina and notauli: absent. Major sculpture of medial mesoscutum anteriorly: umbilicate-foveate. Major sculpture of medial mesoscutum posteriorly: umbilicate-foveate. Microsculpture of medial mesoscutum anteriorly: granulate. Microsculpture of medial mesoscutum posteriorly: granulate. Major sculpture of mesoscutellum: umbilicate-foveate; longitudinally rugose. Microsculpture of mesoscutellum medially: absent. Microsculpture of mesoscutellum laterally: granulate. Mesoscutellar apex: convex or straight. Setae along anterior limit of femoral depression: arising from rows of foveae. Number of carinae crossing speculum above femoral depression: 2. Number of carinae crossing femoral depression: more than 5. Mesepimeral sulcus pits: more than 5. Metascutellum dorsally: concave. Metascutellar sculpture dorsally: smooth or with transverse carinae. Median carina of metascutellum: absent or branched. Metascutellar setae: absent. Metascutellar apex: weakly emarginate. Metapleuron above ventral metapleural area: crossed by carinae. Metasomal depression setae: absent. Anterior areoles of metasomal depression: absent. Anterior longitudinal carinae in metasomal depression: absent. Lateral propodeal areas: meeting for only a short distance medially. Postmarginal vein: present.

Median lobe of T1: with 5 longitudinal carinae. Metasomal apex: with acuminate lateral corners.

#### Diagnosis.

Male: A11 longer than broad. Interantennal process elongate, with a concave, setose apex. Mesoscutellum with granulate sculpture laterally. Metascutellum narrow, angularly emarginate apically. Propodeum with a short expansion where the lateral propodeal carinae meet anteriorly. T1 midlobe with 5 longitudinal carinae. T7 with short, sharp posterolateral corners.

#### Etymology.

Latin noun, genitive case, meaning “tube.”

#### Link to distribution map.

[http://hol.osu.edu/map-full.html?id=275543]

#### Material examined.

Holotype, male: **INDONESIA**: Sulawesi Tengah Prov., nr. Morowali, Ranu River area, 27.I–20.IV.1980, M. J. D. Brendell, OSUC 376620 (deposited in BMNH). *Paratypes*: **INDONESIA**: 2 males, OSUC 376616, 376621 (BMNH).

#### Comments.

*Oxyscelio fistulae* is unusual in having an elongate interantennal process. This state is also found in a species from New Guinea and New Britain, but the process is differently shaped in that species.

### Oxyscelio flabelli

Burks sp. n.

urn:lsid:zoobank.org:act:8AB217B0-511B-432F-BD59-E6B267AC1C9D

urn:lsid:biosci.ohio-state.edu:osuc_concepts:275510

http://species-id.net/wiki/Oxyscelio_flabelli

**Figures 192–195.**
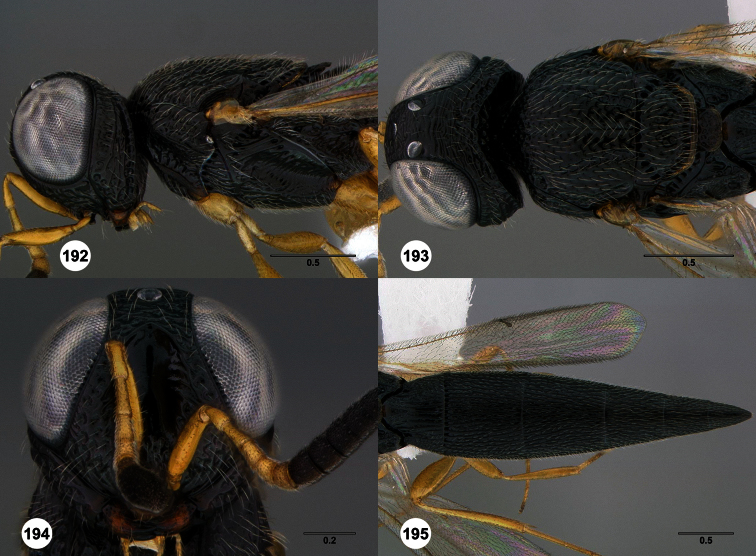
*Oxyscelio flabelli* sp. n., holotype female (OSUC 368744) **192** Head and mesosoma, lateral view **193** Head and mesosoma, dorsal view **194** Head, anterior view **195** Metasoma, dorsal view. Morphbank^62^

Figures 192–195 Morphbank^62^

#### Description.

*Female*. Body length 5.85 mm (n=1).

Radicle color: same color as scape. Scape color: Yellowish. A4: longer than broad. A5: longer than broad. Antennal club: formed, segments compact.

Interantennal process: not elongate. Median longitudinal elevation in frontal depression: absent. Frontal depression: concave. Frontal depression sculpture: with 3 or more broadly interrupted transverse carinae. Submedian carina: strong, formed by a sharp raised carina. Submedian carina medially: with sharp peak. Concavity across dorsal part of frontal depression: absent. Depression extending ventrally from median ocellus: absent. Upper frons: not hood-like. Malar area near antennal foramen: without carina or expansion. Malar area at mouth corner: with radiating striae. Smooth strip along posterior side of malar sulcus: absent or not consistently broad. Middle genal carina: present. Direction of middle genal carina dorsally: parallel to eye margin. Major sculpture of gena anteriorly: umbilicate-foveate; rugose. Major sculpture of gena posteriorly: umbilicate-foveate; rugose. Microsculpture of gena anteroventrally: absent. Microsculpture of gena posteroventrally: absent. Median carina extending posteriorly from hyperoccipital carina: absent. Hyperoccipital carina: indicated by rugae. Lateral connection between hyperoccipital and occipital carinae: present as a weak elevation. Area between vertex and occipital carina: umbilicate-foveate. Occipital carina medially: divided into concave halves, meeting at median peak. Lateral corners of occipital carina: sharp and protruding.

Lateral pronotal area: without bulge projecting towards anterior pit. Epomial corner: strong. Netrion surface anteriorly: not inflexed. Mesoscutum anteriorly: steep. Mesoscutal median carina: present and complete. Longitudinal carina between median carina and notauli: absent. Major sculpture of medial mesoscutum anteriorly: umbilicate-foveate. Major sculpture of medial mesoscutum posteriorly: umbilicate-foveate. Microsculpture of medial mesoscutum anteriorly: granulate. Microsculpture of medial mesoscutum posteriorly: absent. Major sculpture of mesoscutellum: umbilicate-foveate. Microsculpture of mesoscutellum medially: absent. Microsculpture of mesoscutellum laterally: absent. Mesoscutellar apex: convex or straight. Setae along anterior limit of femoral depression: arising from rows of foveae. Number of carinae crossing speculum above femoral depression: 4. Number of carinae crossing femoral depression: more than 5. Mesepimeral sulcus pits: more than 5. Metascutellum dorsally: flat. Metascutellar sculpture dorsally: with scattered rugae. Median carina of metascutellum: absent or branched. Metascutellar setae: absent. Metascutellar apex: weakly emarginate. Metapleuron above ventral metapleural area: crossed by carinae. Metasomal depression setae: absent. Lateral propodeal carinae anteromedially: strongly diverging. Anterior areoles of metasomal depression: absent. Anterior longitudinal carinae in metasomal depression: absent. Lateral propodeal areas: separated medially. Postmarginal vein: absent. Fore wing apex: reaching middle of T4.

T1 midlobe: obscured by other raised sculpture. T1: with small rounded anterior bulge, not reaching metascutellum. T2: with straight longitudinal striae or rugae. T6: longer than broad. Apical flange of T6: exposed apically. Metasomal apex: rounded. Major sculpture of T6: umbilicate-punctate; longitudinally striate or rugose. Microsculpture of T6: granulate.

*Male*. Unknown.

#### Diagnosis.

Female: A5 broader than long. Submedian carina acute dorsally; frontal depression not crossed by carinae. Mesoscutellum without granulate sculpture. Metascutellum broad and rugose. Fore wings long enough to reach middle of T4. T1 midlobe with a long and broad anterior horn. T6 longer than broad, rounded apically.

#### Etymology.

Latin noun, genitive case, meaning “fan.”

#### Link to distribution map.

[http://hol.osu.edu/map-full.html?id=275510]

#### Material examined.

Holotype, female: **THAILAND**: Trang Prov., forest research center, Khao Chong Mountain, 07°33'02"N, 99°47'23"E, 75m, 21.I-26.I.2005, D. Lohman, OSUC 368744 (deposited in CNCI).

#### Comments.

*Oxyscelio flabelli* is unusual in having a broad, rugose metascutellum in combination with a mostly smooth frontal depression.

### Oxyscelio flavipennis

(Kieffer)

urn:lsid:zoobank.org:act:CE946CB9-4D90-4AE3-B34D-7A1C35F3D811

urn:lsid:biosci.ohio-state.edu:osuc_concepts:5017

http://species-id.net/wiki/Oxyscelio_flavipennis

**Figures 196–199.**
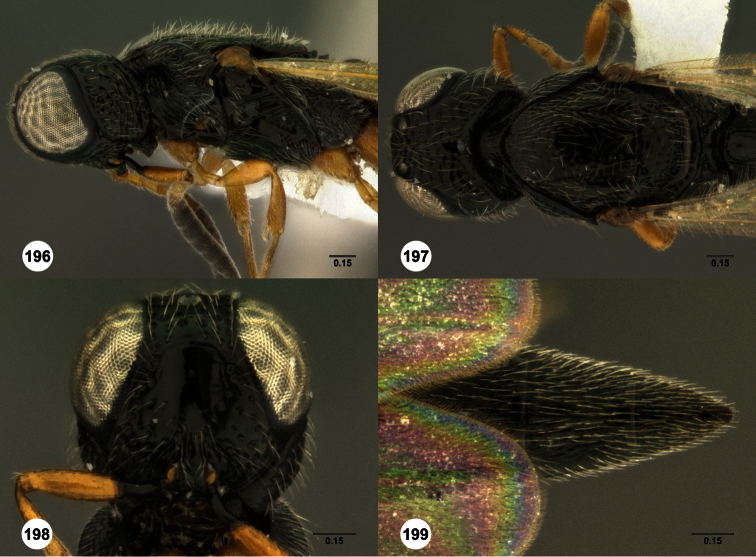
*Oxyscelio flavipennis* (Kieffer), female (OSUC 188474) **196** Head and mesosoma, lateral view **197** Head and mesosoma, dorsal view **198** Head, anterior view **199** Metasoma, dorsal view. Morphbank^63^

Figures 196–199 Morphbank^63^

Xenoteleia flavipennis Kieffer, 1913b: 390 (original description); Kieffer 1926: 427 (description).
Oxyscelio flavipennis (Kieffer): Dodd 1931: 75 (generic transfer); Masner 1976: 24 (type information).

#### Description.

*Female*. Body length 4.75–4.8 mm (n=2).

Radicle color: darker than scape. Scape color: Yellowish. A4: longer than broad. A5: broader than long. Antennal club: formed, segments compact.

Interantennal process: not elongate. Median longitudinal elevation in frontal depression: absent. Frontal depression: concave. Frontal depression sculpture: without transverse or oblique carinae below submedian carina. Submedian carina: weak, shallow and rounded or formed by ledge. Submedian carina medially: without peak. Concavity across dorsal part of frontal depression: absent. Depression extending ventrally from median ocellus: absent. Upper frons: not hood-like. Malar area near antennal foramen: without carina or expansion. Malar area at mouth corner: with radiating striae. Smooth strip along posterior side of malar sulcus: present, broad throughout its length. Middle genal carina: absent. Direction of middle genal carina dorsally: absent (replace with question mark). Major sculpture of gena anteriorly: umbilicate-foveate; rugose. Major sculpture of gena posteriorly: umbilicate-foveate; rugose. Microsculpture of gena anteroventrally: absent. Microsculpture of gena posteroventrally: absent. Median carina extending posteriorly from hyperoccipital carina: absent. Hyperoccipital carina: indicated by rugae. Lateral connection between hyperoccipital and occipital carinae: absent. Area between vertex and occipital carina: umbilicate-foveate. Occipital carina medially: absent. Lateral corners of occipital carina: sharp and protruding.

Lateral pronotal area: without bulge projecting towards anterior pit. Epomial corner: weak. Netrion surface anteriorly: inflexed. Mesoscutum anteriorly: not steep. Mesoscutal median carina: absent or weak and incomplete in places. Longitudinal carina between median carina and notauli: absent. Major sculpture of medial mesoscutum anteriorly: umbilicate-punctate. Major sculpture of medial mesoscutum posteriorly: umbilicate-foveate; umbilicate-punctate. Microsculpture of medial mesoscutum anteriorly: granulate. Microsculpture of medial mesoscutum posteriorly: absent. Major sculpture of mesoscutellum: umbilicate-foveate; umbilicate-punctate. Microsculpture of mesoscutellum medially: absent. Microsculpture of mesoscutellum laterally: absent. Mesoscutellar apex: convex or straight. Setae along anterior limit of femoral depression: arising from tiny pits.

Number of carinae crossing speculum above femoral depression: 2. Number of carinae crossing femoral depression: 3-5. Mesepimeral sulcus pits: more than 5. Metascutellum dorsally: flat. Metascutellar sculpture dorsally: with scattered rugae. Median carina of metascutellum: absent or branched. Metascutellar setae: absent. Metascutellar apex: weakly emarginate. Metapleuron above ventral metapleural area: foveate or rugose. Metasomal depression setae: absent. Lateral propodeal carinae anteromedially: weakly diverging. Anterior areoles of metasomal depression: absent. Anterior longitudinal carinae in metasomal depression: absent. Lateral propodeal areas: meeting for only a short distance medially. Postmarginal vein: present. Fore wing apex: reaching apex of T4; reaching middle of T5.

T1 midlobe: obscured by other raised sculpture. T1: with small rounded anterior bulge, not reaching metascutellum. T2: with straight longitudinal striae or rugae. T6: longer than broad. Apical flange of T6: exposed apically. Metasomal apex: rounded. Major sculpture of T6: umbilicate-punctate; longitudinally striate or rugose. Microsculpture of T6: granulate.

*Male*. Unknown.

#### Diagnosis.

Female: Netrion anteriorly concave. Metascutellum tiny and broad, hardly extending beyond anterior margin of propodeum. Fore wings not long enough to reach T5. *Oxyscelio flavipennis* is one of two species of its genus with a strongly anteriorly concave netrion. The other, *Oxyscelio cavinetrion*, has a downward-directed face as in *Oxyscelio flavipennis*, but has a very different metascutellum.

#### Link to distribution map.

[http://hol.osu.edu/map-full.html?id=5017]

#### Material examined.

Holotype, female, *Xenoteleia flavipennis*: **PHILIPPINES**: Laguna Prov., Los Baños, no date, Baker, USNM Type No. 70480 (deposited in USNM). *Other material*: **PHILIPPINES**: 1 female, OSUC 188474 (ROME).

### Oxyscelio flaviventris

Burks sp. n.

urn:lsid:zoobank.org:act:7263D2AD-AC60-46BC-BA71-E99B3868FEA8

urn:lsid:biosci.ohio-state.edu:osuc_concepts:275524

http://species-id.net/wiki/Oxyscelio_flaviventris

**Figures 200–203.**
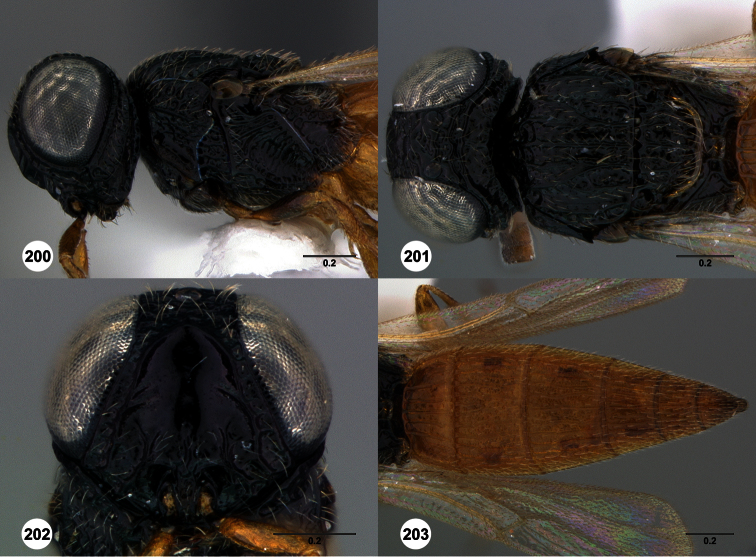
*Oxyscelio flaviventris* sp. n., holotype female (OSUC 369047) **200** Head and mesosoma, lateral view **201** Head and mesosoma, dorsal view **202** Head, anterior view **203** Metasoma, dorsal view. Morphbank^64^

Figures 200–203 Morphbank^64^

#### Description.

*Female*. Body length 2.65–2.9 mm (n=4).

Radicle color: darker than scape. Scape color: Yellowish. A4: broader than long. A5: broader than long. Antennal club: formed, segments compact.

Interantennal process: not elongate. Median longitudinal elevation in frontal depression: absent. Frontal depression: concave. Frontal depression sculpture: with 3 or more broadly interrupted transverse carinae. Submedian carina: strong, formed by a sharp raised carina. Submedian carina medially: with sharp peak. Concavity across dorsal part of frontal depression: absent. Depression extending ventrally from median ocellus: absent. Upper frons: not hood-like. Malar area near antennal foramen: without carina or expansion. Malar area at mouth corner: without striae. Smooth strip along posterior side of malar sulcus: absent or not consistently broad. Middle genal carina: present. Direction of middle genal carina dorsally: curving towards genal carina dorsally. Major sculpture of gena anteriorly: umbilicate-foveate; rugose. Major sculpture of gena posteriorly: umbilicate-foveate. Microsculpture of gena anteroventrally: absent. Microsculpture of gena posteroventrally: granulate. Median carina extending posteriorly from hyperoccipital carina: present. Hyperoccipital carina: indicated by rugae. Lateral connection between hyperoccipital and occipital carinae: absent. Area between vertex and occipital carina: umbilicate-foveate. Occipital carina medially: uniformly rounded. Lateral corners of occipital carina: not protruding.

Lateral pronotal area: without bulge projecting towards anterior pit. Epomial corner: strong. Netrion surface anteriorly: not inflexed. Mesoscutum anteriorly: steep. Mesoscutal median carina: present and complete. Longitudinal carina between median carina and notauli: present. Major sculpture of medial mesoscutum anteriorly: umbilicate-foveate; irregularly rugose. Major sculpture of medial mesoscutum posteriorly: umbilicate-punctate; irregularly rugose. Microsculpture of medial mesoscutum anteriorly: granulate. Microsculpture of medial mesoscutum posteriorly: absent. Major sculpture of mesoscutellum: umbilicate-foveate; longitudinally rugose. Microsculpture of mesoscutellum medially: absent. Microsculpture of mesoscutellum laterally: granulate. Mesoscutellar apex: convex or straight. Setae along anterior limit of femoral depression: arising from rows of foveae. Number of carinae crossing speculum above femoral depression: 3. Number of carinae crossing femoral depression: more than 5. Mesepimeral sulcus pits: more than 5. Metascutellum dorsally: concave. Metascutellar sculpture dorsally: smooth or with transverse carinae. Median carina of metascutellum: absent or branched. Metascutellar setae: absent. Metascutellar apex: weakly emarginate. Metapleuron above ventral metapleural area: smooth. Metasomal depression setae: absent. Lateral propodeal carinae anteromedially: strongly diverging. Anterior areoles of metasomal depression: absent. Anterior longitudinal carinae in metasomal depression: absent. Lateral propodeal areas: separated medially. Postmarginal vein: present. Fore wing apex: reaching apex of T5.

T1 midlobe: with 5 longitudinal carinae. T1: without anterior bulge. T2: with straight longitudinal striae or rugae. T6: longer than broad. Apical flange of T6: exposed apically. Metasomal apex: rounded. Major sculpture of T6: umbilicate-punctate; longitudinally striate or rugose. Microsculpture of T6: absent.

*Male*. Unknown.

#### Diagnosis.

Female: Hyperoccipital carina indicated by strong rugae; occipital carina complete. Mesosoma very tall and steep anteriorly, descending at nearly a right angle. Medial mesoscutum with at least 5 longitudinal carinae or sculptured elevations anteriorly, the lateral pairs merging posteriorly. Mesoscutellum with some granulate sculpture posterolaterally. Metascutellum tiny, not extending over base of T1. Fore wing long enough to reach middle of T5 or T6. T1 midlobe with 5 longitudinal carinae. T6 broader than long. *Oxyscelio flaviventris* is distinctive in the *limae*-group because of its small body and tiny metascutellum.

#### Etymology.

Latin noun, genitive case, meaning “yellow abdomen.”

#### Link to distribution map.

[http://hol.osu.edu/map-full.html?id=275524]

#### Associations.

collected near *Nilaparvatha* Distant: [Hemiptera: Auchenorrhyncha: Fulgoroidea: Delphacidae]; collected near *Oryza* Linnaeus: [Cyperales: Poaceae]

#### Material examined.

Holotype, female: **INDIA**: Karnataka St., Bangalore, 1.IX–9.IX.1987, pan trap, K. Ghorpade, OSUC 369047 (deposited in CNCI). *Paratypes*: **INDIA**: 3 females, OSUC 376576 (BMNH); OSUC 369045-369046 (CNCI).

### Oxyscelio florus

Kononova

urn:lsid:zoobank.org:act:6836FE9A-1218-4498-B609-A99CA8539908

urn:lsid:biosci.ohio-state.edu:osuc_concepts:243848

http://species-id.net/wiki/Oxyscelio_florus

**Figures 204–209.**
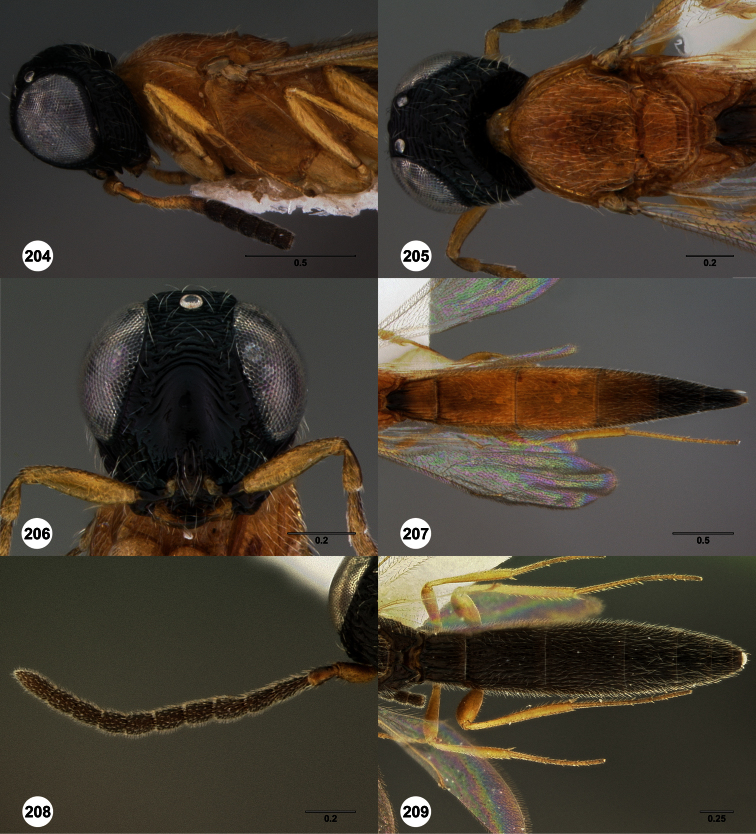
*Oxyscelio florus* Kononova, female (OSUC 368982) **204** Head and mesosoma, lateral view **205** Head and mesosoma, dorsal view **206** Head, anterior view **207** Metasoma, dorsal view. Male (OSUC 368984) **208** Antenna. Paratype male (OSUC 369002) **209** Metasoma, dorsal view. Morphbank^65^

Figures 204–209 Morphbank^65^

Oxyscelio florum Kononova: Kononova and Fursov 2007: 103 (description); Kononova and Fursov 2007: 62 (original description).
Oxyscelio florus Kononova: Kononova and Kozlov 2008: 190, 191 (description, keyed).

#### Description.

*Female*. Body length 4.25–4.65 mm (n=9).

Radicle color: darker than scape. Scape color: Yellowish. A4: longer than broad. A5: longer than broad. Antennal club: formed, segments compact.

Interantennal process: not elongate. Median longitudinal elevation in frontal depression: absent. Frontal depression: concave. Frontal depression sculpture: without transverse or oblique carinae below submedian carina. Submedian carina: indicated by multiple weak carinae. Submedian carina medially: without peak. Concavity across dorsal part of frontal depression: absent. Depression extending ventrally from median ocellus: absent. Upper frons: not hood-like. Malar area near antennal foramen: without carina or expansion. Malar area at mouth corner: with radiating striae. Smooth strip along posterior side of malar sulcus: absent or not consistently broad. Middle genal carina: present. Direction of middle genal carina dorsally: parallel to eye margin. Major sculpture of gena anteriorly: rugose; umbilicate-punctate. Major sculpture of gena posteriorly: rugose; umbilicate-punctate. Microsculpture of gena anteroventrally: absent. Microsculpture of gena posteroventrally: granulate. Median carina extending posteriorly from hyperoccipital carina: absent. Hyperoccipital carina: indicated by rugae. Lateral connection between hyperoccipital and occipital carinae: absent. Area between vertex and occipital carina: umbilicate-foveate. Occipital carina medially: uniformly rounded. Lateral corners of occipital carina: not protruding.

Lateral pronotal area: without bulge projecting towards anterior pit. Epomial corner: weak. Netrion surface anteriorly: not inflexed. Mesoscutum anteriorly: not steep. Mesoscutal median carina: present and complete. Longitudinal carina between median carina and notauli: absent. Major sculpture of medial mesoscutum anteriorly: umbilicate-foveate. Major sculpture of medial mesoscutum posteriorly: umbilicate-foveate. Microsculpture of medial mesoscutum anteriorly: granulate. Microsculpture of medial mesoscutum posteriorly: absent. Major sculpture of mesoscutellum: umbilicate-foveate. Microsculpture of mesoscutellum medially: absent. Microsculpture of mesoscutellum laterally: absent. Mesoscutellar apex: convex or straight. Setae along anterior limit of femoral depression: arising from tiny pits. Number of carinae crossing speculum above femoral depression: 4. Number of carinae crossing femoral depression: more than 5. Mesepimeral sulcus pits: more than 5. Metascutellum dorsally: flat. Metascutellar sculpture dorsally: with scattered rugae. Median carina of metascutellum: absent or branched. Metascutellar setae: absent. Metascutellar apex: convex or straight. Metapleuron above ventral metapleural area: foveate or rugose. Metasomal depression setae: absent. Lateral propodeal carinae anteromedially: strongly diverging. Anterior areoles of metasomal depression: absent. Anterior longitudinal carinae in metasomal depression: absent. Lateral propodeal areas: separated medially. Postmarginal vein: present. Fore wing apex: reaching apex of T4; reaching middle of T5.

T1 midlobe: obscured by other raised sculpture. T1: with long anterior bulge, reaching metascutellum. T2: with straight longitudinal striae or rugae. T6: longer than broad. Apical flange of T6: exposed apically. Metasomal apex: rounded. Major sculpture of T6: umbilicate-punctate; longitudinally striate or rugose. Microsculpture of T6: absent.

*Male*. Body length 3.85–4.15 mm (n=7). A5 tyloid: carina-like, not expanded. A11: longer than broad. Median tooth of frontal depression: absent. Median lobe of T1: with 6 longitudinal carinae. Metasomal apex: with acuminate lateral corners.

#### Diagnosis.

Both sexes: Upper frons with one or more extra carinae dorsal to submedian carina. Hyperoccipital carina indicated by rugae. Mesoscutellum without granulate sculpture. Mesofemoral depression crossed by more than 3 carinae below speculum. Female: Metascutellum subrectangular, with scattered weak rugae. T1 midlobe with long anterior bulge. T2 without sublateral depressions or curved striae. T6 longer than broad, tapering to a rounded apex. *Oxyscelio florus* differs from *Oxyscelio mollitia*, a similar Japanese species, in sculpture, metasomal length, and in having a much stronger T1 horn in females. Especially, the mesofemoral depression lacks a row of foveae along its anterior limit. *Oxyscelio florus* is very similar to the Taiwanese species *Oxyscelio dermatoglyphes* as well, especially in having extra carinae parallel to the submedian carina; these species differ in that *Oxyscelio dermatoglyphes* has no median carina on the mesoscutellum, only a very weak and indistinct one on the mesoscutum, and has a much shorter metasoma in females (fore wing long enough to reach T6 or apex of T5).

#### Etymology.

The Latin adjective florus has three forms, corresponding to gender agreement. Because *Oxyscelio* is a masculine genus based on initial species combination (Kieffer 1907) and agreement with *Scelio* Latreille, the correct form is *Oxyscelio florus*.

#### Link to distribution map.

[http://hol.osu.edu/map-full.html?id=243848]

#### Material examined.

Holotype, female, *Oxyscelio florum*: **JAPAN**: Aichi Pref., Kitashitara Co., Honshu Isl., Shitara Town, hill, Dando-Uradani Virgin Forest, 900m, 15.VIII.2004, V. Fursov, UASK 0108 (deposited in UASK). *Other material*: **JAPAN**: 11 females, 8 males, OSUC 368968-368974, 368981-368986, 368992-368993, 368995, 368997, 369002-369003 (CNCI).

#### Comments.

Coloration features mentioned by Kononova and Fursov (2007) do not hold constant in this species.

### Oxyscelio fodiens

Burks sp. n.

urn:lsid:zoobank.org:act:62A17BE6-2A4D-4C7C-85AE-547448203D46

urn:lsid:biosci.ohio-state.edu:osuc_concepts:275547

http://species-id.net/wiki/Oxyscelio_fodiens

**Figures 210–213.**
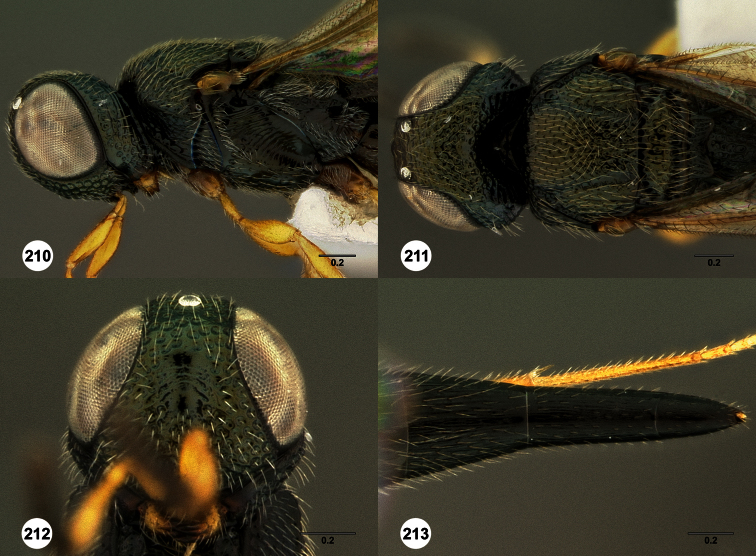
*Oxyscelio fodiens* sp. n., holotype female (OSUC 369042) **210** Head and mesosoma, lateral view **211** Head and mesosoma, dorsal view **212** Head, anterior view **213** Metasoma, dorsal view. Morphbank^66^

Figures 210–213 Morphbank^66^

#### Description.

*Female*. Body length 5.1 mm (n=1).

Radicle color: same color as scape. Scape color: Yellowish. A4: longer than broad. A5: longer than broad. Antennal club: formed, segments compact.

Interantennal process: not elongate. Median longitudinal elevation in frontal depression: absent. Frontal depression: concave. Frontal depression sculpture: with 3-5 complete transverse carinae. Submedian carina: weak, shallow and rounded or formed by ledge. Submedian carina medially: without peak. Concavity across dorsal part of frontal depression: absent. Depression extending ventrally from median ocellus: absent. Upper frons: not hood-like. Malar area near antennal foramen: without carina or expansion. Malar area at mouth corner: without striae. Smooth strip along posterior side of malar sulcus: present, broad throughout its length. Middle genal carina: present. Direction of middle genal carina dorsally: parallel to eye margin. Major sculpture of gena anteriorly: umbilicate-foveate. Major sculpture of gena posteriorly: umbilicate-foveate; rugose. Microsculpture of gena anteroventrally: absent. Microsculpture of gena posteroventrally: absent. Median carina extending posteriorly from hyperoccipital carina: absent. Hyperoccipital carina: not indicated medially. Lateral connection between hyperoccipital and occipital carinae: absent. Area between vertex and occipital carina: umbilicate-foveate. Occipital carina medially: absent. Lateral corners of occipital carina: sharp and protruding.

Lateral pronotal area: without bulge projecting towards anterior pit. Epomial corner: strong. Netrion surface anteriorly: not inflexed. Mesoscutum anteriorly: steep. Mesoscutal median carina: absent or weak and incomplete in places. Longitudinal carina between median carina and notauli: absent. Major sculpture of medial mesoscutum anteriorly: umbilicate-foveate; longitudinally rugose. Major sculpture of medial mesoscutum posteriorly: umbilicate-foveate. Microsculpture of medial mesoscutum anteriorly: granulate. Microsculpture of medial mesoscutum posteriorly: absent. Major sculpture of mesoscutellum: umbilicate-foveate. Microsculpture of mesoscutellum medially: punctate. Microsculpture of mesoscutellum laterally: punctate. Mesoscutellar apex: roundly concave. Setae along anterior limit of femoral depression: arising from rows of foveae. Number of carinae crossing speculum above femoral depression: 4. Number of carinae crossing femoral depression: more than 5. Mesepimeral sulcus pits: more than 5. Metascutellum dorsally: convex. Metascutellar sculpture dorsally: with scattered rugae. Median carina of metascutellum: absent or branched. Metascutellar setae: absent. Metascutellar apex: deeply emarginate. Metapleuron above ventral metapleural area: crossed by carinae. Metasomal depression setae: absent. Lateral propodeal carinae anteromedially: strongly diverging. Anterior areoles of metasomal depression: absent. Anterior longitudinal carinae in metasomal depression: absent. Lateral propodeal areas: separated medially. Postmarginal vein: absent. Fore wing apex: reaching middle of T4.

T1 midlobe: obscured by other raised sculpture. T1: with small rounded anterior bulge, not reaching metascutellum. T2: with long sublateral depressions. T6: longer than broad. Apical flange of T6: exposed apically. Metasomal apex: rounded. Major sculpture of T6: umbilicate-punctate. Microsculpture of T6: granulate.

*Male*. Unknown.

#### Diagnosis.

Female: Frontal depression crossed by a few carinae. Mesoscutellum without granulate areas. Mesoscutellum and metascutellum apically concave. Fore wings long enough to reach middle of T4. T1 with a strongly developed anterior horn that causes the longitudinal carinae to become broad and indistinct anteriorly. T2 with long sublateral depressions bordered medially by strong carinae. T5 and T6 elongate, nearly parallel-sided. *Oxyscelio fodiens* is similar to other members of the *fossarum*-group in having sublateral T2 depressions, but differs in having a broad metascutellum and nearly parallel-sided T5 and T6.

#### Etymology.

Latin participle, meaning “digging.” Does not change spelling under different genders. Refers to the concave sublateral depressions of T2 and the concave posterior margins of the mesoscutellum and metascutellum.

#### Link to distribution map.

[http://hol.osu.edu/map-full.html?id=275547]

#### Material examined.

Holotype, female: **MALAYSIA**: Sarawak St., lowland rainforest, Poring Hot Springs, VIII-1999, malaise trap, D. Quicke, OSUC 369042 (deposited in BMNH). *Paratype*: **INDONESIA**: 1 female, OSUC 247947 (ROME).

### Oxyscelio fossarum

Burks sp. n.

urn:lsid:zoobank.org:act:9921E46D-EFBC-4C1D-B7D9-42CBA0161EDD

urn:lsid:biosci.ohio-state.edu:osuc_concepts:275532

http://species-id.net/wiki/Oxyscelio_fossarum

**Figures 214–219.**
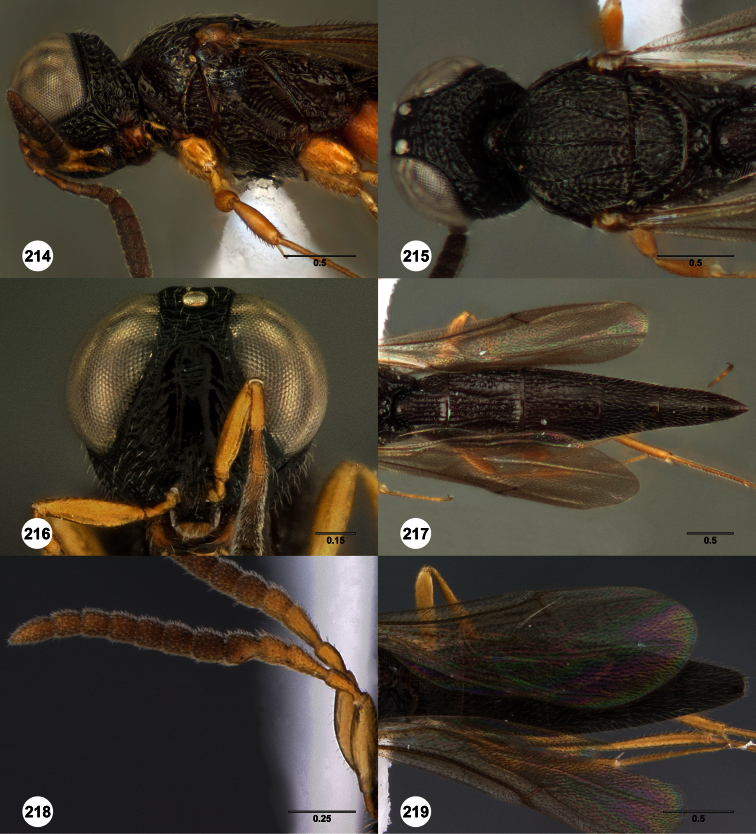
*Oxyscelio fossarum* sp. n., holotype female (OSUC 247931) **214** Head and mesosoma, lateral view **215** Head and mesosoma, dorsal view. Paratype female (OSUC 284829) **216** Head, anterior view. Paratype female (OSUC 257065) **217** Metasoma, dorsal view. Paratype male (OSUC 257076) **218** Antenna **219** Metasoma, dorsal view. Morphbank^67^

Figures 214–219 Morphbank^67^

#### Description.

*Female*. Body length 5.1–5.35 mm (n=7).

Radicle color: same color as scape. Scape color: Yellowish. A4: longer than broad. A5: longer than broad; as long as broad. Antennal club: formed, segments compact.

Interantennal process: not elongate. Median longitudinal elevation in frontal depression: absent. Frontal depression: concave. Frontal depression sculpture: with 3-5 complete transverse carinae. Submedian carina: strong, formed by a sharp raised carina. Submedian carina medially: without peak. Concavity across dorsal part of frontal depression: absent. Depression extending ventrally from median ocellus: absent. Upper frons: not hood-like. Malar area near antennal foramen: without carina or expansion. Malar area at mouth corner: without striae; with radiating striae. Smooth strip along posterior side of malar sulcus: present, broad throughout its length. Middle genal carina: present. Direction of middle genal carina dorsally: parallel to eye margin. Major sculpture of gena anteriorly: umbilicate-foveate. Major sculpture of gena posteriorly: umbilicate-foveate; rugose. Microsculpture of gena anteroventrally: absent. Microsculpture of gena posteroventrally: absent. Median carina extending posteriorly from hyperoccipital carina: absent. Hyperoccipital carina: indicated by rugae. Lateral connection between hyperoccipital and occipital carinae: present as a weak elevation. Area between vertex and occipital carina: umbilicate-foveate. Occipital carina medially: absent. Lateral corners of occipital carina: sharp and protruding.

Lateral pronotal area: without bulge projecting towards anterior pit. Epomial corner: weak. Netrion surface anteriorly: not inflexed. Mesoscutum anteriorly: not steep. Mesoscutal median carina: present and complete. Longitudinal carina between median carina and notauli: absent. Major sculpture of medial mesoscutum anteriorly: umbilicate-foveate. Major sculpture of medial mesoscutum posteriorly: umbilicate-foveate. Microsculpture of medial mesoscutum anteriorly: granulate. Microsculpture of medial mesoscutum posteriorly: absent; granulate. Major sculpture of mesoscutellum: umbilicate-foveate. Microsculpture of mesoscutellum medially: granulate. Microsculpture of mesoscutellum laterally: granulate. Mesoscutellar apex: convex or straight. Setae along anterior limit of femoral depression: arising from rows of foveae. Number of carinae crossing speculum above femoral depression: 2. Number of carinae crossing femoral depression: more than 5. Mesepimeral sulcus pits: more than 5. Metascutellum dorsally: flat. Metascutellar sculpture dorsally: with scattered rugae. Median carina of metascutellum: straight, unbranched carina present. Metascutellar setae: absent. Metascutellar apex: convex or straight; weakly emarginate. Metapleuron above ventral metapleural area: crossed by carinae. Metasomal depression setae: absent. Lateral propodeal carinae anteromedially: strongly diverging. Anterior areoles of metasomal depression: absent. Anterior longitudinal carinae in metasomal depression: absent. Lateral propodeal areas: separated medially. Postmarginal vein: present. Fore wing apex: reaching apex of T4.

T1 midlobe: obscured by other raised sculpture. T1: with small rounded anterior bulge, not reaching metascutellum. T2: with long sublateral depressions. T6: longer than broad. Apical flange of T6: not exposed apically. Metasomal apex: rounded. Major sculpture of T6: umbilicate-punctate. Microsculpture of T6: granulate.

*Male*. Unknown.

#### Diagnosis.

Female: Frontal depression crossed by a few carinae. Mesoscutellum strongly granulate. Metascutellum subrectangular, rugose. Fore wings long enough to reach middle of T4. T1 with a moderately developed anterior horn that causes the longitudinal carinae to become broad and indistinct anteriorly. T2 with long sublateral depressions bordered medially by strong carinae. T6 apically narrow but not sharply acuminate. *Oxyscelio fossarum*, from Borneo, is very similar to O. *fossularum* from Sumatra, but differs in metasomal length and surface sculpture. Males of *Oxyscelio fossarum* are unknown, but should differ from other of members of the *fossarum*-group from Borneo in having a granulate mesoscutellum.

#### Etymology.

Latin noun, genitive case, meaning “trenches.” Refers to the sublateral depressions of T2.

#### Link to distribution map.

[http://hol.osu.edu/map-full.html?id=275532]

#### Material examined.

Holotype, female: **INDONESIA**: Kalimantan Barat Prov., Cabang Panti Research Station, 1° rainforest / alluvial light gap, IIS 910122, Gunung Palung National Park, 01°15'S, 110°05'E, 100–400m, 15.VI–15.VIII.1991, malaise trap, Darling & Rosichon, OSUC 247931 (deposited in MBBJ). *Paratypes*: **INDONESIA**: 6 females, 7 males, OSUC 228698, 228705, 251423, 257076 (MBBJ); OSUC 228696, 247929, 247932, 257063, 257065, 284829 (OSUC); OSUC 247928, 247930, 251424 (ROME).

### Oxyscelio fossularum

Burks sp. n.

urn:lsid:zoobank.org:act:5F269D7F-F37A-4A2D-9678-2EB1D478072D

urn:lsid:biosci.ohio-state.edu:osuc_concepts:275507

http://species-id.net/wiki/Oxyscelio_fossularum

**Figures 220–224.**
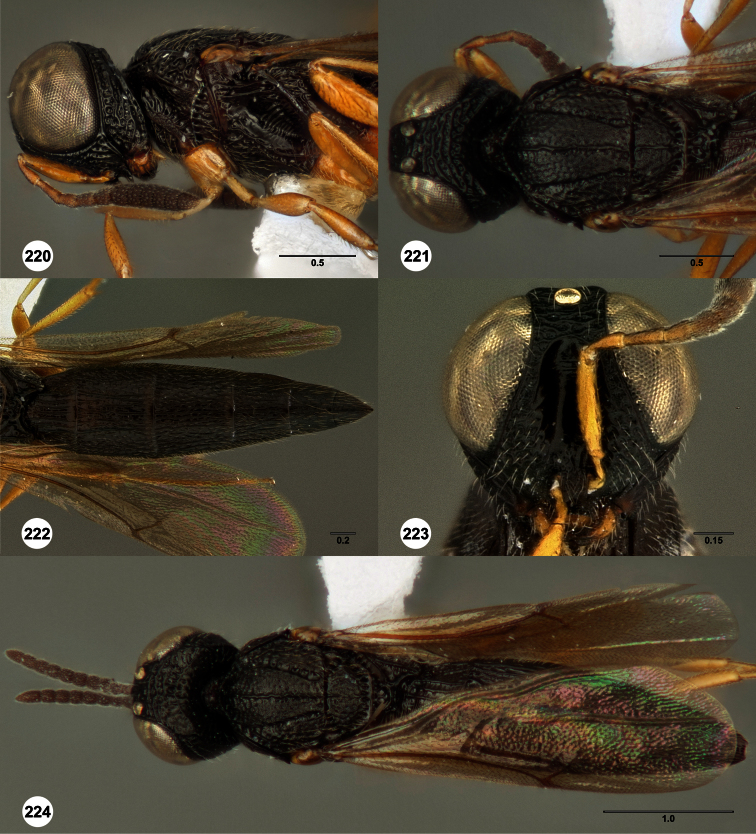
*Oxyscelio fossularum* sp. n., holotype female (OSUC 247975) **220** Head and mesosoma, lateral view **221** Head and mesosoma, dorsal view **222** Metasoma, dorsal view. Paratype female (OSUC 257037) **223** Head, anterior view. Paratype male (OSUC 247857) **224** Body, dorsal view. Morphbank^68^

Figures 220–224 Morphbank^68^

#### Description.

*Female*. Body length 4.55–4.75 mm (n=15).

Radicle color: same color as scape. Scape color: Yellowish. A4: longer than broad. A5: broader than long; as long as broad. Antennal club: formed, segments compact.

Interantennal process: not elongate. Median longitudinal elevation in frontal depression: absent. Frontal depression: concave. Frontal depression sculpture: with 3-5 complete transverse carinae. Submedian carina: strong, formed by a sharp raised carina. Submedian carina medially: without peak. Concavity across dorsal part of frontal depression: absent. Depression extending ventrally from median ocellus: absent. Upper frons: not hood-like. Malar area near antennal foramen: without carina or expansion. Malar area at mouth corner: without striae. Smooth strip along posterior side of malar sulcus: present, broad throughout its length. Middle genal carina: present. Direction of middle genal carina dorsally: parallel to eye margin. Major sculpture of gena anteriorly: umbilicate-foveate. Major sculpture of gena posteriorly: umbilicate-foveate; rugose. Microsculpture of gena anteroventrally: absent. Microsculpture of gena posteroventrally: absent. Median carina extending posteriorly from hyperoccipital carina: absent. Hyperoccipital carina: indicated by rugae. Lateral connection between hyperoccipital and occipital carinae: present as a weak elevation. Area between vertex and occipital carina: umbilicate-foveate; irregularly rugose. Occipital carina medially: sinuate, concave medial to corners, but without a median peak. Lateral corners of occipital carina: sharp and protruding.

Lateral pronotal area: without bulge projecting towards anterior pit. Epomial corner: weak. Netrion surface anteriorly: not inflexed. Mesoscutum anteriorly: not steep. Mesoscutal median carina: present and complete. Longitudinal carina between median carina and notauli: absent. Major sculpture of medial mesoscutum anteriorly: umbilicate-foveate. Major sculpture of medial mesoscutum posteriorly: umbilicate-punctate. Microsculpture of medial mesoscutum anteriorly: granulate. Microsculpture of medial mesoscutum posteriorly: granulate. Major sculpture of mesoscutellum: umbilicate-foveate. Microsculpture of mesoscutellum medially: granulate. Microsculpture of mesoscutellum laterally: granulate. Mesoscutellar apex: convex or straight. Setae along anterior limit of femoral depression: arising from rows of foveae. Number of carinae crossing speculum above femoral depression: 2. Number of carinae crossing femoral depression: 3-5. Mesepimeral sulcus pits: more than 5. Metascutellum dorsally: concave. Metascutellar sculpture dorsally: with scattered rugae. Median carina of metascutellum: absent or branched. Metascutellar setae: absent. Metascutellar apex: convex or straight; weakly emarginate. Metapleuron above ventral metapleural area: crossed by carinae; smooth. Metasomal depression setae: absent. Lateral propodeal carinae anteromedially: strongly diverging. Anterior areoles of metasomal depression: absent. Anterior longitudinal carinae in metasomal depression: absent. Lateral propodeal areas: separated medially. Postmarginal vein: present. Fore wing apex: reaching apex of T5.

T1 midlobe: with 6 or more longitudinal carinae; obscured by other raised sculpture. T1: with small rounded anterior bulge, not reaching metascutellum. T2: with long sublateral depressions. T6: longer than broad; as long as broad. Apical flange of T6: not exposed apically. Metasomal apex: rounded. Major sculpture of T6: umbilicate-punctate. Microsculpture of T6: granulate.

*Male*. Body length 4.35 mm (n=1). A5 tyloid: carina-like, not expanded. A11: longer than broad; as long as broad. Median tooth of frontal depression: absent. Median lobe of T1: with 5 longitudinal carinae. Metasomal apex: with rounded but projecting lobe-like corners.

#### Diagnosis.

Both sexes: Frontal depression crossed by a few carinae. Mesoscutellum strongly granulate. Metascutellum subrectangular, rugose. T2 with long sublateral depressions bordered medially by strong carinae. Female: T1 with a weakly developed anterior horn that has distinct longitudinal carinae. Fore wings long enough to reach middle of T5. T6 apically narrow but not sharply acuminate. Male: A11 longer than broad. T1 midlobe with 5 longitudinal carinae. T7 with rounded, protruding posterolateral corners.

#### Etymology.

Latin noun, genitive case, meaning “little trenches.” Refers to the sublateral metasomal depressions.

#### Link to distribution map.

[http://hol.osu.edu/map-full.html?id=275507]

#### Material examined.

Holotype, female: **INDONESIA**: Aceh Auto. Prov., Sumatra Isl., Ketambe Research Station, 1° rainforest / young forest / terrace 3 closed canopy, IIS 900011, Gunung Leuser National Park, 03°41'N, 97°39'E, 350m, II-1990, malaise trap, C. Darling, OSUC 247975 (deposited in MBBJ). *Paratypes*: **INDONESIA**: 15 females, 1 male, OSUC 464008 (CNCI); OSUC 247853, 257427, 361719 (MBBJ); OSUC 247977-247978, 267545-267546 (OSUC); OSUC 228712, 247857-247858, 247974, 257035, 257037, 257056, 257430 (ROME).

#### Comments.

*Oxyscelio fossularum* is the only member of its species group known from Sumatra.

### Oxyscelio foveatus

Kieffer

urn:lsid:zoobank.org:act:5815E7B9-F3CD-473F-8E7B-0AAD08235C99

urn:lsid:biosci.ohio-state.edu:osuc_concepts:5019

http://species-id.net/wiki/Oxyscelio_foveatus

**Figures 225–228.**
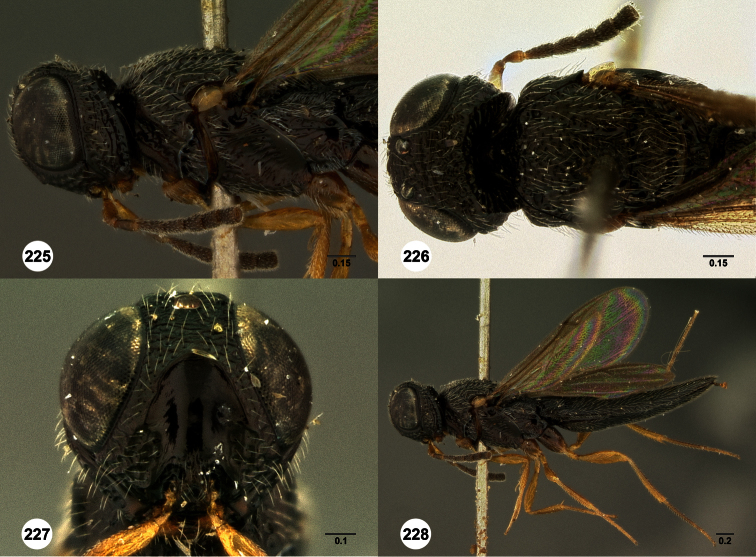
*Oxyscelio foveatus* Kieffer, neotype male (OSUC 436237) **225** Head and mesosoma, lateral view **226** Head and mesosoma, dorsal view **227** Head, anterior view **228** Body, lateral view. Morphbank^69^

Figures 225–228 Morphbank^69^

Oxyscelio foveatus Kieffer, 1907: 310 (original description); Kieffer 1926: 361 (description, keyed).
Chromoteleia (Oxyscelio) foveata (Kieffer): Kieffer 1910a: 313 (generic transfer, subgeneric assignment, keyed); Kieffer 1910b: 69 (generic transfer, emendation).

#### Description.

*Female*. Body length 3.8 mm (n=1).

Radicle color: same color as scape. Scape color: Yellowish. A4: longer than broad. A5: broader than long. Antennal club: formed, segments compact.

Interantennal process: not elongate. Median longitudinal elevation in frontal depression: absent. Frontal depression: concave. Frontal depression sculpture: with 3 or more broadly interrupted transverse carinae. Submedian carina: strong, formed by a sharp raised carina. Submedian carina medially: without peak. Concavity across dorsal part of frontal depression: absent. Depression extending ventrally from median ocellus: absent. Upper frons: not hood-like. Malar area near antennal foramen: with oblique tooth-like flange (facial nubbin). Malar area at mouth corner: with one carina. Smooth strip along posterior side of malar sulcus: present, broad throughout its length. Middle genal carina: present. Direction of middle genal carina dorsally: parallel to eye margin. Major sculpture of gena anteriorly: umbilicate-foveate; rugose. Major sculpture of gena posteriorly: umbilicate-foveate; rugose. Microsculpture of gena anteroventrally: absent. Microsculpture of gena posteroventrally: absent. Median carina extending posteriorly from hyperoccipital carina: absent. Hyperoccipital carina: indicated by rugae. Lateral connection between hyperoccipital and occipital carinae: absent. Area between vertex and occipital carina: umbilicate-foveate; irregularly rugose. Occipital carina medially: absent. Lateral corners of occipital carina: sharp and protruding.

Lateral pronotal area: without bulge projecting towards anterior pit. Epomial corner: strong. Netrion surface anteriorly: not inflexed. Mesoscutum anteriorly: not steep. Mesoscutal median carina: present and complete. Longitudinal carina between median carina and notauli: absent. Major sculpture of medial mesoscutum anteriorly: umbilicate-foveate. Major sculpture of medial mesoscutum posteriorly: umbilicate-foveate. Microsculpture of medial mesoscutum anteriorly: granulate. Microsculpture of medial mesoscutum posteriorly: absent. Major sculpture of mesoscutellum: umbilicate-foveate; irregularly rugose. Microsculpture of mesoscutellum medially: punctate. Microsculpture of mesoscutellum laterally: punctate. Mesoscutellar apex: convex or straight. Setae along anterior limit of femoral depression: arising from rows of foveae. Number of carinae crossing speculum above femoral depression: 2. Number of carinae crossing femoral depression: more than 5. Mesepimeral sulcus pits: 3-5. Metascutellum dorsally: flat. Metascutellar sculpture dorsally: with scattered rugae. Median carina of metascutellum: absent or branched. Metascutellar setae: absent. Metascutellar apex: convex or straight. Metapleuron above ventral metapleural area: crossed by carinae. Metasomal depression setae: absent. Lateral propodeal carinae anteromedially: strongly diverging. Anterior areoles of metasomal depression: absent. Anterior longitudinal carinae in metasomal depression: absent. Lateral propodeal areas: separated medially. Postmarginal vein: present. Fore wing apex: reaching middle of T5.

T1 midlobe: obscured by other raised sculpture. T1: with long anterior bulge, reaching metascutellum. T2: with straight longitudinal striae or rugae. T6: longer than broad. Apical flange of T6: exposed apically. Metasomal apex: rounded. Major sculpture of T6: umbilicate-punctate. Microsculpture of T6: granulate.

*Male*. Body length 3.9 mm (n=1). A5 tyloid: carina-like, not expanded. Median tooth of frontal depression: absent. Median lobe of T1: with 5 longitudinal carinae. Metasomal apex: with acuminate lateral corners.

#### Diagnosis.

Male: Face with oblique expanded flange between antennal foramen and eye. Gena with 1 strong middle carina. Metascutellum flat, with some rugae but only slightly broader than long. T1 midlobe with 5 longitudinal carinae. T7 with acuminate posterolateral corners.

#### Link to distribution map.

[http://hol.osu.edu/map-full.html?id=5019]

#### Material examined.

Neotype, male: **INDONESIA**: Jawa Tengah Prov., Java Isl., Semarang (Samarang), VII-1909, E. Jacobson, OSUC 436237 (deposited in RMNH). *Other material*: **INDONESIA**: 1 female, OSUC 448631 (RMNH).

#### Comments.

The type material of *Oxyscelio foveatus* Kieffer, collected by E. Jacobson from Semarang, Java, could not be found after an extensive search of collections known to house Kieffer type material. The neotype of *Oxyscelio foveatus* is presently designated to clarify the taxonomic status of the genus and species. It was selected because of its collector, collection locality and date and because it agrees with Kieffer’s remarks on metascutellar sculpture of the lost holotype. It is possible that this specimen was collected during the same event as the lost holotype.

### Oxyscelio genae

Burks sp. n.

urn:lsid:zoobank.org:act:CB11EBF5-6FC5-49AC-AC54-74F87BF099C3

urn:lsid:biosci.ohio-state.edu:osuc_concepts:305705

http://species-id.net/wiki/Oxyscelio_genae

**Figures 229–234.**
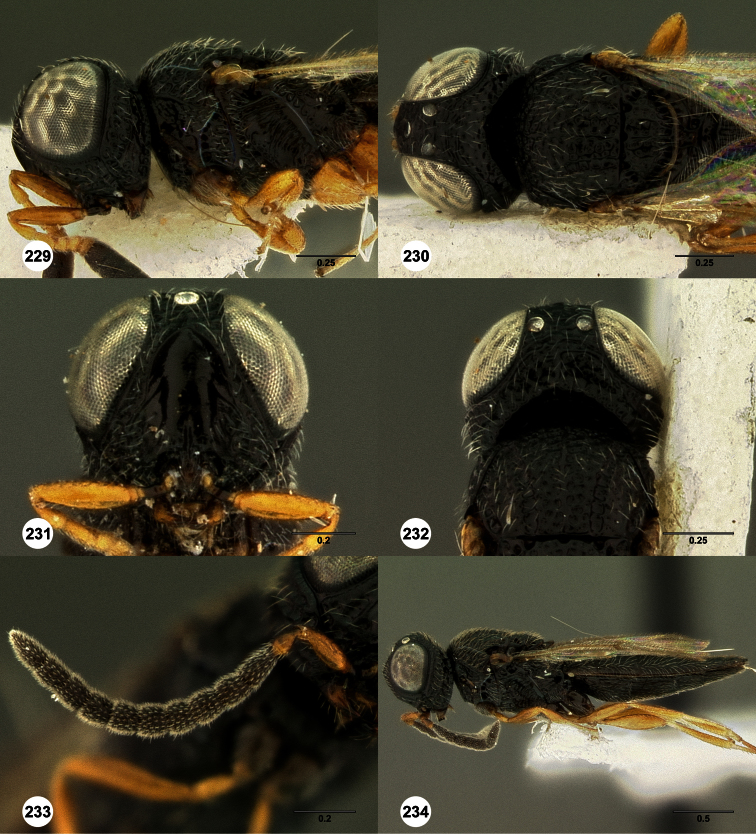
*Oxyscelio genae* sp. n., paratype female (OSUC 382052) **229** Head and mesosoma, lateral view **230** Head and mesosoma, dorsal view **231** Head, anterior view **232** Head, posterodorsal view. Paratype male (OSUC 369089) **233** Antenna **234** Body, lateral view. Morphbank^70^

Figures 229–234 Morphbank^70^

#### Description.

*Female*. Body length 2.85–3.05 mm (n=2).

Radicle color: darker than scape. Scape color: Yellowish. A4: broader than long. A5: broader than long. Antennal club: formed, segments compact.

Interantennal process: not elongate. Median longitudinal elevation in frontal depression: absent. Frontal depression: concave. Frontal depression sculpture: with 2 complete transverse carinae. Submedian carina: strong, formed by a sharp raised carina. Submedian carina medially: with sharp peak. Concavity across dorsal part of frontal depression: absent. Depression extending ventrally from median ocellus: absent. Upper frons: not hood-like. Malar area near antennal foramen: without carina or expansion. Malar area at mouth corner: with radiating striae. Smooth strip along posterior side of malar sulcus: absent or not consistently broad. Middle genal carina: present. Direction of middle genal carina dorsally: parallel to eye margin. Major sculpture of gena anteriorly: umbilicate-foveate. Major sculpture of gena posteriorly: umbilicate-foveate. Microsculpture of gena anteroventrally: absent. Microsculpture of gena posteroventrally: granulate. Median carina extending posteriorly from hyperoccipital carina: absent; present. Hyperoccipital carina: complete, continuous with anterior genal carina. Lateral connection between hyperoccipital and occipital carinae: present as a weak elevation. Area between vertex and occipital carina: umbilicate-foveate. Occipital carina medially: uniformly rounded. Lateral corners of occipital carina: not protruding.

Lateral pronotal area: without bulge projecting towards anterior pit. Epomial corner: strong. Netrion surface anteriorly: not inflexed. Mesoscutum anteriorly: steep. Mesoscutal median carina: present and complete. Longitudinal carina between median carina and notauli: absent. Major sculpture of medial mesoscutum anteriorly: umbilicate-foveate. Major sculpture of medial mesoscutum posteriorly: umbilicate-foveate. Microsculpture of medial mesoscutum anteriorly: granulate. Microsculpture of medial mesoscutum posteriorly: absent. Major sculpture of mesoscutellum: umbilicate-foveate. Microsculpture of mesoscutellum medially: absent. Microsculpture of mesoscutellum laterally: granulate. Mesoscutellar apex: convex or straight. Setae along anterior limit of femoral depression: arising from rows of foveae. Number of carinae crossing speculum above femoral depression: 2. Number of carinae crossing femoral depression: 3-5. Mesepimeral sulcus pits: more than 5. Metascutellum dorsally: concave. Metascutellar sculpture dorsally: smooth or with transverse carinae. Median carina of metascutellum: absent or branched. Metascutellar setae: absent. Metascutellar apex: weakly emarginate. Metapleuron above ventral metapleural area: crossed by carinae. Metasomal depression setae: absent. Lateral propodeal carinae anteromedially: weakly diverging. Anterior areoles of metasomal depression: absent. Anterior longitudinal carinae in metasomal depression: absent. Lateral propodeal areas: separated medially. Postmarginal vein: present. Fore wing apex: reaching apex of T5.

T1 midlobe: with 5 longitudinal carinae. T1: without anterior bulge. T2: with straight longitudinal striae or rugae. T6: broader than long. Apical flange of T6: not exposed apically. Metasomal apex: rounded. Major sculpture of T6: umbilicate-punctate. Microsculpture of T6: absent.

*Male*. Body length 3–3.2 mm (n=4). A5 tyloid: carina-like, not expanded. A11: longer than broad; as long as broad. Median tooth of frontal depression: absent. Median lobe of T1: with 4 longitudinal carinae. Metasomal apex: with acuminate lateral corners.

#### Diagnosis.

Both sexes: Middle genal carina very strong, subparallel with eye margin. Hyperoccipital carina indicated by an occasionally interrupted carina, area between it and occipital carina with a weak median carina. Mesoscutellum with some granulate sculpture laterally. Metascutellum tiny, concave dorsally, smooth aside from some transverse carinae. Female: A4, A5 broader than long. T1 midlobe with 5 longitudinal carinae. T6 rounded apically. Male: A11 slightly longer than broad. Occiput with very strong sculpture. T1 midlobe with 3 longitudinal carinae. T7 with sharp and protruding posterolateral corners. *Oxyscelio genae* is distinct from other members of the *crebritas*-group in having an almost completely outlined occipital depression with a median carina. It can also be recognized by the rough occipital sculpture in males, the tiny metascutellum, and very strong middle genal carina.

#### Etymology.

Latin noun in genitive case, meaning “cheek.” Refers to the strong middle genal carina.

#### Link to distribution map.

[http://hol.osu.edu/map-full.html?id=305705]

#### Material examined.

Holotype, female: **INDIA**: Tamil Nadu St., Coimbatore, 25.IX–1.X.1979, J. S. Noyes, OSUC 376570 (deposited in BMNH). *Paratypes*: (1 female, 4 males) **INDIA**: 1 female, 2 males, OSUC 376569, 376571 (BMNH); OSUC 382052 (OSUC). **SRI LANKA**: 2 males, OSUC 369089 (CNCI); OSUC 268100 (USNM).

### Oxyscelio granorum

Burks sp. n.

urn:lsid:zoobank.org:act:A531A356-5AB2-4314-A0A8-727D9BAC04F3

urn:lsid:biosci.ohio-state.edu:osuc_concepts:275482

http://species-id.net/wiki/Oxyscelio_granorum

**Figures 235–240.**
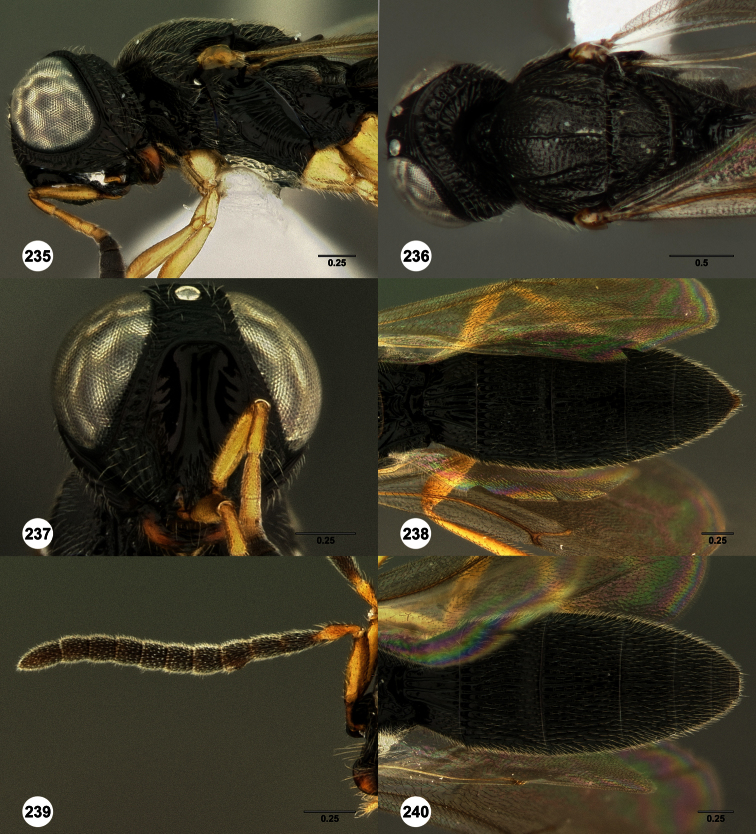
*Oxyscelio granorum* sp. n., paratype female (OSUC 335865) **235** Head and mesosoma, lateral view **236** Head and mesosoma, dorsal view. Paratype female (OSUC 335823) **237** Head, anterior view. Paratype female (OSUC 335868) **238** Metasoma, dorsal view. Paratype male (OSUC 368676) **239** Antenna. Paratype male (OSUC 335860) **240** Metasoma, dorsal view. Morphbank^71^

Figures 235–240 Morphbank^71^

#### Description.

*Female*. Body length 4–5.65 mm (n=20).

Radicle color: same color as scape. Scape color: Yellowish. A4: longer than broad; as long as broad. A5: broader than long. Antennal club: formed, segments compact.

Interantennal process: not elongate. Median longitudinal elevation in frontal depression: absent; present. Frontal depression: concave. Frontal depression sculpture: with 3 or more broadly interrupted transverse carinae. Submedian carina: strong, formed by a sharp raised carina. Submedian carina medially: without peak. Concavity across dorsal part of frontal depression: absent. Depression extending ventrally from median ocellus: absent. Upper frons: hood-like, protruding over pedicel when antenna at rest. Malar area near antennal foramen: without carina or expansion. Malar area at mouth corner: with radiating striae. Smooth strip along posterior side of malar sulcus: absent or not consistently broad. Middle genal carina: present. Direction of middle genal carina dorsally: parallel to eye margin. Major sculpture of gena anteriorly: umbilicate-foveate; rugose. Major sculpture of gena posteriorly: umbilicate-foveate; rugose. Microsculpture of gena anteroventrally: absent. Microsculpture of gena posteroventrally: absent. Median carina extending posteriorly from hyperoccipital carina: absent. Hyperoccipital carina: complete, continuous with anterior genal carina. Lateral connection between hyperoccipital and occipital carinae: absent. Area between vertex and occipital carina: irregularly rugose; crenulate. Occipital carina medially: uniformly rounded. Lateral corners of occipital carina: not protruding.

Lateral pronotal area: with slight bulge projecting anteriorly towards anterior pit. Epomial corner: weak. Netrion surface anteriorly: not inflexed. Mesoscutum anteriorly: not steep. Mesoscutal median carina: present and complete. Longitudinal carina between median carina and notauli: absent. Major sculpture of medial mesoscutum anteriorly: umbilicate-foveate. Major sculpture of medial mesoscutum posteriorly: umbilicate-foveate. Microsculpture of medial mesoscutum anteriorly: granulate. Microsculpture of medial mesoscutum posteriorly: granulate. Major sculpture of mesoscutellum: umbilicate-foveate. Microsculpture of mesoscutellum medially: granulate. Microsculpture of mesoscutellum laterally: granulate. Mesoscutellar apex: convex or straight. Setae along anterior limit of femoral depression: arising from rows of foveae. Number of carinae crossing speculum above femoral depression: 3. Number of carinae crossing femoral depression: more than 5. Mesepimeral sulcus pits: 3-5. Metascutellum dorsally: concave. Metascutellar sculpture dorsally: smooth or with transverse carinae. Median carina of metascutellum: absent or branched. Metascutellar setae: absent. Metascutellar apex: weakly emarginate. Metapleuron above ventral metapleural area: crossed by carinae. Metasomal depression setae: absent. Lateral propodeal carinae anteromedially: weakly diverging. Anterior areoles of metasomal depression: absent; one or more areoles present. Anterior longitudinal carinae in metasomal depression: absent. Lateral propodeal areas: separated medially. Postmarginal vein: present. Fore wing apex: reaching apex of T6.

T1 midlobe: with 4 longitudinal carinae. T1: without anterior bulge. T2: with straight longitudinal striae or rugae. T6: broader than long. Apical flange of T6: exposed apically. Metasomal apex: rounded. Major sculpture of T6: umbilicate-punctate. Microsculpture of T6: granulate.

*Male*. Body length 3.6–5.25 mm (n=20). A5 tyloid: carina-like, not expanded. A11: longer than broad; as long as broad. Median tooth of frontal depression: absent. Median lobe of T1: with 4 longitudinal carinae. Metasomal apex: with no distinct corners.

#### Diagnosis.

Both sexes: Frons without elevation between antennal foramen and eye. Frontal depression hood-like. Hyperoccipital carina present and sharp, continuous with anterior genal carina. Mesoscutellum with granulate sculpture. Metascutellum without dorsal setae. Propodeum without median carina; lateral propodeal carinae very narrowly separated anteriorly. Female: Fore wings not long enough to exceed metasomal apex. T1 midlobe with 4 longitudinal carinae. T6 rounded apically. Male: A11 as long or longer than broad. T1 midlobe with 4 longitudinal carinae. T7 with rounded posterolateral corners. *Oxyscelio granorum* is similar to *Oxyscelio intermedietas* in having extensive granulate sculpture, but is larger and is much more extensively granulate.

#### Etymology.

Latin noun, genitive case, meaning “grains,” referring to the dominant bodily surface sculpture.

#### Link to distribution map.

[http://hol.osu.edu/map-full.html?id=275482]

#### Material examined.

Holotype, female: **THAILAND**: Nakhon Si Thammarat Prov., road to Mhen Mt., 150m from Nern 499, T3101, Namtok Yong National Park, 08°16.959'N, 99°39.149'E, 499m, 13.VIII–20.VIII.2008, malaise trap, S. Samnaokan, OSUC 336125 (deposited in QSBG). *Paratypes*: (36 females, 38 males) **INDONESIA**: 6 females, 9 males, OSUC 368941-368942, 368954, 464005 (CNCI); OSUC 240930, 361273, 361718 (MBBJ); OSUC 248919, 257043 (OSUC); OSUC 240931, 247850, 247862, 247971, 352905, ROMEnt Spec. No. 112239 (ROME). **LAOS**: 4 males, OSUC 368872, 368874, 368888, 368906 (CNCI). **MALAYSIA**: 1 male, OSUC 376595 (BMNH). **THAILAND**: 27 females, 16 males, OSUC 335823, 335868, 352512 (BMNH); OSUC 368672, 368675-368676, 368724, 368763, 464046, 464050, 464061 (CNCI); OSUC 247872, 257399, 285204, 335864, 335866-335867, 335926, 336032, 336036, 336736, 352511 (OSUC); OSUC 237456-237457, 252035, 257396, 320371, 320374-320375, 320404-320405, 322078, 322086, 335860, 352484, 352513, 361356, 361375, 368493 (QSBG); UCRC ENT 41411 (UCRC); OSUC 335861, 335865, 335915 (WINC). **VIETNAM**: 3 females, 8 males, OSUC 369098, 369101 (CNCI); OSUC 277399-277401, 277517, 277704-277707, 281507 (RMNH).

### Oxyscelio granuli

Burks sp. n.

urn:lsid:zoobank.org:act:08EB7D7D-AE16-4601-BF1D-244B6BC09C77

urn:lsid:biosci.ohio-state.edu:osuc_concepts:305706

http://species-id.net/wiki/Oxyscelio_granuli

**Figures 241–244.**
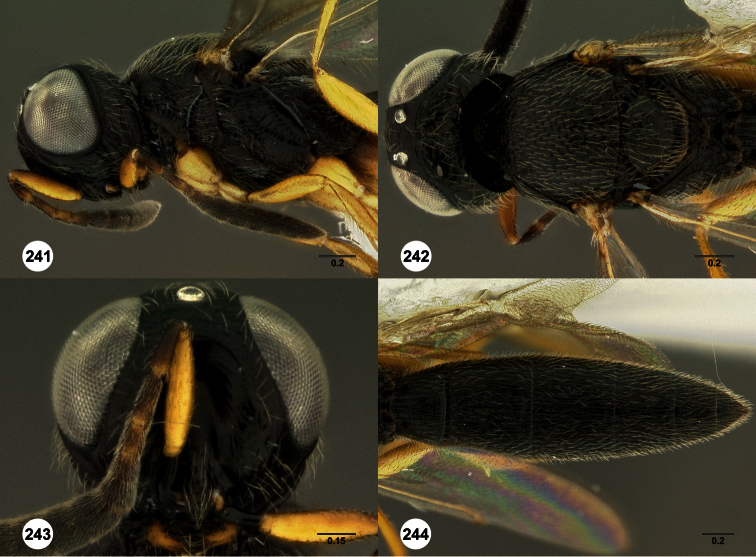
*Oxyscelio granuli* sp. n., holotype female (OSUC 277628) **241** Head and mesosoma, lateral view **242** Head and mesosoma, dorsal view **243** Head, anterior view **244** Metasoma, dorsal view. Morphbank^72^

Figures 241–244 Morphbank^72^

#### Description.

*Female*. Body length 4.15–4.25 mm (n=2).

Radicle color: darker than scape. Scape color: Yellowish. A4: longer than broad. A5: longer than broad. Antennal club: formed, segments compact.

Interantennal process: not elongate. Median longitudinal elevation in frontal depression: absent. Frontal depression: concave. Frontal depression sculpture: without transverse or oblique carinae below submedian carina. Submedian carina: strong, formed by a sharp raised carina. Submedian carina medially: without peak. Concavity across dorsal part of frontal depression: absent. Depression extending ventrally from median ocellus: absent. Upper frons: not hood-like. Malar area near antennal foramen: without carina or expansion. Malar area at mouth corner: with radiating striae. Smooth strip along posterior side of malar sulcus: absent or not consistently broad. Middle genal carina: present. Direction of middle genal carina dorsally: parallel to eye margin. Major sculpture of gena anteriorly: umbilicate-foveate. Major sculpture of gena posteriorly: absent. Microsculpture of gena anteroventrally: granulate. Microsculpture of gena posteroventrally: granulate. Median carina extending posteriorly from hyperoccipital carina: absent. Hyperoccipital carina: indicated by rugae. Lateral connection between hyperoccipital and occipital carinae: present as a weak elevation. Area between vertex and occipital carina: irregularly rugose. Occipital carina medially: uniformly rounded. Lateral corners of occipital carina: not protruding.

Lateral pronotal area: without bulge projecting towards anterior pit. Epomial corner: weak. Netrion surface anteriorly: not inflexed. Mesoscutum anteriorly: not steep. Mesoscutal median carina: present and complete. Longitudinal carina between median carina and notauli: absent. Major sculpture of medial mesoscutum anteriorly: umbilicate-foveate. Major sculpture of medial mesoscutum posteriorly: umbilicate-foveate. Microsculpture of medial mesoscutum anteriorly: granulate. Microsculpture of medial mesoscutum posteriorly: absent. Major sculpture of mesoscutellum: umbilicate-punctate. Microsculpture of mesoscutellum medially: granulate. Microsculpture of mesoscutellum laterally: granulate. Mesoscutellar apex: convex or straight. Setae along anterior limit of femoral depression: arising from rows of foveae. Number of carinae crossing speculum above femoral depression: 4. Number of carinae crossing femoral depression: more than 5. Mesepimeral sulcus pits: more than 5. Metascutellum dorsally: concave. Metascutellar sculpture dorsally: with scattered rugae. Median carina of metascutellum: absent or branched. Metascutellar setae: absent. Metascutellar apex: convex or straight; weakly emarginate. Metapleuron above ventral metapleural area: crossed by carinae. Metasomal depression setae: absent. Lateral propodeal carinae anteromedially: weakly diverging. Anterior areoles of metasomal depression: absent. Anterior longitudinal carinae in metasomal depression: absent. Lateral propodeal areas: separated medially. Postmarginal vein: present. Fore wing apex: reaching middle of T6.

T1 midlobe: with 5 longitudinal carinae. T1: without anterior bulge. T2: with straight longitudinal striae or rugae. T6: broader than long. Apical flange of T6: exposed apically. Metasomal apex: tapering to a sharp point. Major sculpture of T6: umbilicate-punctate; longitudinally striate or rugose. Microsculpture of T6: granulate.

*Male*. Unknown.

#### Diagnosis.

Female: A4, A5 longer than broad. Radicle darker than scape. Gena, mesoscutum, and mesoscutellum strongly granulate. Metascutellum small, concave dorsally. Lateral propodeal carinae subparallel anteriorly. T1 midlobe with 5 longitudinal carinae. T6 acuminate apically.

#### Etymology.

Latin noun, genitive case, meaning “granule.” Refers to the striking predominance of granulate sculpture on the mesoscutum and mesoscutellum.

#### Link to distribution map.

[http://hol.osu.edu/map-full.html?id=305706]

#### Material examined.

Holotype, female: **VIETNAM**: Lao Cai Prov., Tonkin Region, 15km W Sa Pa, Hoang Lien Son-Sa Pa Nature Reserve, ~1900m, 15.X–21.X.1999, malaise trap, C. v. Achterberg, OSUC 277628 (deposited in RMNH). *Paratype*: **VIETNAM**: 1 female, OSUC 277624 (RMNH).

#### Comments.

*Oxyscelio granuli* is unusual in having strongly granulate sculpture in combination with a tiny metascutellum and acuminate T6 in females. Males are unknown, but are expected to have similarly granulate sculpture.

### Oxyscelio greenacus

Burks sp. n.

urn:lsid:zoobank.org:act:84B2E059-7B8A-4942-9FE2-FF569AE811E1

urn:lsid:biosci.ohio-state.edu:osuc_concepts:305769

http://species-id.net/wiki/Oxyscelio_greenacus

**Figures 245–248.**
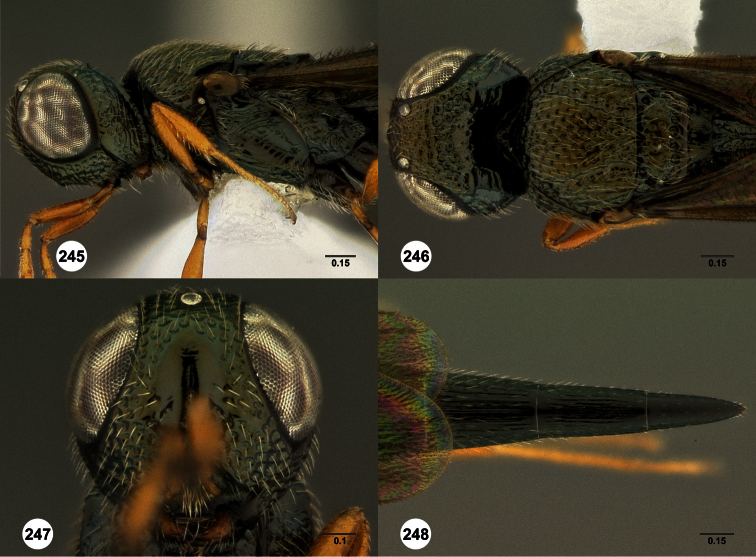
*Oxyscelio greenacus* sp. n., holotype female (OSUC 361339) **245** Head and mesosoma, lateral view **246** Head and mesosoma, dorsal view **247** Head, anterior view **248** Metasoma, dorsal view. Morphbank^73^

Figures 245–248 Morphbank^73^

#### Description.

*Female*. Body length 4.75 mm (n=1).

Radicle color: same color as scape. Scape color: Yellowish. A4: longer than broad. A5: longer than broad. Antennal club: formed, segments compact.

Interantennal process: not elongate. Median longitudinal elevation in frontal depression: absent. Frontal depression: concave. Frontal depression sculpture: without transverse or oblique carinae below submedian carina. Submedian carina: weak, shallow and rounded or formed by ledge. Submedian carina medially: without peak. Concavity across dorsal part of frontal depression: absent. Depression extending ventrally from median ocellus: absent. Upper frons: not hood-like. Malar area near antennal foramen: with oblique tooth-like flange (facial nubbin). Malar area at mouth corner: without striae. Smooth strip along posterior side of malar sulcus: absent or not consistently broad. Middle genal carina: present. Direction of middle genal carina dorsally: parallel to eye margin. Major sculpture of gena anteriorly: umbilicate-foveate; umbilicate-punctate. Major sculpture of gena posteriorly: rugose. Microsculpture of gena anteroventrally: absent. Microsculpture of gena posteroventrally: absent. Median carina extending posteriorly from hyperoccipital carina: absent. Hyperoccipital carina: not indicated medially. Lateral connection between hyperoccipital and occipital carinae: absent. Area between vertex and occipital carina: umbilicate-foveate. Occipital carina medially: absent. Lateral corners of occipital carina: sharp and protruding.

Lateral pronotal area: without bulge projecting towards anterior pit. Epomial corner: strong. Netrion surface anteriorly: not inflexed. Mesoscutum anteriorly: not steep. Mesoscutal median carina: absent or weak and incomplete in places. Longitudinal carina between median carina and notauli: absent. Major sculpture of medial mesoscutum anteriorly: umbilicate-foveate. Major sculpture of medial mesoscutum posteriorly: umbilicate-foveate. Microsculpture of medial mesoscutum anteriorly: granulate. Microsculpture of medial mesoscutum posteriorly: absent; granulate. Major sculpture of mesoscutellum: umbilicate-foveate. Microsculpture of mesoscutellum medially: granulate. Microsculpture of mesoscutellum laterally: granulate. Mesoscutellar apex: roundly concave. Setae along anterior limit of femoral depression: arising from rows of foveae. Number of carinae crossing speculum above femoral depression: 3. Number of carinae crossing femoral depression: more than 5. Mesepimeral sulcus pits: 3-5. Metascutellum dorsally: convex. Metascutellar sculpture dorsally: with scattered rugae. Median carina of metascutellum: absent or branched. Metascutellar setae: absent. Metascutellar apex: weakly emarginate. Metapleuron above ventral metapleural area: crossed by carinae. Metasomal depression setae: absent. Lateral propodeal carinae anteromedially: strongly diverging. Anterior areoles of metasomal depression: absent. Anterior longitudinal carinae in metasomal depression: absent. Lateral propodeal areas: separated medially. Postmarginal vein: absent. Fore wing apex: reaching middle of T4.

T1 midlobe: obscured by other raised sculpture. T1: with long anterior bulge, reaching metascutellum. T2: with straight longitudinal striae or rugae. T6: longer than broad. Apical flange of T6: exposed apically. Metasomal apex: rounded. Major sculpture of T6: umbilicate-punctate. Microsculpture of T6: granulate.

*Male*. Unknown.

#### Diagnosis.

Female: Antennal club formed. A4 longer than broad. Face with low, carina-like oblique expanded flange between antennal foramen and eye. Metascutellum convex, weakly sculptured and nearly truncate posteriorly. Fore wing long enough to reach base of T4. T1 horn elongate, reaching metascutellum. T5, T6 elongate and nearly parallel-sided. *Oxyscelio greenacus* is similar to some other species with elongate, nearly parallel-sided T5 and T6 in females, but differs in the chiefly smooth metascutellum.

#### Etymology.

Compound noun, Latin genitive case, of English word “green” and Latin noun acus, intended to mean “green needle.” Refers to the green body and elongate, nearly parallel-sided T5 and T6.

#### Link to distribution map.

[http://hol.osu.edu/map-full.html?id=305769]

#### Material examined.

Holotype, female: **THAILAND**: Nakhon Si Thammarat Prov., behind campground lavatory, T4675, Namtok Yong National Park, 08°10.434'N, 99°44.508'E, 95m, 23.II–2.III.2009, malaise trap, K. U-prai, OSUC 361339 (deposited in QSBG).

### Oxyscelio halmaherae

Burks sp. n.

urn:lsid:zoobank.org:act:4AF5B97A-1667-4682-ADBD-DB7C35D33B91

urn:lsid:biosci.ohio-state.edu:osuc_concepts:275549

http://species-id.net/wiki/Oxyscelio_halmaherae

**Figures 249–252.**
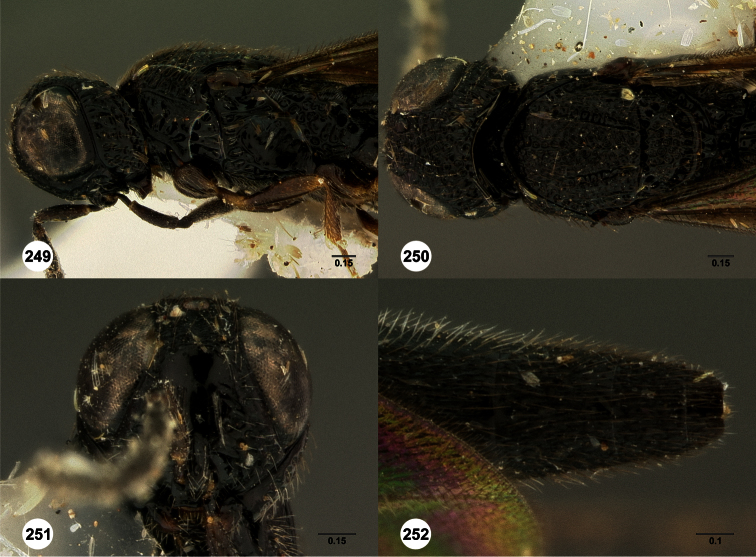
*Oxyscelio halmaherae* sp. n., holotype male (OSUC 268179) **249** Head and mesosoma, lateral view **250** Head and mesosoma, dorsal view **251** Head, anterior view **252** Metasomal apex, dorsal view. Morphbank^74^

Figures 249–252 Morphbank^74^

#### Description.

*Female*. Unknown.

*Male*. Body length 4.75 mm (n=1).

Radicle color: darker than scape. Scape color: Brown. A5 tyloid: carina-like, not expanded. A11: longer than broad.

Interantennal process: not elongate. Median longitudinal elevation in frontal depression: absent. Frontal depression: concave. Frontal depression sculpture: with 3 or more broadly interrupted transverse carinae. Submedian carina: strong, formed by a sharp raised carina. Submedian carina medially: without peak. Median tooth of frontal depression: absent. Concavity across dorsal part of frontal depression: absent. Depression extending ventrally from median ocellus: present. Upper frons: not hood-like. Malar area near antennal foramen: without carina or expansion. Malar area at mouth corner: with radiating striae. Smooth strip along posterior side of malar sulcus: absent or not consistently broad. Middle genal carina: absent. Direction of middle genal carina dorsally: absent (replace with question mark). Major sculpture of gena anteriorly: umbilicate-foveate. Major sculpture of gena posteriorly: umbilicate-foveate. Microsculpture of gena anteroventrally: absent. Microsculpture of gena posteroventrally: absent. Median carina extending posteriorly from hyperoccipital carina: absent. Hyperoccipital carina: indicated by rugae. Lateral connection between hyperoccipital and occipital carinae: absent. Area between vertex and occipital carina: umbilicate-foveate. Occipital carina medially: slightly convex, flatter medially than laterally. Lateral corners of occipital carina: sharp and protruding.

Lateral pronotal area: without bulge projecting towards anterior pit. Epomial corner: strong. Netrion surface anteriorly: not inflexed. Mesoscutum anteriorly: not steep. Mesoscutal median carina: present and complete. Longitudinal carina between median carina and notauli: absent. Major sculpture of medial mesoscutum anteriorly: umbilicate-foveate. Major sculpture of medial mesoscutum posteriorly: umbilicate-foveate. Microsculpture of medial mesoscutum anteriorly: granulate. Microsculpture of medial mesoscutum posteriorly: absent. Major sculpture of mesoscutellum: umbilicate-foveate. Microsculpture of mesoscutellum medially: absent. Microsculpture of mesoscutellum laterally: absent. Mesoscutellar apex: convex or straight. Setae along anterior limit of femoral depression: arising from rows of foveae. Number of carinae crossing speculum above femoral depression: 2. Number of carinae crossing femoral depression: 3-5. Mesepimeral sulcus pits: more than 5. Metascutellum dorsally: concave. Metascutellar sculpture dorsally: smooth or with transverse carinae. Median carina of metascutellum: absent or branched. Metascutellar setae: absent. Metascutellar apex: convex or straight. Metapleuron above ventral metapleural area: foveate or rugose. Metasomal depression setae: absent. Anterior areoles of metasomal depression: absent. Anterior longitudinal carinae in metasomal depression: absent. Lateral propodeal areas: separated medially. Postmarginal vein: present.

Median lobe of T1: with 4 longitudinal carinae. Metasomal apex: with rounded but projecting lobe-like corners.

#### Diagnosis.

Male: A5 tyloid not expanded. A11 longer than broad. Broad depression extending from median ocellus to submedian carina. Occipital carina with strong lateral corners. Mesoscutellum without granulate areas. Metascutellum tiny and concave, smooth dorsally. T1 midlobe with 4 longitudinal carinae. T7 with rounded lobes posterolaterally.

#### Etymology.

Latinisation of Halmahera, noun in genitive case.

#### Link to distribution map.

[http://hol.osu.edu/map-full.html?id=275549]

#### Material examined.

Holotype, male: **INDONESIA**: Maluku Utara Prov., Halmahera Isl., Pasirputih, 00°53'N, 127°41'E, 1.VI–14.VI.1981, A. C. Messer & P. M. Taylor, OSUC 268179 (deposited in USNM).

#### Comments.

*Oxyscelio halmaherae* is known from a single male specimen, described here because it is distinctive and apparently endemic to Halmahera.

### Oxyscelio intermedietas Oxyscelio intermedietas

Burks sp. n.

urn:lsid:zoobank.org:act:5902A8F2-DCD3-4FEE-9C14-AC346622EA2A

urn:lsid:biosci.ohio-state.edu:osuc_concepts:275481

http://species-id.net/wiki/Oxyscelio_intermedietas

**Figures 253–258.**
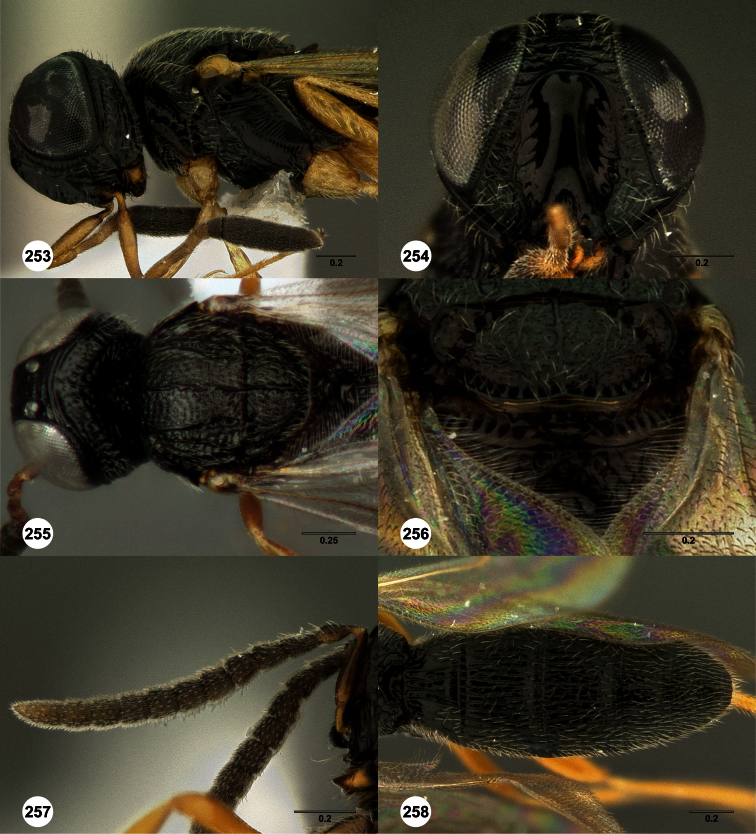
*Oxyscelio intermedietas* sp. n., paratype female (OSUC 322085) **253** Head and mesosoma, lateral view **254** Head, anterior view. Holotype female (OSUC 336091) **255** Head and mesosoma, dorsal view **256** Propodeum, posterodorsal view. Paratype male (OSUC 352446) **257** Antenna **258 **Metasoma, dorsal view. Morphbank^75^

Figures 253–258 Morphbank^75^

#### Description.

*Female*. Body length 2.85–4 mm (n=12).

Radicle color: same color as scape. Scape color: Yellowish. A4: broader than long. A5: broader than long. Antennal club: formed, segments compact.

Interantennal process: not elongate. Median longitudinal elevation in frontal depression: absent. Frontal depression: concave. Frontal depression sculpture: with 3 or more broadly interrupted transverse carinae. Submedian carina: strong, formed by a sharp raised carina. Submedian carina medially: without peak. Concavity across dorsal part of frontal depression: absent. Depression extending ventrally from median ocellus: absent. Upper frons: not hood-like. Malar area near antennal foramen: without carina or expansion. Malar area at mouth corner: with radiating striae. Smooth strip along posterior side of malar sulcus: absent or not consistently broad. Middle genal carina: present. Direction of middle genal carina dorsally: parallel to eye margin. Major sculpture of gena anteriorly: umbilicate-foveate. Major sculpture of gena posteriorly: umbilicate-foveate; rugose. Microsculpture of gena anteroventrally: absent. Microsculpture of gena posteroventrally: granulate. Median carina extending posteriorly from hyperoccipital carina: absent. Hyperoccipital carina: complete, continuous with anterior genal carina. Lateral connection between hyperoccipital and occipital carinae: absent. Area between vertex and occipital carina: irregularly rugose. Occipital carina medially: uniformly rounded; convex, with a sharp median peak. Lateral corners of occipital carina: not protruding.

Lateral pronotal area: with slight bulge projecting anteriorly towards anterior pit. Epomial corner: strong. Netrion surface anteriorly: not inflexed. Mesoscutum anteriorly: steep. Mesoscutal median carina: present and complete. Longitudinal carina between median carina and notauli: absent. Major sculpture of medial mesoscutum anteriorly: umbilicate-foveate. Major sculpture of medial mesoscutum posteriorly: umbilicate-foveate. Microsculpture of medial mesoscutum anteriorly: granulate. Microsculpture of medial mesoscutum posteriorly: granulate. Major sculpture of mesoscutellum: umbilicate-foveate; irregularly rugose. Microsculpture of mesoscutellum medially: absent; granulate. Microsculpture of mesoscutellum laterally: granulate. Mesoscutellar apex: convex or straight. Setae along anterior limit of femoral depression: arising from rows of foveae. Number of carinae crossing speculum above femoral depression: 3; 4. Number of carinae crossing femoral depression: more than 5. Mesepimeral sulcus pits: 3-5. Metascutellum dorsally: concave. Metascutellar sculpture dorsally: smooth or with transverse carinae. Median carina of metascutellum: absent or branched. Metascutellar setae: absent. Metascutellar apex: deeply emarginate; weakly emarginate. Metapleuron above ventral metapleural area: crossed by carinae. Metasomal depression setae: absent. Lateral propodeal carinae anteromedially: weakly diverging. Anterior areoles of metasomal depression: one or more areoles present. Anterior longitudinal carinae in metasomal depression: absent. Lateral propodeal areas: separated medially. Postmarginal vein: present. Fore wing apex: reaching beyond T6.

T1 midlobe: with 4 longitudinal carinae. T1: without anterior bulge. T2: with straight longitudinal striae or rugae. T6: broader than long. Apical flange of T6: exposed apically. Metasomal apex: rounded. Major sculpture of T6: umbilicate-punctate. Microsculpture of T6: absent.

*Male*.Body length 2.7–3.95 mm (n=20). A5 tyloid: carina-like, not expanded. A11: broader than long; as long as broad. Median tooth of frontal depression: absent. Median lobe of T1: with 4 longitudinal carinae. Metasomal apex: with no distinct corners.

#### Diagnosis.

Both sexes: Frons without elevation between antennal foramen and eye. Frontal depression shallow, not hood-like. Hyperoccipital carina present and sharp, continuous with anterior genal carina. Mesoscutellum with granulate sculpture. Metascutellum without dorsal setae. Propodeum without median carina; lateral propodeal carinae very narrowly separated anteriorly. Female: Fore wings long enough to exceed metasomal apex. T1 midlobe with 4 longitudinal carinae. T6 rounded apically. Male: A11 as broad or broader than long. T1 midlobe with 4 longitudinal carinae. T7 with rounded posterolateral corners.

#### Etymology.

Latin noun, genitive case, meaning “that which is between.” Refers to its similarity to several other species in the *cuculli*-group.

#### Link to distribution map.

[http://hol.osu.edu/map-full.html?id=275481]

#### Material examined.

Holotype, female: **THAILAND**: Kamphaeng Phet Prov., Chong Yen Mt., T2812, Mae Wong National Park, 16°05.968'N, 99°06.472'E, 1306m, 3.IX–10.IX.2007, malaise trap, C. Piluk & A. Inpuang, OSUC 336091 (deposited in QSBG). *Paratypes*: (14 females, 55 males) **LAOS**: 4 males, OSUC 368883-368884, 368887, 368890 (CNCI). **NEPAL**: 4 females, 3 males, OSUC 369148, 369158, 369161-369165 (CNCI). **THAILAND**: 7 females, 47 males, OSUC 322084, 352452, 352457 (BMNH); OSUC 368693, 368748, 462828, 464012-464013 (CNCI); OSUC 247628, 247632, 335092, 335857, 336010-336013, 336701, 352461-352469 (OSUC); OSUC 247603, 247916, 285237, 322082, 322085, 336705, 352446-352447, 352449, 352451, 352454, 352458, 352460, 361188-361189, 361191-361192, 361194-361199, 361205, 361207, 361350 (QSBG); OSUC 247602, 247626 (WINC). **VIETNAM**: 3 females, 1 male, OSUC 369107-369108 (CNCI); OSUC 277676, 281609 (RMNH).

#### Comments.

*Oxyscelio intermedietas* seems to be a small-bodied relative of *Oxyscelio granorum*. It otherwise strongly resembles several other species nearer to its own size, including *Oxyscelio cuculli*. It differs from these species in several subtle ways, including surface sculpture, shallowness of the frontal depression, and the very narrow anterior separation of the lateral propodeal carinae. While the possibility remains that *Oxyscelio intermedietas* is just a small form of *Oxyscelio granorum*, these features proved convincing enough to allow recognition of it as a separate species.

### Oxyscelio jaune

Burks sp. n.

urn:lsid:zoobank.org:act:B957AAA8-C08A-48C5-9065-843936BFD8C5

urn:lsid:biosci.ohio-state.edu:osuc_concepts:275513

http://species-id.net/wiki/Oxyscelio_jaune

**Figures 259–262.**
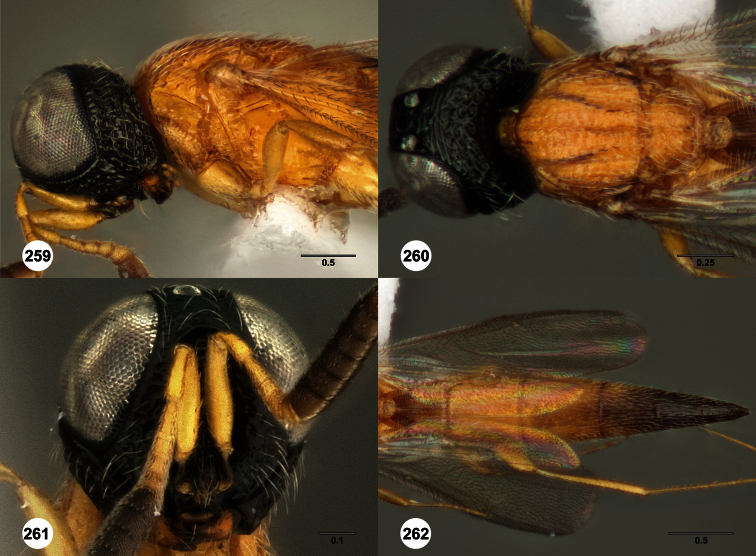
*Oxyscelio jaune* sp. n., holotype female (OSUC 352523) **259** Head and mesosoma, lateral view **260** Head and mesosoma, dorsal view **261** Head, anterior view **262** Metasoma, dorsal view. Morphbank^76^

Figures 259–262 Morphbank^76^

#### Description.

*Female*. Body length 3.65–3.8 mm (n=2).

Radicle color: darker than scape. Scape color: Yellowish. A4: broader than long. A5: broader than long. Antennal club: formed, segments compact.

Interantennal process: not elongate. Median longitudinal elevation in frontal depression: absent. Frontal depression: concave. Frontal depression sculpture: with 3-5 complete transverse carinae. Submedian carina: strong, formed by a sharp raised carina. Submedian carina medially: without peak. Concavity across dorsal part of frontal depression: absent. Depression extending ventrally from median ocellus: absent. Upper frons: not hood-like. Malar area near antennal foramen: without carina or expansion. Malar area at mouth corner: without striae. Smooth strip along posterior side of malar sulcus: absent or not consistently broad. Middle genal carina: present. Direction of middle genal carina dorsally: parallel to eye margin. Major sculpture of gena anteriorly: umbilicate-foveate. Major sculpture of gena posteriorly: umbilicate-foveate; rugose. Microsculpture of gena anteroventrally: absent. Microsculpture of gena posteroventrally: absent. Median carina extending posteriorly from hyperoccipital carina: absent. Hyperoccipital carina: not indicated medially. Lateral connection between hyperoccipital and occipital carinae: absent. Area between vertex and occipital carina: umbilicate-foveate. Occipital carina medially: absent. Lateral corners of occipital carina: not protruding.

Lateral pronotal area: without bulge projecting towards anterior pit. Epomial corner: strong. Netrion surface anteriorly: not inflexed. Mesoscutum anteriorly: not steep. Mesoscutal median carina: present and complete. Longitudinal carina between median carina and notauli: absent. Major sculpture of medial mesoscutum anteriorly: umbilicate-foveate. Major sculpture of medial mesoscutum posteriorly: umbilicate-foveate. Microsculpture of medial mesoscutum anteriorly: absent. Microsculpture of medial mesoscutum posteriorly: absent. Major sculpture of mesoscutellum: umbilicate-foveate. Microsculpture of mesoscutellum medially: absent. Microsculpture of mesoscutellum laterally: absent. Mesoscutellar apex: convex or straight. Setae along anterior limit of femoral depression: arising from rows of foveae. Number of carinae crossing speculum above femoral depression: 2. Number of carinae crossing femoral depression: 3-5. Mesepimeral sulcus pits: 3-5. Metascutellum dorsally: flat. Metascutellar sculpture dorsally: with scattered rugae. Median carina of metascutellum: absent or branched. Metascutellar setae: absent. Metascutellar apex: convex or straight. Metapleuron above ventral metapleural area: crossed by carinae. Metasomal depression setae: absent. Lateral propodeal carinae anteromedially: strongly diverging. Anterior areoles of metasomal depression: absent. Anterior longitudinal carinae in metasomal depression: absent. Lateral propodeal areas: separated medially. Postmarginal vein: present. Fore wing apex: reaching apex of T4.

T1 midlobe: obscured by other raised sculpture. T1: with long anterior bulge, reaching metascutellum. T2: with straight longitudinal striae or rugae. T6: longer than broad. Apical flange of T6: exposed apically. Metasomal apex: rounded. Major sculpture of T6: umbilicate-punctate. Microsculpture of T6: granulate.

*Male*. Unknown.

#### Diagnosis.

Female: Upper frons without additional carinae near the strong submedian carina. Hyperoccipital carina indicated by rugae. Mesoscutellum without granulate sculpture. Mesofemoral depression crossed by 3 carinae below speculum. Metascutellum subrectangular, with scattered weak rugae. T1 midlobe with long anterior bulge. T2 without sublateral depressions or curved striae. T6 longer than broad, tapering to a rounded apex. *Oxyscelio jaune* is similar to *Oxyscelio longiventris* and *Oxyscelio regionis*, in that they have a dark antennal radicle, long body, and very strong T1 horn in females. The color of the holotype is distinctive but may not be constant within the species, meaning that the small number of carinae (3) crossing the femoral depression is the best means of distinguishing it from *Oxyscelio longiventris* and *Oxyscelio regionis*.

#### Etymology.

French word meaning “yellow,” does not change spelling under different genders.

#### Link to distribution map.

[http://hol.osu.edu/map-full.html?id=275513]

#### Material examined.

Holotype, female: **THAILAND**: Surat Thani Prov., Klong Mog Unit, T3918, Khao Sok National Park, 08°53.725'N, 98°39.025'E, 87m, 13.II-20.I.2009, malaise trap, Pongphan, OSUC 352523 (deposited in QSBG).*Paratype*: **MALAYSIA**: 1 female, OSUC 442270 (UQIC).

### Oxyscelio jugi

Burks sp. n.

urn:lsid:zoobank.org:act:2D8F217A-8522-4F42-B41C-9038D798B6E7

urn:lsid:biosci.ohio-state.edu:osuc_concepts:275571

http://species-id.net/wiki/Oxyscelio_jugi

**Figures 263–268.**
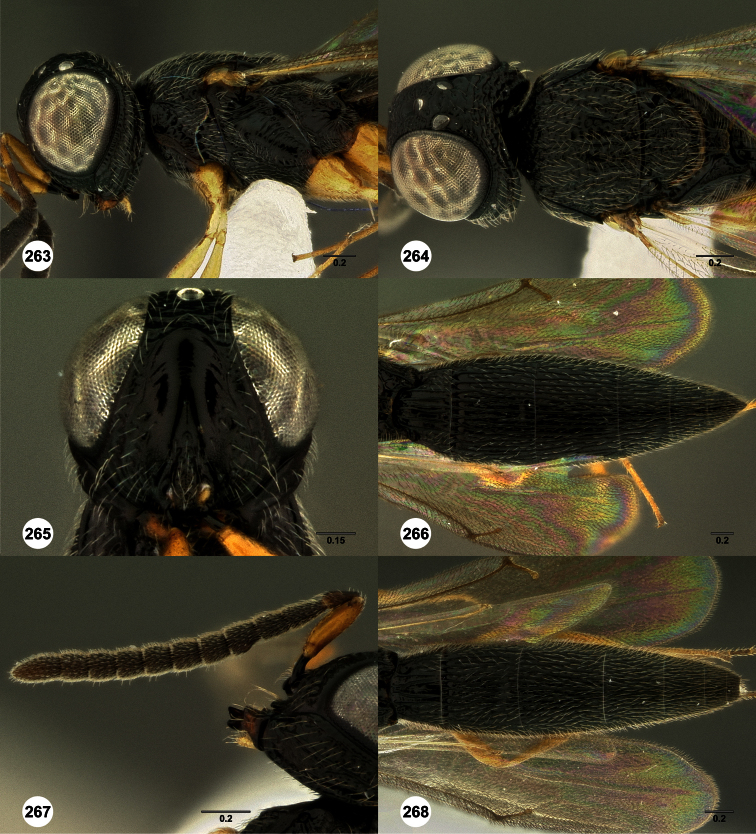
*Oxyscelio jugi* sp. n., holotype female (OSUC 369039) **263** Head and mesosoma, lateral view **264** Head and mesosoma, dorsal view. Paratype female (OSUC 322119) **265** Head, anterior view **266** Metasoma, dorsal view. Paratype male (OSUC 352478) **267** Antenna **268** Metasoma, dorsal view. Morphbank^77^

Figures 263–268 Morphbank^77^

#### Description.

*Female*. Body length 3.55–4.5 mm (n=5).

Radicle color: darker than scape. Scape color: Yellowish. A4: longer than broad; as long as broad. A5: broader than long. Antennal club: formed, segments compact.

Interantennal process: not elongate. Median longitudinal elevation in frontal depression: absent. Frontal depression: concave. Frontal depression sculpture: with 3 or more broadly interrupted transverse carinae. Submedian carina: strong, formed by a sharp raised carina. Submedian carina medially: without peak; with sharp peak. Concavity across dorsal part of frontal depression: absent. Depression extending ventrally from median ocellus: absent. Upper frons: not hood-like. Malar area near antennal foramen: without carina or expansion. Malar area at mouth corner: with radiating striae. Smooth strip along posterior side of malar sulcus: absent or not consistently broad. Middle genal carina: present. Direction of middle genal carina dorsally: parallel to eye margin. Major sculpture of gena anteriorly: umbilicate-foveate; rugose. Major sculpture of gena posteriorly: umbilicate-foveate; rugose. Microsculpture of gena anteroventrally: absent. Microsculpture of gena posteroventrally: absent. Median carina extending posteriorly from hyperoccipital carina: absent. Hyperoccipital carina: indicated by rugae. Lateral connection between hyperoccipital and occipital carinae: absent. Area between vertex and occipital carina: umbilicate-foveate. Occipital carina medially: absent. Lateral corners of occipital carina: not protruding.

Lateral pronotal area: without bulge projecting towards anterior pit. Epomial corner: strong. Netrion surface anteriorly: not inflexed. Mesoscutum anteriorly: not steep. Mesoscutal median carina: present and complete. Longitudinal carina between median carina and notauli: present. Major sculpture of medial mesoscutum anteriorly: umbilicate-foveate. Major sculpture of medial mesoscutum posteriorly: umbilicate-foveate. Microsculpture of medial mesoscutum anteriorly: granulate. Microsculpture of medial mesoscutum posteriorly: absent. Major sculpture of mesoscutellum: umbilicate-foveate. Microsculpture of mesoscutellum medially: absent. Microsculpture of mesoscutellum laterally: granulate. Mesoscutellar apex: convex or straight. Setae along anterior limit of femoral depression: arising from rows of foveae. Number of carinae crossing speculum above femoral depression: 2. Number of carinae crossing femoral depression: more than 5. Mesepimeral sulcus pits: 3-5. Metascutellum dorsally: concave. Metascutellar sculpture dorsally: smooth or with transverse carinae. Median carina of metascutellum: absent or branched. Metascutellar setae: absent. Metascutellar apex: weakly emarginate. Metapleuron above ventral metapleural area: crossed by carinae. Metasomal depression setae: absent. Lateral propodeal carinae anteromedially: strongly diverging. Anterior areoles of metasomal depression: absent. Anterior longitudinal carinae in metasomal depression: absent. Lateral propodeal areas: separated medially. Postmarginal vein: present. Fore wing apex: reaching middle of T5; reaching apex of T5; reaching apex of T6; reaching middle of T6.

T1 midlobe: with 5 longitudinal carinae. T1: without anterior bulge. T2: with straight longitudinal striae or rugae. T6: broader than long; longer than broad; as long as broad. Apical flange of T6: not exposed apically. Metasomal apex: tapering to a sharp point. Major sculpture of T6: umbilicate-punctate; longitudinally striate or rugose. Microsculpture of T6: absent.

*Male*. Body length 3.5–5.05 mm (n=20). A5 tyloid: carina-like, not expanded. A11: longer than broad. Median tooth of frontal depression: absent. Median lobe of T1: with 4 longitudinal carinae. Metasomal apex: with acuminate lateral corners.

#### Diagnosis.

Both sexes: Middle genal carina subparallel with eye margin. Hyperoccipital carina indicated by rugae. Mesoscutellum with granulate sculpture laterally but not medially. Metascutellum concave dorsally, smooth aside from some transverse carinae. Female: A5 broader than long. T1 midlobe with 5 longitudinal carinae. T6 acuminate apically. Male: A11 longer than broad. T1 midlobe with 4 longitudinal carinae. T7 with short, sharp and protruding posterolateral corners. *Oxyscelio jugi* can be distinguished from similar species by its strong mesoscutal sculpture and laterally granulate mesoscutellum. In males, the medial pair of longitudinal carinae of the T1 midlobe are usually curved to closely approach one another anteriorly.

#### Etymology.

Latin noun, genitive case, meaning “ridge.” Refers to the extra longitudinal carinae found on the mesoscutum.

#### Link to distribution map.

[http://hol.osu.edu/map-full.html?id=275571]

#### Material examined.

Holotype, female: **MALAYSIA**: Pahang St., Genting Tea Estate, 2000ft, VII-1985 - VIII-1985, malaise trap, W. Bundenberg, OSUC 369039 (deposited in CNCI). *Paratypes*: (4 females, 64 males) **INDONESIA**: 1 male, ROMEnt Spec. No. 112235 (ROME). **LAOS**: 11 males, OSUC 368850-368853, 368855-368856, 368862-368864, 368877, 368897 (CNCI). **MALAYSIA**: 1 female, 7 males, OSUC 369010, 369017, 369019, 369026, 369030, 369033, 369037-369038 (CNCI). **THAILAND**: 3 females, 43 males, OSUC 368641-368649, 368651-368671, 368753, 464040 (CNCI); OSUC 247618, 247923, 322119, 336700, 352477, 352479-352480 (OSUC); OSUC 320382, 320408, 352478, 352912, 361208, 361351, 361365 (QSBG). **VIETNAM**: 2 males, OSUC 369106 (CNCI); OSUC 277433 (RMNH).

#### Comments.

Only a few females of *Oxyscelio jugi* are known, and these appear to be variable in sculpture and metasomal length. This variation is attributed to differences in body size.

### Oxyscelio kiefferi

Dodd

urn:lsid:zoobank.org:act:E0DD3650-11B0-4D2C-934B-135E9B56790A

urn:lsid:biosci.ohio-state.edu:osuc_concepts:5024

http://species-id.net/wiki/Oxyscelio_kiefferi

**Figures 269–271.**
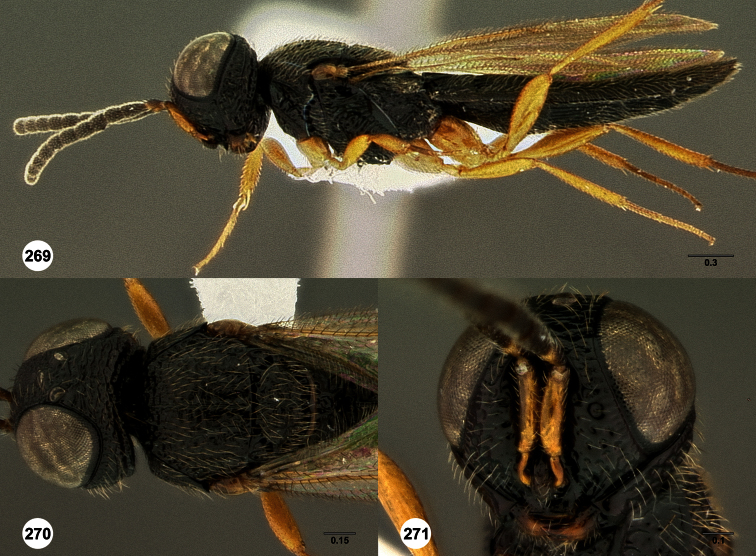
*Oxyscelio kiefferi* Dodd, neotype male (OSUC 228721) **269** Body, lateral view **270 **Head and mesosoma, dorsal view **271** Head, oblique anterior view. Morphbank^78^

Figures 269–271 Morphbank^78^

Camptoteleia flavipennis Kieffer, 1914: 296, 297 (original description, keyed; preoccupied by *Xenoteleia flavipennis*Kieffer (1913b); Kieffer 1916: 171 (keyed); Kieffer 1926: 380, 383 (description, keyed).
Oxyscelio kiefferi Dodd: Dodd 1931: 75 (replacement name, generic transfer).

#### Description.

*Female*. Unknown.

*Male*. Body length 3.8–3.85 mm (n=2).

Radicle color: same color as scape. Scape color: Yellowish. A5 tyloid: carina-like, not expanded. A11: longer than broad.

Interantennal process: not elongate. Median longitudinal elevation in frontal depression: absent. Frontal depression: concave. Frontal depression sculpture: without transverse or oblique carinae below submedian carina. Submedian carina: strong, formed by a sharp raised carina. Submedian carina medially: without peak. Median tooth of frontal depression: absent. Concavity across dorsal part of frontal depression: absent. Depression extending ventrally from median ocellus: absent. Upper frons: not hood-like. Malar area near antennal foramen: without carina or expansion. Malar area at mouth corner: with radiating striae. Smooth strip along posterior side of malar sulcus: absent or not consistently broad. Middle genal carina: present. Direction of middle genal carina dorsally: parallel to eye margin. Major sculpture of gena anteriorly: umbilicate-foveate. Major sculpture of gena posteriorly: umbilicate-foveate; rugose. Microsculpture of gena anteroventrally: absent. Microsculpture of gena posteroventrally: absent. Median carina extending posteriorly from hyperoccipital carina: absent. Hyperoccipital carina: indicated by rugae. Lateral connection between hyperoccipital and occipital carinae: absent. Area between vertex and occipital carina: umbilicate-foveate. Occipital carina medially: absent. Lateral corners of occipital carina: not protruding.

Lateral pronotal area: without bulge projecting towards anterior pit. Epomial corner: strong. Netrion surface anteriorly: not inflexed. Mesoscutum anteriorly: not steep. Mesoscutal median carina: present and complete. Longitudinal carina between median carina and notauli: absent. Major sculpture of medial mesoscutum anteriorly: umbilicate-foveate; irregularly rugose. Major sculpture of medial mesoscutum posteriorly: umbilicate-foveate. Microsculpture of medial mesoscutum anteriorly: granulate. Microsculpture of medial mesoscutum posteriorly: absent; granulate. Major sculpture of mesoscutellum: umbilicate-foveate. Microsculpture of mesoscutellum medially: absent. Microsculpture of mesoscutellum laterally: granulate. Mesoscutellar apex: convex or straight. Setae along anterior limit of femoral depression: arising from rows of foveae. Number of carinae crossing speculum above femoral depression: 3. Number of carinae crossing femoral depression: more than 5. Mesepimeral sulcus pits: more than 5. Metascutellum dorsally: concave. Metascutellar sculpture dorsally: smooth or with transverse carinae. Median carina of metascutellum: absent or branched. Metascutellar setae: absent. Metascutellar apex: convex or straight. Metapleuron above ventral metapleural area: crossed by carinae. Metasomal depression setae: absent. Anterior areoles of metasomal depression: absent. Anterior longitudinal carinae in metasomal depression: absent. Lateral propodeal areas: separated medially. Postmarginal vein: present.

Median lobe of T1: with 3 longitudinal carinae. Metasomal apex: with acuminate lateral corners.

#### Diagnosis.

Male: A11 longer than broad. Middle genal carina subparallel with eye margin. Hyperoccipital carina indicated by rugae. Mesoscutellum with granulate sculpture laterally but not medially. Metascutellum concave dorsally, smooth aside from some transverse carinae. T1 midlobe with 3 longitudinal carinae. T7 with short, sharp and protruding posterolateral corners.

#### Link to distribution map.

[http://hol.osu.edu/map-full.html?id=5024]

#### Material examined.

Neotype, male: **PHILIPPINES**: Negros Oriental Prov., 16km W Dumaguete, disturbed riparian forest, ROM 873021, Camp Lookout, 14.V-16.V.1987, malaise trap, D. C. Darling, OSUC 228721 (deposited in ROME). *Other material*: **PHILIPPINES**: 1 male, OSUC 268275 (USNM).

#### Comments.

The type material of *Camptoteleia flavipennis* Kieffer, collected from Laguna, Los Baños, in the Philippines, could not be found after an extensive search of collections known to house Kieffer type material. The neotype of *Camptoteleia flavipennis* is presently designated to clarify the taxonomic status of the species. It was selected because it was collected in the Philippines and resembles Kieffer’s (1913b) description in having a short metasoma and smooth frontal depression. We presume that Kieffer was mistaken when he mentioned that T7 lacked armature.

### Oxyscelio kramatos

Burks sp. n.

urn:lsid:zoobank.org:act:F06E950E-9DE5-4330-A32A-F6C42BE982C6

urn:lsid:biosci.ohio-state.edu:osuc_concepts:305703

http://species-id.net/wiki/Oxyscelio_kramatos

**Figures 272–277.**
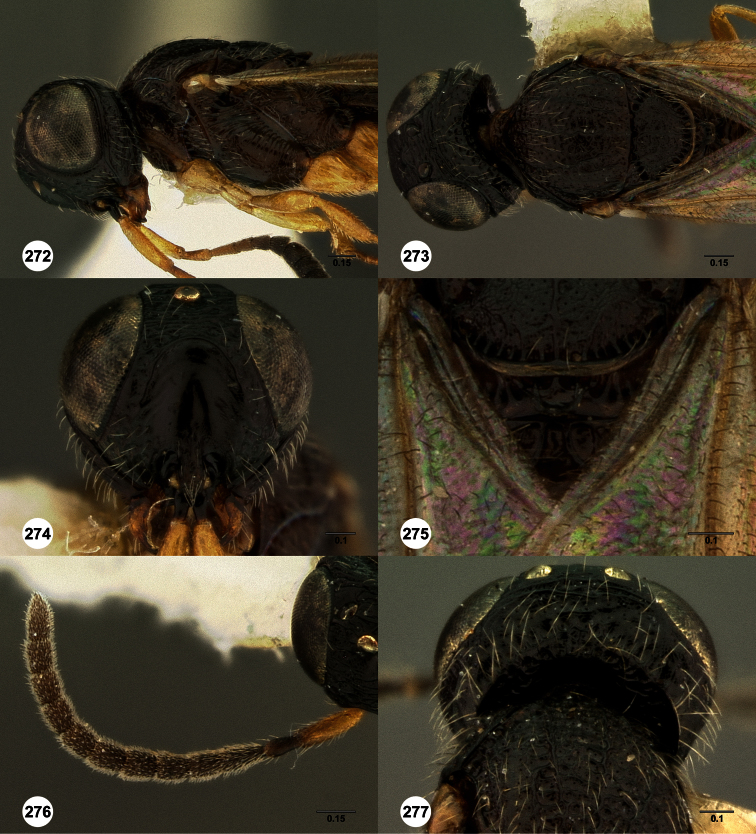
*Oxyscelio kramatos* sp. n., holotype female (OSUC 439696) **272** Head and mesosoma, lateral view **273** Head and mesosoma, dorsal view **274** Head, anterior view **275** Propodeum, posterodorsal view. Paratype male (OSUC 439741) **276** Antenna **277** Metasoma, dorsal view. Morphbank^79^

Figures 272–277 Morphbank^79^

#### Description.

*Female*. Body length 3.65 mm (n=1).

Radicle color: darker than scape. Scape color: Yellowish. A4: longer than broad. A5: longer than broad; as long as broad. Antennal club: formed, segments compact.

Interantennal process: not elongate. Median longitudinal elevation in frontal depression: absent. Frontal depression: concave. Frontal depression sculpture: with 3 or more broadly interrupted transverse carinae. Submedian carina: strong, formed by a sharp raised carina. Submedian carina medially: without peak. Concavity across dorsal part of frontal depression: absent. Depression extending ventrally from median ocellus: absent. Upper frons: not hood-like. Malar area near antennal foramen: without carina or expansion. Malar area at mouth corner: with radiating striae. Smooth strip along posterior side of malar sulcus: absent or not consistently broad. Middle genal carina: present. Direction of middle genal carina dorsally: parallel to eye margin. Major sculpture of gena anteriorly: umbilicate-foveate; rugose. Major sculpture of gena posteriorly: umbilicate-foveate; rugose. Microsculpture of gena anteroventrally: granulate. Microsculpture of gena posteroventrally: granulate. Median carina extending posteriorly from hyperoccipital carina: absent. Hyperoccipital carina: indicated by rugae. Lateral connection between hyperoccipital and occipital carinae: present as a weak elevation. Area between vertex and occipital carina: irregularly rugose. Occipital carina medially: slightly convex, flatter medially than laterally. Lateral corners of occipital carina: not protruding.

Lateral pronotal area: without bulge projecting towards anterior pit. Epomial corner: strong. Netrion surface anteriorly: not inflexed. Mesoscutum anteriorly: not steep. Mesoscutal median carina: present and complete. Longitudinal carina between median carina and notauli: absent. Major sculpture of medial mesoscutum anteriorly: umbilicate-foveate. Major sculpture of medial mesoscutum posteriorly: umbilicate-punctate; irregularly rugose. Microsculpture of medial mesoscutum anteriorly: granulate. Microsculpture of medial mesoscutum posteriorly: absent. Major sculpture of mesoscutellum: umbilicate-foveate; umbilicate-punctate. Microsculpture of mesoscutellum medially: granulate. Microsculpture of mesoscutellum laterally: granulate. Mesoscutellar apex: convex or straight. Setae along anterior limit of femoral depression: arising from rows of foveae. Number of carinae crossing speculum above femoral depression: 4. Number of carinae crossing femoral depression: more than 5. Mesepimeral sulcus pits: more than 5. Metascutellum dorsally: concave. Metascutellar sculpture dorsally: smooth or with transverse carinae. Median carina of metascutellum: absent or branched. Metascutellar setae: absent. Metascutellar apex: weakly emarginate. Metapleuron above ventral metapleural area: crossed by carinae; smooth. Metasomal depression setae: absent. Lateral propodeal carinae anteromedially: weakly diverging. Anterior areoles of metasomal depression: absent. Anterior longitudinal carinae in metasomal depression: absent. Lateral propodeal areas: separated medially. Postmarginal vein: present. Fore wing apex: reaching beyond T6.

T1 midlobe: with 4 longitudinal carinae. T1: without anterior bulge. T2: with straight longitudinal striae or rugae. T6: broader than long. Apical flange of T6: exposed apically. Metasomal apex: rounded. Major sculpture of T6: umbilicate-punctate; longitudinally striate or rugose. Microsculpture of T6: granulate.

*Male*. Body length 3.65–4 mm (n=2). A5 tyloid: carina-like, not expanded. A11: longer than broad. Median tooth of frontal depression: absent. Median lobe of T1: with 4 longitudinal carinae. Metasomal apex: with tiny rounded tubercles.

#### Diagnosis.

Both sexes: Frons without elevation between antennal foramen and eye. Hyperoccipital carina present, continuous with vague anterior genal carina. Gena with some strong sculpture, at least one strong ruga along middle. Mesoscutellum strongly granulate. Metascutellum subrectangular, weakly emarginate. Metasomal depression elongate, without extensive sculpture; lateral propodeal carinae narrowly separated anteriorly. Female: T1 midlobe with 5 longitudinal carinae. T6 rounded apically. Male: T1 midlobe with 4 longitudinal carinae. T7 with posterolateral tubercles. *Oxyscelio kramatos* shares several characters with *Oxyscelio vadorum*, but has stronger genal sculpture and little or no postmarginal vein. It also strongly resembles the *crateris*-group in having a vaguely crater-like occiput, but this is not as laterally well-defined as in that group.

#### Etymology.

Noun based on Greek, meaning “mixture.” Meant to indicate the morphological similarity to both *Oxyscelio vadorum* and the *crateris*-group.

#### Link to distribution map.

[http://hol.osu.edu/map-full.html?id=305703]

#### Material examined.

Holotype, female: **TAIWAN**: Taiwan Prov., Nantou Co., Dongbu (Tungpu), 1200m, 18.X–21.X.1982, K.-C. Chou & S.-C. Lin, OSUC 439696 (deposited in TARI). *Paratypes*: **TAIWAN**: 2 males, OSUC 439719, 439741 (TARI).

### Oxyscelio labis

Burks sp. n.

urn:lsid:zoobank.org:act:7014AC65-912B-468C-8065-B739BA288373

urn:lsid:biosci.ohio-state.edu:osuc_concepts:275572

http://species-id.net/wiki/Oxyscelio_labis

**Figures 278–281.**
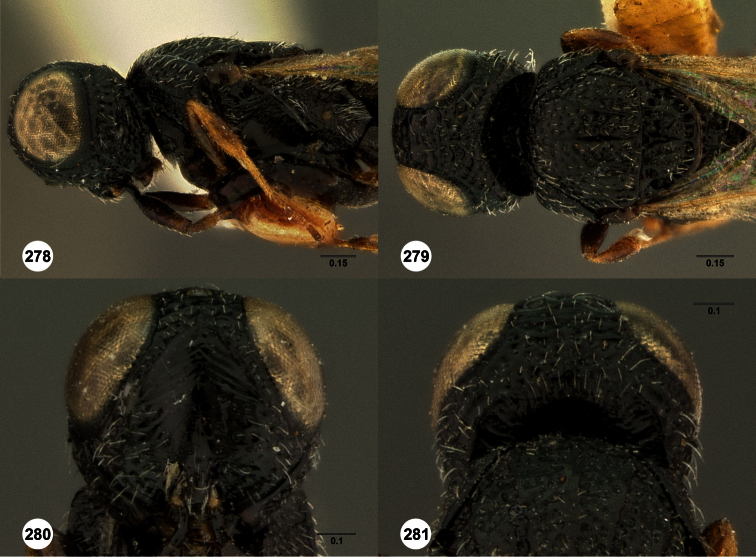
*Oxyscelio labis* sp. n., holotype female (OSUC 376761) **278** Head and mesosoma, lateral view **279** Head and mesosoma, dorsal view **280** Head, anterior view **281** Head, posterodorsal view. Morphbank^80^

Figures 278–281 Morphbank^80^

#### Description.

*Female*. Body length 2.9 mm (n=1).

Radicle color: darker than scape. Scape color: Brown. A4: broader than long. A5: broader than long. Antennal club: formed, segments compact.

Interantennal process: not elongate. Median longitudinal elevation in frontal depression: present. Frontal depression: concave. Frontal depression sculpture: with 3 or more broadly interrupted transverse carinae. Submedian carina: weak, shallow and rounded or formed by ledge. Submedian carina medially: with sharp peak. Concavity across dorsal part of frontal depression: absent. Depression extending ventrally from median ocellus: absent. Upper frons: not hood-like. Malar area near antennal foramen: without carina or expansion. Malar area at mouth corner: with radiating striae. Smooth strip along posterior side of malar sulcus: absent or not consistently broad. Middle genal carina: present. Direction of middle genal carina dorsally: parallel to eye margin. Major sculpture of gena anteriorly: umbilicate-foveate. Major sculpture of gena posteriorly: umbilicate-foveate; rugose. Microsculpture of gena anteroventrally: absent. Microsculpture of gena posteroventrally: granulate. Median carina extending posteriorly from hyperoccipital carina: present. Hyperoccipital carina: not indicated medially. Lateral connection between hyperoccipital and occipital carinae: absent. Area between vertex and occipital carina: umbilicate-foveate. Occipital carina medially: slightly convex, flatter medially than laterally. Lateral corners of occipital carina: sharp and protruding.

Lateral pronotal area: without bulge projecting towards anterior pit. Epomial corner: strong. Netrion surface anteriorly: not inflexed. Mesoscutum anteriorly: not steep. Mesoscutal median carina: present and complete. Longitudinal carina between median carina and notauli: absent. Major sculpture of medial mesoscutum anteriorly: umbilicate-foveate; umbilicate-punctate. Major sculpture of medial mesoscutum posteriorly: umbilicate-foveate. Microsculpture of medial mesoscutum anteriorly: granulate. Microsculpture of medial mesoscutum posteriorly: absent. Major sculpture of mesoscutellum: umbilicate-foveate; longitudinally rugose. Microsculpture of mesoscutellum medially: absent. Microsculpture of mesoscutellum laterally: granulate. Mesoscutellar apex: convex or straight. Setae along anterior limit of femoral depression: arising from rows of foveae. Number of carinae crossing speculum above femoral depression: 2. Number of carinae crossing femoral depression: more than 5. Mesepimeral sulcus pits: more than 5. Metascutellum dorsally: concave. Metascutellar sculpture dorsally: smooth or with transverse carinae. Median carina of metascutellum: absent or branched. Metascutellar setae: absent. Metascutellar apex: weakly emarginate. Metapleuron above ventral metapleural area: crossed by carinae. Metasomal depression setae: absent. Lateral propodeal carinae anteromedially: weakly diverging. Anterior areoles of metasomal depression: one or more areoles present. Anterior longitudinal carinae in metasomal depression: absent. Lateral propodeal areas: separated medially. Postmarginal vein: present. Fore wing apex: reaching middle of T6.

T1 midlobe: with 5 longitudinal carinae. T1: without anterior bulge. T2: with straight longitudinal striae or rugae. T6: broader than long. Apical flange of T6: exposed apically. Metasomal apex: rounded. Major sculpture of T6: umbilicate-punctate. Microsculpture of T6: granulate.

*Male*. Unknown.

#### Diagnosis.

Female: A4, A5 broader than long. Radicle elongate, much darker than scape. Submedian carina with a sharp median peak. Occiput with median carina and lateral rugae extending from weakly defined hyperoccipital carina. Occipital carina sinuate medially. Mesoscutellum granulate laterally. Fore wings long enough to reach middle of T6. T1 midlobe with 5 longitudinal carinae. T6 broader than long, rounded apically.

#### Etymology.

Latin noun, genitive case, meaning “a fall.”

#### Link to distribution map.

[http://hol.osu.edu/map-full.html?id=275572]

#### Material examined.

Holotype, female: **SINGAPORE**: no date, Baker, OSUC 376761 (deposited in MCZC).

### Oxyscelio lacunae

Burks sp. n.

urn:lsid:zoobank.org:act:A1B6F1A6-BE62-4FFE-8C19-3F754C5908D2

urn:lsid:biosci.ohio-state.edu:osuc_concepts:305773

http://species-id.net/wiki/Oxyscelio_lacunae

**Figures 282–283.**
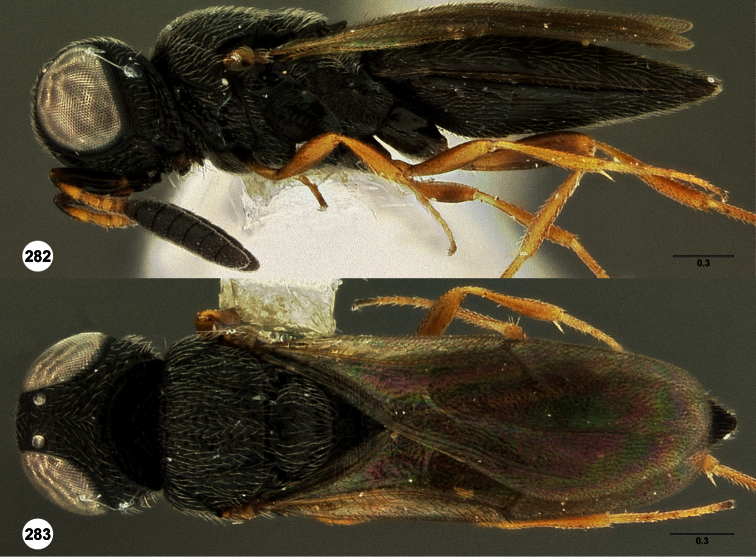
*Oxyscelio lacunae* sp. n., holotype female (OSUC 448561) **282** Head and mesosoma, lateral view **283** Head and mesosoma, dorsal view. Morphbank^81^

Figures 282–283 Morphbank^81^

#### Description.

*Female*. Body length 3.45 mm (n=1).

Radicle color: same color as scape. Scape color: Brown. A4: broader than long. A5: broader than long. Antennal club: formed, segments compact.

Interantennal process: not elongate. Median longitudinal elevation in frontal depression: absent. Frontal depression: concave. Frontal depression sculpture: with 3-5 complete transverse carinae. Submedian carina: weak, shallow and rounded or formed by ledge. Submedian carina medially: without peak. Concavity across dorsal part of frontal depression: absent. Depression extending ventrally from median ocellus: absent. Upper frons: not hood-like. Malar area near antennal foramen: without carina or expansion. Malar area at mouth corner: without striae. Smooth strip along posterior side of malar sulcus: absent or not consistently broad. Middle genal carina: present. Direction of middle genal carina dorsally: parallel to eye margin. Major sculpture of gena anteriorly: umbilicate-foveate. Major sculpture of gena posteriorly: rugose; umbilicate-punctate. Microsculpture of gena anteroventrally: absent. Microsculpture of gena posteroventrally: granulate. Median carina extending posteriorly from hyperoccipital carina: absent. Hyperoccipital carina: indicated by rugae. Lateral connection between hyperoccipital and occipital carinae: absent. Area between vertex and occipital carina: umbilicate-foveate. Occipital carina medially: uniformly rounded. Lateral corners of occipital carina: not protruding.

Lateral pronotal area: without bulge projecting towards anterior pit. Epomial corner: strong. Netrion surface anteriorly: not inflexed. Mesoscutum anteriorly: steep. Mesoscutal median carina: present and complete. Longitudinal carina between median carina and notauli: absent. Major sculpture of medial mesoscutum anteriorly: umbilicate-foveate. Major sculpture of medial mesoscutum posteriorly: umbilicate-punctate. Microsculpture of medial mesoscutum anteriorly: granulate. Microsculpture of medial mesoscutum posteriorly: absent. Major sculpture of mesoscutellum: umbilicate-foveate; irregularly rugose. Microsculpture of mesoscutellum medially: absent. Microsculpture of mesoscutellum laterally: absent. Mesoscutellar apex: convex or straight. Setae along anterior limit of femoral depression: arising from rows of foveae.

Number of carinae crossing speculum above femoral depression: 2. Number of carinae crossing femoral depression: more than 5. Mesepimeral sulcus pits: more than 5. Metascutellum dorsally: concave. Metascutellar sculpture dorsally: smooth or with transverse carinae. Median carina of metascutellum: absent or branched. Metascutellar setae: absent. Metascutellar apex: convex or straight. Metapleuron above ventral metapleural area: foveate or rugose. Metasomal depression setae: absent. Lateral propodeal carinae anteromedially: strongly diverging. Anterior areoles of metasomal depression: absent. Anterior longitudinal carinae in metasomal depression: absent. Lateral propodeal areas: meeting for only a short distance medially. Postmarginal vein: absent. Fore wing apex: reaching apex of T5.

T1 midlobe: with 5 longitudinal carinae. T1: without anterior bulge. T2: with straight longitudinal striae or rugae. T6: broader than long. Apical flange of T6: exposed apically. Metasomal apex: rounded. Major sculpture of T6: umbilicate-punctate. Microsculpture of T6: granulate.

*Male*. Unknown.

#### Diagnosis.

Female: A4, A5 broader than long. Frontal depression crossed by many carinae. Submedian carina undefined. Hyperoccipital carina indicated by rugae; occipital carina without distinct lateral corners. Mesoscutellum without granulate sculpture. Metascutellum tiny, narrow. T1 midlobe with 5 longitudinal carinae. Fore wings long enough to reach apex of T5. T6 broader than long. *Oxyscelio lacunae* is an unusual species that resembles *Oxyscelio crebritas* in some ways, but has an indistinct submedian carina, tiny metascutellum, and irregular metapleural sculpture. The overall body color is dark, with the scape, wings, and coxae brownish.

#### Etymology.

Latin noun, genitive case, meaning “pit.”

#### Link to distribution map.

[http://hol.osu.edu/map-full.html?id=305773]

#### Material examined.

Holotype, female: **MALAYSIA**: Selangor St., Putra University of Malaysia (UPM) campus, scrub / around rubber plantation, Serdang, 22.VIII-4.IX.1992, sweeping, A. D. Austin, OSUC 448561 (deposited in UKMC).

### Oxyscelio latinubbin

Burks sp. n.

urn:lsid:zoobank.org:act:417CE843-7ABB-413C-8407-2A3465D74AE7

urn:lsid:biosci.ohio-state.edu:osuc_concepts:275544

http://species-id.net/wiki/Oxyscelio_latinubbin

**Figures 284–287.**
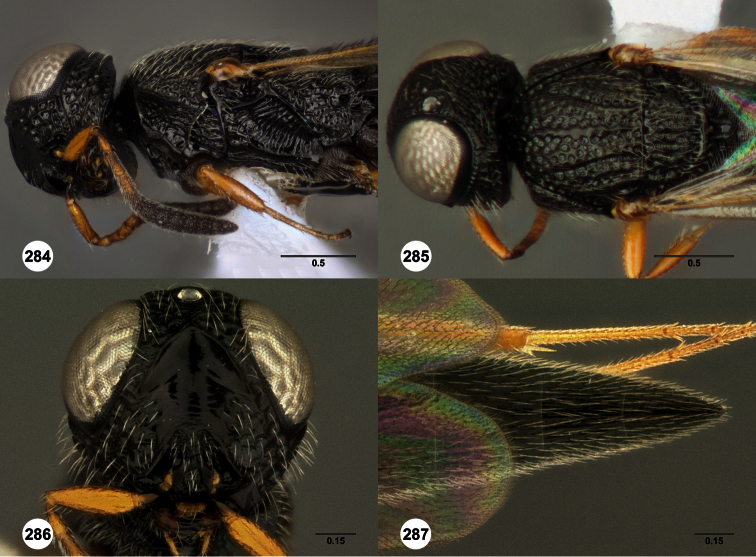
*Oxyscelio latinubbin* sp. n., paratype female (OSUC 252040) **284** Head and mesosoma, lateral view **285** Head and mesosoma, dorsal view **286** Head, anterior view **287** Metasoma, dorsal view. Morphbank^82^

Figures 284–287 Morphbank^82^

#### Description.

*Female*. Body length 4.9–5.25 mm (n=2).

Radicle color: darker than scape. Scape color: Yellowish. A4: longer than broad. A5: longer than broad. Antennal club: formed, segments compact.

Interantennal process: not elongate. Median longitudinal elevation in frontal depression: absent. Frontal depression: concave. Frontal depression sculpture: with 1 complete transverse carina. Submedian carina: strong, formed by a sharp raised carina. Submedian carina medially: with sharp peak. Concavity across dorsal part of frontal depression: absent. Depression extending ventrally from median ocellus: absent. Upper frons: not hood-like. Malar area near antennal foramen: with oblique tooth-like flange (facial nubbin). Malar area at mouth corner: without striae. Smooth strip along posterior side of malar sulcus: absent or not consistently broad. Middle genal carina: present. Direction of middle genal carina dorsally: parallel to eye margin. Major sculpture of gena anteriorly: umbilicate-foveate. Major sculpture of gena posteriorly: absent; umbilicate-foveate. Microsculpture of gena anteroventrally: absent. Microsculpture of gena posteroventrally: absent. Median carina extending posteriorly from hyperoccipital carina: absent. Hyperoccipital carina: not indicated medially. Lateral connection between hyperoccipital and occipital carinae: absent. Area between vertex and occipital carina: umbilicate-foveate. Occipital carina medially: sinuate, concave medial to corners, but without a median peak. Lateral corners of occipital carina: sharp and protruding.

Lateral pronotal area: without bulge projecting towards anterior pit. Epomial corner: strong. Netrion surface anteriorly: not inflexed. Mesoscutum anteriorly: not steep. Mesoscutal median carina: present and complete. Longitudinal carina between median carina and notauli: absent. Major sculpture of medial mesoscutum anteriorly: umbilicate-foveate. Major sculpture of medial mesoscutum posteriorly: umbilicate-foveate. Microsculpture of medial mesoscutum anteriorly: granulate. Microsculpture of medial mesoscutum posteriorly: granulate. Major sculpture of mesoscutellum: umbilicate-foveate. Microsculpture of mesoscutellum medially: granulate. Microsculpture of mesoscutellum laterally: granulate. Mesoscutellar apex: convex or straight. Setae along anterior limit of femoral depression: arising from rows of foveae. Number of carinae crossing speculum above femoral depression: 2. Number of carinae crossing femoral depression: more than 5. Mesepimeral sulcus pits: more than 5. Metascutellum dorsally: concave. Metascutellar sculpture dorsally: smooth or with transverse carinae. Median carina of metascutellum: absent or branched. Metascutellar setae: absent. Metascutellar apex: convex or straight. Metapleuron above ventral metapleural area: crossed by carinae. Metasomal depression setae: absent. Lateral propodeal carinae anteromedially: strongly diverging. Anterior areoles of metasomal depression: absent. Anterior longitudinal carinae in metasomal depression: absent. Lateral propodeal areas: meeting for only a short distance medially. Postmarginal vein: absent. Fore wing apex: reaching middle of T4; reaching apex of T4.

T1 midlobe: with 5 longitudinal carinae. T1: without anterior bulge. T2: with straight longitudinal striae or rugae. T6: longer than broad. Apical flange of T6: not exposed apically. Metasomal apex: tapering to a sharp point. Major sculpture of T6: umbilicate-punctate; longitudinally striate or rugose. Microsculpture of T6: granulate.

*Male*. Unknown.

#### Diagnosis.

Female: Antennal club formed. A4, A5 longer than broad. Face with broad oblique expanded flange between antennal foramen and eye. Metascutellum longer than broad, with central smooth channel. *Oxyscelio latinubbin* is similar to *Oxyscelio aclavae*, in having a long metascutellum with a median channel and a propodeum that forms a short arch above the anterior part of T1. It differs in having a swollen, compact antennal club.

#### Etymology.

Compound noun intended to mean “broad nubbin.” Refers to the unusual broad oblique flange between the antennal foramen and eye.

#### Link to distribution map.

[http://hol.osu.edu/map-full.html?id=275544]

#### Material examined.

Holotype, female: **THAILAND**: Chiang Mai Prov., Thung Buatong Viewpoint, T2849, Huai Nam Dang National Park, 19°17.470'N, 98°36.033'E, 1464m, 31.VII-7.VIII.2007, malaise trap, Thawatchai & Anuchart, OSUC 361193 (deposited in QSBG). *Paratype*: **THAILAND**: 1 female, OSUC 252040 (OSUC).

### Oxyscelio latitudinis

Burks sp. n.

urn:lsid:zoobank.org:act:057F9DAD-0E68-40D6-9652-89DA119BFE87

urn:lsid:biosci.ohio-state.edu:osuc_concepts:275546

http://species-id.net/wiki/Oxyscelio_latitudinis

**Figures 288–293.**
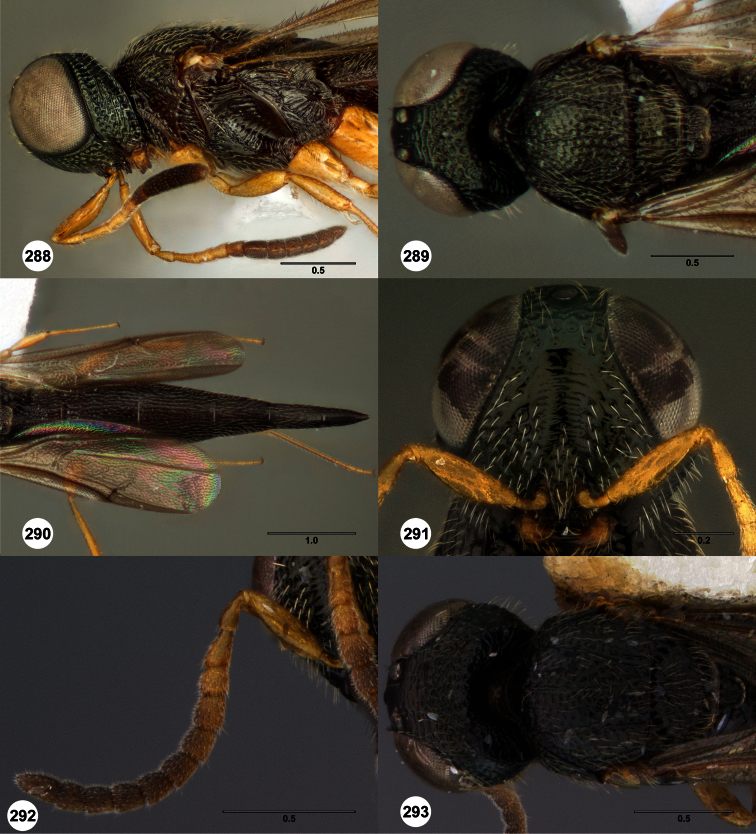
*Oxyscelio latitudinis* sp. n., holotype female (OSUC 247927) **288** Head and mesosoma, lateral view **289** Head and mesosoma, dorsal view **290** Metasoma, dorsal view. Paratype female (OSUC 368751) **291** Head, anterior view. Paratype male (OSUC 436883) **292** Antenna **293** Metasoma, dorsal view. Morphbank^83^

Figures 288–293 Morphbank^83^

#### Description.

*Female*. Body length 4.65–5.8 mm (n=15).

Radicle color: same color as scape. Scape color: Yellowish. A4: longer than broad. A5: longer than broad; as long as broad. Antennal club: formed, segments compact.

Interantennal process: not elongate. Median longitudinal elevation in frontal depression: present. Frontal depression: concave. Frontal depression sculpture: crossed by many tiny furrows. Submedian carina: weak, shallow and rounded or formed by ledge. Submedian carina medially: without peak. Concavity across dorsal part of frontal depression: absent. Depression extending ventrally from median ocellus: absent. Upper frons: not hood-like. Malar area near antennal foramen: without carina or expansion. Malar area at mouth corner: with radiating striae. Smooth strip along posterior side of malar sulcus: present, broad throughout its length. Middle genal carina: present. Direction of middle genal carina dorsally: parallel to eye margin. Major sculpture of gena anteriorly: umbilicate-foveate; rugose. Major sculpture of gena posteriorly: umbilicate-foveate; rugose. Microsculpture of gena anteroventrally: absent. Microsculpture of gena posteroventrally: granulate. Median carina extending posteriorly from hyperoccipital carina: absent. Hyperoccipital carina: indicated by rugae. Lateral connection between hyperoccipital and occipital carinae: absent. Area between vertex and occipital carina: umbilicate-foveate. Occipital carina medially: absent. Lateral corners of occipital carina: sharp and protruding.

Lateral pronotal area: without bulge projecting towards anterior pit. Epomial corner: strong. Netrion surface anteriorly: not inflexed. Mesoscutum anteriorly: not steep. Mesoscutal median carina: absent or weak and incomplete in places. Longitudinal carina between median carina and notauli: absent. Major sculpture of medial mesoscutum anteriorly: umbilicate-foveate. Major sculpture of medial mesoscutum posteriorly: umbilicate-foveate; longitudinally rugose. Microsculpture of medial mesoscutum anteriorly: granulate. Microsculpture of medial mesoscutum posteriorly: absent. Major sculpture of mesoscutellum: umbilicate-foveate. Microsculpture of mesoscutellum medially: granulate. Microsculpture of mesoscutellum laterally: granulate. Mesoscutellar apex: convex or straight; roundly concave. Setae along anterior limit of femoral depression: arising from rows of foveae. Number of carinae crossing speculum above femoral depression: 3. Number of carinae crossing femoral depression: more than 5. Mesepimeral sulcus pits: more than 5. Metascutellum dorsally: flat; convex. Metascutellar sculpture dorsally: with scattered rugae. Median carina of metascutellum: absent or branched. Metascutellar setae: absent. Metascutellar apex: convex or straight; weakly emarginate. Metapleuron above ventral metapleural area: crossed by carinae. Metasomal depression setae: absent. Lateral propodeal carinae anteromedially: strongly diverging. Anterior areoles of metasomal depression: absent. Anterior longitudinal carinae in metasomal depression: absent. Lateral propodeal areas: separated medially. Postmarginal vein: present. Fore wing apex: reaching middle of T4; reaching apex of T4.

T1 midlobe: obscured by other raised sculpture. T1: with long anterior bulge, reaching metascutellum. T2: with straight longitudinal striae or rugae. T6: longer than broad. Apical flange of T6: not exposed apically. Metasomal apex: rounded. Major sculpture of T6: umbilicate-punctate. Microsculpture of T6: granulate.

*Male*. Body length 4.4 mm (n=1). A5 tyloid: carina-like, not expanded. A11: broader than long. Median tooth of frontal depression: absent. Median lobe of T1: with 5 longitudinal carinae. Metasomal apex: with acuminate lateral corners.

#### Diagnosis.

Both sexes: Frontal depression crossed by many carinae that are medially discontinuous. Mesoscutellum strongly granulate. Metascutellum dorsally bare. Female: A4 longer than broad, A5 about as long as broad. Metascutellum very broad, rugose. T1 with strong anterior horn. Fore wings not long enough to reach middle of T5. T5 and T6 elongate and nearly parallel-sided. Male: A11 broader than long. T1 midlobe with 5 longitudinal carinae; T7 with acuminate lateral corners.

#### Etymology.

Latin noun, genitive case, meaning “width.” Refers to the large metascutellum.

#### Link to distribution map.

[http://hol.osu.edu/map-full.html?id=275546]

#### Material examined.

Holotype, female: **INDONESIA**: Kalimantan Barat Prov., Cabang Panti Research Station, 1° rainforest / alluvial light gap, IIS 910122, Gunung Palung National Park, 01°15'S, 110°05'E, 100-400m, 15.VI-15.VIII.1991, malaise trap, Darling & Rosichon, OSUC 247927 (deposited in MBBJ). *Paratypes*: (15 females, 1 male) **BRUNEI**: 1 female, OSUC 376641 (BMNH). **INDONESIA**: 5 females, OSUC 369078 (CNCI); OSUC 228714 (MBBJ); OSUC 257092, 369076 (OSUC); OSUC 240913 (ROME). **SINGAPORE**: 5 females, 1 male, OSUC 436882-436883 (ANIC); OSUC 376753-376754, 376760, 376762 (MCZC). **THAILAND**: 4 females, OSUC 368733, 368751 (CNCI); UCRC ENT 135010, 135137 (UCRC).

### Oxyscelio limae

Burks sp. n.

urn:lsid:zoobank.org:act:C7BA9F04-F257-4CA2-BEAD-6D673281A7C9

urn:lsid:biosci.ohio-state.edu:osuc_concepts:275508

http://species-id.net/wiki/Oxyscelio_limae

**Figures 294–299.**
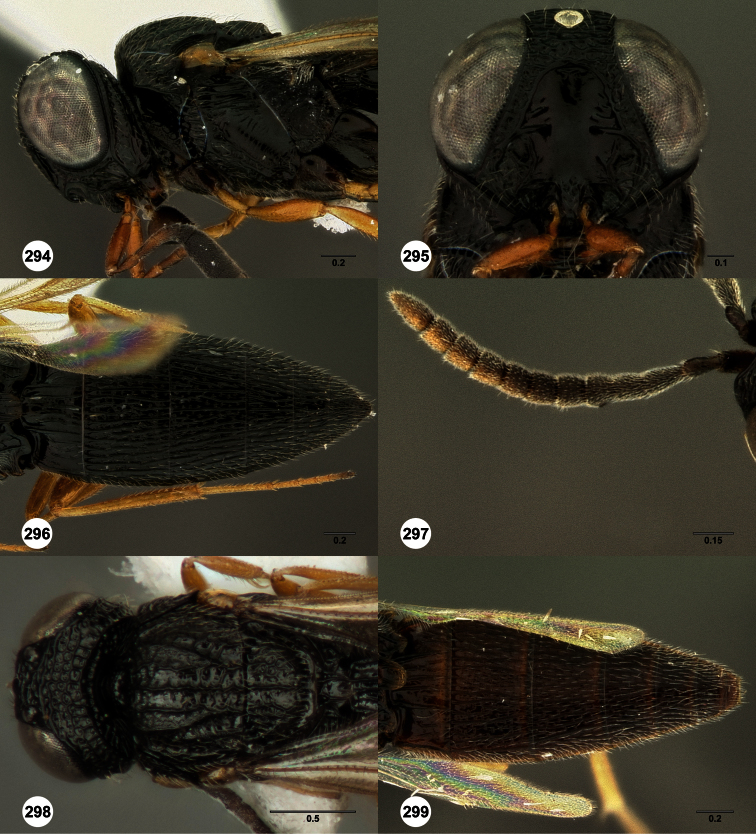
*Oxyscelio limae* sp. n., holotype female (OSUC 369087) **294** Head and mesosoma, lateral view **295** Head, anterior view. Paratype female (OSUC 268169) **296** Metasoma, dorsal view. Paratype male (OSUC 268114) **297** Antenna. Paratype male (OSUC 268148) **298** Head and mesosoma, dorsal view. Paratype male (OSUC 268278) **299** Metasoma, dorsal view. Morphbank^84^

Figures 294–299 Morphbank^84^

#### Description.

*Female*. Body length 3.65–4.1 mm (n=20).

Radicle color: same color as scape. Scape color: Yellowish. A4: broader than long. A5: broader than long. Antennal club: formed, segments compact.

Interantennal process: not elongate. Median longitudinal elevation in frontal depression: absent. Frontal depression: concave. Frontal depression sculpture: with 3 or more broadly interrupted transverse carinae. Submedian carina: strong, formed by a sharp raised carina. Submedian carina medially: without peak. Concavity across dorsal part of frontal depression: present. Depression extending ventrally from median ocellus: absent. Upper frons: not hood-like. Malar area near antennal foramen: without carina or expansion. Malar area at mouth corner: without striae. Smooth strip along posterior side of malar sulcus: absent or not consistently broad. Middle genal carina: present. Direction of middle genal carina dorsally: parallel to eye margin. Major sculpture of gena anteriorly: rugose; umbilicate-punctate. Major sculpture of gena posteriorly: umbilicate-foveate. Microsculpture of gena anteroventrally: absent. Microsculpture of gena posteroventrally: absent. Median carina extending posteriorly from hyperoccipital carina: absent. Hyperoccipital carina: not indicated medially. Lateral connection between hyperoccipital and occipital carinae: absent. Area between vertex and occipital carina: with transverse carinae. Occipital carina medially: uniformly rounded. Lateral corners of occipital carina: not protruding.

Lateral pronotal area: without bulge projecting towards anterior pit. Epomial corner: strong. Netrion surface anteriorly: not inflexed. Mesoscutum anteriorly: steep. Mesoscutal median carina: present and complete. Longitudinal carina between median carina and notauli: present. Major sculpture of medial mesoscutum anteriorly: umbilicate-punctate; irregularly rugose. Major sculpture of medial mesoscutum posteriorly: umbilicate-foveate; umbilicate-punctate; irregularly rugose. Microsculpture of medial mesoscutum anteriorly: granulate. Microsculpture of medial mesoscutum posteriorly: absent. Major sculpture of mesoscutellum: umbilicate-foveate; irregularly rugose. Microsculpture of mesoscutellum medially: absent. Microsculpture of mesoscutellum laterally: granulate. Mesoscutellar apex: convex or straight. Setae along anterior limit of femoral depression: arising from rows of foveae. Number of carinae crossing speculum above femoral depression: 3. Number of carinae crossing femoral depression: more than 5. Mesepimeral sulcus pits: 3-5. Metascutellum dorsally: flat. Metascutellar sculpture dorsally: with scattered rugae. Median carina of metascutellum: absent or branched. Metascutellar setae: absent. Metascutellar apex: convex or straight. Metapleuron above ventral metapleural area: crossed by carinae. Metasomal depression setae: absent. Lateral propodeal carinae anteromedially: strongly diverging. Anterior areoles of metasomal depression: absent. Anterior longitudinal carinae in metasomal depression: absent. Lateral propodeal areas: separated medially. Postmarginal vein: absent. Fore wing apex: reaching apex of T4.

T1 midlobe: obscured by other raised sculpture. T1: with small rounded anterior bulge, not reaching metascutellum. T2: with straight longitudinal striae or rugae. T6: broader than long. Apical flange of T6: not exposed apically. Metasomal apex: rounded. Major sculpture of T6: umbilicate-punctate; longitudinally striate or rugose. Microsculpture of T6: absent.

*Male*. Body length 3.5–4.1 mm (n=20). A5 tyloid: carina-like, not expanded. A11: broader than long. Median tooth of frontal depression: absent. Median lobe of T1: with 4 longitudinal carinae. Metasomal apex: with acuminate lateral corners.

#### Diagnosis.

Both sexes: Hyperoccipital carina indicated by a strong carina or ruga; occipital carina complete. Mesosoma very tall and steep anteriorly, descending at a right angle. Medial mesoscutum with at least 5 longitudinal carinae or sculptured elevations anteriorly, the lateral pairs merging posteriorly. Mesoscutellum with some granulate sculpture posterolaterally. Metascutellum elongate, smooth medially, extending over base of T1. Fore wing venation not reaching anterior wing margin. Female: Fore wing long enough to reach middle of T6. T1 midlobe with a smooth elevation obscuring carinae anteriorly. T6 broader than long.

Male: T1 midlobe with 4-5 longitudinal carinae. T7 with sharp, protruding posterolateral corners.

#### Etymology.

Latin noun, genitive case, meaning “carpenter’s file.” Refers to the strong longitudinal sculpture of the mesoscutum.

#### Link to distribution map.

[http://hol.osu.edu/map-full.html?id=275508]

#### Material examined.

Holotype, female: **SRI LANKA**: Central Prov., Kandy Dist., Victoria-Randenigala-Rantambe Sanctuary, 07°12.624'N, 80°56.540'E, 23.VIII-30.VIII.1999, malaise trap, M. Wasbauer & J. Wasbauer, OSUC 369087 (deposited in CNCI). *Paratypes*: **SRI LANKA**: 25 females, 28 males, OSUC 369086, 369092-369093 (CNCI); OSUC 268097, 268108-268112, 268114-268121, 268124-268126, 268129, 268131-268134, 268140-268141, 268143-268146, 268148-268149, 268151-268157, 268159-268162, 268165-268170, 268176, 268277-268278 (USNM).

#### Comments.

*Oxyscelio limae* is distinctive within its species group in being short-bodied, with a long metascutellum. The extra mesoscutal carinae appear to be more sharply and distinctly defined versions of elevated areas found between the notauli in some other *Oxyscelio* species. In males, these carinae may be less distinct, with some granulate sculpture.

### Oxyscelio longiventris

Burks sp. n.

urn:lsid:zoobank.org:act:6BE6E08D-9977-4EC8-A7B5-50CDB92457FD

urn:lsid:biosci.ohio-state.edu:osuc_concepts:275522

http://species-id.net/wiki/Oxyscelio_longiventris

**Figures 300–305.**
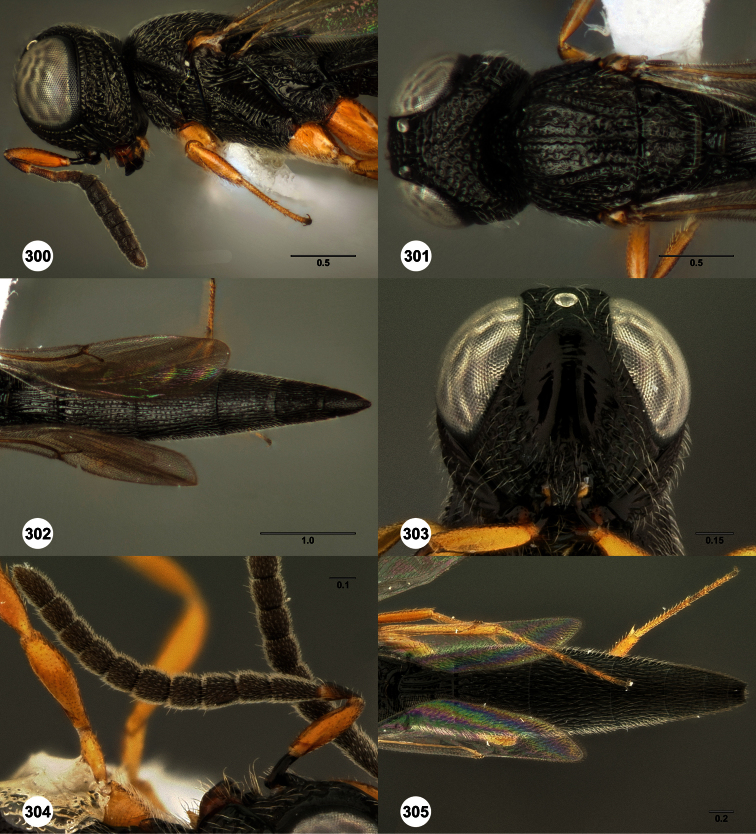
*Oxyscelio longiventris* sp. n., paratype female (OSUC 247609) **300** Head and mesosoma, lateral view **301** Head and mesosoma, dorsal view **302** Metasoma, dorsal view. Paratype female (OSUC 247625) **303** Head, anterior view Paratype male (OSUC 247612) **304** Antenna **305** Metasoma, dorsal view. Morphbank^85^

Figures 300–305 Morphbank^85^

#### Description.

*Female*. Body length 5.25–6 mm (n=11).

Radicle color: darker than scape. Scape color: Yellowish. A4: broader than long; as long as broad. A5: broader than long. Antennal club: formed, segments compact.

Interantennal process: not elongate. Median longitudinal elevation in frontal depression: absent. Frontal depression: concave. Frontal depression sculpture: with 3-5 complete transverse carinae. Submedian carina: strong, formed by a sharp raised carina. Submedian carina medially: without peak. Concavity across dorsal part of frontal depression: absent. Depression extending ventrally from median ocellus: absent. Upper frons: not hood-like. Malar area near antennal foramen: without carina or expansion. Malar area at mouth corner: with radiating striae. Smooth strip along posterior side of malar sulcus: absent or not consistently broad. Middle genal carina: present. Direction of middle genal carina dorsally: parallel to eye margin. Major sculpture of gena anteriorly: umbilicate-foveate. Major sculpture of gena posteriorly: umbilicate-foveate; rugose. Microsculpture of gena anteroventrally: absent; granulate. Microsculpture of gena posteroventrally: absent. Median carina extending posteriorly from hyperoccipital carina: absent. Hyperoccipital carina: indicated by rugae. Lateral connection between hyperoccipital and occipital carinae: absent. Area between vertex and occipital carina: umbilicate-foveate. Occipital carina medially: absent. Lateral corners of occipital carina: not protruding.

Lateral pronotal area: with slight bulge projecting anteriorly towards anterior pit. Epomial corner: strong. Netrion surface anteriorly: not inflexed. Mesoscutum anteriorly: steep. Mesoscutal median carina: present and complete. Longitudinal carina between median carina and notauli: present. Major sculpture of medial mesoscutum anteriorly: umbilicate-foveate. Major sculpture of medial mesoscutum posteriorly: umbilicate-foveate; umbilicate-punctate. Microsculpture of medial mesoscutum anteriorly: granulate. Microsculpture of medial mesoscutum posteriorly: absent. Major sculpture of mesoscutellum: umbilicate-foveate. Microsculpture of mesoscutellum medially: absent. Microsculpture of mesoscutellum laterally: absent. Mesoscutellar apex: convex or straight. Setae along anterior limit of femoral depression: arising from rows of foveae. Number of carinae crossing speculum above femoral depression: 2. Number of carinae crossing femoral depression: more than 5. Mesepimeral sulcus pits: more than 5. Metascutellum dorsally: concave. Metascutellar sculpture dorsally: smooth or with transverse carinae. Median carina of metascutellum: absent or branched. Metascutellar setae: absent. Metascutellar apex: weakly emarginate. Metapleuron above ventral metapleural area: crossed by carinae. Metasomal depression setae: absent. Lateral propodeal carinae anteromedially: strongly diverging. Anterior areoles of metasomal depression: absent. Anterior longitudinal carinae in metasomal depression: absent. Lateral propodeal areas: separated medially. Postmarginal vein: present. Fore wing apex: reaching middle of T4; reaching apex of T4.

T1 midlobe: obscured by other raised sculpture. T1: with small rounded anterior bulge, not reaching metascutellum. T2: with straight longitudinal striae or rugae. T6: longer than broad. Apical flange of T6: exposed apically. Metasomal apex: rounded. Major sculpture of T6: umbilicate-punctate; longitudinally striate or rugose. Microsculpture of T6: absent.

*Male*. Body length 4.85–5.05 mm (n=2). A5 tyloid: carina-like, not expanded. A11: longer than broad; broader than long; as long as broad. Median tooth of frontal depression: absent. Median lobe of T1: with 5 longitudinal carinae. Metasomal apex: with acuminate lateral corners.

#### Diagnosis.

Both sexes: Middle genal carina subparallel with eye margin. Hyperoccipital carina indicated by rugae. Mesoscutellum without granulate sculpture. Metascutellum concave dorsally, smooth aside from some transverse carinae. Female: T1 midlobe with anterior bulge. Fore wing long enough to reach middle of T4. T6 longer than broad, tapering to a rounded apex. Male: T1 midlobe with 5 longitudinal carinae. T7 with sharp and protruding posterolateral corners. *Oxyscelio longiventris* is very similar to several other members of the *crebritas*-group. It is distinguished by the long metasoma, weakly developed T1 horn, 5 T1 midlobe carinae and males, and lack of other unusual characters.

#### Etymology.

Latin noun in genitive case, meaning “long abdomen.”

#### Link to distribution map.

[http://hol.osu.edu/map-full.html?id=275522]

#### Material examined.

Holotype, female: **THAILAND**: Phetchabun Prov., Tham Pra Laad Forest Unit, T427, Nam Nao National Park, 16°44.963'N, 101°27.833'E, 711m, 14.VIII-21.VIII.2006, malaise trap, L. Janteab, OSUC 247644 (deposited in QSBG). *Paratypes*: (10 females, 2 males) **LAOS**: 1 female, OSUC 368882 (CNCI). **THAILAND**: 9 females, 2 males, OSUC 247653 (BMNH); OSUC 247609-247610, 247612, 247622, 247625 (OSUC); OSUC 247597, 247600, 247611, 247620, 317857 (QSBG).

### Oxyscelio magnus

(Kieffer)

urn:lsid:zoobank.org:act:FBE92587-D500-4DEC-979E-B08D842CEF0D

urn:lsid:biosci.ohio-state.edu:osuc_concepts:5026

http://species-id.net/wiki/Oxyscelio_magnus

**Figures 306–311.**
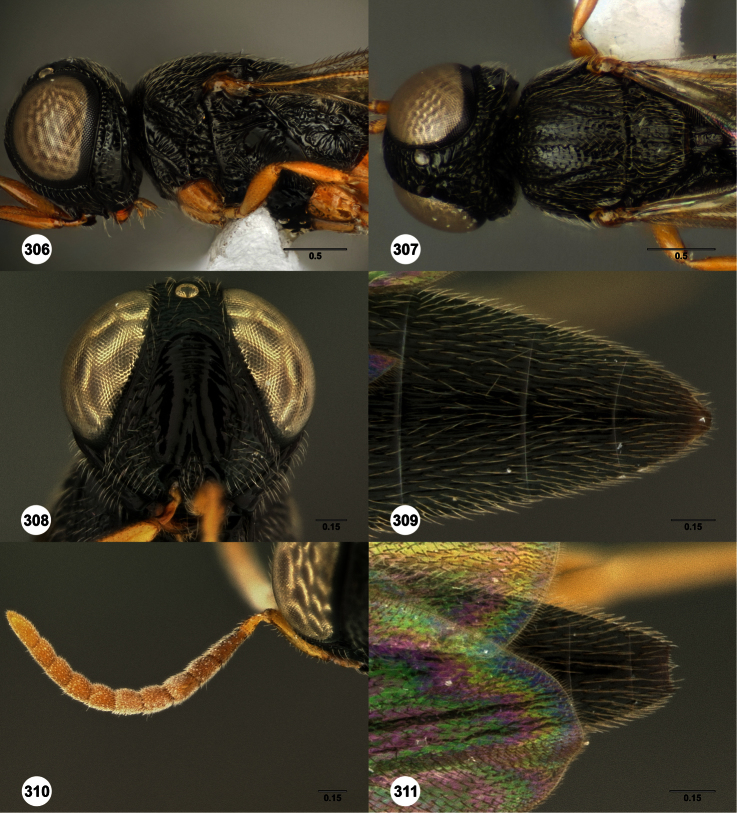
*Oxyscelio magnus* (Kieffer), female (OSUC 257058) **306** Head and mesosoma, lateral view **307** Head and mesosoma, dorsal view. Female (OSUC 257072) **308** Head, anterior view. Female (OSUC 257058) **309** Metasomal apex, dorsal view. Male (OSUC 240934) **310** Antenna **311 **Metasoma, dorsal view. Morphbank^86^

Figures 306–311 Morphbank^86^

Camptoteleia magna Kieffer, 1914: 296 (original description, keyed); Kieffer 1916: 171 (keyed); Kieffer 1926: 380, 382 (description, keyed).
Oxyscelio magnus (Kieffer): Dodd 1931: 76 (generic transfer).

#### Description.

*Female*. Body length 5–5.55 mm (n=8).

Radicle color: same color as scape. Scape color: Yellowish. A4: broader than long. A5: broader than long. Antennal club: formed, segments compact.

Interantennal process: not elongate. Median longitudinal elevation in frontal depression: present. Frontal depression: concave. Frontal depression sculpture: with 3-5 complete transverse carinae. Submedian carina: strong, formed by a sharp raised carina. Submedian carina medially: without peak. Concavity across dorsal part of frontal depression: absent. Depression extending ventrally from median ocellus: present. Upper frons: not hood-like. Malar area near antennal foramen: without carina or expansion. Malar area at mouth corner: with radiating striae. Smooth strip along posterior side of malar sulcus: absent or not consistently broad. Middle genal carina: present. Direction of middle genal carina dorsally: parallel to eye margin. Major sculpture of gena anteriorly: umbilicate-punctate. Major sculpture of gena posteriorly: umbilicate-foveate; rugose. Microsculpture of gena anteroventrally: absent. Microsculpture of gena posteroventrally: absent. Median carina extending posteriorly from hyperoccipital carina: absent. Hyperoccipital carina: indicated by rugae. Lateral connection between hyperoccipital and occipital carinae: absent. Area between vertex and occipital carina: umbilicate-foveate. Occipital carina medially: uniformly rounded. Lateral corners of occipital carina: sharp and protruding.

Lateral pronotal area: without bulge projecting towards anterior pit. Epomial corner: strong. Netrion surface anteriorly: not inflexed. Mesoscutum anteriorly: steep. Mesoscutal median carina: present and complete. Longitudinal carina between median carina and notauli: absent. Major sculpture of medial mesoscutum anteriorly: umbilicate-foveate. Major sculpture of medial mesoscutum posteriorly: umbilicate-foveate. Microsculpture of medial mesoscutum anteriorly: granulate. Microsculpture of medial mesoscutum posteriorly: absent. Major sculpture of mesoscutellum: umbilicate-foveate; longitudinally rugose. Microsculpture of mesoscutellum medially: absent. Microsculpture of mesoscutellum laterally: absent; granulate. Mesoscutellar apex: convex or straight. Setae along anterior limit of femoral depression: arising from rows of foveae. Number of carinae crossing speculum above femoral depression: 2. Number of carinae crossing femoral depression: 3-5. Mesepimeral sulcus pits: 3-5. Metascutellum dorsally: concave. Metascutellar sculpture dorsally: smooth or with transverse carinae. Median carina of metascutellum: absent or branched. Metascutellar setae: absent. Metascutellar apex: convex or straight. Metapleuron above ventral metapleural area: crossed by carinae. Metasomal depression setae: absent. Lateral propodeal carinae anteromedially: strongly diverging. Anterior areoles of metasomal depression: absent. Anterior longitudinal carinae in metasomal depression: absent. Lateral propodeal areas: separated medially. Postmarginal vein: present. Fore wing apex: reaching middle of T5; reaching apex of T5.

T1 midlobe: with 6 or more longitudinal carinae. T1: without anterior bulge. T2: with straight longitudinal striae or rugae. T6: broader than long. Apical flange of T6: exposed apically. Metasomal apex: rounded. Major sculpture of T6: umbilicate-punctate; longitudinally striate or rugose. Microsculpture of T6: granulate.

*Male*. Body length 4.7–5.2 mm (n=3). A5 tyloid: expanded, teardrop-shaped or sinuate. A11: longer than broad. Median tooth of frontal depression: absent. Median lobe of T1: with 5 longitudinal carinae. Metasomal apex: with acuminate lateral corners.

#### Diagnosis.

Both sexes: Frons without flange between antennal foramen and eye; longitudinal carina present along middle of frontal depression; weak impression extending anteriorly from median ocellus. Gena becoming nearly smooth in ventral portion. Mesoscutellum with strong longitudinal rugae in addition to other sculpture. Metascutellum elongate and with a smooth or nearly smooth median channel. Female: A4, A5 broader than long. T1 midlobe with 6-7 longitudinal carinae. T6 broader than long. Male: A11 longer than broad, A12 elongate and tapering to a narrow point. T7 with sharp, protruding posterolateral corners.

#### Link to distribution map.

[http://hol.osu.edu/map-full.html?id=5026]

#### Material examined.

Neotype, female: **PHILIPPINES**: Davao del Sur Prov., Mindanao Isl., Davao, no date, C. F. Baker, OSUC 376667 (deposited in BMNH). *Other material*: (8 females, 4 males) **INDONESIA**: 3 females, OSUC 257058, 257072, 257075 (ROME). **MALAYSIA**: 1 male, OSUC 376744 (MCZC). **PHILIPPINES**: 1 female, 1 male, OSUC 376668-376669 (BMNH). **SINGAPORE**: 2 females, OSUC 376752, 376757 (MCZC). **THAILAND**: 2 females, 1 male, OSUC 368689 (CNCI); OSUC 352524, 361342 (OSUC). **VIETNAM**: 1 male, OSUC 240934 (ROME).

#### Comments.

The type material of *Camptoteleia magna* Kieffer, collected from Mount Makiling, Luzon, in the Philippines, could not be found after an extensive search of collections known to house Kieffer type material. The neotype of *Camptoteleia magna* is presently designated to clarify the taxonomic status of the species. It was selected because of its collection locality and because it resembles Kieffer’s (1914) description in having an elongate metascutellum. *Oxyscelio magnus* under our concept is rarely collected, but is widespread throughout southeast Asia. There is some variation in size and sculpture, most surprisingly in metascutellar size and sculpture medially, but this seems to represent a single species. There are some other Asian species with a similarly elongate metascutellum having a median channel, such as *Oxyscelio aclavae* and *Oxyscelio cyrtomesos*, but both of these species differ from *Oxyscelio magnus* in several significant ways.

### Oxyscelio marginalis

(Kieffer)

urn:lsid:zoobank.org:act:D133686B-181B-4F91-BDE2-11942282B77D

urn:lsid:biosci.ohio-state.edu:osuc_concepts:5027

http://species-id.net/wiki/Oxyscelio_marginalis

**Figures 312–315.**
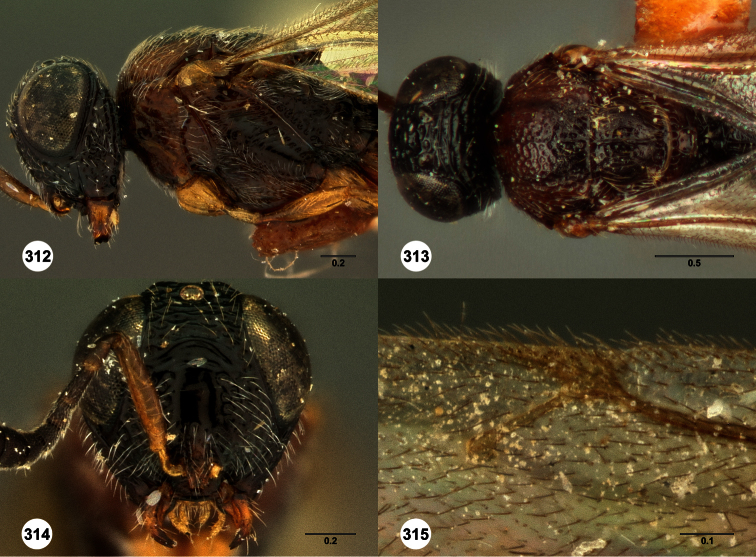
*Oxyscelio marginalis* (Kieffer), neotype female (OSUC 268217) **312** Head and mesosoma, lateral view **313** Head and mesosoma, dorsal view **314** Head, anterior view **315** Fore wing venation, dorsal view. Morphbank^87^

Figures 312–315 Morphbank^87^

Camptoteleia marginalis Kieffer, 1916: 64, 172 (original description, keyed); Kieffer 1926: 380, 385 (description, keyed).
Oxyscelio marginalis (Kieffer): Dodd 1931: 76 (generic transfer).

#### Description.

*Female*. Body length 4.35 mm (n=1).

Radicle color: same color as scape. Scape color: Brown. A4: longer than broad. A5: broader than long. Antennal club: formed, segments compact.

Interantennal process: not elongate. Median longitudinal elevation in frontal depression: absent. Frontal depression: flat. Frontal depression sculpture: with 3 or more broadly interrupted transverse carinae. Submedian carina: weak, shallow and rounded or formed by ledge. Submedian carina medially: without peak. Concavity across dorsal part of frontal depression: absent. Depression extending ventrally from median ocellus: absent. Upper frons: not hood-like. Malar area near antennal foramen: without carina or expansion. Malar area at mouth corner: with radiating striae. Smooth strip along posterior side of malar sulcus: absent or not consistently broad. Middle genal carina: absent. Direction of middle genal carina dorsally: parallel to eye margin. Major sculpture of gena anteriorly: umbilicate-foveate. Major sculpture of gena posteriorly: umbilicate-foveate. Microsculpture of gena anteroventrally: absent. Microsculpture of gena posteroventrally: punctate. Median carina extending posteriorly from hyperoccipital carina: absent. Hyperoccipital carina: indicated by rugae. Lateral connection between hyperoccipital and occipital carinae: absent. Area between vertex and occipital carina: umbilicate-foveate; irregularly rugose. Occipital carina medially: absent. Lateral corners of occipital carina: not protruding.

Lateral pronotal area: without bulge projecting towards anterior pit. Epomial corner: strong. Netrion surface anteriorly: not inflexed. Mesoscutum anteriorly: steep. Mesoscutal median carina: present and complete. Longitudinal carina between median carina and notauli: absent. Major sculpture of medial mesoscutum anteriorly: umbilicate-foveate. Major sculpture of medial mesoscutum posteriorly: umbilicate-foveate. Microsculpture of medial mesoscutum anteriorly: absent. Microsculpture of medial mesoscutum posteriorly: granulate. Major sculpture of mesoscutellum: umbilicate-foveate; umbilicate-punctate. Microsculpture of mesoscutellum medially: absent. Microsculpture of mesoscutellum laterally: granulate. Mesoscutellar apex: convex or straight. Setae along anterior limit of femoral depression: arising from rows of foveae. Number of carinae crossing speculum above femoral depression: 2. Number of carinae crossing femoral depression: 3-5. Mesepimeral sulcus pits: more than 5. Metascutellum dorsally: concave. Metascutellar sculpture dorsally: smooth or with transverse carinae. Median carina of metascutellum: absent or branched. Metascutellar setae: absent. Metascutellar apex: weakly emarginate. Metapleuron above ventral metapleural area: crossed by carinae. Metasomal depression setae: absent. Lateral propodeal carinae anteromedially: weakly diverging. Anterior areoles of metasomal depression: one or more areoles present. Anterior longitudinal carinae in metasomal depression: absent. Lateral propodeal areas: separated medially. Postmarginal vein: present. Fore wing apex: reaching beyond T6.

T1 midlobe: with 5 longitudinal carinae. T1: without anterior bulge. T2: with straight longitudinal striae or rugae. T6: broader than long. Apical flange of T6: exposed apically. Metasomal apex: rounded. Major sculpture of T6: umbilicate-punctate. Microsculpture of T6: granulate.

*Male*. Unknown.

#### Diagnosis.

Female: Frontal depression flat, not margined laterally and with only its dorsal portion well-indicated. Frons without elevation between antennal foramen and eye. Hyperoccipital carina present, continuous with anterior genal carina. Netrion smooth between its anterior and posterior rows of pits. Metascutellum weakly emarginate, subrectangular. Metasomal depression elongate, without median carina; lateral propodeal carinae narrowly separated anteriorly. T1 midlobe with 5 longitudinal carinae. T6 rounded apically.

#### Link to distribution map.

[http://hol.osu.edu/map-full.html?id=5027]

#### Material examined.

Neotype, female: **PHILIPPINES**: Benguet Prov., Luzon Isl., Baguio, no date, Baker, OSUC 268217 (deposited in USNM).

#### Comments.

The type material of *Camptoteleia marginalis* Kieffer, collected from Palawan Island (Puerto Princesa) in the Philippines, could not be found after an extensive search of collections known to house Kieffer type material. The neotype of *Camptoteleia marginalis* is presently designated to clarify the taxonomic status of the species. It was selected because of its collection locality, and because it agrees with Kieffer’s (1916) description in having largely granulate sculpture, a narrow frontal depression that is not laterally carinate, well-separated mandibular teeth, and a thick marginal vein that is nearly as long as the stigmal vein. The neotype female is the only examined specimen from the Philippines that reasonably matches the above characters, and we therefore conclude that Kieffer was mistaken in his account of the postmarginal and stigmal veins in the lost holotype. *Oxyscelio marginalis* is an unusual species within its genus, but agrees with the *cuculli*-group in having a complete hyperoccipital carina that is continuous with an anterior genal carina. The relatively flat frontal depression is apparently a convergent character shared with some other species from the Philippines, and is correlated with reduced surface sculpture. The shape of the lateral propodeal carinae suggests that this species belongs in the *Oxyscelio convergens* species complex within the *cuculli*-group.

### Oxyscelio mesiodentis

Burks sp. n.

urn:lsid:zoobank.org:act:DB58BA09-A681-4F93-8BC4-138E993E54DE

urn:lsid:biosci.ohio-state.edu:osuc_concepts:275559

http://species-id.net/wiki/Oxyscelio_mesiodentis

**Figures 316–321.**
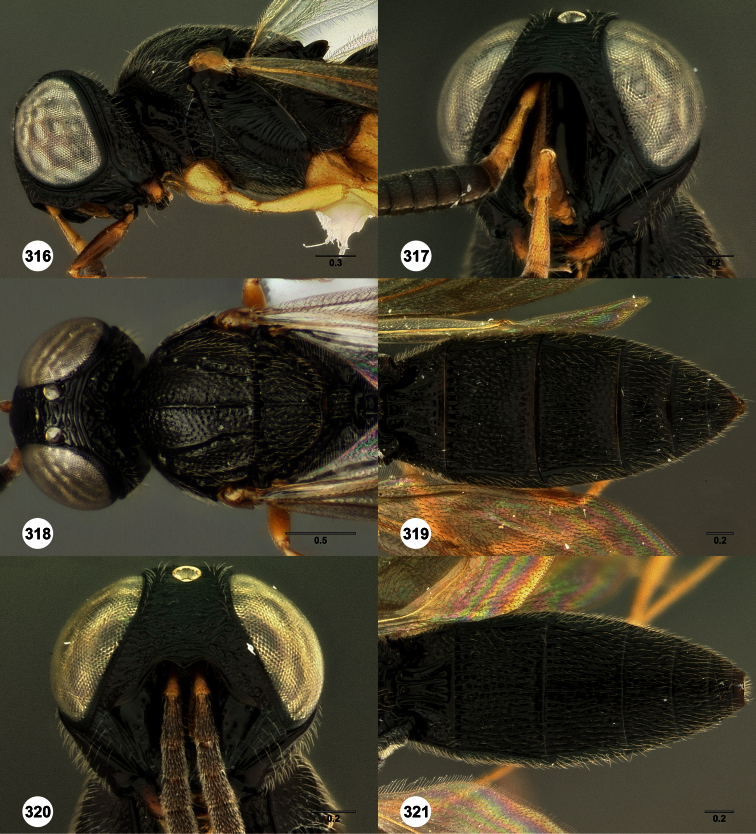
*Oxyscelio mesiodentis* sp. n., holotype female (OSUC 369067) **316** Head and mesosoma, lateral view **317** Head, anterior view. Paratype female (OSUC 257083) **318** Head and mesosoma, dorsal view. Paratype female (OSUC 240926) **319** Metasoma, dorsal view. Paratype male (OSUC 247943) **320** Head, anterior view. Paratype male (OSUC 228736) **321** Metasoma, dorsal view. Morphbank^88^

Figures 316–321 Morphbank^88^

#### Description.

*Female*. Body length 4.35–5.45 mm (n=13).

Radicle color: same color as scape. Scape color: Yellowish; Brown. A4: broader than long. A5: broader than long. Antennal club: formed, segments compact.

Interantennal process: not elongate. Median longitudinal elevation in frontal depression: absent. Frontal depression: concave. Frontal depression sculpture: without transverse or oblique carinae below submedian carina. Submedian carina: strong, formed by a sharp raised carina. Submedian carina medially: without peak. Concavity across dorsal part of frontal depression: absent. Depression extending ventrally from median ocellus: absent. Upper frons: hood-like, protruding over pedicel when antenna at rest. Malar area near antennal foramen: with vertical carina extending from clypeus towards frontal depression. Malar area at mouth corner: with radiating striae. Smooth strip along posterior side of malar sulcus: absent or not consistently broad. Middle genal carina: present. Direction of middle genal carina dorsally: absent (replace with question mark). Major sculpture of gena anteriorly: umbilicate-foveate; rugose. Major sculpture of gena posteriorly: umbilicate-foveate; rugose. Microsculpture of gena anteroventrally: absent. Microsculpture of gena posteroventrally: absent. Median carina extending posteriorly from hyperoccipital carina: absent. Hyperoccipital carina: complete, continuous with anterior genal carina. Lateral connection between hyperoccipital and occipital carinae: absent. Area between vertex and occipital carina: irregularly rugose. Occipital carina medially: uniformly rounded. Lateral corners of occipital carina: not protruding; sharp and protruding.

Lateral pronotal area: with slight bulge projecting anteriorly towards anterior pit. Epomial corner: weak. Netrion surface anteriorly: not inflexed. Mesoscutum anteriorly: steep; not steep. Mesoscutal median carina: present and complete. Longitudinal carina between median carina and notauli: absent. Major sculpture of medial mesoscutum anteriorly: umbilicate-foveate. Major sculpture of medial mesoscutum posteriorly: umbilicate-foveate. Microsculpture of medial mesoscutum anteriorly: granulate. Microsculpture of medial mesoscutum posteriorly: absent. Major sculpture of mesoscutellum: umbilicate-foveate; longitudinally rugose. Microsculpture of mesoscutellum medially: absent. Microsculpture of mesoscutellum laterally: absent. Mesoscutellar apex: convex or straight. Setae along anterior limit of femoral depression: arising from rows of foveae. Number of carinae crossing speculum above femoral depression: 3. Number of carinae crossing femoral depression: more than 5. Mesepimeral sulcus pits: more than 5. Metascutellum dorsally: concave. Metascutellar sculpture dorsally: foveate. Median carina of metascutellum: absent or branched. Metascutellar setae: with many dorsal setae. Metascutellar apex: weakly emarginate. Metapleuron above ventral metapleural area: crossed by carinae. Metasomal depression setae: absent. Lateral propodeal carinae anteromedially: weakly diverging. Anterior areoles of metasomal depression: one or more areoles present. Anterior longitudinal carinae in metasomal depression: absent. Lateral propodeal areas: separated medially. Postmarginal vein: present. Fore wing apex: reaching apex of T6. 1st metatarsomere over 1.1x as long as metatarsomeres 2–5 combined.

T1 midlobe: with 4 longitudinal carinae. T1: without anterior bulge. T2: with straight longitudinal striae or rugae. T6: broader than long. Apical flange of T6: exposed apically. Metasomal apex: rounded. Major sculpture of T6: umbilicate-punctate; longitudinally striate or rugose. Microsculpture of T6: absent.

*Male*.Body length 3.5–4.95 mm (n=20). A5 tyloid: carina-like, not expanded. A11: longer than broad. Median tooth of frontal depression: present. Median lobe of T1: with 4 longitudinal carinae. Metasomal apex: with acuminate lateral corners.

#### Diagnosis.

A4, A5 broader than long. Face with vertical elevation between antennal foramen and eye. Hyperoccipital carina present, continuous with anterior genal carina. Medial mesoscutum and mesoscutellum with many strong longitudinal rugae. Metascutellum with dorsal setae. Female: T1 with 4 longitudinal carinae. Male: A5 tyloid expanded. Frontal depression with tooth-like median protrusion dorsally. T1 midlobe with 3 longitudinal carinae. T7 without distinct posterolateral corners.

#### Etymology.

Latin noun, genitive case, after the dental term mesiodens, the condition of having a supernumerary middle tooth. Refers to the middle tooth of the frontal depression in males.

#### Link to distribution map.

[http://hol.osu.edu/map-full.html?id=275559]

#### Material Examined.

Holotype, female: **MALAYSIA**: Sabah St., Borneo Isl., Kinabalu National Park, VIII-1999, malaise trap, D. Quicke, OSUC 369067 (deposited in BMNH). *Paratypes*: (18 females, 40 males) **INDONESIA**: 4 females, 14 males, OSUC 368945 (CNCI); OSUC 228724, 247934, 247943, 251425, 257031, 257083 (MBBJ); OSUC 240926 (OSUC); OSUC 228715, 228720, 228723, 228736, 247828, 247830, 247844, 247939, 247941, ROMEnt Spec. No. 112253 (ROME). **LAOS**: 1 female, 3 males, OSUC 368876, 368886, 368891, 368909 (CNCI). **MALAYSIA**: 2 females, 1 male, OSUC 369027 (CNCI); OSUC 448588-448589 (WINC). **NEPAL**: 3 males, OSUC 369168, 369170, 369175 (CNCI). **THAILAND**: 11 females, 21 males, OSUC 335862 (BMNH); OSUC 368615, 368650, 368687, 368698-368701, 368705, 368712, 368714-368715, 368729, 368746-368747 (CNCI); OSUC 319481, 335856, 336122, 352481, 352917, 361220, 361954, 368547 (OSUC); OSUC 257384, 320402, 335229, 335829, 336160, 336162, 368546, 404913 (QSBG); UCRC ENT 135264 (UCRC).

#### Comments.

The vertical elevation between the antennal foramen and eye in *Oxyscelio mesiodentis* and similar species bears a granulate patch. Some males from Borneo and Laos were intermediate in these features, but could not be associated with females. These were left unassigned to species until corresponding females can be found. While *Oxyscelio mesiodentis* and *Oxyscelio brevidentis* may represent variant forms of the same species, the differences in metatarsomere length indicate that they are kept separate pending new information.

### Oxyscelio mollitia

Burks sp. n.

urn:lsid:zoobank.org:act:E395DF2E-A099-4761-8E00-A6FDA6873030

urn:lsid:biosci.ohio-state.edu:osuc_concepts:275526

http://species-id.net/wiki/Oxyscelio_mollitia

**Figures 322–326.**
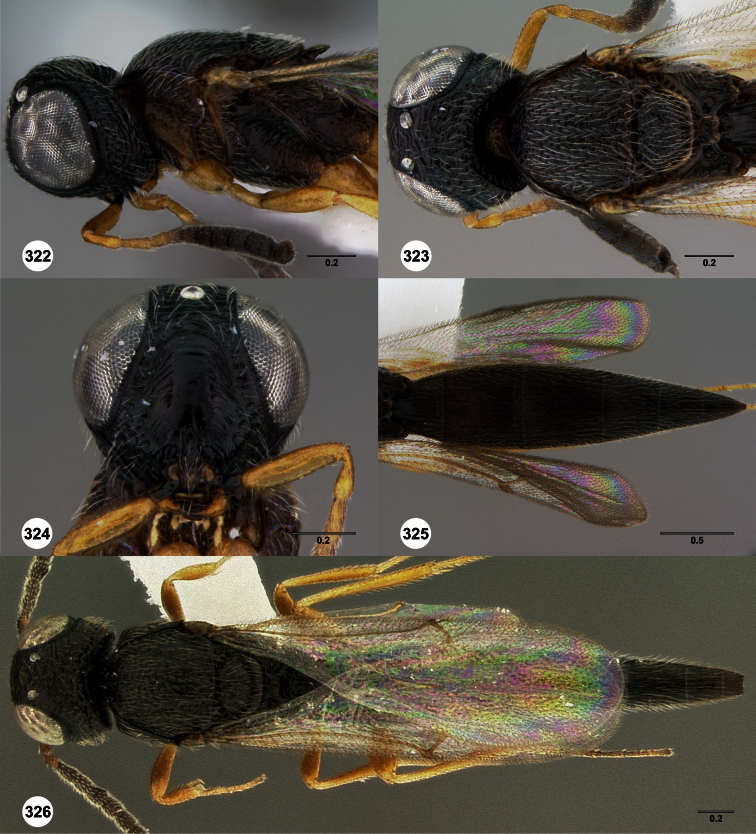
*Oxyscelio mollitia* sp. n., holotype female (OSUC 368975) **322** Head and mesosoma, lateral view **323** Head and mesosoma, dorsal view **324** Head, anterior view **325** Metasoma, dorsal view. Paratype male (OSUC 368987) **326** Body, dorsal view. Morphbank^89^

Figures 322–326 Morphbank^89^

#### Description.

*Female*. Body length 3.8–4.4 mm (n=17).

Radicle color: darker than scape. Scape color: Yellowish. A4: longer than broad. A5: longer than broad. Antennal club: formed, segments compact.

Interantennal process: not elongate. Median longitudinal elevation in frontal depression: absent. Frontal depression: concave. Frontal depression sculpture: with 3-5 complete transverse carinae. Submedian carina: strong, formed by a sharp raised carina. Submedian carina medially: without peak. Concavity across dorsal part of frontal depression: absent. Depression extending ventrally from median ocellus: absent. Upper frons: not hood-like. Malar area near antennal foramen: without carina or expansion. Malar area at mouth corner: with radiating striae. Smooth strip along posterior side of malar sulcus: absent or not consistently broad. Middle genal carina: present. Direction of middle genal carina dorsally: parallel to eye margin. Major sculpture of gena anteriorly: umbilicate-foveate. Major sculpture of gena posteriorly: rugose; umbilicate-punctate. Microsculpture of gena anteroventrally: absent. Microsculpture of gena posteroventrally: absent. Median carina extending posteriorly from hyperoccipital carina: absent. Hyperoccipital carina: indicated by rugae. Lateral connection between hyperoccipital and occipital carinae: absent. Area between vertex and occipital carina: umbilicate-foveate. Occipital carina medially: uniformly rounded. Lateral corners of occipital carina: not protruding.

Lateral pronotal area: without bulge projecting towards anterior pit. Epomial corner: weak. Netrion surface anteriorly: not inflexed. Mesoscutum anteriorly: not steep. Mesoscutal median carina: present and complete. Longitudinal carina between median carina and notauli: absent. Major sculpture of medial mesoscutum anteriorly: umbilicate-foveate. Major sculpture of medial mesoscutum posteriorly: umbilicate-foveate. Microsculpture of medial mesoscutum anteriorly: granulate. Microsculpture of medial mesoscutum posteriorly: granulate. Major sculpture of mesoscutellum: umbilicate-foveate. Microsculpture of mesoscutellum medially: absent. Microsculpture of mesoscutellum laterally: absent. Mesoscutellar apex: convex or straight. Setae along anterior limit of femoral depression: arising from rows of foveae. Number of carinae crossing speculum above femoral depression: 3. Number of carinae crossing femoral depression: more than 5. Mesepimeral sulcus pits: more than 5. Metascutellum dorsally: concave. Metascutellar sculpture dorsally: smooth or with transverse carinae. Median carina of metascutellum: absent or branched. Metascutellar setae: absent. Metascutellar apex: weakly emarginate. Metapleuron above ventral metapleural area: smooth. Metasomal depression setae: absent. Lateral propodeal carinae anteromedially: strongly diverging. Anterior areoles of metasomal depression: absent. Anterior longitudinal carinae in metasomal depression: absent. Lateral propodeal areas: separated medially. Postmarginal vein: present. Fore wing apex: reaching middle of T5.

T1 midlobe: obscured by other raised sculpture. T1: with small rounded anterior bulge, not reaching metascutellum. T2: with straight longitudinal striae or rugae. T6: longer than broad. Apical flange of T6: exposed apically. Metasomal apex: rounded. Major sculpture of T6: umbilicate-punctate; longitudinally striate or rugose. Microsculpture of T6: granulate.

*Male*. Body length 4.3 mm (n=1). A5 tyloid: carina-like, not expanded. A11: longer than broad. Median tooth of frontal depression: absent. Median lobe of T1: with 5 longitudinal carinae. Metasomal apex: with acuminate lateral corners.

#### Diagnosis.

Both sexes: Upper frons without additional carinae dorsal to submedian carina. Hyperoccipital carina indicated by rugae. Mesoscutellum without granulate sculpture. Mesofemoral depression crossed by more than 3 carinae below speculum. Mesopleuron along anteroventral edge of femoral depression with rows of foveae. Female: Metascutellum subrectangular, with scattered rugae. T1 midlobe with weak anterior bulge. T2 without sublateral depressions or curved striae. Fore wing long enough to reach middle of T5. T6 longer than broad, tapering to a rounded apex. Male: Flagellomeres longer than broad. T1 midlobe with 5 longitudinal carinae. T7 with sharp, protruding posterolateral corners. *Oxyscelio mollitia* is similar to *Oxyscelio florus*, another Palearctic species. It differs in having one or more rows of foveae along the anterior limit of the femoral depression, a transversely carinate metascutellum, a usually shorter anterior horn on the T1 midlobe, and shorter metasoma in females (fore wing long enough to reach middle of T5).

#### Etymology.

Latin noun in apposition, meaning “flexibility.” Refers to the tendency of various body parts to buckle when specimens of this species are dried.

#### Link to distribution map.

[http://hol.osu.edu/map-full.html?id=275526]

#### Material examined.

Holotype, female: **JAPAN**: Aichi Pref, Toyota City, Narai, 15.VII-22.VII.1990, pan trap, K. Yamagishi, OSUC 368975 (deposited in CNCI). *Paratypes*: (17 females, 1 male) **JAPAN**: 12 females, 1 male, OSUC 368964, 368966-368967, 368980, 368987, 368991, 368994, 368998-369000, 369007 (CNCI); OSUC 369001, 369008 (OSUC). **SOUTH KOREA**: 5 females, OSUC 369069-369073 (CNCI).

#### Comments.

The submedian carina in *Oxyscelio mollitia* is not accompanied by any additional carinae dorsal to it, but this part of the upper frons can have weak, transverse rugae with granulate sculpture between them.

### Oxyscelio naraws

Kozlov & Lê

urn:lsid:zoobank.org:act:377E4C35-6CDC-4D4E-9C7B-537662DFB8F2

urn:lsid:biosci.ohio-state.edu:osuc_concepts:179750

http://species-id.net/wiki/Oxyscelio_naraws

**Figures 327–332.**
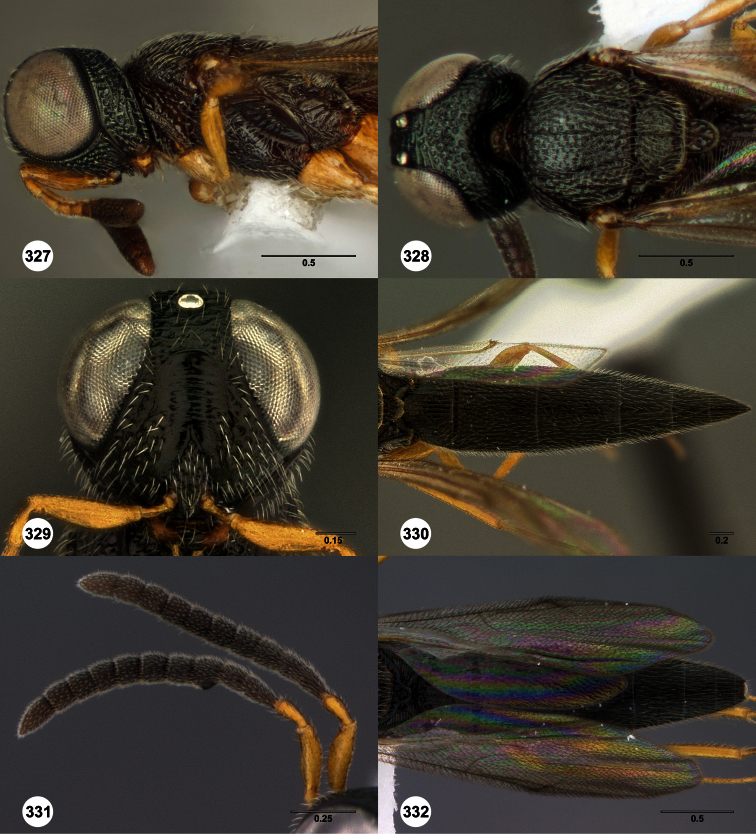
*Oxyscelio naraws* Kozlov & Lê, female (OSUC 257093) **327** Head and mesosoma, lateral view **328** Head and mesosoma, dorsal view. Female (OSUC 335201) **329** Head, anterior view. Female (OSUC 368961) **330** Metasoma, dorsal view. Paratype male (OSUC 361343) **331** Antenna **332 **Metasoma, dorsal view. Morphbank^90^

Figures 327–332 Morphbank^90^

Oxyscelio naraws Kozlov & Lê, 2000: 40, 326 (original description).

#### Description.

*Female*. Body length 3.75–5.45 mm (n=20).

Radicle color: same color as scape. Scape color: Yellowish. A4: longer than broad; as long as broad. A5: broader than long; longer than broad; as long as broad. Antennal club: formed, segments compact.

Interantennal process: not elongate. Median longitudinal elevation in frontal depression: absent. Frontal depression: concave. Frontal depression sculpture: with 3-5 complete transverse carinae. Submedian carina: strong, formed by a sharp raised carina. Submedian carina medially: without peak. Concavity across dorsal part of frontal depression: absent. Depression extending ventrally from median ocellus: absent. Upper frons: not hood-like. Malar area near antennal foramen: without carina or expansion. Malar area at mouth corner: with radiating striae. Smooth strip along posterior side of malar sulcus: absent or not consistently broad. Middle genal carina: present. Direction of middle genal carina dorsally: parallel to eye margin. Major sculpture of gena anteriorly: umbilicate-foveate. Major sculpture of gena posteriorly: umbilicate-foveate; rugose. Microsculpture of gena anteroventrally: absent; granulate. Microsculpture of gena posteroventrally: absent; granulate. Median carina extending posteriorly from hyperoccipital carina: absent. Hyperoccipital carina: not indicated medially. Lateral connection between hyperoccipital and occipital carinae: absent. Area between vertex and occipital carina: umbilicate-foveate. Occipital carina medially: absent. Lateral corners of occipital carina: sharp and protruding.

Lateral pronotal area: without bulge projecting towards anterior pit. Epomial corner: strong. Netrion surface anteriorly: not inflexed. Mesoscutum anteriorly: not steep. Mesoscutal median carina: present and complete. Longitudinal carina between median carina and notauli: absent. Major sculpture of medial mesoscutum anteriorly: umbilicate-foveate. Major sculpture of medial mesoscutum posteriorly: umbilicate-foveate. Microsculpture of medial mesoscutum anteriorly: granulate. Microsculpture of medial mesoscutum posteriorly: absent. Major sculpture of mesoscutellum: umbilicate-foveate. Microsculpture of mesoscutellum medially: absent; granulate. Microsculpture of mesoscutellum laterally: granulate. Mesoscutellar apex: convex or straight. Setae along anterior limit of femoral depression: arising from rows of foveae; arising from tiny pits. Number of carinae crossing speculum above femoral depression: 2. Number of carinae crossing femoral depression: more than 5. Mesepimeral sulcus pits: more than 5. Metascutellum dorsally: flat. Metascutellar sculpture dorsally: with scattered rugae. Median carina of metascutellum: absent or branched; straight, unbranched carina present. Metascutellar setae: absent. Metascutellar apex: convex or straight. Metapleuron above ventral metapleural area: crossed by carinae. Metasomal depression setae: absent. Lateral propodeal carinae anteromedially: strongly diverging. Anterior areoles of metasomal depression: absent. Anterior longitudinal carinae in metasomal depression: absent. Lateral propodeal areas: separated medially. Postmarginal vein: absent. Fore wing apex: reaching apex of T4; reaching middle of T5.

T1 midlobe: obscured by other raised sculpture. T1: with small rounded anterior bulge, not reaching metascutellum. T2: with straight longitudinal striae or rugae. T6: longer than broad. Apical flange of T6: not exposed apically. Metasomal apex: rounded. Major sculpture of T6: umbilicate-punctate; longitudinally striate or rugose. Microsculpture of T6: absent; granulate.

*Male*.Body length 3.3–4.3 mm (n=7). A5 tyloid: carina-like, not expanded. A11: broader than long. Median tooth of frontal depression: absent. Median lobe of T1: with 5 longitudinal carinae. Metasomal apex: with acuminate lateral corners.

#### Diagnosis.

Both sexes: Frontal depression crossed by many carinae. Mesoscutellum strongly granulate. Metascutellum dorsally bare. Female: Metascutellum fingernail-shaped, rugose. T1 with strong anterior horn. T6 strongly narrowing towards nearly acuminate apex. Male: T1 midlobe with 5 longitudinal carinae. T7 with sharp, protruding posterolateral corners.

#### Link to distribution map.

[http://hol.osu.edu/map-full.html?id=179750]

#### Material examined.

*Other material*: (32 females, 7 males) **BRUNEI**: 1 female, OSUC 376628 (BMNH). **INDONESIA**: 4 females, OSUC 368961 (CNCI); OSUC 247838, 248920, 257093 (ROME). **MALAYSIA**: 12 females, OSUC 376579, 376612 (BMNH); OSUC 369031, 369035-369036, 369040, 463996 (CNCI); OSUC 268183-268184, 268186-268187, 268210 (USNM). **THAILAND**: 15 females, 7 males, OSUC 247643, 320373, 320412, 335201, 335203, 335838, 335863, 335914, 335927, 336030, 336033, 336035, 336121, 352502-352504, 361334, 361336, 361338, 361343, 368533, 368538 (OSUC).

#### Comments.

*Oxyscelio naraws* exhibits a wide range of variation in the lengths of A4, A5, the metascutellum, and the metasoma in females. This may indicate that it represents a suite of very similar species, but no convincing consistent features were found to support any separation.

### Oxyscelio nasolabii

Burks sp. n.

urn:lsid:zoobank.org:act:2F28C6E0-F926-4350-966C-EB8B4344D118

urn:lsid:biosci.ohio-state.edu:osuc_concepts:275525

http://species-id.net/wiki/Oxyscelio_nasolabii

**Figures 333–336.**
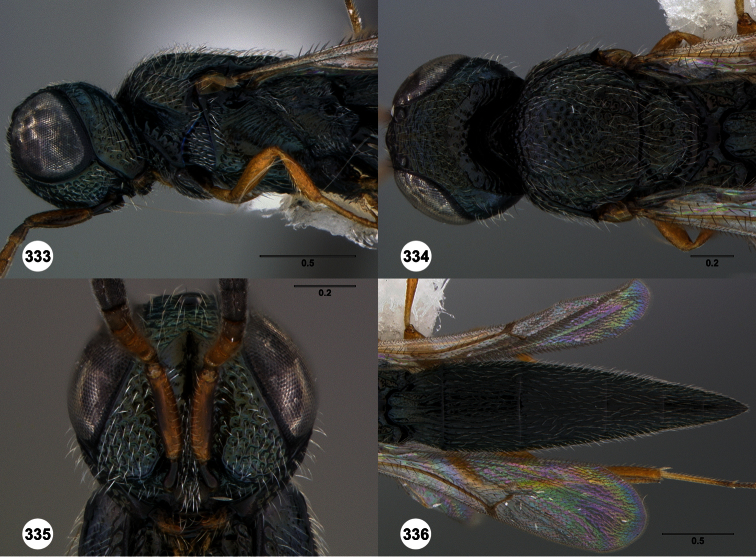
*Oxyscelio nasolabii* sp. n., holotype female (OSUC 369096) **333** Head and mesosoma, lateral view **334** Head and mesosoma, dorsal view **335** Head, anterior view **336** Metasoma, dorsal view. Morphbank^91^

Figures 333–336 Morphbank^91^

#### Description.

*Female*. Body length 4.35 mm (n=1).

Radicle color: darker than scape. Scape color: Brown. A4: longer than broad. A5: longer than broad. Antennal club: formed, segments compact.

Interantennal process: not elongate. Median longitudinal elevation in frontal depression: absent. Frontal depression: concave. Frontal depression sculpture: with 3 or more broadly interrupted transverse carinae. Submedian carina: weak, shallow and rounded or formed by ledge. Submedian carina medially: with sharp peak. Concavity across dorsal part of frontal depression: absent. Depression extending ventrally from median ocellus: absent. Upper frons: not hood-like. Malar area near antennal foramen: with long ruga extending from edge of frontal depression. Malar area at mouth corner: with radiating striae. Smooth strip along posterior side of malar sulcus: present, broad throughout its length. Middle genal carina: present. Direction of middle genal carina dorsally: parallel to eye margin. Major sculpture of gena anteriorly: umbilicate-foveate; rugose. Major sculpture of gena posteriorly: umbilicate-foveate; rugose. Microsculpture of gena anteroventrally: granulate. Microsculpture of gena posteroventrally: absent. Median carina extending posteriorly from hyperoccipital carina: absent. Hyperoccipital carina: indicated by rugae. Lateral connection between hyperoccipital and occipital carinae: present as a weak elevation. Area between vertex and occipital carina: umbilicate-foveate; irregularly rugose. Occipital carina medially: uniformly rounded. Lateral corners of occipital carina: sharp and protruding.

Lateral pronotal area: without bulge projecting towards anterior pit. Epomial corner: strong. Netrion surface anteriorly: not inflexed. Mesoscutum anteriorly: not steep. Mesoscutal median carina: absent or weak and incomplete in places. Longitudinal carina between median carina and notauli: absent. Major sculpture of medial mesoscutum anteriorly: umbilicate-foveate. Major sculpture of medial mesoscutum posteriorly: umbilicate-punctate. Microsculpture of medial mesoscutum anteriorly: granulate. Microsculpture of medial mesoscutum posteriorly: absent. Major sculpture of mesoscutellum: umbilicate-foveate. Microsculpture of mesoscutellum medially: granulate. Microsculpture of mesoscutellum laterally: granulate. Mesoscutellar apex: convex or straight. Setae along anterior limit of femoral depression: arising from rows of foveae. Number of carinae crossing speculum above femoral depression: 4. Number of carinae crossing femoral depression: more than 5. Mesepimeral sulcus pits: more than 5. Metascutellum dorsally: concave. Metascutellar sculpture dorsally: smooth or with transverse carinae. Median carina of metascutellum: straight, unbranched carina present. Metascutellar setae: absent. Metascutellar apex: weakly emarginate. Metapleuron above ventral metapleural area: crossed by carinae. Metasomal depression setae: absent. Lateral propodeal carinae anteromedially: strongly diverging. Anterior areoles of metasomal depression: absent. Anterior longitudinal carinae in metasomal depression: absent. Lateral propodeal areas: separated medially. Postmarginal vein: absent. Fore wing apex: reaching middle of T4.

T1 midlobe: obscured by other raised sculpture. T1: with small rounded anterior bulge, not reaching metascutellum. T2: with straight longitudinal striae or rugae. T6: longer than broad. Apical flange of T6: not exposed apically. Metasomal apex: rounded. Major sculpture of T6: umbilicate-punctate. Microsculpture of T6: granulate.

*Male*. Unknown.

#### Diagnosis.

Female: A4, A5 not longer than broad. Radicle darker than scape. Face with long expanded flange between antennal foramen and eye. Mesoscutum and mesoscutellum without median carina. Metascutellum broad but smooth medially. T1 midlobe elevated but with 3 strong carinae. T2 without sublateral depressions or curved striae.T6 longer than broad.

#### Etymology.

Latin noun, genitive case, intended to mean “nasolabial fold.” Refers to the similarity of the ridge between the antennal foramen and eye to the nasolabial fold of the human face.

#### Link to distribution map.

[http://hol.osu.edu/map-full.html?id=275525]

#### Material examined.

Holotype, female: **VIETNAM**: Ha Tinh Prov., Huong Son, 18°22'N, 105°13'E, 900m, 18.V-28.V.1998, malaise trap, L. Herman, OSUC 369096 (deposited in AMNH).

#### Comments.

*Oxyscelio nasolabii* is unusual within its genus in having a medially smooth metascutellum but lacking median carinae of the mesoscutum and mesoscutellum. The coxae of the holotype are darker than other parts of the legs, but this character is variable in Asian species.

### Oxyscelio nodorum

Burks sp. n.

urn:lsid:zoobank.org:act:98493786-C388-4851-98EB-B126F85735B3

urn:lsid:biosci.ohio-state.edu:osuc_concepts:275523

http://species-id.net/wiki/Oxyscelio_nodorum

**Figures 337–340.**
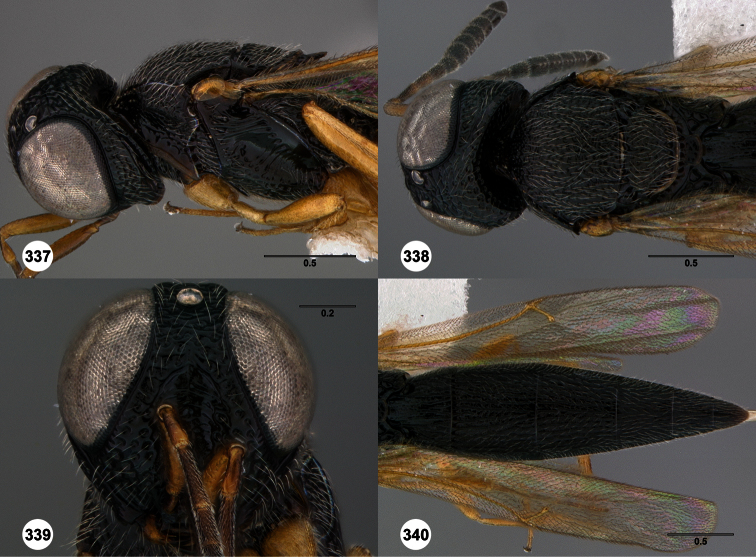
*Oxyscelio nodorum* sp. n., holotype female (OSUC 369217) **337** Head and mesosoma, lateral view **338** Head and mesosoma, dorsal view **339** Head, anterior view **340** Metasoma, dorsal view. Morphbank^92^

Figures 337–340 Morphbank^92^

#### Description.

*Female*. Body length 4.65 mm (n=1).

Radicle color: same color as scape. Scape color: Brown. A4: longer than broad. A5: longer than broad. Antennal club: formed, segments compact.

Interantennal process: not elongate. Median longitudinal elevation in frontal depression: absent. Frontal depression: concave. Frontal depression sculpture: crossed by many tiny furrows. Submedian carina: weak, shallow and rounded or formed by ledge. Submedian carina medially: without peak. Concavity across dorsal part of frontal depression: absent. Depression extending ventrally from median ocellus: absent. Upper frons: not hood-like. Malar area near antennal foramen: without carina or expansion. Malar area at mouth corner: with radiating striae. Smooth strip along posterior side of malar sulcus: absent or not consistently broad. Middle genal carina: present. Direction of middle genal carina dorsally: parallel to eye margin. Major sculpture of gena anteriorly: umbilicate-foveate. Major sculpture of gena posteriorly: rugose; umbilicate-punctate. Microsculpture of gena anteroventrally: absent. Microsculpture of gena posteroventrally: absent. Median carina extending posteriorly from hyperoccipital carina: absent. Hyperoccipital carina: indicated by rugae. Lateral connection between hyperoccipital and occipital carinae: absent. Area between vertex and occipital carina: umbilicate-foveate. Occipital carina medially: uniformly rounded. Lateral corners of occipital carina: not protruding.

Lateral pronotal area: without bulge projecting towards anterior pit. Epomial corner: weak. Netrion surface anteriorly: not inflexed. Mesoscutum anteriorly: not steep. Mesoscutal median carina: absent or weak and incomplete in places. Longitudinal carina between median carina and notauli: absent. Major sculpture of medial mesoscutum anteriorly: umbilicate-foveate. Major sculpture of medial mesoscutum posteriorly: umbilicate-foveate. Microsculpture of medial mesoscutum anteriorly: granulate. Microsculpture of medial mesoscutum posteriorly: absent. Major sculpture of mesoscutellum: umbilicate-foveate; irregularly rugose. Microsculpture of mesoscutellum medially: absent. Microsculpture of mesoscutellum laterally: granulate. Mesoscutellar apex: convex or straight. Setae along anterior limit of femoral depression: arising from rows of foveae. Number of carinae crossing speculum above femoral depression: 3. Number of carinae crossing femoral depression: 3-5. Mesepimeral sulcus pits: more than 5. Metascutellum dorsally: concave. Metascutellar sculpture dorsally: smooth or with transverse carinae. Median carina of metascutellum: absent or branched. Metascutellar setae: absent. Metascutellar apex: convex or straight. Metapleuron above ventral metapleural area: crossed by carinae. Metasomal depression setae: absent. Lateral propodeal carinae anteromedially: strongly diverging. Anterior areoles of metasomal depression: absent. Anterior longitudinal carinae in metasomal depression: absent. Lateral propodeal areas: separated medially. Postmarginal vein: present. Fore wing apex: reaching apex of T5.

T1 midlobe: obscured by other raised sculpture. T1: with small rounded anterior bulge, not reaching metascutellum. T2: with straight longitudinal striae or rugae. T6: longer than broad. Apical flange of T6: exposed apically. Metasomal apex: rounded. Major sculpture of T6: umbilicate-punctate. Microsculpture of T6: granulate.

*Male*. Unknown.

#### Diagnosis.

Female: A4, A5 longer than broad. Frontal depression narrow and crossed by many (>3) carinae. Hyperoccipital carina indicated by rugae; occipital carina without strong lateral corners. Mesoscutellum granulate laterally. Metascutellum smooth and subrectangular, without rugae but with an incomplete median carina. Fore wings long enough to reach middle of T6. T1 midlobe with a strong anterior horn. T6 longer than broad, rounded apically.

#### Etymology.

Latin noun, genitive case, meaning “a fishing net.”

#### Link to distribution map.

[http://hol.osu.edu/map-full.html?id=275523]

#### Material examined.

Holotype, female: **INDONESIA**: Sulawesi Utara Prov., Lake Danau, canopy, Kotamobagu, 1200m, 31.VII.1985, N. E. Stork, OSUC 369217 (deposited in BMNH).

#### Comments.

*Oxyscelio nodorum* is unusual because of the long and narrow T1 horn and strongly sculptured frontal depression in combination with a smooth metascutellum.

### Oxyscelio noduli

Burks sp. n.

urn:lsid:zoobank.org:act:E98B4D52-B483-405C-BA3B-A3F2E5E1C259

urn:lsid:biosci.ohio-state.edu:osuc_concepts:275517

http://species-id.net/wiki/Oxyscelio_noduli

**Figures 341–344.**
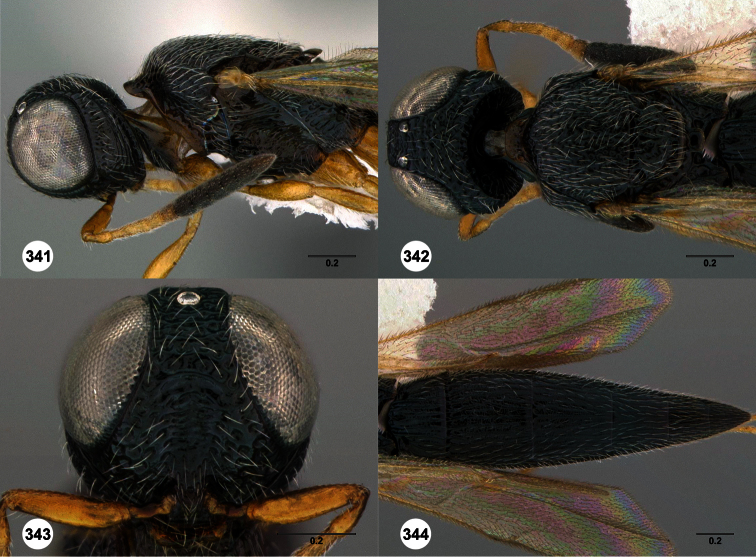
*Oxyscelio noduli* sp. n., holotype female (OSUC 369234) **341** Head and mesosoma, lateral view **342** Head and mesosoma, dorsal view **343** Head, anterior view **344** Metasoma, dorsal view. Morphbank^93^

Figures 341–344 Morphbank^93^

#### Description.

*Female*. Body length 3.25 mm (n=1).

Radicle color: same color as scape. Scape color: Yellowish. A4: longer than broad. A5: longer than broad. Antennal club: formed, segments compact.

Interantennal process: not elongate. Median longitudinal elevation in frontal depression: absent. Frontal depression: concave. Frontal depression sculpture: crossed by many tiny furrows. Submedian carina: weak, shallow and rounded or formed by ledge. Submedian carina medially: without peak. Concavity across dorsal part of frontal depression: absent. Depression extending ventrally from median ocellus: absent. Upper frons: not hood-like. Malar area near antennal foramen: without carina or expansion. Malar area at mouth corner: with radiating striae. Smooth strip along posterior side of malar sulcus: absent or not consistently broad. Middle genal carina: present. Direction of middle genal carina dorsally: parallel to eye margin. Major sculpture of gena anteriorly: umbilicate-foveate. Major sculpture of gena posteriorly: rugose. Microsculpture of gena anteroventrally: absent. Microsculpture of gena posteroventrally: granulate. Median carina extending posteriorly from hyperoccipital carina: absent. Hyperoccipital carina: indicated by rugae. Lateral connection between hyperoccipital and occipital carinae: absent. Area between vertex and occipital carina: umbilicate-foveate. Occipital carina medially: uniformly rounded. Lateral corners of occipital carina: not protruding.

Lateral pronotal area: without bulge projecting towards anterior pit. Epomial corner: strong. Netrion surface anteriorly: not inflexed. Mesoscutum anteriorly: not steep. Mesoscutal median carina: present and complete. Longitudinal carina between median carina and notauli: absent. Major sculpture of medial mesoscutum anteriorly: umbilicate-foveate. Major sculpture of medial mesoscutum posteriorly: umbilicate-foveate. Microsculpture of medial mesoscutum anteriorly: granulate. Microsculpture of medial mesoscutum posteriorly: absent. Major sculpture of mesoscutellum: umbilicate-foveate. Microsculpture of mesoscutellum medially: absent. Microsculpture of mesoscutellum laterally: granulate. Mesoscutellar apex: convex or straight. Setae along anterior limit of femoral depression: arising from rows of foveae. Number of carinae crossing speculum above femoral depression: 4. Number of carinae crossing femoral depression: more than 5. Mesepimeral sulcus pits: more than 5. Metascutellum dorsally: concave. Metascutellar sculpture dorsally: smooth or with transverse carinae. Median carina of metascutellum: absent or branched. Metascutellar setae: absent. Metascutellar apex: convex or straight. Metapleuron above ventral metapleural area: foveate or rugose. Metasomal depression setae: absent. Lateral propodeal carinae anteromedially: strongly diverging. Anterior areoles of metasomal depression: absent. Anterior longitudinal carinae in metasomal depression: absent. Lateral propodeal areas: separated medially. Postmarginal vein: present. Fore wing apex: reaching middle of T5.

T1 midlobe: with 6 or more longitudinal carinae. T1: with small rounded anterior bulge, not reaching metascutellum. T2: with straight longitudinal striae or rugae. T6: longer than broad. Apical flange of T6: exposed apically. Metasomal apex: rounded. Major sculpture of T6: umbilicate-punctate. Microsculpture of T6: granulate.

*Male*. Unknown.

#### Diagnosis.

Female: A4 longer than broad, A5 broader than long. Frontal depression narrow and crossed by many (>3) carinae. Hyperoccipital carina indicated by rugae; occipital carina without strong lateral corners. Mesoscutellum granulate laterally. Metascutellum smooth and subrectangular, without rugae. Fore wings long enough to reach middle of T5. T1 midlobe with an anterior smooth area but without a horn. T6 longer than broad, rounded apically. *Oxyscelio noduli* is similar to *Oxyscelio nodorum*, but is much smaller and exhibits only a very small smooth area anteriorly on T1, instead of a long horn.

#### Etymology.

Latin noun, genitive case, meaning “little knot.”

#### Link to distribution map.

[http://hol.osu.edu/map-full.html?id=275517]

#### Material examined.

Holotype, female: **INDONESIA**: Sulawesi Utara Prov., Toraut, Bogani Nani Wartabone (Dumoga-Bone) National Park, 220m, 9.V-16.V.1985, J. S. Noyes, OSUC 369234 (deposited in BMNH).

#### Comments.

Many of the striae on the metasomal terga of *Oxyscelio noduli* have flat, granulate tops.

### Oxyscelio nubbin

Burks sp. n.

urn:lsid:zoobank.org:act:3FFB3E2E-CC40-4A8D-970A-2F1374E38A99

urn:lsid:biosci.ohio-state.edu:osuc_concepts:275480

http://species-id.net/wiki/Oxyscelio_nubbin

**Figures 345–350.**
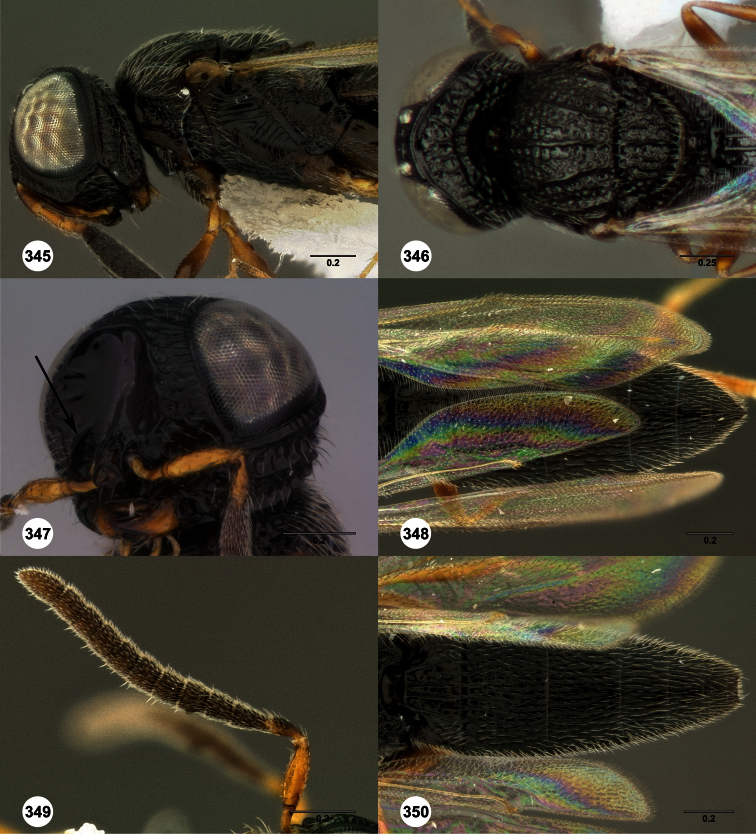
*Oxyscelio nubbin* sp. n., paratype female (OSUC 361932) **345** Head and mesosoma, lateral view. Paratype female (OSUC 252043) **346** Head and mesosoma, dorsal view. Paratype female (OSUC 265260) **347** Head, oblique view; arrow indicates oblique flange. Paratype female (OSUC 361190) **348** Metasoma, dorsal view. Paratype male (OSUC 335807) **349** Antenna **350**. Paratype male (OSUC 237467)Metasoma, dorsal view. Morphbank^94^

Figures 345–350 Morphbank^94^

#### Description.

*Female*. Body length 2.65–3.05 mm (n=13).

Radicle color: same color as scape. Scape color: Yellowish; Brown. A4: broader than long; as long as broad. A5: broader than long. Antennal club: formed, segments compact.

Interantennal process: not elongate. Median longitudinal elevation in frontal depression: absent. Frontal depression: concave. Frontal depression sculpture: with 3 or more broadly interrupted transverse carinae. Submedian carina: strong, formed by a sharp raised carina. Submedian carina medially: without peak. Concavity across dorsal part of frontal depression: absent. Depression extending ventrally from median ocellus: absent. Upper frons: hood-like, protruding over pedicel when antenna at rest. Malar area near antennal foramen: with oblique tooth-like flange (facial nubbin). Malar area at mouth corner: with radiating striae. Smooth strip along posterior side of malar sulcus: absent or not consistently broad. Middle genal carina: present. Direction of middle genal carina dorsally: parallel to eye margin. Major sculpture of gena anteriorly: umbilicate-foveate; rugose. Major sculpture of gena posteriorly: umbilicate-foveate; rugose. Microsculpture of gena anteroventrally: absent. Microsculpture of gena posteroventrally: absent. Median carina extending posteriorly from hyperoccipital carina: absent. Hyperoccipital carina: complete, continuous with anterior genal carina. Lateral connection between hyperoccipital and occipital carinae: absent. Area between vertex and occipital carina: irregularly rugose. Occipital carina medially: sinuate, concave medial to corners, but without a median peak. Lateral corners of occipital carina: not protruding.

Lateral pronotal area: without bulge projecting towards anterior pit. Epomial corner: strong. Netrion surface anteriorly: not inflexed. Mesoscutum anteriorly: steep. Mesoscutal median carina: present and complete. Longitudinal carina between median carina and notauli: present. Major sculpture of medial mesoscutum anteriorly: umbilicate-foveate; irregularly rugose. Major sculpture of medial mesoscutum posteriorly: umbilicate-foveate; irregularly rugose. Microsculpture of medial mesoscutum anteriorly: granulate. Microsculpture of medial mesoscutum posteriorly: absent. Major sculpture of mesoscutellum: umbilicate-foveate; longitudinally rugose. Microsculpture of mesoscutellum medially: absent. Microsculpture of mesoscutellum laterally: absent. Mesoscutellar apex: convex or straight. Setae along anterior limit of femoral depression: arising from rows of foveae. Number of carinae crossing speculum above femoral depression: 3; 4. Number of carinae crossing femoral depression: more than 5. Mesepimeral sulcus pits: more than 5. Metascutellum dorsally: concave. Metascutellar sculpture dorsally: smooth or with transverse carinae. Median carina of metascutellum: absent or branched. Metascutellar setae: absent. Metascutellar apex: weakly emarginate. Metapleuron above ventral metapleural area: crossed by carinae. Metasomal depression setae: absent. Lateral propodeal carinae anteromedially: weakly diverging. Anterior areoles of metasomal depression: one or more areoles present. Anterior longitudinal carinae in metasomal depression: absent. Lateral propodeal areas: separated medially. Postmarginal vein: present. Fore wing apex: reaching apex of T5.

T1 midlobe: with 4 longitudinal carinae. T1: without anterior bulge. T2: with straight longitudinal striae or rugae. T6: broader than long. Apical flange of T6: not exposed apically. Metasomal apex: tapering to a sharp point. Major sculpture of T6: umbilicate-punctate. Microsculpture of T6: absent.

*Male*. Body length 2.65–3.5 mm (n=20). A5 tyloid: carina-like, not expanded. A11: broader than long. Median tooth of frontal depression: absent. Median lobe of T1: with 4 longitudinal carinae. Metasomal apex: with acuminate lateral corners.

#### Diagnosis.

Both sexes: Oblique tooth-like elevation present between antennal foramen and eye. Hyperoccipital carina present and sharp, continuous with anterior genal carina. Mesoscutellum without granulate sculpture. Metascutellum without dorsal setae. Propodeum without median carina; lateral propodeal carinae narrowly separated anteriorly. Female: T1 midlobe with 4 longitudinal carinae. T6 acuminate apically. Male: A11 broader than long. T1 midlobe with 4 longitudinal carinae. T7 with tiny, sharp posterolateral projections.

#### Etymology.

English noun, referring to the enlarged oblique flange between the antennal foramen and the eye.

#### Link to distribution map.

[http://hol.osu.edu/map-full.html?id=275480]

#### Material examined.

Holotype, female: **THAILAND**: Uthai Thani Prov., Huai Kha Khaeng Wildlife Sanctuary, 400m, III-1986, M. Allen, OSUC 368680 (deposited in CNCI). *Paratypes*: (13 females, 136 males) **CHINA**: 1 female, OSUC 442261 (BMNH). **THAILAND**: 12 females, 122 males, OSUC 285225, 285230, 361190 (BMNH); OSUC 368606, 368616-368617, 368621, 368629, 368632, 368681-368683, 368685-368686, 368696, 368766-368767, 368773, 464042 (CNCI); OSUC 237458, 247613, 247621, 247635, 247645, 247866, 247877, 247883-247884, 247907, 265260, 280497, 317854, 317862, 317868, 317871-317872, 317891, 320397, 320400-320401, 322098, 322120, 322122, 335546, 336632, 336741, 336781, 352456, 352471, 352903, 352907-352909, 361209-361210, 361278-361284, 361352, 361932, 361942-361946, 361963 (OSUC); OSUC 210386, 237467, 247615, 247646, 247654, 247885, 247891, 247893, 247911, 247915, 252043, 257400, 280508, 280512, 285213-285214, 285219, 285224, 285226, 285231, 309596, 335091, 335799, 335802-335804, 335807-335810, 335814, 335816, 335834, 336128-336130, 352470, 352904, 368492, 368494-368495, 368497-368498, 368511-368513, 368517, 368525, 368527-368532, 368535, 368540, 368542-368543 (QSBG); OSUC 335805, 335836 (ROME); UCRC ENT 150828-150829 (UCRC); OSUC 237463-237464 (WINC). **VIETNAM**: 14 males, OSUC 463999-464000 (CNCI); OSUC 278510, 278516-278517, 278519, 278533, 278535, 278538, 278541, 278543-278545, 281492 (RMNH).

#### Comments.

A3 and the pedicel in females are variable in length relative to other antennal segments, but A3 is slightly shorter than the pedicel in all examined females. The oblique elevation between the antennal foramen and eye occurs in a few other, almost certainly not closely related, *Oxyscelio* species, and may have a functional role. *Oxyscelio nubbin* is smaller-bodied than most other *Oxyscelio*.

### Oxyscelio obsidiani

Burks sp. n.

urn:lsid:zoobank.org:act:150CCE52-FDCC-48D7-AA9A-231226A10608

urn:lsid:biosci.ohio-state.edu:osuc_concepts:275501

http://species-id.net/wiki/Oxyscelio_obsidiani

**Figures 351–354.**
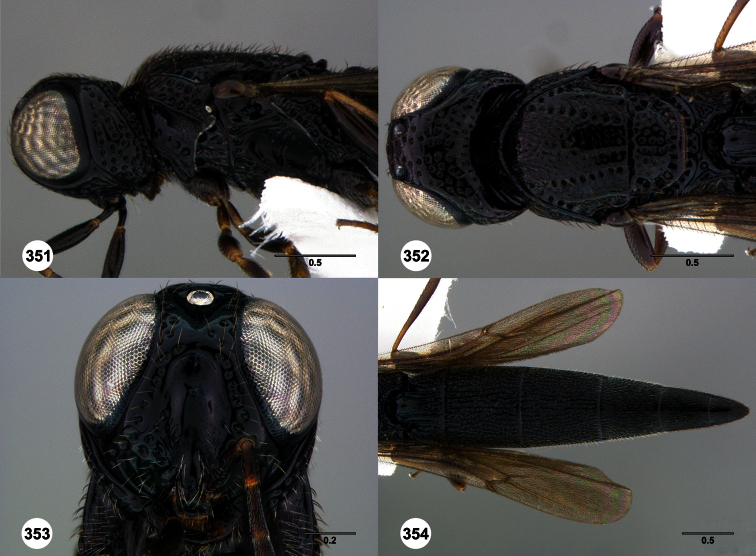
*Oxyscelio obsidiani* sp. n., holotype female (OSUC 368930) **351** Head and mesosoma, lateral view **352** Head and mesosoma, dorsal view **353** Head, anterior view **354** Metasoma, dorsal view. Morphbank^95^

Figures 351–354 Morphbank^95^

#### Description.

*Female*. Body length 5.6–6 mm (n=3).

Radicle color: darker than scape. Scape color: Brown. A4: longer than broad. A5: longer than broad. Antennal club: formed, segments compact.

Interantennal process: not elongate. Median longitudinal elevation in frontal depression: absent. Frontal depression: concave. Frontal depression sculpture: without transverse or oblique carinae below submedian carina. Submedian carina: weak, shallow and rounded or formed by ledge. Submedian carina medially: without peak. Concavity across dorsal part of frontal depression: absent. Depression extending ventrally from median ocellus: present. Upper frons: not hood-like. Malar area near antennal foramen: without carina or expansion. Malar area at mouth corner: with radiating striae. Smooth strip along posterior side of malar sulcus: absent or not consistently broad. Middle genal carina: absent. Direction of middle genal carina dorsally: absent (replace with question mark). Major sculpture of gena anteriorly: umbilicate-foveate. Major sculpture of gena posteriorly: umbilicate-foveate. Microsculpture of gena anteroventrally: absent. Microsculpture of gena posteroventrally: absent. Median carina extending posteriorly from hyperoccipital carina: absent. Hyperoccipital carina: not indicated medially. Lateral connection between hyperoccipital and occipital carinae: absent. Area between vertex and occipital carina: umbilicate-foveate. Occipital carina medially: absent. Lateral corners of occipital carina: sharp and protruding.

Lateral pronotal area: without bulge projecting towards anterior pit. Epomial corner: strong. Netrion surface anteriorly: not inflexed. Mesoscutum anteriorly: not steep. Mesoscutal median carina: absent or weak and incomplete in places. Longitudinal carina between median carina and notauli: absent. Major sculpture of medial mesoscutum anteriorly: umbilicate-punctate. Major sculpture of medial mesoscutum posteriorly: umbilicate-foveate; umbilicate-punctate. Microsculpture of medial mesoscutum anteriorly: absent. Microsculpture of medial mesoscutum posteriorly: absent. Major sculpture of mesoscutellum: umbilicate-foveate; umbilicate-punctate. Microsculpture of mesoscutellum medially: absent. Microsculpture of mesoscutellum laterally: absent. Mesoscutellar apex: convex or straight. Setae along anterior limit of femoral depression: arising from rows of foveae. Number of carinae crossing speculum above femoral depression: 3. Number of carinae crossing femoral depression: 3-5. Mesepimeral sulcus pits: more than 5. Metascutellum dorsally: concave. Metascutellar sculpture dorsally: smooth or with transverse carinae. Median carina of metascutellum: absent or branched. Metascutellar setae: absent. Metascutellar apex: convex or straight. Metapleuron above ventral metapleural area: foveate or rugose. Metasomal depression setae: absent. Lateral propodeal carinae anteromedially: strongly diverging. Anterior areoles of metasomal depression: absent. Anterior longitudinal carinae in metasomal depression: absent. Lateral propodeal areas: separated medially. Postmarginal vein: present. Fore wing apex: reaching middle of T4.

T1 midlobe: obscured by other raised sculpture. T1: with small rounded anterior bulge, not reaching metascutellum. T2: with straight longitudinal striae or rugae. T6: longer than broad. Apical flange of T6: exposed apically. Metasomal apex: rounded. Major sculpture of T6: umbilicate-punctate; longitudinally striate or rugose. Microsculpture of T6: granulate.

*Male*. Unknown.

#### Diagnosis.

Female: Head and mesosoma nearly smooth, submedian carina hardly indicated. A4 longer than broad. Frons without flange between antennal foramen and eye. Mesoscutellum without median carina. Metascutellum tiny, smooth centrally. Fore wing long enough to reach middle of T4. T1 midlobe with slight bulge obscuring longitudinal carinae. T6 longer than broad.

#### Etymology.

Latin, genitive case, referring to the similarity of the mesosoma to obsidian in color, smoothness, and gloss.

#### Link to distribution map.

[http://hol.osu.edu/map-full.html?id=275501]

#### Associations.

unspecified association Uncaria Schreber: [Rubiales: Rubiaceae]

#### Material Examined.

Holotype, female: **INDONESIA**: Maluku Prov., Ceram (Seram) Isl., Solea, VIII-1987, malaise trap, M. C. Day, OSUC 368930 (deposited in BMNH). *Paratypes*: **INDONESIA**: 2 females, OSUC 368923, 368939 (CNCI).

#### Comments.

*Oxyscelio obsidiani* is a distinctive species from Seram, exhibiting very weak sculpture as in some other species from that island. The elongate body, downward-directed face, and tiny metascutellum are shared by *Oxyscelio cupularis* and *Oxyscelio flavipennis*. Some or all of these species may form a monophyletic complex, but currently it would be defined by features that can only be vaguely communicated. An alternative placement would be in the *ogive*-group, but this would render that group difficult to clearly define.

### Oxyscelio ogive

Burks sp. n.

urn:lsid:zoobank.org:act:61291DA0-66FC-45B8-882D-7BA0CF9F6620

urn:lsid:biosci.ohio-state.edu:osuc_concepts:275519

http://species-id.net/wiki/Oxyscelio_ogive

**Figures 355–358.**
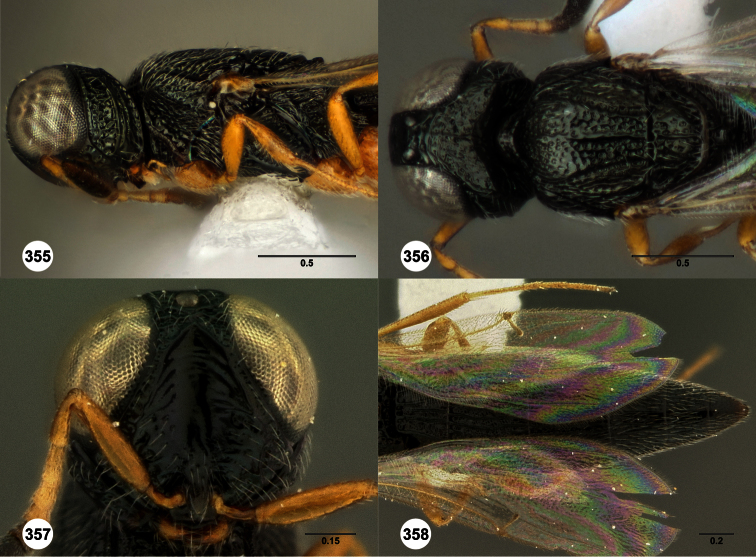
*Oxyscelio ogive* sp. n., paratype female (OSUC 361927) **355** Head and mesosoma, lateral view **356** Head and mesosoma, dorsal view. Holotype female (OSUC 368774) **357** Head, anterior view **358** Metasoma, dorsal view. Morphbank^96^

Figures 355–358 Morphbank^96^

#### Description.

*Female*. Body length 3.2–3.7 mm (n=12).

Radicle color: same color as scape. Scape color: Yellowish. A4: longer than broad. A5: broader than long. Antennal club: formed, segments compact.

Interantennal process: not elongate. Median longitudinal elevation in frontal depression: absent. Frontal depression: concave. Frontal depression sculpture: with 3-5 complete transverse carinae. Submedian carina: strong, formed by a sharp raised carina. Submedian carina medially: with sharp peak. Concavity across dorsal part of frontal depression: absent. Depression extending ventrally from median ocellus: absent. Upper frons: not hood-like. Malar area near antennal foramen: without carina or expansion. Malar area at mouth corner: with radiating striae. Smooth strip along posterior side of malar sulcus: absent or not consistently broad. Middle genal carina: present. Direction of middle genal carina dorsally: parallel to eye margin. Major sculpture of gena anteriorly: umbilicate-foveate; rugose. Major sculpture of gena posteriorly: rugose. Microsculpture of gena anteroventrally: absent. Microsculpture of gena posteroventrally: granulate. Median carina extending posteriorly from hyperoccipital carina: present. Hyperoccipital carina: indicated by rugae. Lateral connection between hyperoccipital and occipital carinae: present as a weak elevation. Area between vertex and occipital carina: with transverse carinae. Occipital carina medially: divided into concave halves, meeting at median peak. Lateral corners of occipital carina: sharp and protruding.

Lateral pronotal area: without bulge projecting towards anterior pit. Epomial corner: strong. Netrion surface anteriorly: not inflexed. Mesoscutum anteriorly: not steep. Mesoscutal median carina: present and complete. Longitudinal carina between median carina and notauli: absent. Major sculpture of medial mesoscutum anteriorly: umbilicate-foveate. Major sculpture of medial mesoscutum posteriorly: umbilicate-foveate. Microsculpture of medial mesoscutum anteriorly: granulate. Microsculpture of medial mesoscutum posteriorly: absent. Major sculpture of mesoscutellum: umbilicate-foveate. Microsculpture of mesoscutellum medially: absent. Microsculpture of mesoscutellum laterally: granulate. Mesoscutellar apex: convex or straight. Setae along anterior limit of femoral depression: arising from rows of foveae. Number of carinae crossing speculum above femoral depression: 3. Number of carinae crossing femoral depression: more than 5. Mesepimeral sulcus pits: more than 5. Metascutellum dorsally: concave. Metascutellar sculpture dorsally: smooth or with transverse carinae. Median carina of metascutellum: absent or branched. Metascutellar setae: absent. Metascutellar apex: weakly emarginate. Metapleuron above ventral metapleural area: crossed by carinae. Metasomal depression setae: absent. Lateral propodeal carinae anteromedially: strongly diverging. Anterior areoles of metasomal depression: one or more areoles present. Anterior longitudinal carinae in metasomal depression: median carina present. Lateral propodeal areas: separated medially. Postmarginal vein: absent. Fore wing apex: reaching apex of T4; reaching middle of T5; reaching apex of T5.

T1 midlobe: with 4 longitudinal carinae; with 5 longitudinal carinae. T1: without anterior bulge. T2: with straight longitudinal striae or rugae. T6: broader than long. Apical flange of T6: exposed apically. Metasomal apex: rounded. Major sculpture of T6: umbilicate-punctate; longitudinally striate or rugose. Microsculpture of T6: absent.

*Male*. Unknown.

#### Diagnosis.

Female: A4, A5 broader than long. Submedian carina with a sharp median peak. Occipital carina complete as a distinct carina, but medial portions concave and meeting at a peak. Mesoscutellum granulate laterally. Metascutellum tiny, dorsally concave. Fore wings not long enough to reach beyond T5. T1 midlobe with 4 or 5 longitudinal carinae. T6 rounded apically.

#### Etymology.

French noun describing the tapered end of an object or arch. Refers to the similarity between the pointed arch formed by the submedian carina and an ogival arch in Gothic architecture.

#### Link to distribution map.

[http://hol.osu.edu/map-full.html?id=275519]

#### Material examined.

Holotype, female: **THAILAND**: Uthai Thani Prov., Khao Nang Rum Wildlife Research Station, 400m, V-1986, malaise trap, M. G. Allen, OSUC 368774 (deposited in CNCI). *Paratypes*: (12 females) **INDONESIA**: 1 female, OSUC 369262 (CNCI). **THAILAND**: 10 females, OSUC 335560 (BMNH); OSUC 368684, 368726 (CNCI); OSUC 361926-361927, 368499 (OSUC); OSUC 252044, 335561, 368534 (QSBG); UCRC ENT 135265 (UCRC). **VIETNAM**: 1 female, OSUC 369126 (CNCI).

#### Comments.

*Oxyscelio ogive* is similar to several different small-bodied species of *Oxyscelio* without a sharp hyperoccipital carina. The ogival arch formed by the submedian carina, and the shape and distinctness of the occipital carina, can help distinguish it. It exhibits an unusual range of surface sculpture variation, including flattened and granulate T1 midlobe carinae in some specimens. This variation was not considered sufficient to justify splitting these into separate species.

### Oxyscelio operimenti

Burks sp. n.

urn:lsid:zoobank.org:act:CBEBD404-BA2A-47BD-BAC9-B86B294B4F50

urn:lsid:biosci.ohio-state.edu:osuc_concepts:305704

http://species-id.net/wiki/Oxyscelio_operimenti

**Figures 359–362.**
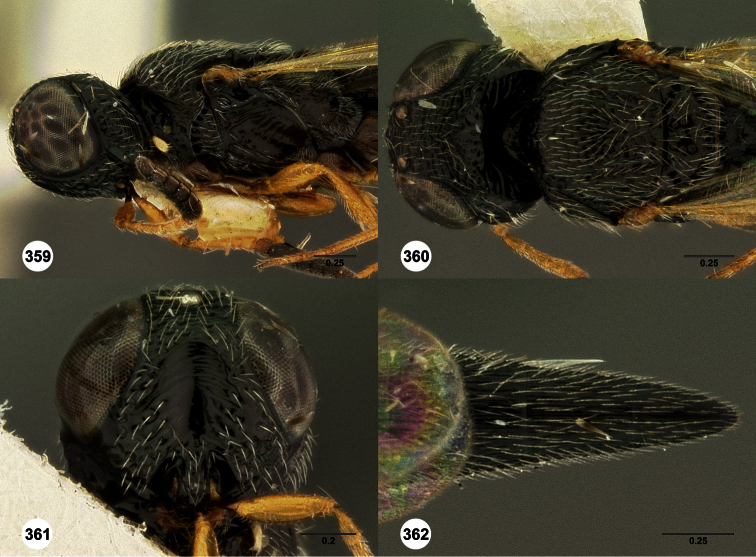
*Oxyscelio operimenti* sp. n., holotype female (OSUC 376738) **359** Head and mesosoma, lateral view **360** Head and mesosoma, dorsal view **361** Head, anterior view **362** Metasoma, dorsal view. Morphbank^97^

Figures 359–362 Morphbank^97^

#### Description.

*Female*. Body length 4.85 mm (n=1).

Radicle color: darker than scape. Scape color: Yellowish. A4: longer than broad. A5: broader than long. Antennal club: formed, segments compact.

Interantennal process: not elongate. Median longitudinal elevation in frontal depression: absent. Frontal depression: concave. Frontal depression sculpture: with 3-5 complete transverse carinae. Submedian carina: weak, shallow and rounded or formed by ledge. Submedian carina medially: without peak. Concavity across dorsal part of frontal depression: absent. Depression extending ventrally from median ocellus: absent. Upper frons: not hood-like. Malar area near antennal foramen: with oblique tooth-like flange (facial nubbin). Malar area at mouth corner: without striae. Smooth strip along posterior side of malar sulcus: present, broad throughout its length. Middle genal carina: present. Direction of middle genal carina dorsally: parallel to eye margin. Major sculpture of gena anteriorly: umbilicate-foveate. Major sculpture of gena posteriorly: umbilicate-foveate; rugose. Microsculpture of gena anteroventrally: absent. Microsculpture of gena posteroventrally: absent. Median carina extending posteriorly from hyperoccipital carina: absent. Hyperoccipital carina: indicated by rugae. Lateral connection between hyperoccipital and occipital carinae: present as a weak elevation. Area between vertex and occipital carina: umbilicate-foveate; irregularly rugose. Occipital carina medially: sinuate, concave medial to corners, but without a median peak. Lateral corners of occipital carina: sharp and protruding.

Lateral pronotal area: without bulge projecting towards anterior pit. Epomial corner: strong. Netrion surface anteriorly: not inflexed. Mesoscutum anteriorly: not steep. Mesoscutal median carina: present and complete. Longitudinal carina between median carina and notauli: absent. Major sculpture of medial mesoscutum anteriorly: umbilicate-foveate. Major sculpture of medial mesoscutum posteriorly: umbilicate-foveate. Microsculpture of medial mesoscutum anteriorly: granulate. Microsculpture of medial mesoscutum posteriorly: absent. Major sculpture of mesoscutellum: umbilicate-foveate. Microsculpture of mesoscutellum medially: absent. Microsculpture of mesoscutellum laterally: absent; granulate. Mesoscutellar apex: roundly concave. Setae along anterior limit of femoral depression: arising from rows of foveae. Number of carinae crossing speculum above femoral depression: 2. Number of carinae crossing femoral depression: 3-5. Mesepimeral sulcus pits: 3-5. Metascutellum dorsally: convex. Metascutellar sculpture dorsally: with scattered rugae; granulate. Median carina of metascutellum: absent or branched. Metascutellar setae: absent. Metascutellar apex: deeply emarginate. Metapleuron above ventral metapleural area: crossed by carinae. Metasomal depression setae: absent. Lateral propodeal carinae anteromedially: strongly diverging. Anterior areoles of metasomal depression: absent. Anterior longitudinal carinae in metasomal depression: absent. Lateral propodeal areas: separated medially. Postmarginal vein: absent. Fore wing apex: reaching middle of T4.

T1 midlobe: obscured by other raised sculpture. T1: with long anterior bulge, reaching metascutellum. T2: with straight longitudinal striae or rugae. T6: longer than broad. Apical flange of T6: exposed apically. Metasomal apex: rounded. Major sculpture of T6: umbilicate-punctate; longitudinally striate or rugose. Microsculpture of T6: granulate.

*Male*. Unknown.

#### Diagnosis.

Female: Antennal club formed. A4 longer than broad, A5 slightly broader than long. Face with oblique expanded flange between antennal foramen and eye. Gena with 5 carinae posteriorly. Metascutellum convex, deeply triangularly emarginate posteriorly. Fore wing long enough to reach middle of T4. T1 horn elongate, reaching metascutellum.

#### Etymology.

Latin noun, genitive case, meaning “a covering.” Refers to how the metascutellum covers the T1 horn.

#### Link to distribution map.

[http://hol.osu.edu/map-full.html?id=305704]

#### Material examined.

Holotype, female: **PHILIPPINES**: Davao del Sur Prov., Mindanao Isl., Calian, 14-VII, C. S. Clagg, OSUC 376738 (deposited in MCZC).

### Oxyscelio peludo

Burks sp. n.

urn:lsid:zoobank.org:act:735DD7C7-B2A4-466C-87A8-5CA6798A342B

urn:lsid:biosci.ohio-state.edu:osuc_concepts:275528

http://species-id.net/wiki/Oxyscelio_peludo

**Figures 363–368.**
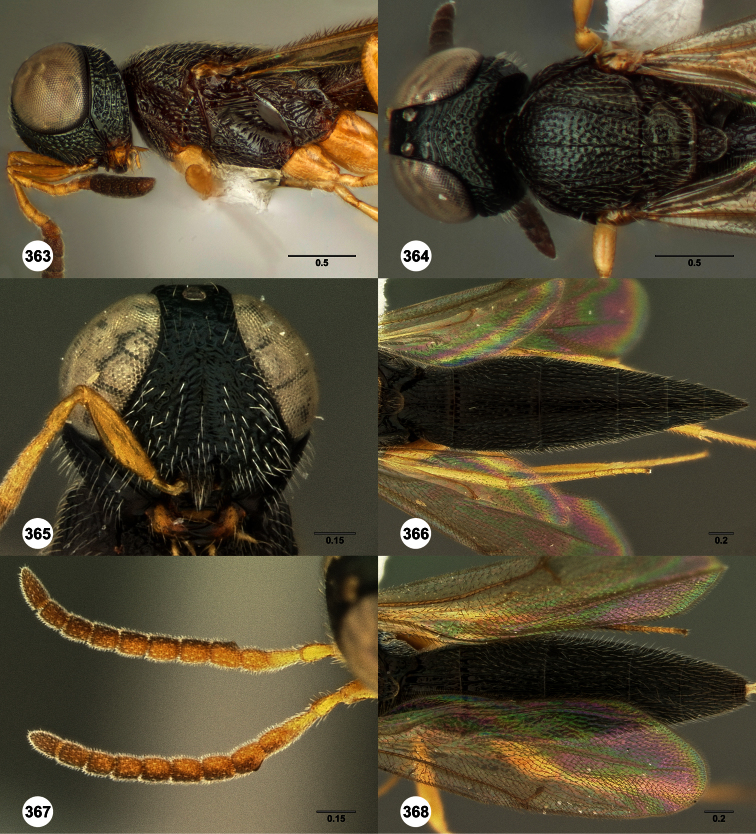
*Oxyscelio peludo* sp. n., holotype female (OSUC 257095) **363** Head and mesosoma, lateral view **364** Head and mesosoma, dorsal view. Paratype female (OSUC 376605) **365** Head, anterior view. Paratype female (OSUC 257081) **366** Metasoma, dorsal view. Paratype male (OSUC 361275) **367 **Antenna. Paratype male (OSUC 247856) **368** Metasoma, dorsal view. Morphbank^98^

Figures 363–368 Morphbank^98^

#### Description.

*Female*. Body length 4.5–5.05 mm (n=10).

Radicle color: same color as scape. Scape color: Yellowish. A4: longer than broad. A5: broader than long; as long as broad. Antennal club: formed, segments compact.

Interantennal process: not elongate. Median longitudinal elevation in frontal depression: absent. Frontal depression: concave. Frontal depression sculpture: with 3-5 complete transverse carinae. Submedian carina: weak, shallow and rounded or formed by ledge. Submedian carina medially: without peak. Concavity across dorsal part of frontal depression: absent. Depression extending ventrally from median ocellus: absent. Upper frons: not hood-like. Malar area near antennal foramen: without carina or expansion. Malar area at mouth corner: with radiating striae. Smooth strip along posterior side of malar sulcus: present, broad throughout its length. Middle genal carina: present. Direction of middle genal carina dorsally: parallel to eye margin. Major sculpture of gena anteriorly: umbilicate-foveate. Major sculpture of gena posteriorly: umbilicate-foveate; rugose. Microsculpture of gena anteroventrally: absent. Microsculpture of gena posteroventrally: granulate. Median carina extending posteriorly from hyperoccipital carina: absent. Hyperoccipital carina: indicated by rugae. Lateral connection between hyperoccipital and occipital carinae: present as a weak elevation. Area between vertex and occipital carina: umbilicate-foveate. Occipital carina medially: absent. Lateral corners of occipital carina: sharp and protruding.

Lateral pronotal area: without bulge projecting towards anterior pit. Epomial corner: weak. Netrion surface anteriorly: not inflexed. Mesoscutum anteriorly: not steep. Mesoscutal median carina: present and complete. Longitudinal carina between median carina and notauli: absent. Major sculpture of medial mesoscutum anteriorly: umbilicate-foveate. Major sculpture of medial mesoscutum posteriorly: umbilicate-foveate. Microsculpture of medial mesoscutum anteriorly: granulate. Microsculpture of medial mesoscutum posteriorly: absent. Major sculpture of mesoscutellum: umbilicate-foveate. Microsculpture of mesoscutellum medially: granulate. Microsculpture of mesoscutellum laterally: absent; granulate. Mesoscutellar apex: convex or straight. Setae along anterior limit of femoral depression: arising from rows of foveae. Number of carinae crossing speculum above femoral depression: 3. Number of carinae crossing femoral depression: more than 5. Mesepimeral sulcus pits: more than 5. Metascutellum dorsally: flat. Metascutellar sculpture dorsally: with scattered rugae. Median carina of metascutellum: absent or branched. Metascutellar setae: with many dorsal setae. Metascutellar apex: convex or straight. Metapleuron above ventral metapleural area: crossed by carinae. Metasomal depression setae: absent. Lateral propodeal carinae anteromedially: strongly diverging. Anterior areoles of metasomal depression: absent. Anterior longitudinal carinae in metasomal depression: absent. Lateral propodeal areas: separated medially. Postmarginal vein: present. Fore wing apex: reaching middle of T5.

T1 midlobe: obscured by other raised sculpture. T1: with small rounded anterior bulge, not reaching metascutellum. T2: with straight longitudinal striae or rugae. T6: longer than broad. Apical flange of T6: not exposed apically. Metasomal apex: tapering to a sharp point. Major sculpture of T6: umbilicate-punctate; longitudinally striate or rugose. Microsculpture of T6: granulate.

*Male*. Body length 3.65–4.5 mm (n=4). A5 tyloid: carina-like, not expanded. A11: longer than broad. Median tooth of frontal depression: absent. Median lobe of T1: with 5 longitudinal carinae. Metasomal apex: with acuminate lateral corners.

**Diagnosis**. Both sexes: A4 longer than broad, A5 about as long as broad. Frontal depression crossed by many carinae. Mesoscutellum strongly granulate. Metascutellum dorsally setose. Female: Metascutellum fingernail-shaped, rugose. T1 with strong anterior horn. Fore wings long enough to reach middle of T5. T6 strongly narrowing towards nearly acuminate apex. Male: T1 midlobe with 5 longitudinal carinae. T7 with sharp, protruding posterolateral corners. The metascutellar setae of *Oxyscelio peludo* are easily overlooked in dorsal view, but are more apparent from an oblique or lateral view. They are present in males as well, making males of *Oxyscelio peludo* much more easily recognizable than those of most other species. Among species with a bare metascutellum, *Oxyscelio naraws* very strongly resembles *Oxyscelio peludo*.

#### Etymology.

Portuguese and Spanish, meaning “hairy.” Refers to the setose metascutellum.

#### Link to distribution map.

[http://hol.osu.edu/map-full.html?id=275528]

#### Material Examined.

Holotype, female: **INDONESIA**: Kalimantan Barat Prov., Cabang Panti Research Station, RR6, 1° rainforest / sandstone closed canopy, IIS 910136, Gunung Palung National Park, 01°15'S, 110°05'E, 100m, 17.VI–29.VI.1991, canopy malaise trap, Darling, Rosichon & Sutrisno, OSUC 257095 (deposited in MBBJ). *Paratypes*: (12 females, 4 males) **INDONESIA**: 6 females, 3 males, OSUC 361275, ROMEnt Spec. No. 112263, ROMEnt Spec. No. 112681 (MBBJ); OSUC 228710, 257081, 273319 (OSUC); OSUC 247856, 247970, 257098 (ROME). **MALAYSIA**: 6 females, 1 male, OSUC 376590, 376602, 376605, 376611 (BMNH); OSUC 453764, 453768, 453772 (OSUC).

### Oxyscelio perpensus

Kononova

urn:lsid:zoobank.org:act:8323BFBD-BFC7-4DA7-9C8A-767CECA029EC

urn:lsid:biosci.ohio-state.edu:osuc_concepts:243849

http://species-id.net/wiki/Oxyscelio_perpensus

**Figures 369–372.**
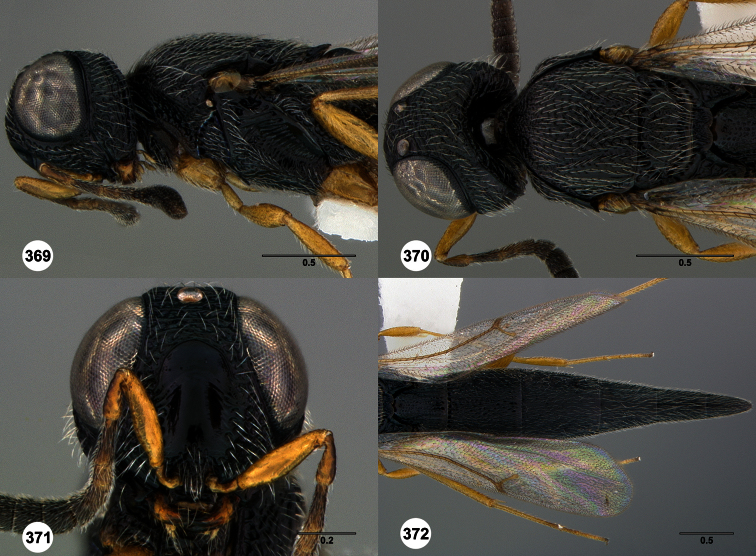
*Oxyscelio perpensus* Kononova, female (OSUC 368979) **369** Head and mesosoma, lateral view **370** Head and mesosoma, dorsal view **371** Head, anterior view **372** Metasoma, dorsal view. Morphbank^99^

Figures 369–372 Morphbank^99^

Oxyscelio perpensum Kononova: Kononova and Fursov 2007: 104 (description); Kononova and Fursov 2007: 63 (original description).
Oxyscelio perpensus Kononova: Kononova and Kozlov 2008: 190, 192 (description, keyed).

#### Description.

*Female*. Body length 5.15–5.4 mm (n=7).

Radicle color: same color as scape. Scape color: Yellowish. A4: longer than broad. A5: longer than broad. Antennal club: formed, segments compact.

Interantennal process: not elongate. Median longitudinal elevation in frontal depression: absent. Frontal depression: concave. Frontal depression sculpture: with 3 or more broadly interrupted transverse carinae. Submedian carina: weak, shallow and rounded or formed by ledge. Submedian carina medially: without peak. Concavity across dorsal part of frontal depression: absent. Depression extending ventrally from median ocellus: absent. Upper frons: not hood-like. Malar area near antennal foramen: without carina or expansion. Malar area at mouth corner: with radiating striae. Smooth strip along posterior side of malar sulcus: absent or not consistently broad. Middle genal carina: present. Direction of middle genal carina dorsally: parallel to eye margin. Major sculpture of gena anteriorly: umbilicate-foveate. Major sculpture of gena posteriorly: umbilicate-foveate; rugose. Microsculpture of gena anteroventrally: absent. Microsculpture of gena posteroventrally: granulate. Median carina extending posteriorly from hyperoccipital carina: absent. Hyperoccipital carina: not indicated medially. Lateral connection between hyperoccipital and occipital carinae: present as a weak elevation. Area between vertex and occipital carina: umbilicate-foveate. Occipital carina medially: sinuate, concave medial to corners, but without a median peak. Lateral corners of occipital carina: sharp and protruding.

Lateral pronotal area: without bulge projecting towards anterior pit. Epomial corner: strong. Netrion surface anteriorly: not inflexed. Mesoscutum anteriorly: not steep. Mesoscutal median carina: absent or weak and incomplete in places. Longitudinal carina between median carina and notauli: absent. Major sculpture of medial mesoscutum anteriorly: umbilicate-foveate. Major sculpture of medial mesoscutum posteriorly: umbilicate-foveate. Microsculpture of medial mesoscutum anteriorly: granulate. Microsculpture of medial mesoscutum posteriorly: absent. Major sculpture of mesoscutellum: umbilicate-foveate; irregularly rugose. Microsculpture of mesoscutellum medially: absent. Microsculpture of mesoscutellum laterally: granulate. Mesoscutellar apex: convex or straight. Setae along anterior limit of femoral depression: arising from tiny pits. Number of carinae crossing speculum above femoral depression: 3. Number of carinae crossing femoral depression: more than 5. Mesepimeral sulcus pits: more than 5. Metascutellum dorsally: flat. Metascutellar sculpture dorsally: with scattered rugae. Median carina of metascutellum: absent or branched. Metascutellar setae: absent. Metascutellar apex: weakly emarginate. Metapleuron above ventral metapleural area: crossed by carinae. Metasomal depression setae: absent. Lateral propodeal carinae anteromedially: strongly diverging. Anterior areoles of metasomal depression: absent. Anterior longitudinal carinae in metasomal depression: absent. Lateral propodeal areas: separated medially. Postmarginal vein: absent. Fore wing apex: reaching middle of T4.

T1 midlobe: obscured by other raised sculpture. T1: with small rounded anterior bulge, not reaching metascutellum. T2: with straight longitudinal striae or rugae. T6: longer than broad. Apical flange of T6: not exposed apically. Metasomal apex: rounded. Major sculpture of T6: umbilicate-punctate; longitudinally striate or rugose. Microsculpture of T6: granulate.

*Male*. Unknown.

#### Diagnosis.

Female: A4 longer than broad. Upper frons without additional carinae dorsal to submedian carina. Hyperoccipital carina indicated by rugae. Mesoscutellum with granulate sculpture posterolaterally. Mesofemoral depression crossed by many carinae below speculum. Mesopleuron along anteroventral edge of femoral depression without rows of foveae, setae arising from tiny pits. Metascutellum broad and rounded, with many scattered rugae. T1 midlobe with strong, broad anterior bulge. T2 without sublateral depressions or curved striae. T6 longer than broad, tapering to a rounded apex. *Oxyscelio perpensus* differs from other Palearctic *Oxyscelio* in having a broader metascutellum.

#### Link to distribution map.

[http://hol.osu.edu/map-full.html?id=243849]

#### Associations.

emerged from egg of Orthoptera: [Orthoptera]

#### Material examined.

Holotype, female, *Oxyscelio perpensum*: **JAPAN**: Aichi Pref., Kitashitara Co., Honshu Isl., Shitara Town, hill, Dando-Uradani Virgin Forest, 900m, 15.VIII.2004, V. Fursov, UASK 0109 (deposited in UASK).*Paratypes*: **JAPAN**: 2 females, 1 male, OSUC 173067-173069 (UASK). *Other material*: **JAPAN**: 7 females, OSUC 368976-368979, 369004-369006 (CNCI).

#### Comments.

The mesoscutal median carina is less visible in our figure (Fig. 374) than in that of Kononova and Fursov (2007: fig. 9.1), but this is because the carina is relatively weak and rounded, becoming less visible under diffused lighting.

### Oxyscelio planocarinae

Burks sp. n.

urn:lsid:zoobank.org:act:D919CE92-E6BA-4359-819D-8827A2C504E7

urn:lsid:biosci.ohio-state.edu:osuc_concepts:275542

http://species-id.net/wiki/Oxyscelio_planocarinae

**Figures 373–376.**
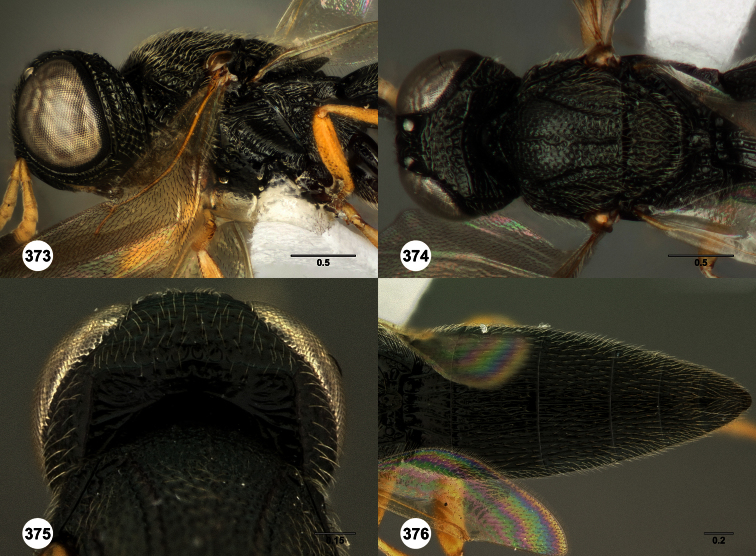
*Oxyscelio planocarinae* sp. n., holotype female (OSUC 257082) **373** Head and mesosoma, lateral view **374** Head and mesosoma, dorsal view **375** Head, posterodorsal view **376 **Metasoma, dorsal view. Morphbank^100^

Figures 373–376 Morphbank^100^

#### Description.

*Female*. Body length 3.6–4.7 mm (n=3).

Radicle color: darker than scape. Scape color: Yellowish. A4: broader than long. A5: broader than long. Antennal club: formed, segments compact.

Interantennal process: not elongate. Median longitudinal elevation in frontal depression: absent. Frontal depression: concave. Frontal depression sculpture: with 3 or more broadly interrupted transverse carinae. Submedian carina: strong, formed by a sharp raised carina. Submedian carina medially: without peak. Concavity across dorsal part of frontal depression: absent. Depression extending ventrally from median ocellus: present. Upper frons: hood-like, protruding over pedicel when antenna at rest. Malar area near antennal foramen: without carina or expansion. Malar area at mouth corner: without striae; with one carina. Smooth strip along posterior side of malar sulcus: present, broad throughout its length. Middle genal carina: present. Direction of middle genal carina dorsally: parallel to eye margin. Major sculpture of gena anteriorly: umbilicate-foveate. Major sculpture of gena posteriorly: umbilicate-foveate. Microsculpture of gena anteroventrally: absent. Microsculpture of gena posteroventrally: absent. Median carina extending posteriorly from hyperoccipital carina: absent. Hyperoccipital carina: indicated by rugae. Lateral connection between hyperoccipital and occipital carinae: present as a weak elevation. Area between vertex and occipital carina: umbilicate-foveate. Occipital carina medially: flat. Lateral corners of occipital carina: sharp and protruding.

Lateral pronotal area: without bulge projecting towards anterior pit. Epomial corner: strong. Netrion surface anteriorly: not inflexed. Mesoscutum anteriorly: not steep. Mesoscutal median carina: present and complete. Longitudinal carina between median carina and notauli: absent. Major sculpture of medial mesoscutum anteriorly: umbilicate-foveate. Major sculpture of medial mesoscutum posteriorly: umbilicate-foveate. Microsculpture of medial mesoscutum anteriorly: granulate. Microsculpture of medial mesoscutum posteriorly: absent. Major sculpture of mesoscutellum: umbilicate-foveate; longitudinally rugose. Microsculpture of mesoscutellum medially: absent. Microsculpture of mesoscutellum laterally: absent. Mesoscutellar apex: convex or straight. Setae along anterior limit of femoral depression: arising from rows of foveae. Number of carinae crossing speculum above femoral depression: 3. Number of carinae crossing femoral depression: more than 5. Mesepimeral sulcus pits: more than 5. Metascutellum dorsally: concave. Metascutellar sculpture dorsally: smooth or with transverse carinae. Median carina of metascutellum: absent or branched. Metascutellar setae: absent. Metascutellar apex: weakly emarginate. Metapleuron above ventral metapleural area: foveate or rugose. Metasomal depression setae: absent. Lateral propodeal carinae anteromedially: weakly diverging. Anterior areoles of metasomal depression: one or more areoles present. Anterior longitudinal carinae in metasomal depression: median carina present. Lateral propodeal areas: separated medially. Postmarginal vein: present. Fore wing apex: reaching apex of T5.

T1 midlobe: with 5 longitudinal carinae. T1: without anterior bulge. T2: with straight longitudinal striae or rugae. T6: broader than long. Apical flange of T6: exposed apically. Metasomal apex: rounded. Major sculpture of T6: umbilicate-punctate; longitudinally striate or rugose. Microsculpture of T6: granulate.

*Male*. Unknown.

#### Diagnosis.

Female: Occipital carina complete but flat medially. Metascutellum narrowing posteriorly, with a median channel. T1 midlobe with 5 longitudinal carinae.

#### Etymology.

Latin noun, genitive case, meaning “flat carina.” Refers to the medially flat occipital carina.

#### Link to distribution map.

[http://hol.osu.edu/map-full.html?id=275542]

#### Material examined.

Holotype, female: **INDONESIA**: Kalimantan Barat Prov., Cabang Panti Research Station, 1° rainforest / peat swamp, IIS 910131, Gunung Palung National Park, 01°15'S, 110°05'E, 100-400m, 15.VI–15.VIII.1991, malaise trap, Darling, Rosichon & Sutrisno, OSUC 257082 (deposited in MBBJ). *Paratypes*: (4 females) **INDONESIA**: 1 female, OSUC 368944 (CNCI). **MALAYSIA**: 3 females, OSUC 369043, 463997-463998 (CNCI).

#### Comments.

*Oxyscelio planocarinae* is distinctive in several ways, including the medially flat occipital carina, hood-like frontal depression, and narrowing metascutellum with a median channel. Its coloration is distinctive, with dark coxae but very pale A3-A5. Its body has a stout look, with a relatively broad metasoma.

### Oxyscelio praecipitis

Burks sp. n.

urn:lsid:zoobank.org:act:23A50CE3-17CE-41C1-AD8B-C7195A590C68

urn:lsid:biosci.ohio-state.edu:osuc_concepts:275565

http://species-id.net/wiki/Oxyscelio_praecipitis

**Figures 377–380.**
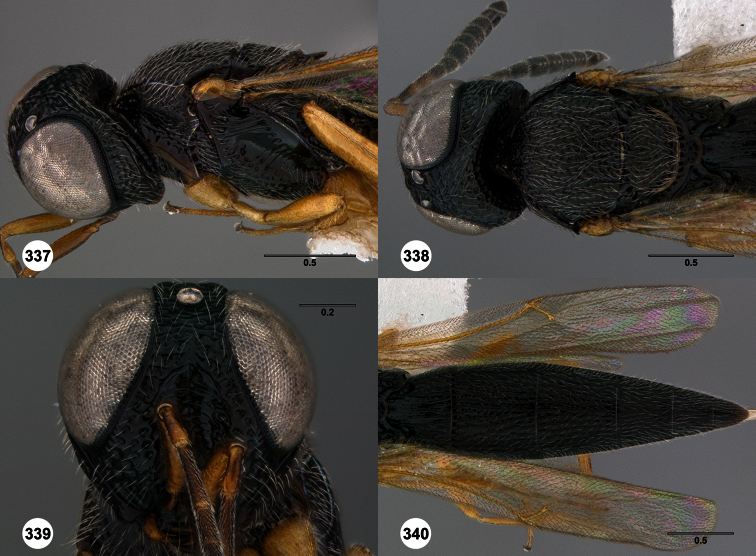
*Oxyscelio praecipitis* sp. n., holotype male (OSUC 268220) **377** Head and mesosoma, lateral view **378** Head and mesosoma, dorsal view **379** Head, anterior view **380** Propodeum, posterior view. Morphbank^101^

Figures 377–380 Morphbank^101^

#### Description.

*Female*. Unknown.

*Male*. Body length 3.8 mm (n=1).

Radicle color: same color as scape. Scape color: Brown. A5 tyloid: carina-like, not expanded. A11: longer than broad.

Interantennal process: not elongate. Median longitudinal elevation in frontal depression: absent. Frontal depression: concave. Frontal depression sculpture: without transverse or oblique carinae below submedian carina. Submedian carina: strong, formed by a sharp raised carina. Submedian carina medially: without peak. Median tooth of frontal depression: absent. Concavity across dorsal part of frontal depression: absent. Depression extending ventrally from median ocellus: absent. Upper frons: not hood-like. Malar area near antennal foramen: without carina or expansion. Malar area at mouth corner: with radiating striae. Smooth strip along posterior side of malar sulcus: absent or not consistently broad. Middle genal carina: absent. Direction of middle genal carina dorsally: absent (replace with question mark). Major sculpture of gena anteriorly: umbilicate-foveate; rugose. Major sculpture of gena posteriorly: umbilicate-foveate; rugose. Microsculpture of gena anteroventrally: absent; granulate. Microsculpture of gena posteroventrally: absent. Median carina extending posteriorly from hyperoccipital carina: absent. Hyperoccipital carina: indicated by rugae. Lateral connection between hyperoccipital and occipital carinae: absent. Area between vertex and occipital carina: umbilicate-foveate; irregularly rugose. Occipital carina medially: absent. Lateral corners of occipital carina: not protruding.

Lateral pronotal area: without bulge projecting towards anterior pit. Epomial corner: strong. Netrion surface anteriorly: not inflexed. Mesoscutum anteriorly: steep. Mesoscutal median carina: present and complete. Longitudinal carina between median carina and notauli: absent. Major sculpture of medial mesoscutum anteriorly: umbilicate-foveate. Major sculpture of medial mesoscutum posteriorly: umbilicate-foveate. Microsculpture of medial mesoscutum anteriorly: absent; granulate. Microsculpture of medial mesoscutum posteriorly: absent; granulate. Major sculpture of mesoscutellum: umbilicate-foveate. Microsculpture of mesoscutellum medially: absent. Microsculpture of mesoscutellum laterally: granulate. Mesoscutellar apex: convex or straight. Setae along anterior limit of femoral depression: arising from rows of foveae. Number of carinae crossing speculum above femoral depression: 3. Number of carinae crossing femoral depression: more than 5. Mesepimeral sulcus pits: more than 5. Metascutellum dorsally: concave. Metascutellar sculpture dorsally: smooth or with transverse carinae. Median carina of metascutellum: absent or branched. Metascutellar setae: with many dorsal setae. Metascutellar apex: weakly emarginate. Metapleuron above ventral metapleural area: crossed by carinae. Metasomal depression setae: absent. Anterior areoles of metasomal depression: one or more areoles present. Anterior longitudinal carinae in metasomal depression: median carina present. Lateral propodeal areas: separated medially. Postmarginal vein: present.

Median lobe of T1: with 4 longitudinal carinae. Metasomal apex: with acuminate lateral corners.

#### Diagnosis.

Male: Frontal depression shallow, submedian carina only indicated dorsally. Hyperoccipital carina complete but indicated by a set of rugae. Mesosoma tall and steep anteriorly. Medial mesoscutum and mesoscutellum with granulate sculpture. Metascutellum setose dorsally. Petiolar depression with median carina anteriorly. T1 midlobe with 4 longitudinal carinae. T7 with sharp, protruding posterolateral corners. *Oxyscelio praecipitis* is unusual among Philippine species in having an anteriorly tall and steep mesosoma. The dorsally setose metascutellum is also very unusual, especially in species with a weakly defined hyperoccipital carina.

#### Etymology.

Latin noun, genitive case, meaning “danger” or “steep place.”

#### Link to distribution map.

[http://hol.osu.edu/map-full.html?id=275565]

#### Material examined.

Holotype, male: **PHILIPPINES**: Laguna Prov., Mount Makiling (Maquiling), no date, Baker, OSUC 268220 (deposited in USNM).

### Oxyscelio reflectens

Burks sp. n.

urn:lsid:zoobank.org:act:B4593C80-677A-4F9C-B118-9C60399EE7C2

urn:lsid:biosci.ohio-state.edu:osuc_concepts:275459

http://species-id.net/wiki/Oxyscelio_reflectens

**Figures 381–386.**
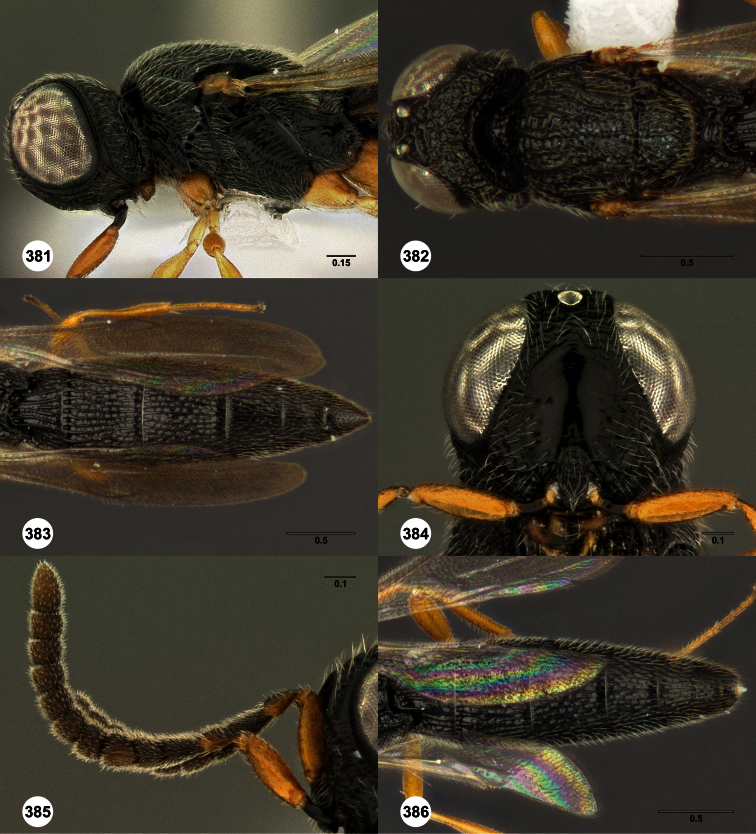
*Oxyscelio reflectens* sp. n., paratype female (OSUC 322063) **381** Head and mesosoma, lateral view. Paratype female (OSUC 280506) **382** Head and mesosoma, dorsal view **383** Metasoma, dorsal view. Paratype female (OSUC 317875) **384** Head, anterior view. Paratype male (OSUC 317855) **385** A5 tyloid. Paratype male (OSUC 247903) **386** Metasoma, dorsal view. Morphbank^102^

Figures 381–386 Morphbank^102^

#### Description.

*Female*. Body length 3.05–5.65 mm (n=20).

Radicle color: darker than scape. Scape color: Yellowish. A4: broader than long; as long as broad. A5: broader than long. Antennal club: formed, segments compact.

Interantennal process: not elongate. Median longitudinal elevation in frontal depression: absent. Frontal depression: concave. Frontal depression sculpture: with 3-5 complete transverse carinae. Submedian carina: strong, formed by a sharp raised carina. Submedian carina medially: without peak. Concavity across dorsal part of frontal depression: absent. Depression extending ventrally from median ocellus: absent. Upper frons: not hood-like. Malar area near antennal foramen: without carina or expansion. Malar area at mouth corner: with radiating striae. Smooth strip along posterior side of malar sulcus: absent or not consistently broad. Middle genal carina: present. Direction of middle genal carina dorsally: curving towards genal carina dorsally. Major sculpture of gena anteriorly: umbilicate-foveate. Major sculpture of gena posteriorly: umbilicate-foveate; rugose. Microsculpture of gena anteroventrally: absent. Microsculpture of gena posteroventrally: absent. Median carina extending posteriorly from hyperoccipital carina: absent. Hyperoccipital carina: not indicated medially. Lateral connection between hyperoccipital and occipital carinae: absent. Area between vertex and occipital carina: umbilicate-foveate. Occipital carina medially: uniformly rounded. Lateral corners of occipital carina: not protruding.

Lateral pronotal area: without bulge projecting towards anterior pit. Epomial corner: strong. Netrion surface anteriorly: not inflexed. Mesoscutum anteriorly: not steep. Mesoscutal median carina: present and complete. Longitudinal carina between median carina and notauli: absent. Major sculpture of medial mesoscutum anteriorly: umbilicate-foveate. Major sculpture of medial mesoscutum posteriorly: umbilicate-punctate. Microsculpture of medial mesoscutum anteriorly: granulate. Microsculpture of medial mesoscutum posteriorly: absent. Major sculpture of mesoscutellum: umbilicate-foveate; irregularly rugose. Microsculpture of mesoscutellum medially: absent. Microsculpture of mesoscutellum laterally: absent. Mesoscutellar apex: convex or straight. Setae along anterior limit of femoral depression: arising from rows of foveae; arising from tiny pits. Number of carinae crossing speculum above femoral depression: 2. Number of carinae crossing femoral depression: more than 5. Mesepimeral sulcus pits: more than 5. Metascutellum dorsally: concave. Metascutellar sculpture dorsally: smooth or with transverse carinae. Median carina of metascutellum: absent or branched. Metascutellar setae: absent. Metascutellar apex: convex or straight. Metapleuron above ventral metapleural area: crossed by carinae. Metasomal depression setae: absent. Lateral propodeal carinae anteromedially: strongly diverging. Anterior areoles of metasomal depression: absent. Anterior longitudinal carinae in metasomal depression: absent. Lateral propodeal areas: separated medially. Postmarginal vein: present. Fore wing apex: reaching middle of T5; reaching apex of T5.

T1 midlobe: with 5 longitudinal carinae. T1: without anterior bulge. T2: with straight longitudinal striae or rugae. T6: broader than long. Apical flange of T6: exposed apically. Metasomal apex: rounded. Major sculpture of T6: umbilicate-punctate; longitudinally striate or rugose. Microsculpture of T6: absent.

*Male*. Body length 2.95–5.25 mm (n=20). A5 tyloid: expanded, teardrop-shaped or sinuate. A11: broader than long. Median tooth of frontal depression: absent. Median lobe of T1: with 5 longitudinal carinae. Metasomal apex: with no distinct corners.

#### Diagnosis.

Both sexes: Middle genal carina short and angled towards genal carina dorsally. Hyperoccipital carina absent. Metascutellum concave dorsally, smooth aside from some transverse carinae. Female: A4, A5 broader than long. T1 midlobe with 5 longitudinal carinae. T6 broader than long. Male: A11 broader than long. A5 tyloid expanded, sinuate or teardrop-shaped. T7 with weakly rounded lobes posterolaterally. *Oxyscelio reflectens* is very similar *Oxyscelio crebritas*, but differs in the form of the genal ridge, the A5 tyloid in males, and the rounded apex of T7 in males. The lateral genal ridge maintains its shape and shortness even when it is weakly developed, and is usually visible as a strong ridge in dorsal view.

#### Etymology.

Latin participle not changing spelling under different genders, meaning “reflexed.” Refers to the way that the middle genal carina bends towards the posterior genal carina.

#### Link to distribution map.

[http://hol.osu.edu/map-full.html?id=275459]

#### Material Examined.

Holotype, female: **THAILAND**: Khon Kaen Prov., office, T115, Nam Phong National Park, 16°37.341'N, 102°34.467'E, 5.VII-12.VII.2006, malaise trap, K. Jaidee, OSUC 372600 (deposited in QSBG). *Paratypes*: (182 females, 156 males) **INDONESIA**: 1 female, 6 males, OSUC 369075 (CNCI); ROMEnt Spec. No. 112255-112256, 112258 (MBBJ); ROMEnt Spec. No. 112244-112245, 112254 (ROME). **LAOS**: 1 female, 4 males, OSUC 368896, 368898-368899, 368901, 368905 (CNCI). **THAILAND**: 180 females, 145 males, OSUC 247900, 247902, 320378-320379, 335824-335825, 368521, 368541 (BMNH); OSUC 368593, 368595-368596, 368598-368605, 368607-368608, 368610, 368618, 368620, 368622, 368624, 368626, 368630-368631, 368634-368637, 368639-368640, 368673, 368691, 368718, 368720, 464018, 464020-464023, 464027-464030, 464044, 464047-464049, 464051-464052, 464054-464055, 464057-464058, 464062-464064 (CNCI); OSUC 224349, 224372, 224385, 237459, 247595-247596, 247599, 247601, 247604, 247606-247607, 247616, 247636-247638, 247640-247642, 247648, 247867-247868, 247870, 247874-247876, 247888-247890, 247920, 247922, 247925, 257380, 280496, 280498, 317858-317859, 317865, 317881-317882, 320385, 320398-320399, 320411, 320413, 322064, 322067, 322077, 322087, 322090-322091, 335089, 335094, 335223-335228, 335894, 335912, 335917, 335920-335921, 335924-335925, 335928, 336017-336022, 336024-336026, 336028-336029, 336031, 336034, 336037-336041, 336706, 336710, 336737-336739, 336760, 336763, 336765-336767, 352489-352494, 361346, 361354-361355, 361918, 361929, 361940, 361949, 368506 (OSUC); OSUC 209425, 224374-224375, 237462, 247608, 247617, 247639, 247650, 247656, 247659, 247873, 247878-247879, 247886-247887, 247901, 247903-247906, 247912-247914, 247926, 252041, 257381-257382, 257394-257395, 257398, 280500-280506, 280513, 285205, 285215-285217, 285229, 317855, 317874-317875, 317880, 317883-317885, 320377, 320388-320389, 320391, 320419, 322061-322063, 322065-322066, 322068-322071, 322073-322076, 322079-322081, 322092-322095, 322099, 322116, 322123-322124, 322126-322127, 335513-335515, 335517-335522, 335826-335827, 335831-335832, 335835, 335895, 335918, 335923, 336015, 336163, 336713-336714, 336716-336717, 336762, 336764, 352488, 352509, 361218, 361287-361292, 361296, 361345, 361369, 361373, 361906, 361921-361925, 361930, 361935, 361938, 361947, 361957, 361961, 368496, 368500-368503, 368505, 368507-368510, 368516, 368518-368519, 368537, 58673 (QSBG); OSUC 320380-320381 (ROME); OSUC 247894, 285201, 322117, 335093, 335120-335121, 361950, 368539 (WINC). **VIETNAM**: 1 male, OSUC 369111 (CNCI).

#### Comments.

Two distinct size ranges occur among specimen series, which is also the case in *Oxyscelio capilli*. As in the latter species, these are assumed to be the result of different-sized hosts.

### Oxyscelio regionis

Burks sp. n.

urn:lsid:zoobank.org:act:05D99D7B-BA1E-457D-8850-46369A0F8B55

urn:lsid:biosci.ohio-state.edu:osuc_concepts:275514

http://species-id.net/wiki/Oxyscelio_regionis

**Figures 387–390.**
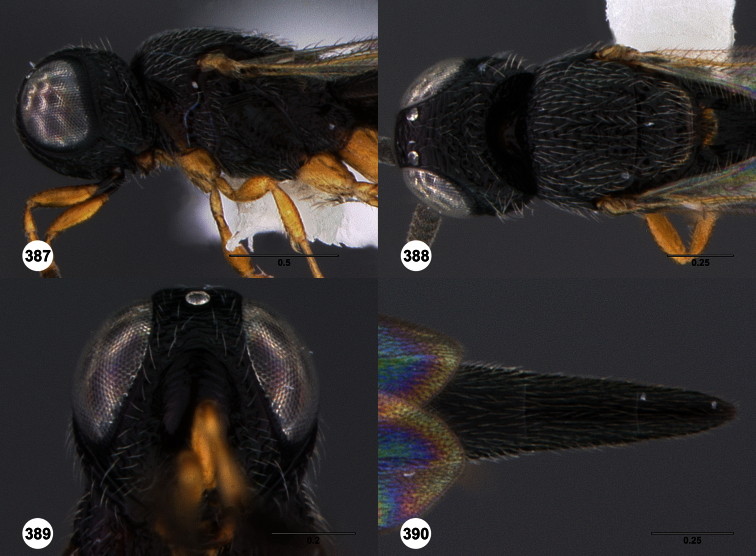
*Oxyscelio regionis* sp. n., holotype female (OSUC 247898) **387** Head and mesosoma, lateral view **388** Head and mesosoma, dorsal view **389** Head, anterior view **390** Metasoma, dorsal view. Morphbank^103^

Figures 387–390 Morphbank^103^

#### Description.

*Female*. Body length 3.6–3.75 mm (n=2).

Radicle color: darker than scape. Scape color: Yellowish. A4: broader than long; as long as broad. A5: broader than long. Antennal club: formed, segments compact.

Interantennal process: not elongate. Median longitudinal elevation in frontal depression: absent. Frontal depression: concave. Frontal depression sculpture: with 3-5 complete transverse carinae. Submedian carina: weak, shallow and rounded or formed by ledge. Submedian carina medially: without peak. Concavity across dorsal part of frontal depression: absent. Depression extending ventrally from median ocellus: absent. Upper frons: not hood-like. Malar area near antennal foramen: without carina or expansion. Malar area at mouth corner: without striae. Smooth strip along posterior side of malar sulcus: present, broad throughout its length. Middle genal carina: present. Direction of middle genal carina dorsally: parallel to eye margin. Major sculpture of gena anteriorly: umbilicate-foveate; rugose. Major sculpture of gena posteriorly: umbilicate-foveate; rugose. Microsculpture of gena anteroventrally: absent. Microsculpture of gena posteroventrally: absent. Median carina extending posteriorly from hyperoccipital carina: absent. Hyperoccipital carina: not indicated medially. Lateral connection between hyperoccipital and occipital carinae: absent. Area between vertex and occipital carina: umbilicate-foveate. Occipital carina medially: absent. Lateral corners of occipital carina: not protruding.

Lateral pronotal area: without bulge projecting towards anterior pit. Epomial corner: strong. Netrion surface anteriorly: not inflexed. Mesoscutum anteriorly: not steep. Mesoscutal median carina: present and complete. Longitudinal carina between median carina and notauli: absent. Major sculpture of medial mesoscutum anteriorly: umbilicate-foveate. Major sculpture of medial mesoscutum posteriorly: umbilicate-foveate. Microsculpture of medial mesoscutum anteriorly: granulate. Microsculpture of medial mesoscutum posteriorly: absent. Major sculpture of mesoscutellum: umbilicate-foveate; irregularly rugose. Microsculpture of mesoscutellum medially: absent. Microsculpture of mesoscutellum laterally: absent. Mesoscutellar apex: roundly concave. Setae along anterior limit of femoral depression: arising from rows of foveae. Number of carinae crossing speculum above femoral depression: 3. Number of carinae crossing femoral depression: more than 5. Mesepimeral sulcus pits: more than 5. Metascutellum dorsally: flat. Metascutellar sculpture dorsally: with scattered rugae. Median carina of metascutellum: absent or branched. Metascutellar setae: absent. Metascutellar apex: convex or straight. Metapleuron above ventral metapleural area: foveate or rugose. Metasomal depression setae: absent. Lateral propodeal carinae anteromedially: strongly diverging. Anterior areoles of metasomal depression: absent. Anterior longitudinal carinae in metasomal depression: absent. Lateral propodeal areas: separated medially. Postmarginal vein: present. Fore wing apex: reaching middle of T4.

T1 midlobe: obscured by other raised sculpture. T1: with small rounded anterior bulge, not reaching metascutellum. T2: with straight longitudinal striae or rugae. T6: longer than broad. Apical flange of T6: not exposed apically. Metasomal apex: rounded. Major sculpture of T6: umbilicate-punctate. Microsculpture of T6: absent.

*Male*. Unknown.

#### Diagnosis.

Female: Upper frons one or more extra carinae dorsal to submedian carina. Hyperoccipital carina indicated by rugae. Mesoscutellum without granulate sculpture. Mesofemoral depression crossed by more than 3 carinae below speculum. Metascutellum subrectangular, with scattered weak rugae. T1 midlobe with long anterior bulge. T2 without sublateral depressions or curved striae. T6 longer than broad, tapering to a rounded apex.

#### Etymology.

Latin noun, genitive case, meaning “boundary.”

#### Link to distribution map.

[http://hol.osu.edu/map-full.html?id=275514]

#### Material examined.

Holotype, female: **THAILAND**: Loei Prov., Koke Hin Ngam, T492, Phu Kradung National Park, 16°51.817'N, 101°50.704'E, 270m, 30.VIII-6.IX.2006, malaise trap, S. Khonglasae, OSUC 247898 (deposited in QSBG). *Paratype*: **THAILAND**: 1 female, OSUC 267523 (OSUC).

#### Comments.

*Oxyscelio regionis* is striking in that it is very small-bodied but with an elongate head and metasoma.

### Oxyscelio rugosus

(Kieffer)

urn:lsid:zoobank.org:act:B7C72C9B-958C-4D5B-8CCB-C08E1419AF86

urn:lsid:biosci.ohio-state.edu:osuc_concepts:5032

http://species-id.net/wiki/Oxyscelio_rugosus

**Figures 391–394.**
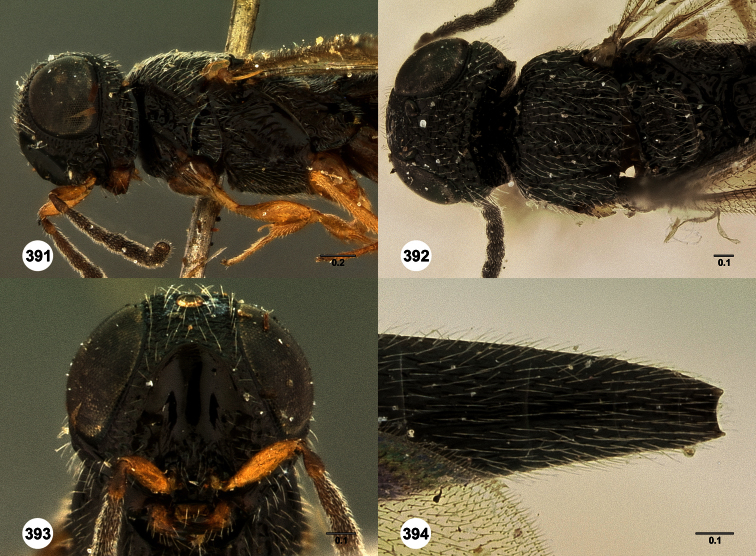
*Oxyscelio rugosus* (Kieffer), holotype male (OSUC 365209) **391** Head and mesosoma, lateral view **392** Head and mesosoma, dorsal view **393** Head, anterior view **394** Metasomal apex, dorsal view. Morphbank^104^

Figures 391–394 Morphbank^104^

Dicroteleia rugosa Kieffer, 1908: 92 (original description); Kieffer 1926: 387, 388 (description, keyed).
Chromoteleia (Oxyscelio) rugosa (Kieffer): Kieffer 1910a: 313 (generic transfer, subgeneric assignment, keyed).
Oxyscelio (Dicroteleia) rugosa (Kieffer): Kieffer 1910b: 68 (generic transfer, subgeneric assignment).
Oxyscelio rugosus (Kieffer): Dodd 1931: 76 (generic transfer); Masner 1976: 24 (description).

#### Description.

*Female*. Unknown.

*Male*. Body length 4.05 mm (n=1).

Radicle color: same color as scape. Scape color: Yellowish. A5 tyloid: carina-like, not expanded. A11: longer than broad.

Interantennal process: not elongate. Median longitudinal elevation in frontal depression: absent. Frontal depression: concave. Frontal depression sculpture: with 3 or more broadly interrupted transverse carinae. Submedian carina: strong, formed by a sharp raised carina. Submedian carina medially: without peak. Median tooth of frontal depression: absent. Concavity across dorsal part of frontal depression: absent. Depression extending ventrally from median ocellus: absent. Upper frons: not hood-like. Malar area near antennal foramen: with oblique tooth-like flange (facial nubbin). Malar area at mouth corner: with radiating striae. Smooth strip along posterior side of malar sulcus: present, broad throughout its length. Middle genal carina: absent. Direction of middle genal carina dorsally: absent (replace with question mark). Major sculpture of gena anteriorly: umbilicate-foveate. Major sculpture of gena posteriorly: umbilicate-foveate. Microsculpture of gena anteroventrally: absent. Microsculpture of gena posteroventrally: absent. Median carina extending posteriorly from hyperoccipital carina: absent. Hyperoccipital carina: indicated by rugae. Lateral connection between hyperoccipital and occipital carinae: absent. Area between vertex and occipital carina: umbilicate-foveate; irregularly rugose. Occipital carina medially: slightly convex, flatter medially than laterally. Lateral corners of occipital carina: sharp and protruding.

Lateral pronotal area: without bulge projecting towards anterior pit. Epomial corner: strong. Netrion surface anteriorly: not inflexed. Mesoscutum anteriorly: steep. Mesoscutal median carina: present and complete. Longitudinal carina between median carina and notauli: absent. Major sculpture of medial mesoscutum anteriorly: umbilicate-foveate. Major sculpture of medial mesoscutum posteriorly: umbilicate-foveate. Microsculpture of medial mesoscutum anteriorly: granulate. Microsculpture of medial mesoscutum posteriorly: absent. Major sculpture of mesoscutellum: umbilicate-foveate; longitudinally rugose. Microsculpture of mesoscutellum medially: absent. Microsculpture of mesoscutellum laterally: punctate. Mesoscutellar apex: convex or straight. Setae along anterior limit of femoral depression: arising from rows of foveae. Number of carinae crossing speculum above femoral depression: 2. Number of carinae crossing femoral depression: 3-5. Mesepimeral sulcus pits: 3-5. Metascutellum dorsally: concave. Metascutellar sculpture dorsally: smooth or with transverse carinae. Median carina of metascutellum: absent or branched. Metascutellar setae: absent. Metascutellar apex: convex or straight. Metapleuron above ventral metapleural area: crossed by carinae. Metasomal depression setae: absent. Anterior areoles of metasomal depression: absent. Anterior longitudinal carinae in metasomal depression: absent. Lateral propodeal areas: separated medially. Postmarginal vein: present.

Median lobe of T1: with 5 longitudinal carinae. Metasomal apex: with acuminate lateral corners.

#### Diagnosis.

Male: A11 longer than broad. Face with broad oblique expanded flange between antennal foramen and eye. Metascutellum not longer than broad, nearly square, with broad concave area medially. T2 with sublateral depressions bordered medially by a very strong carina.

#### Link to distribution map.

[http://hol.osu.edu/map-full.html?id=5032]

#### Material examined.

Holotype, male, *Dicroteleia rugosa*: **INDONESIA**: Jakarta Raya Special Dist., Jakarta (Batavia), XI-1907, E. Jacobson, RMNH 0002 (deposited in RMNH).

#### Comments.

*Oxyscelio rugosus* possesses the characters of a few different distinctive species groups, and therefore is difficult to place based on the strength of the single existing specimen. Because of the T2 sublateral depression medially bordered by a strong carina, *Oxyscelio rugosus* is provisionally placed in the *fossarum*-group.

### Oxyscelio sinuum

Burks sp. n.

urn:lsid:zoobank.org:act:2281CD64-1094-4081-8DF4-7C4296E0B115

urn:lsid:biosci.ohio-state.edu:osuc_concepts:275539

http://species-id.net/wiki/Oxyscelio_sinuum

**Figures 395–398.**
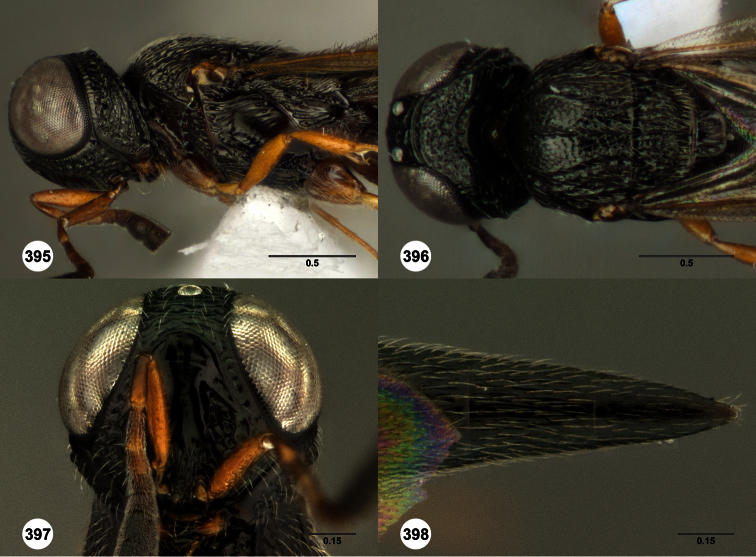
*Oxyscelio sinuum* sp. n., paratype female (OSUC 352505) **395** Head and mesosoma, lateral view **396** Head and mesosoma, dorsal view **397** Head, anterior view **398** Metasoma, dorsal view. Morphbank^105^

Figures 395–398 Morphbank^105^

#### Description.

*Female*. Body length 4.1–4.65 mm (n=4).

Radicle color: same color as scape. Scape color: Brown. A4: longer than broad. A5: broader than long. Antennal club: formed, segments compact.

Interantennal process: not elongate. Median longitudinal elevation in frontal depression: absent. Frontal depression: concave. Frontal depression sculpture: with 3 or more broadly interrupted transverse carinae. Submedian carina: strong, formed by a sharp raised carina. Submedian carina medially: with sharp peak. Concavity across dorsal part of frontal depression: absent. Depression extending ventrally from median ocellus: absent. Upper frons: not hood-like. Malar area near antennal foramen: without carina or expansion. Malar area at mouth corner: with radiating striae. Smooth strip along posterior side of malar sulcus: absent or not consistently broad. Middle genal carina: present. Direction of middle genal carina dorsally: parallel to eye margin. Major sculpture of gena anteriorly: umbilicate-foveate; rugose. Major sculpture of gena posteriorly: umbilicate-foveate; rugose. Microsculpture of gena anteroventrally: absent. Microsculpture of gena posteroventrally: absent. Median carina extending posteriorly from hyperoccipital carina: absent. Hyperoccipital carina: indicated by rugae. Lateral connection between hyperoccipital and occipital carinae: present as a weak elevation. Area between vertex and occipital carina: umbilicate-foveate. Occipital carina medially: divided into concave halves, meeting at median peak. Lateral corners of occipital carina: sharp and protruding.

Lateral pronotal area: without bulge projecting towards anterior pit. Epomial corner: strong. Netrion surface anteriorly: not inflexed. Mesoscutum anteriorly: not steep. Mesoscutal median carina: present and complete. Longitudinal carina between median carina and notauli: absent. Major sculpture of medial mesoscutum anteriorly: umbilicate-foveate. Major sculpture of medial mesoscutum posteriorly: umbilicate-foveate. Microsculpture of medial mesoscutum anteriorly: absent. Microsculpture of medial mesoscutum posteriorly: absent. Major sculpture of mesoscutellum: umbilicate-foveate; longitudinally rugose. Microsculpture of mesoscutellum medially: absent. Microsculpture of mesoscutellum laterally: absent. Mesoscutellar apex: convex or straight. Setae along anterior limit of femoral depression: arising from rows of foveae. Number of carinae crossing speculum above femoral depression: 3. Number of carinae crossing femoral depression: 3-5. Mesepimeral sulcus pits: 3-5. Metascutellum dorsally: flat. Metascutellar sculpture dorsally: with scattered rugae. Median carina of metascutellum: absent or branched. Metascutellar setae: absent. Metascutellar apex: convex or straight; weakly emarginate. Metapleuron above ventral metapleural area: crossed by carinae. Metasomal depression setae: absent. Lateral propodeal carinae anteromedially: strongly diverging. Anterior areoles of metasomal depression: absent. Anterior longitudinal carinae in metasomal depression: absent. Lateral propodeal areas: separated medially. Postmarginal vein: present. Fore wing apex: reaching apex of T4.

T1 midlobe: obscured by other raised sculpture. T1: with long anterior bulge, reaching metascutellum. T2: with straight longitudinal striae or rugae. T6: longer than broad. Apical flange of T6: not exposed apically. Metasomal apex: rounded. Major sculpture of T6: umbilicate-punctate. Microsculpture of T6: granulate.

*Male*. Unknown.

#### Diagnosis.

Female: Occipital carina complete as a distinct carina, but medial portions concave and meeting at a rounded peak. Mesoscutellum with many narrow longitudinal rugae. Netrion not concave. Metascutellum broad and subrectangular, not concave. Fore wings long enough to reach apex of T4. T1 midlobe with anterior horn. T6 longer than broad and tapering to a narrow but rounded apex.

#### Etymology.

Latin noun, 4th declension, genitive case, referring to the medially sinuate occipital carina.

#### Link to distribution map.

[http://hol.osu.edu/map-full.html?id=275539]

#### Material examined.

Holotype, female: **THAILAND**: Nakhon Si Thammarat Prov., TV aerial, T3103, Namtok Yong National Park, 08°14.262'N, 99°48.289'E, 966m, 18.VIII–25.VIII.2008, malaise trap, Paiboon, OSUC 352506 (deposited in QSBG). *Paratypes*: (3 females) **SINGAPORE**: 1 female, OSUC 376755 (MCZC). **THAILAND**: 2 females, OSUC 352505 (OSUC); OSUC 361953 (QSBG).

#### Comments.

*Oxyscelio sinuum* resembles some other species with a complete, sinuate occipital carina. It differs in a combination of characters, including the flat, rugose metascutellum.

### Oxyscelio spinae

Burks sp. n.

urn:lsid:zoobank.org:act:ED1E5D11-08E8-4A4A-9541-B4198F046C6A

urn:lsid:biosci.ohio-state.edu:osuc_concepts:275505

http://species-id.net/wiki/Oxyscelio_spinae

**Figures 399–403.**
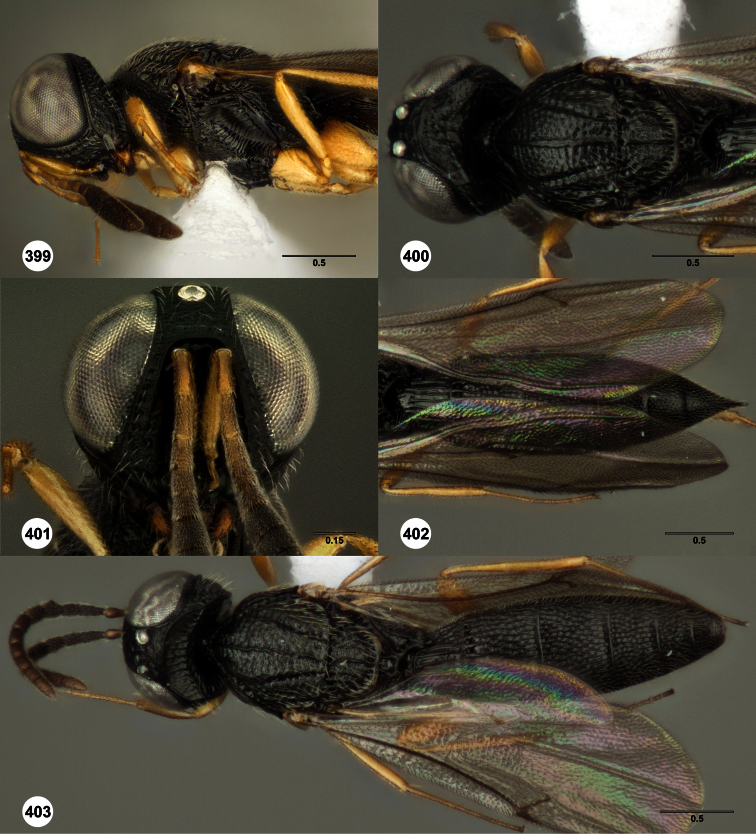
*Oxyscelio spinae* sp. n., holotype female (OSUC 361212) **399** Head and mesosoma, lateral view **400** Head and mesosoma, dorsal view **401** Head, anterior view **402** Metasoma, dorsal view. Paratype male (OSUC 335117) **403** Body, dorsal view. Morphbank^106^

Figures 399–403 Morphbank^106^

#### Description.

*Female*. Body length 4–4.1 mm (n=2).

Radicle color: same color as scape. Scape color: Yellowish. A4: longer than broad. A5: longer than broad. Antennal club: formed, segments compact.

Interantennal process: not elongate. Median longitudinal elevation in frontal depression: absent. Frontal depression: concave. Frontal depression sculpture: with 3 or more broadly interrupted transverse carinae. Submedian carina: strong, formed by a sharp raised carina. Submedian carina medially: without peak. Concavity across dorsal part of frontal depression: absent. Depression extending ventrally from median ocellus: absent. Upper frons: not hood-like. Malar area near antennal foramen: with oblique tooth-like flange (facial nubbin). Malar area at mouth corner: with radiating striae. Smooth strip along posterior side of malar sulcus: absent or not consistently broad. Middle genal carina: absent. Direction of middle genal carina dorsally: absent (replace with question mark). Major sculpture of gena anteriorly: rugose; umbilicate-punctate. Major sculpture of gena posteriorly: rugose; umbilicate-punctate. Microsculpture of gena anteroventrally: granulate. Microsculpture of gena posteroventrally: granulate. Median carina extending posteriorly from hyperoccipital carina: absent. Hyperoccipital carina: complete, continuous with anterior genal carina. Lateral connection between hyperoccipital and occipital carinae: present as a distinct carina. Area between vertex and occipital carina: irregularly rugose. Occipital carina medially: divided into concave halves, meeting at median peak. Lateral corners of occipital carina: sharp and protruding.

Lateral pronotal area: without bulge projecting towards anterior pit. Epomial corner: weak. Netrion surface anteriorly: not inflexed. Mesoscutum anteriorly: not steep. Mesoscutal median carina: present and complete. Longitudinal carina between median carina and notauli: absent. Major sculpture of medial mesoscutum anteriorly: umbilicate-foveate. Major sculpture of medial mesoscutum posteriorly: umbilicate-foveate. Microsculpture of medial mesoscutum anteriorly: granulate. Microsculpture of medial mesoscutum posteriorly: absent. Major sculpture of mesoscutellum: umbilicate-foveate. Microsculpture of mesoscutellum medially: absent. Microsculpture of mesoscutellum laterally: absent. Mesoscutellar apex: convex or straight. Setae along anterior limit of femoral depression: arising from tiny pits. Number of carinae crossing speculum above femoral depression: 4. Number of carinae crossing femoral depression: more than 5. Mesepimeral sulcus pits: 3-5. Metascutellum dorsally: concave. Metascutellar sculpture dorsally: smooth or with transverse carinae. Median carina of metascutellum: absent or branched. Metascutellar setae: absent. Metascutellar apex: deeply emarginate. Metapleuron above ventral metapleural area: crossed by carinae. Metasomal depression setae: absent. Lateral propodeal carinae anteromedially: strongly diverging. Anterior areoles of metasomal depression: one or more areoles present. Anterior longitudinal carinae in metasomal depression: median carina present. Lateral propodeal areas: separated medially. Postmarginal vein: present. Fore wing apex: reaching apex of T6.

T1 midlobe: obscured by other raised sculpture. T1: with small rounded anterior bulge, not reaching metascutellum. T2: with straight longitudinal striae or rugae. T6: longer than broad. Apical flange of T6: exposed apically. Metasomal apex: tapering to a sharp point. Major sculpture of T6: umbilicate-punctate; longitudinally striate or rugose. Microsculpture of T6: absent.

*Male*. Body length 3.75–3.9 mm (n=5). A5 tyloid: carina-like, not expanded. A11: longer than broad. Median tooth of frontal depression: absent. Median lobe of T1: with 5 longitudinal carinae. Metasomal apex: with no distinct corners.

#### Diagnosis.

Both sexes: Face with oblique expanded flange between antennal foramen and eye. Hyperoccipital carina present, continuous with an anterior genal carina, connected with occipital carina by a distinct longitudinal carina. Metascutellum slightly emarginate, posterior corners rounded and lobe-like. Female: T1 midlobe with 6 or more longitudinal carinae. Metasoma with a greatly elongate spine-like apex. Male: A11 longer than broad. T7 with weakly rounded lobes posterolaterally.

#### Etymology.

Latin noun, genitive case, meaning “spine.” Refers to the elongate and sharply pointed metasomal apex in females.

#### Link to distribution map.

[http://hol.osu.edu/map-full.html?id=275505]

#### Material examined.

Holotype, female: **THAILAND**: Nakhon Si Thammarat Prov., road to Mhen Mt., 150m from Nern 499, T3100, Namtok Yong National Park, 08°16.959'N, 99°39.149'E, 499m, 6.VIII-13.VIII.2008, malaise trap, S. Samnaokan, OSUC 361212 (deposited in QSBG). *Paratypes*:(2 females, 5 males) **THAILAND**: 1 female, 5 males, OSUC 368752, 368754 (CNCI); OSUC 335117, 336123 (OSUC); OSUC 352922, 361357 (QSBG). **VIETNAM**: 1 female, OSUC 119929 (OSUC).

#### Comments.

Females of *Oxyscelio spinae* are very distinctive in having a very long, sharply pointed metasomal apex, including S6 and the apical rim of T6. This species is similar to *Oxyscelio crateris* in some characters of the occiput and metascutellum, and may be closely related to it.

### Oxyscelio spinosiceps

(Kieffer)

urn:lsid:zoobank.org:act:B4A47889-6AA0-4306-9F49-3BBAEE6382DE

urn:lsid:biosci.ohio-state.edu:osuc_concepts:5036

http://species-id.net/wiki/Oxyscelio_spinosiceps

**Figures 404–409.**
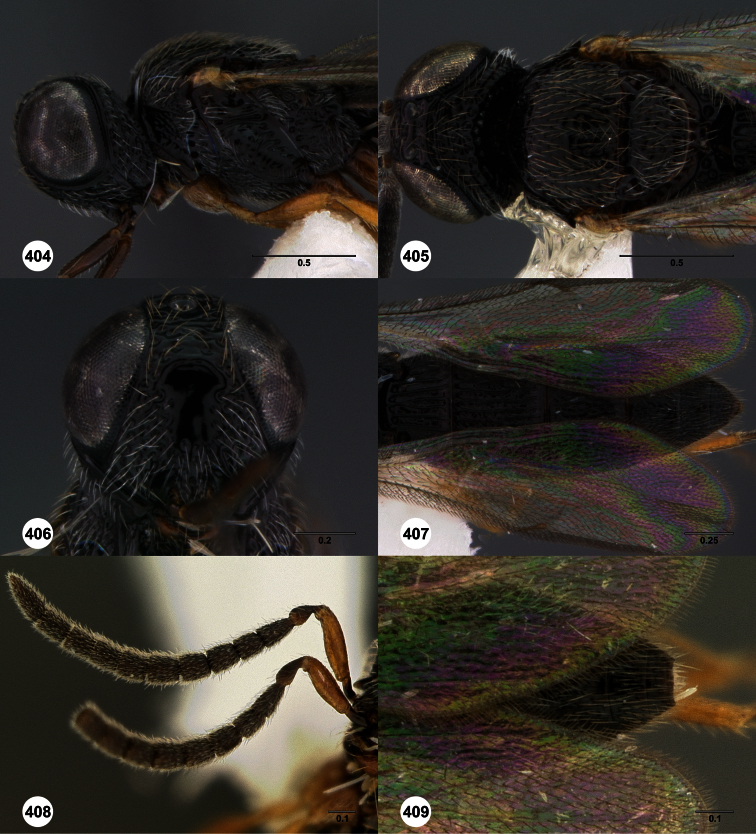
*Oxyscelio spinosiceps* (Kieffer), female (OSUC 369052) **404** Head and mesosoma, lateral view **405** Head, anterior view.Female (OSUC 369053) **406** Head and mesosoma, dorsal view. **407** Metasoma, dorsal view. Paratype male (ROMEnt Spec. No. 112228) **408** Antenna **409** Metasoma, dorsal view. Morphbank^107^

Figures 404–409 Morphbank^107^

Psilanteris spinosiceps Kieffer, 1916: 177, 178 (original description, keyed).
Camptoteleia spinosiceps (Kieffer): Kieffer 1926: 379, 386 (generic transfer, description, keyed).
Oxyscelio spinosiceps (Kieffer): Dodd 1931: 76 (generic transfer); Masner 1976: 24 (type information).

#### Description.

*Female*. Body length 3.3–3.75 mm (n=4).

Radicle color: darker than scape. Scape color: Yellowish; Brown. A4: broader than long. A5: broader than long. Antennal club: formed, segments compact.

Interantennal process: not elongate. Median longitudinal elevation in frontal depression: absent. Frontal depression: concave; flat. Frontal depression sculpture: without transverse or oblique carinae below submedian carina. Submedian carina: weak, shallow and rounded or formed by ledge. Submedian carina medially: without peak. Concavity across dorsal part of frontal depression: absent. Depression extending ventrally from median ocellus: absent. Upper frons: not hood-like. Malar area near antennal foramen: with oblique tooth-like flange (facial nubbin). Malar area at mouth corner: with radiating striae. Smooth strip along posterior side of malar sulcus: absent or not consistently broad. Middle genal carina: absent. Direction of middle genal carina dorsally: absent (replace with question mark). Major sculpture of gena anteriorly: umbilicate-foveate. Major sculpture of gena posteriorly: umbilicate-foveate. Microsculpture of gena anteroventrally: absent. Microsculpture of gena posteroventrally: absent. Median carina extending posteriorly from hyperoccipital carina: absent. Hyperoccipital carina: indicated by rugae. Lateral connection between hyperoccipital and occipital carinae: absent. Area between vertex and occipital carina: umbilicate-foveate; irregularly rugose. Occipital carina medially: uniformly rounded. Lateral corners of occipital carina: not protruding.

Lateral pronotal area: without bulge projecting towards anterior pit. Epomial corner: strong. Netrion surface anteriorly: not inflexed. Mesoscutum anteriorly: not steep. Mesoscutal median carina: present and complete. Longitudinal carina between median carina and notauli: absent. Major sculpture of medial mesoscutum anteriorly: umbilicate-punctate. Major sculpture of medial mesoscutum posteriorly: umbilicate-foveate. Microsculpture of medial mesoscutum anteriorly: granulate. Microsculpture of medial mesoscutum posteriorly: absent. Major sculpture of mesoscutellum: umbilicate-foveate. Microsculpture of mesoscutellum medially: absent. Microsculpture of mesoscutellum laterally: absent. Mesoscutellar apex: convex or straight. Setae along anterior limit of femoral depression: arising from rows of foveae. Number of carinae crossing speculum above femoral depression: 3. Number of carinae crossing femoral depression: more than 5. Mesepimeral sulcus pits: more than 5. Metascutellum dorsally: concave. Metascutellar sculpture dorsally: smooth or with transverse carinae. Median carina of metascutellum: absent or branched. Metascutellar setae: with many dorsal setae. Metascutellar apex: deeply emarginate. Metapleuron above ventral metapleural area: crossed by carinae. Metasomal depression setae: absent. Lateral propodeal carinae anteromedially: strongly diverging. Anterior areoles of metasomal depression: one or more areoles present. Anterior longitudinal carinae in metasomal depression: absent; median carina present. Lateral propodeal areas: separated medially. Postmarginal vein: present. Fore wing apex: reaching beyond T6.

T1 midlobe: with 5 longitudinal carinae. T1: without anterior bulge. T2: with straight longitudinal striae or rugae. T6: broader than long. Apical flange of T6: exposed apically. Metasomal apex: rounded. Major sculpture of T6: umbilicate-punctate. Microsculpture of T6: absent.

*Male*. Body length 3.2–3.35 mm (n=16). A5 tyloid: carina-like, not expanded. A11: as long as broad. Median tooth of frontal depression: absent. Median lobe of T1: with 5 longitudinal carinae. Metasomal apex: with acuminate lateral corners.

#### Diagnosis.

Both sexes: Face with flange between antennal foramen and eye; frontal depression flat or shallowly concave. Hyperoccipital carina defined by ruga, but continuous with anterior genal carina. Metascutellum with dorsal setae. Metasomal depression long and extensively sculptured; lateral propodeal carinae broadly separated anteriorly. Female: A4, A5 broader than long. T1 midlobe with 5 longitudinal carinae. Male: T1 midlobe with 5 longitudinal carinae. T7 with tiny, sharp and weakly protruding posterolateral corners. *Oxyscelio spinosiceps* is similar to *Oxyscelio nubbin* in having a flange between the antennal foramen and eye, but differs in many characters. The frontal depression is flat and not laterally carinate. A similar flange occurs in *Oxyscelio marginalis*, but these species do not otherwise resemble one other.

#### Link to distribution map.

[http://hol.osu.edu/map-full.html?id=5036]

#### Material examined.

Holotype, female, *Psilanteris spinosiceps*: **PHILIPPINES**: Laguna Prov., Luzon Isl., Mount Makiling, no date, Baker, USNM Type No. 70482 (deposited in USNM). *Other material*: **PHILIPPINES**: 4 females, 16 males, OSUC 369052-369053 (CNCI); OSUC 247965, 352919, ROMEnt Spec. No. 112205, ROMEnt Spec. No. 112208, ROMEnt Spec. No. 112209, ROMEnt Spec. No. 112219, ROMEnt Spec. No. 112227, ROMEnt Spec. No. 112228, ROMEnt Spec. No. 112229, ROMEnt Spec. No. 112230, ROMEnt Spec. No. 112233, ROMEnt Spec. No. 112683, ROMEnt Spec. No. 112686, ROMEnt Spec. No. 112687, ROMEnt Spec. No. 112688, ROMEnt Spec. No. 112690, ROMEnt Spec. No. 112692 (ROME); OSUC 268218 (USNM).

### Oxyscelio striarum

Burks sp. n.

urn:lsid:zoobank.org:act:DAE4E3B3-8F03-4E08-88F1-DD684EE132A1

urn:lsid:biosci.ohio-state.edu:osuc_concepts:275511

http://species-id.net/wiki/Oxyscelio_striarum

**Figures 410–413.**
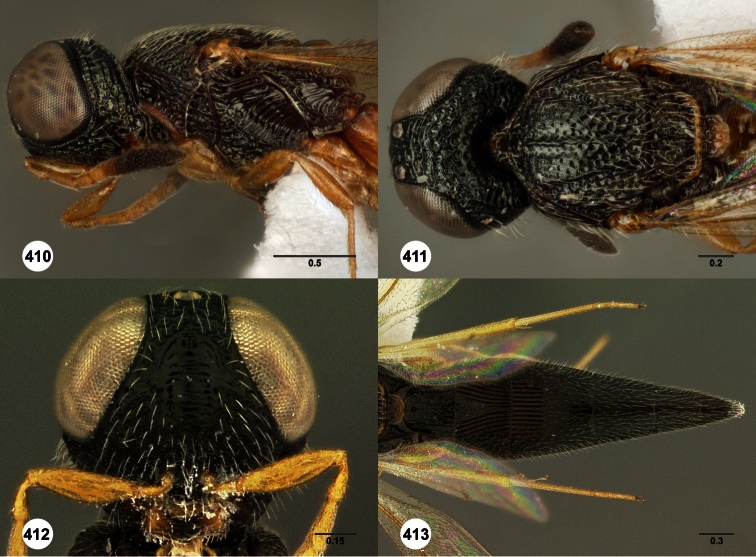
*Oxyscelio striarum* sp. n., paratype female (OSUC 257067) **410** Head and mesosoma, lateral view **411** Head and mesosoma, dorsal view. Holotype female (OSUC 368958) **412** Head, anterior view **413** Metasoma, dorsal view. Morphbank^108^

Figures 410–413 Morphbank^108^

#### Description.

*Female*. Body length 4.8–5.45 mm (n=12).

Radicle color: same color as scape. Scape color: Yellowish. A4: longer than broad. A5: longer than broad. Antennal club: formed, segments compact.

Interantennal process: not elongate. Median longitudinal elevation in frontal depression: absent. Frontal depression: concave. Frontal depression sculpture: with 3-5 complete transverse carinae. Submedian carina: weak, shallow and rounded or formed by ledge. Submedian carina medially: without peak. Concavity across dorsal part of frontal depression: absent. Depression extending ventrally from median ocellus: absent. Upper frons: not hood-like. Malar area near antennal foramen: without carina or expansion. Malar area at mouth corner: without striae. Smooth strip along posterior side of malar sulcus: present, broad throughout its length. Middle genal carina: present. Direction of middle genal carina dorsally: parallel to eye margin. Major sculpture of gena anteriorly: umbilicate-foveate. Major sculpture of gena posteriorly: umbilicate-foveate; rugose. Microsculpture of gena anteroventrally: absent. Microsculpture of gena posteroventrally: absent. Median carina extending posteriorly from hyperoccipital carina: absent. Hyperoccipital carina: indicated by rugae. Lateral connection between hyperoccipital and occipital carinae: present as a weak elevation. Area between vertex and occipital carina: umbilicate-foveate. Occipital carina medially: absent. Lateral corners of occipital carina: sharp and protruding.

Lateral pronotal area: without bulge projecting towards anterior pit. Epomial corner: strong. Netrion surface anteriorly: not inflexed. Mesoscutum anteriorly: not steep. Mesoscutal median carina: present and complete. Longitudinal carina between median carina and notauli: absent. Major sculpture of medial mesoscutum anteriorly: umbilicate-foveate. Major sculpture of medial mesoscutum posteriorly: umbilicate-foveate. Microsculpture of medial mesoscutum anteriorly: granulate. Microsculpture of medial mesoscutum posteriorly: absent. Major sculpture of mesoscutellum: umbilicate-foveate; longitudinally rugose. Microsculpture of mesoscutellum medially: absent. Microsculpture of mesoscutellum laterally: absent. Mesoscutellar apex: convex or straight. Setae along anterior limit of femoral depression: arising from rows of foveae. Number of carinae crossing speculum above femoral depression: 2. Number of carinae crossing femoral depression: more than 5. Mesepimeral sulcus pits: more than 5. Metascutellum dorsally: flat. Metascutellar sculpture dorsally: with scattered rugae. Median carina of metascutellum: absent or branched. Metascutellar setae: absent. Metascutellar apex: convex or straight. Metapleuron above ventral metapleural area: crossed by carinae. Metasomal depression setae: absent. Lateral propodeal carinae anteromedially: strongly diverging. Anterior areoles of metasomal depression: absent. Anterior longitudinal carinae in metasomal depression: absent. Lateral propodeal areas: separated medially. Postmarginal vein: absent. Fore wing apex: reaching apex of T4; reaching middle of T5.

T1 midlobe: obscured by other raised sculpture. T1: with long anterior bulge, reaching metascutellum. T2: with strong set of curved striae. T6: longer than broad. Apical flange of T6: not exposed apically. Metasomal apex: rounded. Major sculpture of T6: umbilicate-punctate; longitudinally striate or rugose. Microsculpture of T6: granulate.

*Male*. Unknown.

#### Diagnosis.

Female: Mesoscutellum without granulate areas. Metascutellum broad, rugose. T1 with a moderately developed anterior horn that causes the longitudinal carinae to become broad and indistinct anteriorly. T2 and T3 with long, approximated curved striate that for much of their length are not separated by setal pits. Fore wings long enough to reach apex of T4 or middle of T5.

#### Etymology.

Latin noun, genitive case, meaning “furrows.” Refers to the distinctive striae on T2 and T3.

#### Link to distribution map.

[http://hol.osu.edu/map-full.html?id=275511]

#### Material examined.

Holotype, female: **INDONESIA**: Kalimantan Barat Prov., Cabang Panti Research Station, 1° rainforest / alluvial closed canopy, IIS 910126, Gunung Palung National Park, 01°15'S, 110°05'E, 100-400m, 15.VI-15.VIII.1991, malaise trap/pan trap, Darling, Rosichon & Sutrisno, OSUC 368958 (deposited in MBBJ). *Paratypes*: (13 females) **BRUNEI**: 1 female, OSUC 376637 (BMNH). **INDONESIA**: 4 females, OSUC 368946 (CNCI); OSUC 257067 (MBBJ); OSUC 257085, 257089 (ROME). **MALAYSIA**: 6 females, OSUC 376608-376609 (BMNH); OSUC 369029, 369058, 369062 (CNCI); OSUC 453788 (OSUC). **THAILAND**: 2 females, OSUC 368765 (CNCI); UCRC ENT 135267 (UCRC).

#### Comments.

*Oxyscelio striarum* varies in metasomal length, with some specimens having a long and nearly parallel-sided T5 but with others having a much shorter and broader metasomal apex. There is a continuum between these two extremes, throughout the distribution of this species, such that they could not be satisfactorily separated into distinct species.

### Oxyscelio tecti

Burks sp. n.

urn:lsid:zoobank.org:act:70655016-CE83-44DE-8FDA-176C28387B6B

urn:lsid:biosci.ohio-state.edu:osuc_concepts:275550

http://species-id.net/wiki/Oxyscelio_tecti

**Figures 414–419.**
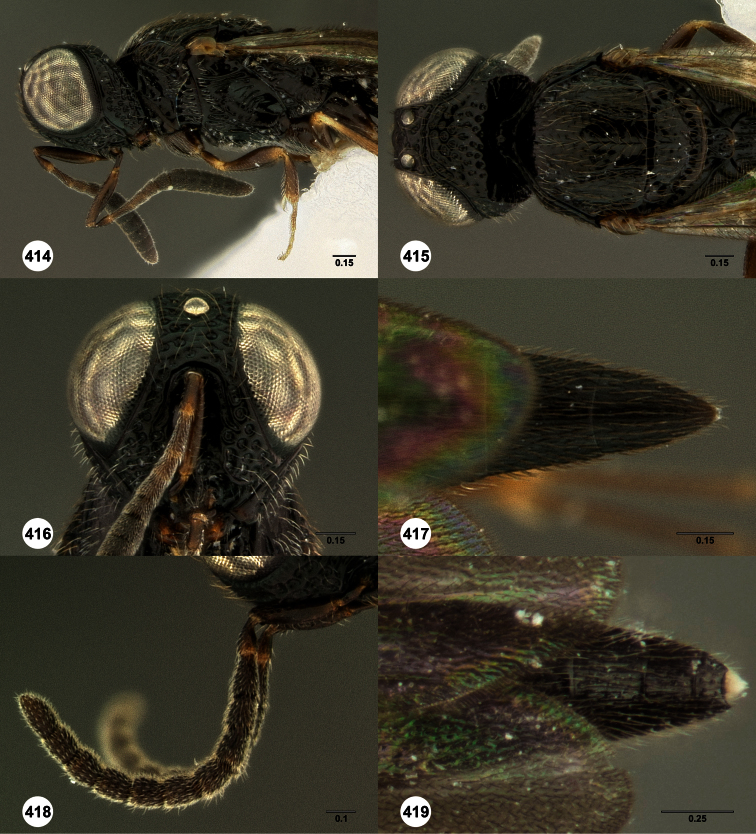
*Oxyscelio tecti* sp. n., holotype female (OSUC 368938) **414** Head and mesosoma, lateral view **415** Head and mesosoma, dorsal view **416** Head, anterior view **417** Metasoma, dorsal view. Paratype male (OSUC 368940) **418** Antenna. Paratype male (OSUC 228709) **419** Metasoma, dorsal view. Morphbank^109^

Figures 414–419 Morphbank^109^

#### Description.

*Female*. Body length 4.45 mm (n=1).

Radicle color: same color as scape. Scape color: Brown. A4: longer than broad. A5: longer than broad. Antennal club: formed, segments compact.

Interantennal process: not elongate. Median longitudinal elevation in frontal depression: absent. Frontal depression: concave. Frontal depression sculpture: without transverse or oblique carinae below submedian carina. Submedian carina: weak, shallow and rounded or formed by ledge. Submedian carina medially: without peak. Concavity across dorsal part of frontal depression: absent. Depression extending ventrally from median ocellus: absent. Upper frons: not hood-like. Malar area near antennal foramen: without carina or expansion. Malar area at mouth corner: without striae. Smooth strip along posterior side of malar sulcus: absent or not consistently broad. Middle genal carina: absent. Direction of middle genal carina dorsally: parallel to eye margin. Major sculpture of gena anteriorly: umbilicate-foveate. Major sculpture of gena posteriorly: umbilicate-foveate. Microsculpture of gena anteroventrally: absent. Microsculpture of gena posteroventrally: absent. Median carina extending posteriorly from hyperoccipital carina: absent. Hyperoccipital carina: indicated by rugae. Lateral connection between hyperoccipital and occipital carinae: present as a distinct carina. Area between vertex and occipital carina: umbilicate-foveate. Occipital carina medially: absent. Lateral corners of occipital carina: sharp and protruding.

Lateral pronotal area: without bulge projecting towards anterior pit. Epomial corner: strong. Netrion surface anteriorly: not inflexed. Mesoscutum anteriorly: not steep. Mesoscutal median carina: present and complete. Longitudinal carina between median carina and notauli: absent. Major sculpture of medial mesoscutum anteriorly: umbilicate-punctate. Major sculpture of medial mesoscutum posteriorly: umbilicate-punctate. Microsculpture of medial mesoscutum anteriorly: absent; granulate. Microsculpture of medial mesoscutum posteriorly: absent. Major sculpture of mesoscutellum: umbilicate-punctate. Microsculpture of mesoscutellum medially: absent. Microsculpture of mesoscutellum laterally: punctate. Mesoscutellar apex: convex or straight. Setae along anterior limit of femoral depression: arising from rows of foveae. Number of carinae crossing speculum above femoral depression: 2. Number of carinae crossing femoral depression: 3-5. Mesepimeral sulcus pits: 3-5. Metascutellum dorsally: flat. Metascutellar sculpture dorsally: with scattered rugae. Median carina of metascutellum: absent or branched; straight, unbranched carina present. Metascutellar setae: absent. Metascutellar apex: convex or straight. Metapleuron above ventral metapleural area: crossed by carinae. Metasomal depression setae: absent. Lateral propodeal carinae anteromedially: strongly diverging. Anterior areoles of metasomal depression: absent. Anterior longitudinal carinae in metasomal depression: absent. Lateral propodeal areas: separated medially. Postmarginal vein: present. Fore wing apex: reaching middle of T5.

T1 midlobe: obscured by other raised sculpture. T1: without anterior bulge. T2: with straight longitudinal striae or rugae. T6: longer than broad. Apical flange of T6: not exposed apically. Metasomal apex: rounded. Major sculpture of T6: umbilicate-punctate. Microsculpture of T6: granulate.

*Male*. Body length 4.15–4.2 mm (n=2). A5 tyloid: carina-like, not expanded. A11: longer than broad. Median tooth of frontal depression: absent. Median lobe of T1: with 5 longitudinal carinae. Metasomal apex: with acuminate lateral corners.

#### Diagnosis.

Both sexes: Hyperoccipital carina indicated by rugae. Medial mesoscutum (posteriorly) and mesoscutellum very weakly sculptured but without granulate areas, setae arising from tiny pits. Metascutellum slightly broadening posteriorly, with a weak median carina, nearly smooth lateral to carina. Female: A4 longer than broad. T1 midlobe with 6 or more longitudinal carinae. T6 longer than broad. Male: T7 with sharp, protruding posterolateral corners.

#### Etymology.

Latin noun, genitive case, meaning “roof.” Refers to the nearly smooth medial mesoscutum.

#### Link to distribution map.

[http://hol.osu.edu/map-full.html?id=275550]

#### Material examined.

Holotype, female: **INDONESIA**: Maluku Prov., Ceram (Seram) Isl., forest, Solea, VIII-1987, malaise trap, M. C. Day, OSUC 368938 (deposited in BMNH). *Paratypes*: **INDONESIA**: 2 males, OSUC 368940 (CNCI); OSUC 228709 (ROME).

#### Comments.

*Oxyscelio tecti*, as in other species from Seram, has very weak surface sculpture. This species is somewhat similar to the *crateris*-group, but may not be closely related to any Asian species.

### Oxyscelio unguis

Burks sp. n.

urn:lsid:zoobank.org:act:FE35292E-8F3C-4B65-A058-72E9C57CBC64

urn:lsid:biosci.ohio-state.edu:osuc_concepts:275561

http://species-id.net/wiki/Oxyscelio_unguis

**Figures 420–425.**
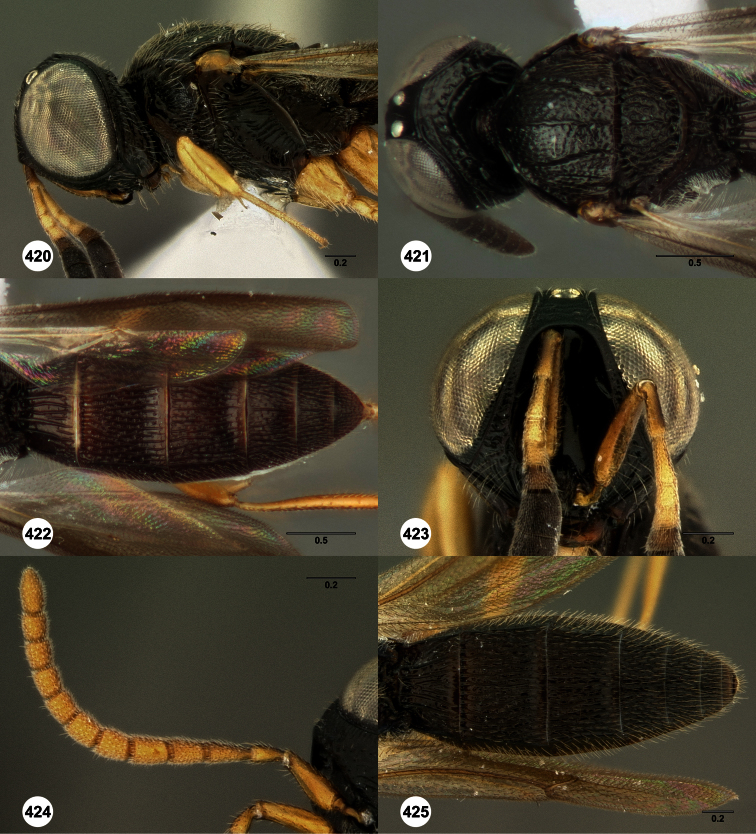
*Oxyscelio unguis* sp. n., paratype female (OSUC 240919) **420** Head and mesosoma, lateral view. Holotype female (OSUC 257040) **421** Head and mesosoma, dorsal view. **422** Metasoma, dorsal view. Paratype female (OSUC 241814) **423** Head, anterior view. Paratype male (OSUC 251427) **424** Antenna **425** Metasoma, dorsal view. Morphbank^110^

Figures 420–425 Morphbank^110^

#### Description.

*Female*. Body length 3.2–4.45 mm (n=12).

Radicle color: darker than scape. Scape color: Yellowish. A4: broader than long; as long as broad. A5: broader than long. Antennal club: formed, segments compact.

Interantennal process: not elongate. Median longitudinal elevation in frontal depression: absent. Frontal depression: concave. Frontal depression sculpture: with 2 oblique interrupted carinae. Submedian carina: strong, formed by a sharp raised carina. Submedian carina medially: without peak. Concavity across dorsal part of frontal depression: absent. Depression extending ventrally from median ocellus: absent. Upper frons: hood-like, protruding over pedicel when antenna at rest. Malar area near antennal foramen: without carina or expansion. Malar area at mouth corner: with radiating striae. Smooth strip along posterior side of malar sulcus: present, broad throughout its length. Middle genal carina: absent. Direction of middle genal carina dorsally: absent (replace with question mark). Major sculpture of gena anteriorly: rugose; umbilicate-punctate. Major sculpture of gena posteriorly: rugose; umbilicate-punctate. Microsculpture of gena anteroventrally: absent. Microsculpture of gena posteroventrally: absent. Median carina extending posteriorly from hyperoccipital carina: absent. Hyperoccipital carina: complete, continuous with anterior genal carina. Lateral connection between hyperoccipital and occipital carinae: absent. Area between vertex and occipital carina: irregularly rugose. Occipital carina medially: uniformly rounded. Lateral corners of occipital carina: sharp and protruding.

Lateral pronotal area: with slight bulge projecting anteriorly towards anterior pit. Epomial corner: weak. Netrion surface anteriorly: not inflexed. Mesoscutum anteriorly: not steep. Mesoscutal median carina: present and complete. Longitudinal carina between median carina and notauli: absent. Major sculpture of medial mesoscutum anteriorly: umbilicate-foveate. Major sculpture of medial mesoscutum posteriorly: umbilicate-punctate. Microsculpture of medial mesoscutum anteriorly: granulate. Microsculpture of medial mesoscutum posteriorly: absent. Major sculpture of mesoscutellum: umbilicate-foveate; longitudinally rugose. Microsculpture of mesoscutellum medially: absent. Microsculpture of mesoscutellum laterally: absent. Mesoscutellar apex: convex or straight. Setae along anterior limit of femoral depression: arising from rows of foveae. Number of carinae crossing speculum above femoral depression: 3. Number of carinae crossing femoral depression: more than 5. Mesepimeral sulcus pits: more than 5. Metascutellum dorsally: concave. Metascutellar sculpture dorsally: with scattered rugae. Median carina of metascutellum: absent or branched. Metascutellar setae: with many dorsal setae. Metascutellar apex: weakly emarginate. Metapleuron above ventral metapleural area: crossed by carinae. Metasomal depression setae: absent. Lateral propodeal carinae anteromedially: weakly diverging. Anterior areoles of metasomal depression: one or more areoles present. Anterior longitudinal carinae in metasomal depression: absent. Lateral propodeal areas: separated medially. Postmarginal vein: present. Fore wing apex: reaching apex of T6; reaching beyond T6.

T1 midlobe: with 4 longitudinal carinae. T1: without anterior bulge. T2: with straight longitudinal striae or rugae. T6: broader than long. Apical flange of T6: exposed apically. Metasomal apex: rounded. Major sculpture of T6: umbilicate-punctate. Microsculpture of T6: absent.

*Male*. Body length 4.1–4.25 mm (n=4). A5 tyloid: expanded, teardrop-shaped or sinuate. A11: longer than broad. Median tooth of frontal depression: absent. Median lobe of T1: with 5 longitudinal carinae. Metasomal apex: with acuminate lateral corners.

#### Diagnosis.

Both sexes: Frons without elevation between antennal foramen and eye. Hyperoccipital carina present, continuous with anterior genal carina. Medial mesoscutum weakly sculptured, without longitudinal rugae. Metascutellum with dorsal setae. Metasomal depression short; lateral propodeal carinae narrowly separated anteriorly. Postmarginal vein very long: more than 1/3 stigmal vein length, marginal vein narrow. Female: A4 as broad or broader than long, A5 broader than long. T1 with 4 longitudinal carinae, in two sets that are broadly separated medially. Male: A5 tyloid expanded. Frontal depression without tooth-like median protrusion dorsally. T1 midlobe with 5 longitudinal carinae. T7 with tiny, sharp and weakly protruding posterolateral corners.

#### Etymology.

Latin noun, genitive case, meaning “fingernail.” Refers to the usually elongate apical flange of T6 in females.

#### Link to distribution map.

[http://hol.osu.edu/map-full.html?id=275561]

#### Material examined.

Holotype, female: **INDONESIA**: Kalimantan Barat Prov., Cabang Panti Research Station, 1° rainforest / sandstone, IIS 910129, Gunung Palung National Park, 01°15'S, 110°05'E, 100–400m, 15.VI–15.VIII.1991, malaise trap/pan trap, Darling, Rosichon & Sutrisno, OSUC 257040 (deposited in MBBJ). *Paratypes*: (12 females, 4 males) **INDONESIA**: 7 females, 3 males, OSUC 464007 (CNCI); OSUC 240922, 240924, 257051 (MBBJ); OSUC 228708, 240921, 241814 (OSUC); OSUC 240919, 251427, 257090 (ROME). **MALAYSIA**: 4 females, 1 male, OSUC 376586, 376588 (BMNH); OSUC 369064-369065 (CNCI); OSUC 364961 (MZLU). **THAILAND**: 1 female, OSUC 335145 (QSBG).

#### Comments.

In females of *Oxyscelio unguis*, the longitudinal carinae of the T1 midlobe are separated into two sets by a broad median smooth area. A median 5th carina is present in males, which are also distinctive in having an expanded A5 tyloid. Several other species of *Oxyscelio* have a similarly expanded tyloid, including the very similar species *Oxyscelio ceylonensis*.

### Oxyscelio vadorum

Burks sp. n.

urn:lsid:zoobank.org:act:541F6577-6053-4340-BAE9-A78559C8D736

urn:lsid:biosci.ohio-state.edu:osuc_concepts:275483

http://species-id.net/wiki/Oxyscelio_vadorum

**Figures 426–429.**
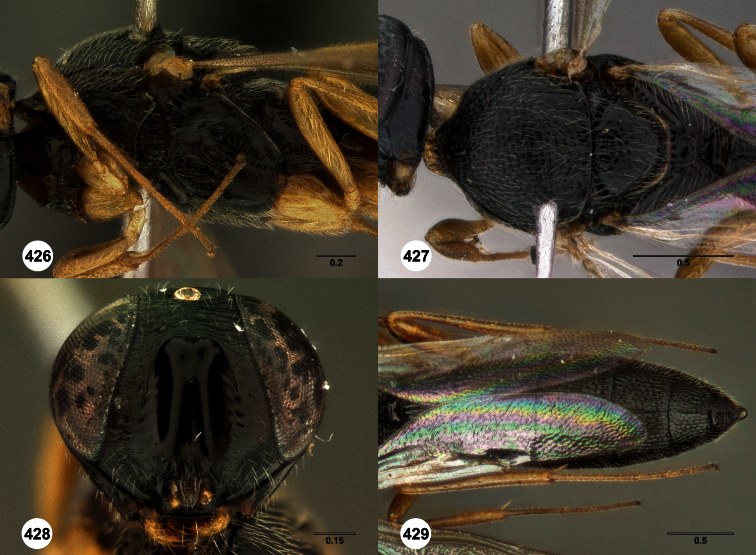
*Oxyscelio vadorum* sp. n., holotype female (OSUC 268137) **426** Head and mesosoma, lateral view **427** Head and mesosoma, dorsal view **428** Head, anterior view **429** Metasoma, dorsal view. Morphbank^111^

Figures 426–429 Morphbank^111^

#### Description.

*Female*. Body length 4.75 mm (n=1).

Interantennal process: not elongate. Median longitudinal elevation in frontal depression: present. Frontal depression: concave. Frontal depression sculpture: with 3 or more broadly interrupted transverse carinae. Submedian carina: strong, formed by a sharp raised carina. Submedian carina medially: without peak. Concavity across dorsal part of frontal depression: absent. Depression extending ventrally from median ocellus: absent. Upper frons: hood-like, protruding over pedicel when antenna at rest. Malar area near antennal foramen: without carina or expansion. Malar area at mouth corner: with radiating striae. Smooth strip along posterior side of malar sulcus: absent or not consistently broad. Middle genal carina: absent. Direction of middle genal carina dorsally: absent (replace with question mark). Major sculpture of gena anteriorly: umbilicate-foveate; umbilicate-punctate. Major sculpture of gena posteriorly: umbilicate-punctate. Microsculpture of gena anteroventrally: granulate. Microsculpture of gena posteroventrally: granulate. Median carina extending posteriorly from hyperoccipital carina: absent. Hyperoccipital carina: complete, continuous with anterior genal carina. Lateral connection between hyperoccipital and occipital carinae: absent. Area between vertex and occipital carina: umbilicate-punctate. Occipital carina medially: uniformly rounded. Lateral corners of occipital carina: not protruding.

Lateral pronotal area: without bulge projecting towards anterior pit. Epomial corner: weak. Netrion surface anteriorly: not inflexed. Mesoscutum anteriorly: not steep. Mesoscutal median carina: absent or weak and incomplete in places. Longitudinal carina between median carina and notauli: absent. Major sculpture of medial mesoscutum anteriorly: umbilicate-foveate. Major sculpture of medial mesoscutum posteriorly: umbilicate-foveate. Microsculpture of medial mesoscutum anteriorly: granulate. Microsculpture of medial mesoscutum posteriorly: absent. Major sculpture of mesoscutellum: umbilicate-foveate. Microsculpture of mesoscutellum medially: granulate. Microsculpture of mesoscutellum laterally: granulate. Mesoscutellar apex: convex or straight. Setae along anterior limit of femoral depression: arising from tiny pits. Number of carinae crossing speculum above femoral depression: 4. Number of carinae crossing femoral depression: 3-5. Mesepimeral sulcus pits: 3-5. Metascutellum dorsally: concave. Metascutellar sculpture dorsally: smooth or with transverse carinae. Median carina of metascutellum: absent or branched. Metascutellar setae: absent. Metascutellar apex: weakly emarginate. Metapleuron above ventral metapleural area: crossed by carinae. Metasomal depression setae: absent. Lateral propodeal carinae anteromedially: weakly diverging. Anterior areoles of metasomal depression: one or more areoles present. Anterior longitudinal carinae in metasomal depression: median carina present. Lateral propodeal areas: separated medially. Postmarginal vein: present. Fore wing apex: reaching beyond T6.

T1 midlobe: with 5 longitudinal carinae. T1: without anterior bulge. T2: with straight longitudinal striae or rugae. T6: broader than long. Apical flange of T6: exposed apically. Metasomal apex: rounded. Major sculpture of T6: umbilicate-punctate; longitudinally striate or rugose. Microsculpture of T6: absent.

*Male*. Unknown.

#### Diagnosis.

Female: Frons without elevation between antennal foramen and eye. Hyperoccipital carina present, continuous with anterior genal carina. Gena almost entirely granulate, without rugae, foveae, or carinae over most of its surface. Mesoscutellum strongly granulate. Metascutellum narrowing posteriorly, weakly emarginate. Metasomal depression elongate, with extensive sculpture; lateral propodeal carinae narrowly separated anteriorly. T1 midlobe with 5 longitudinal carinae. T6 rounded apically.

#### Etymology.

Latin noun, genitive case, meaning “shallows.” A pun referring to the predominantly weak surface sculpture.

#### Link to distribution map.

[http://hol.osu.edu/map-full.html?id=275483]

#### Material examined.

Holotype, female: **SRI LANKA**: Central Prov., Nuwara Eliya Dist., Hakgala Strict Natural Reserve, 23.VI–24.VI.1981, K. V. Krombein, T. Wijesinhe & L. Weeratunge, OSUC 268137 (deposited in ISDF).

#### Comments.

*Oxyscelio vadorum* superficially resembles many weakly sculptured species outside the *cuculli*-group. However, it has the elongate, sculptured metasomal depression found in *Oxyscelio convergens* and similar species. Even though only a single, damaged specimen is known, this species is described because of its many unusual character states, including the chiefly granulate gena.

### Oxyscelio vittae

Burks sp. n.

urn:lsid:zoobank.org:act:73405DA1-8966-4076-A5F2-92F1C718978D

urn:lsid:biosci.ohio-state.edu:osuc_concepts:305772

http://species-id.net/wiki/Oxyscelio_vittae

**Figures 430–433.**
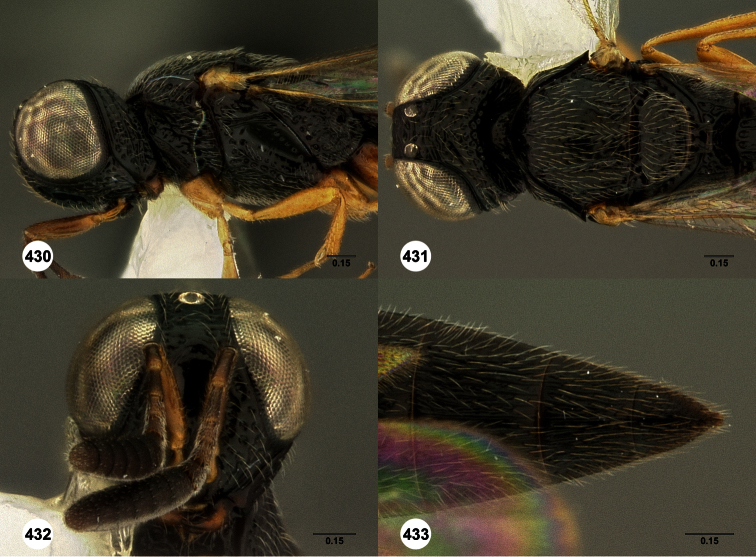
*Oxyscelio vittae* sp. n., holotype female (ROMEnt Spec. No. 112216) **430** Head and mesosoma, lateral view **431** Head and mesosoma, dorsal view **432** Head, anterior view **433** Metasoma, dorsal view. Morphbank^112^

Figures 430–433 Morphbank^112^

#### Description.

*Female*. Body length 4.2 mm (n=1).

Radicle color: same color as scape. Scape color: Brown. A4: broader than long; as long as broad. A5: broader than long. Antennal club: formed, segments compact.

Interantennal process: not elongate. Median longitudinal elevation in frontal depression: absent. Frontal depression: concave. Frontal depression sculpture: without transverse or oblique carinae below submedian carina. Submedian carina: weak, shallow and rounded or formed by ledge. Submedian carina medially: without peak. Concavity across dorsal part of frontal depression: absent. Depression extending ventrally from median ocellus: absent. Upper frons: not hood-like. Malar area near antennal foramen: without carina or expansion. Malar area at mouth corner: with radiating striae. Smooth strip along posterior side of malar sulcus: present, broad throughout its length. Middle genal carina: present. Direction of middle genal carina dorsally: parallel to eye margin. Major sculpture of gena anteriorly: umbilicate-foveate. Major sculpture of gena posteriorly: umbilicate-foveate. Microsculpture of gena anteroventrally: absent. Microsculpture of gena posteroventrally: absent. Median carina extending posteriorly from hyperoccipital carina: absent. Hyperoccipital carina: indicated by rugae. Lateral connection between hyperoccipital and occipital carinae: absent. Area between vertex and occipital carina: umbilicate-foveate. Occipital carina medially: absent. Lateral corners of occipital carina: not protruding.

Lateral pronotal area: without bulge projecting towards anterior pit. Epomial corner: strong. Netrion surface anteriorly: not inflexed. Mesoscutum anteriorly: not steep. Mesoscutal median carina: present and complete. Longitudinal carina between median carina and notauli: absent. Major sculpture of medial mesoscutum anteriorly: umbilicate-foveate; umbilicate-punctate. Major sculpture of medial mesoscutum posteriorly: umbilicate-foveate; umbilicate-punctate. Microsculpture of medial mesoscutum anteriorly: granulate. Microsculpture of medial mesoscutum posteriorly: granulate. Major sculpture of mesoscutellum: umbilicate-foveate. Microsculpture of mesoscutellum medially: granulate. Microsculpture of mesoscutellum laterally: granulate. Mesoscutellar apex: convex or straight. Setae along anterior limit of femoral depression: arising from rows of foveae. Number of carinae crossing speculum above femoral depression: 3. Number of carinae crossing femoral depression: 3-5. Mesepimeral sulcus pits: more than 5. Metascutellum dorsally: concave. Metascutellar sculpture dorsally: smooth or with transverse carinae. Median carina of metascutellum: absent or branched. Metascutellar setae: absent. Metascutellar apex: convex or straight. Metapleuron above ventral metapleural area: foveate or rugose. Metasomal depression setae: absent. Lateral propodeal carinae anteromedially: strongly diverging. Anterior areoles of metasomal depression: absent. Anterior longitudinal carinae in metasomal depression: absent. Lateral propodeal areas: separated medially. Postmarginal vein: present. Fore wing apex: reaching middle of T5.

T1 midlobe: with 5 longitudinal carinae. T1: without anterior bulge. T2: with straight longitudinal striae or rugae. T6: longer than broad. Apical flange of T6: exposed apically. Metasomal apex: rounded. Major sculpture of T6: umbilicate-punctate. Microsculpture of T6: granulate.

*Male*. Unknown.

#### Diagnosis.

Female: Frons without elevation between antennal foramen and eye; frontal depression weakly concave. Hyperoccipital carina defined by rugae, one of which is continuous with anterior genal carina. Metascutellum truncate apically, with 2 subapical setae. Fore wings long enough to reach middle of T5. Lateral propodeal carinae broadly separated anteriorly. T1 midlobe with 5 longitudinal carinae. *Oxyscelio vittae* is very similar to *Oxyscelio carinatus*, but differs in metascutellar shape and in the longer metasoma.

#### Etymology.

Latin noun, genitive case, meaning “ribbon.”

#### Link to Distribution Map.

[http://hol.osu.edu/map-full.html?id=305772]

#### Material examined.

Holotype, female: **PHILIPPINES**: Negros Oriental Prov., 7km W Valencia, 1° forest edge, ROM 873059, Cuernos de Negros Mountain, 09°17'N, 123°15'E, 700m, 19.VI–25.VI.1987, malaise trap/pan trap, D. C. Darling & E. Mayordo, ROMEnt Spec. No. 112216 (deposited in ROME).

### Oxyscelio zeuctomesos

Burks sp. n.

urn:lsid:zoobank.org:act:71E08BCE-715B-45FB-A40A-D3B177B3A037

urn:lsid:biosci.ohio-state.edu:osuc_concepts:275488

http://species-id.net/wiki/Oxyscelio_zeuctomesos

**Figures 434–439.**
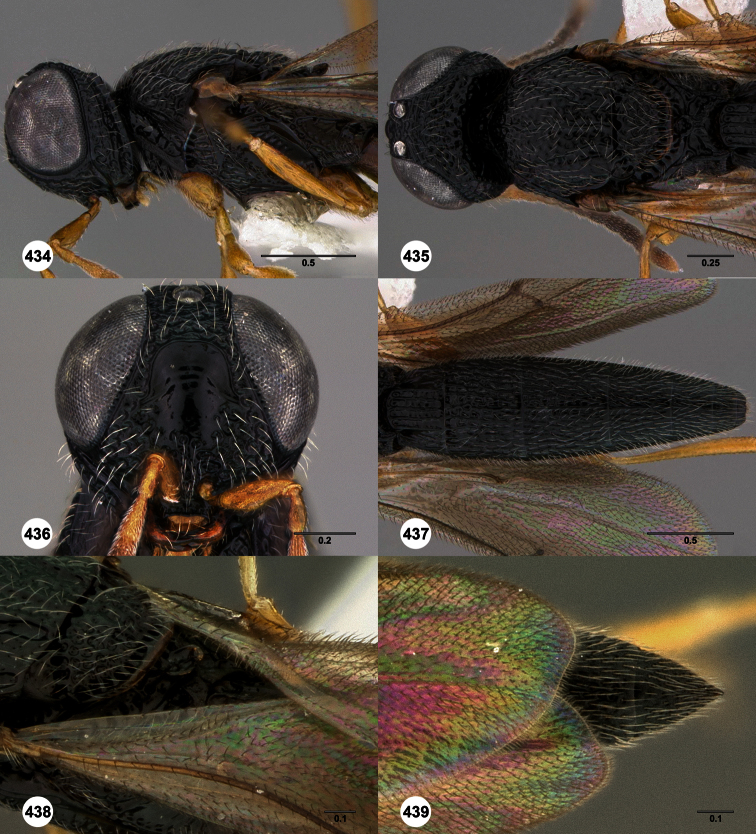
*Oxyscelio zeuctomesos* sp. n., paratype male (OSUC 369285) **434** Head and mesosoma, lateral view **435** Head and mesosoma, dorsal view **436** Head, anterior view **437** Metasoma, dorsal view. Paratype female (OSUC 369306) **438** Propodeum, oblique view **439** Metasomal apex, dorsal view. Morphbank^113^

Figures 434–439 Morphbank^113^

#### Description.

*Female*. Body length 3.85–4.4 mm (n=5).

Radicle color: same color as scape. Scape color: Yellowish. A4: longer than broad. A5: longer than broad; as long as broad. Antennal club: formed, segments compact.

Interantennal process: not elongate. Median longitudinal elevation in frontal depression: absent. Frontal depression: concave. Frontal depression sculpture: with 3-5 complete transverse carinae. Submedian carina: weak, shallow and rounded or formed by ledge. Submedian carina medially: without peak. Concavity across dorsal part of frontal depression: absent. Depression extending ventrally from median ocellus: absent. Upper frons: not hood-like. Malar area near antennal foramen: without carina or expansion. Malar area at mouth corner: without striae. Smooth strip along posterior side of malar sulcus: absent or not consistently broad. Middle genal carina: present. Direction of middle genal carina dorsally: parallel to eye margin. Major sculpture of gena anteriorly: umbilicate-foveate; rugose. Major sculpture of gena posteriorly: umbilicate-foveate; rugose. Microsculpture of gena anteroventrally: absent. Microsculpture of gena posteroventrally: absent. Median carina extending posteriorly from hyperoccipital carina: absent. Hyperoccipital carina: indicated by rugae. Lateral connection between hyperoccipital and occipital carinae: absent. Area between vertex and occipital carina: umbilicate-foveate. Occipital carina medially: divided into concave halves, meeting at median peak. Lateral corners of occipital carina: sharp and protruding.

Lateral pronotal area: without bulge projecting towards anterior pit. Epomial corner: weak. Netrion surface anteriorly: not inflexed. Mesoscutum anteriorly: not steep. Mesoscutal median carina: present and complete. Longitudinal carina between median carina and notauli: present. Major sculpture of medial mesoscutum anteriorly: umbilicate-foveate. Major sculpture of medial mesoscutum posteriorly: umbilicate-foveate; irregularly rugose. Microsculpture of medial mesoscutum anteriorly: granulate. Microsculpture of medial mesoscutum posteriorly: absent. Major sculpture of mesoscutellum: umbilicate-foveate; longitudinally rugose. Microsculpture of mesoscutellum medially: absent. Microsculpture of mesoscutellum laterally: absent. Mesoscutellar apex: convex or straight. Setae along anterior limit of femoral depression: arising from rows of foveae. Number of carinae crossing speculum above femoral depression: 4. Number of carinae crossing femoral depression: 3-5. Mesepimeral sulcus pits: more than 5. Metascutellum dorsally: concave. Metascutellar sculpture dorsally: smooth or with transverse carinae. Median carina of metascutellum: absent or branched. Metascutellar setae: absent. Metascutellar apex: weakly emarginate. Metapleuron above ventral metapleural area: smooth. Metasomal depression setae: absent. Lateral propodeal carinae anteromedially: weakly diverging. Anterior areoles of metasomal depression: absent. Anterior longitudinal carinae in metasomal depression: absent. Lateral propodeal areas: meeting for most of propodeal length as part of a raised structure. Postmarginal vein: absent. Fore wing apex: reaching middle of T5.

T1 midlobe: with 5 longitudinal carinae. T1: without anterior bulge. T2: with long sublateral depressions. T6: broader than long; as long as broad. Apical flange of T6: not exposed apically. Metasomal apex: tapering to a sharp point. Major sculpture of T6: umbilicate-punctate. Microsculpture of T6: absent; granulate.

*Male*. Body length 4.05 mm (n=2). A5 tyloid: carina-like, not expanded. A11: longer than broad. Median tooth of frontal depression: absent. Median lobe of T1: with 5 longitudinal carinae. Metasomal apex: with no distinct corners.

**Diagnosis**. Both sexes: Mesoscutellum without granulate sculpture. Metascutellum rounded, slightly expanded apically. Propodeum forming a roughly sculptured arch over the base of T1. Female: A4 longer than broad, A5 as long or longer than broad. T1 midlobe with 5 longitudinal carinae. T2 with sublateral depressions. T6 strongly tapering to a narrow point. Male: A11 as broad or slightly broader than long. T1 midlobe with 5 longitudinal carinae. T7 with rounded posterolateral corners.

#### Etymology.

Compound noun based on Greek, meaning “joined middle.” Refers to the arch formed by the propodeum over the base of T1.

#### Link to distribution map.

[http://hol.osu.edu/map-full.html?id=275488]

#### Material examined.

Holotype, female: **INDONESIA**: Sulawesi Utara Prov., Toraut, Bogani Nani Wartabone (Dumoga-Bone) National Park, 220m, 9.V–16.V.1985, J. S. Noyes, OSUC 369294 (deposited in BMNH). *Paratypes*:**INDONESIA**: 3 females, 2 males, OSUC 369227, 369285 (CNCI); OSUC 369271, 369306 (OSUC); OSUC 442264 (WINC).

#### Comments.

*Oxyscelio zeuctomesos* and *Oxyscelio cyrtomesos* form a species complex within the *fossarum*-group. They differ from most members of that group in that males do not have the T2 sublateral depressions.

## Supplementary Material

XML Treatment for Oxyscelio

XML Treatment for Oxyscelio aclavae

XML Treatment for Oxyscelio acutiventris

XML Treatment for Oxyscelio amrichae

XML Treatment for Oxyscelio anguli

XML Treatment for Oxyscelio angustifrons

XML Treatment for Oxyscelio angustinubbin

XML Treatment for Oxyscelio arcus

XML Treatment for Oxyscelio arvi

XML Treatment for Oxyscelio asperi

XML Treatment for Oxyscelio aureamediocritas

XML Treatment for Oxyscelio bipunctuum

XML Treatment for Oxyscelio brevidentis

XML Treatment for Oxyscelio brevinervis

XML Treatment for Oxyscelio caesitas

XML Treatment for Oxyscelio capilli

XML Treatment for Oxyscelio capitis

XML Treatment for Oxyscelio carinatus

XML Treatment for Oxyscelio cavinetrion

XML Treatment for Oxyscelio ceylonensis

XML Treatment for Oxyscelio chimaerae

XML Treatment for Oxyscelio codae

XML Treatment for Oxyscelio consobrinus

XML Treatment for Oxyscelio convergens

XML Treatment for Oxyscelio cordis

XML Treatment for Oxyscelio crassicornis

XML Treatment for Oxyscelio crateris

XML Treatment for Oxyscelio crebritas

XML Treatment for Oxyscelio crustum

XML Treatment for Oxyscelio cuculli

XML Treatment for Oxyscelio cupularis

XML Treatment for Oxyscelio cyrtomesos

XML Treatment for Oxyscelio dasymesos

XML Treatment for Oxyscelio dasynoton

XML Treatment for Oxyscelio dermatoglyphes

XML Treatment for Oxyscelio dorsalis

XML Treatment for Oxyscelio doumao

XML Treatment for Oxyscelio excavatus

XML Treatment for Oxyscelio fistulae

XML Treatment for Oxyscelio flabelli

XML Treatment for Oxyscelio flavipennis

XML Treatment for Oxyscelio flaviventris

XML Treatment for Oxyscelio florus

XML Treatment for Oxyscelio fodiens

XML Treatment for Oxyscelio fossarum

XML Treatment for Oxyscelio fossularum

XML Treatment for Oxyscelio foveatus

XML Treatment for Oxyscelio genae

XML Treatment for Oxyscelio granorum

XML Treatment for Oxyscelio granuli

XML Treatment for Oxyscelio greenacus

XML Treatment for Oxyscelio halmaherae

XML Treatment for Oxyscelio intermedietas Oxyscelio intermedietas

XML Treatment for Oxyscelio jaune

XML Treatment for Oxyscelio jugi

XML Treatment for Oxyscelio kiefferi

XML Treatment for Oxyscelio kramatos

XML Treatment for Oxyscelio labis

XML Treatment for Oxyscelio lacunae

XML Treatment for Oxyscelio latinubbin

XML Treatment for Oxyscelio latitudinis

XML Treatment for Oxyscelio limae

XML Treatment for Oxyscelio longiventris

XML Treatment for Oxyscelio magnus

XML Treatment for Oxyscelio marginalis

XML Treatment for Oxyscelio mesiodentis

XML Treatment for Oxyscelio mollitia

XML Treatment for Oxyscelio naraws

XML Treatment for Oxyscelio nasolabii

XML Treatment for Oxyscelio nodorum

XML Treatment for Oxyscelio noduli

XML Treatment for Oxyscelio nubbin

XML Treatment for Oxyscelio obsidiani

XML Treatment for Oxyscelio ogive

XML Treatment for Oxyscelio operimenti

XML Treatment for Oxyscelio peludo

XML Treatment for Oxyscelio perpensus

XML Treatment for Oxyscelio planocarinae

XML Treatment for Oxyscelio praecipitis

XML Treatment for Oxyscelio reflectens

XML Treatment for Oxyscelio regionis

XML Treatment for Oxyscelio rugosus

XML Treatment for Oxyscelio sinuum

XML Treatment for Oxyscelio spinae

XML Treatment for Oxyscelio spinosiceps

XML Treatment for Oxyscelio striarum

XML Treatment for Oxyscelio tecti

XML Treatment for Oxyscelio unguis

XML Treatment for Oxyscelio vadorum

XML Treatment for Oxyscelio vittae

XML Treatment for Oxyscelio zeuctomesos

## Endnotes

1 http://lsid.tdwg.org/urn:lsid:biosci.ohio-state.edu:osuc_pubs:1008

2 http://lsid.tdwg.org/urn:lsid:biosci.ohio-state.edu:osuc_pubs:32972

3 http://lsid.tdwg.org/urn:lsid:biosci.ohio-state.edu:osuc_pubs:32981

4 http://lsid.tdwg.org/urn:lsid:biosci.ohio-state.edu:osuc_pubs:1009

5 http://lsid.tdwg.org/urn:lsid:biosci.ohio-state.edu:osuc_pubs:1012

6 http://lsid.tdwg.org/urn:lsid:biosci.ohio-state.edu:osuc_pubs:1021

7 http://lsid.tdwg.org/urn:lsid:biosci.ohio-state.edu:osuc_pubs:33545

8 http://lsid.tdwg.org/urn:lsid:biosci.ohio-state.edu:osuc_pubs:33791

9 http://lsid.tdwg.org/urn:lsid:biosci.ohio-state.edu:osuc_pubs:33864

10 http://lsid.tdwg.org/urn:lsid:biosci.ohio-state.edu:osuc_pubs:1014

11 http://lsid.tdwg.org/urn:lsid:biosci.ohio-state.edu:osuc_pubs:34157

12 http://lsid.tdwg.org/urn:lsid:biosci.ohio-state.edu:osuc_pubs:34161

13 http://lsid.tdwg.org/urn:lsid:biosci.ohio-state.edu:osuc_pubs:34210

14 http://lsid.tdwg.org/urn:lsid:biosci.ohio-state.edu:osuc_pubs:34212

15 http://lsid.tdwg.org/urn:lsid:biosci.ohio-state.edu:osuc_pubs:1017

16 http://lsid.tdwg.org/urn:lsid:biosci.ohio-state.edu:osuc_pubs:34248

17 http://lsid.tdwg.org/urn:lsid:biosci.ohio-state.edu:osuc_pubs:34403

18 http://lsid.tdwg.org/urn:lsid:biosci.ohio-state.edu:osuc_pubs:1019

19 http://lsid.tdwg.org/urn:lsid:biosci.ohio-state.edu:osuc_pubs:34593

20 http://lsid.tdwg.org/urn:lsid:biosci.ohio-state.edu:osuc_pubs:35301

21 http://lsid.tdwg.org/urn:lsid:biosci.ohio-state.edu:osuc_pubs:33750

22 http://lsid.tdwg.org/urn:lsid:biosci.ohio-state.edu:osuc_pubs:1016

23 http://lsid.tdwg.org/urn:lsid:biosci.ohio-state.edu:osuc_pubs:34456

24 http://www.morphbank.net/?id=807654

25 http://www.morphbank.net/?id=807655

26 http://www.morphbank.net/?id=807656

27 http://www.morphbank.net/?id=807657

28 http://www.morphbank.net/?id=807658

29 http://www.morphbank.net/?id=807659

30 http://www.morphbank.net/?id=807660

31 http://www.morphbank.net/?id=807661

32 http://www.morphbank.net/?id=807662

33 http://www.morphbank.net/?id=807663

34 http://www.morphbank.net/?id=807664

35 http://www.morphbank.net/?id=807665

36 http://www.morphbank.net/?id=807666

37 http://www.morphbank.net/?id=807667

38 http://www.morphbank.net/?id=807668

39 http://www.morphbank.net/?id=807669

40 http://www.morphbank.net/?id=807670

41 http://www.morphbank.net/?id=807671

42 http://www.morphbank.net/?id=807672

43 http://www.morphbank.net/?id=807673

44 http://www.morphbank.net/?id=807674

45 http://www.morphbank.net/?id=807675

46 http://www.morphbank.net/?id=807676

47 http://www.morphbank.net/?id=807677

48 http://www.morphbank.net/?id=807678

49 http://www.morphbank.net/?id=807679

50 http://www.morphbank.net/?id=807680

51 http://www.morphbank.net/?id=807681

52 http://www.morphbank.net/?id=807682

53 http://www.morphbank.net/?id=807683

54 http://www.morphbank.net/?id=807684

55 http://www.morphbank.net/?id=807685

56 http://www.morphbank.net/?id=807686

57 http://www.morphbank.net/?id=807687

58 http://www.morphbank.net/?id=807688

59 http://www.morphbank.net/?id=807689

60 http://www.morphbank.net/?id=807690

61 http://www.morphbank.net/?id=807691

62 http://www.morphbank.net/?id=807692

63 http://www.morphbank.net/?id=807693

64 http://www.morphbank.net/?id=807694

65 http://www.morphbank.net/?id=807695

66 http://www.morphbank.net/?id=807696

67 http://www.morphbank.net/?id=807697

68 http://www.morphbank.net/?id=807698

69 http://www.morphbank.net/?id=807699

70 http://www.morphbank.net/?id=807700

71 http://www.morphbank.net/?id=807701

72 http://www.morphbank.net/?id=807702

73 http://www.morphbank.net/?id=807703

74 http://www.morphbank.net/?id=807704

75 http://www.morphbank.net/?id=807705

76 http://www.morphbank.net/?id=807706

77 http://www.morphbank.net/?id=807707

78 http://www.morphbank.net/?id=807708

79 http://www.morphbank.net/?id=807709

80 http://www.morphbank.net/?id=807710

81 http://www.morphbank.net/?id=807711

82 http://www.morphbank.net/?id=807712

83 http://www.morphbank.net/?id=807713

84 http://www.morphbank.net/?id=807714

85 http://www.morphbank.net/?id=807715

86 http://www.morphbank.net/?id=807716

87 http://www.morphbank.net/?id=807717

88 http://www.morphbank.net/?id=807718

89 http://www.morphbank.net/?id=807719

90 http://www.morphbank.net/?id=807720

91 http://www.morphbank.net/?id=807721

92 http://www.morphbank.net/?id=807722

93 http://www.morphbank.net/?id=807723

94 http://www.morphbank.net/?id=807724

95 http://www.morphbank.net/?id=807725

96 http://www.morphbank.net/?id=807726

97 http://www.morphbank.net/?id=807727

98 http://www.morphbank.net/?id=807728

99 http://www.morphbank.net/?id=807729

100 http://www.morphbank.net/?id=807730

101 http://www.morphbank.net/?id=807731

102 http://www.morphbank.net/?id=807732

103 http://www.morphbank.net/?id=807733

104 http://www.morphbank.net/?id=807734

105 http://www.morphbank.net/?id=807735

106 http://www.morphbank.net/?id=807736

107 http://www.morphbank.net/?id=807737

108 http://www.morphbank.net/?id=807738

109 http://www.morphbank.net/?id=807739

110 http://www.morphbank.net/?id=807740

111 http://www.morphbank.net/?id=807741

112 http://www.morphbank.net/?id=807742

113 http://www.morphbank.net/?id=807743

114 http://lsid.tdwg.org/urn:lsid:biosci.ohio-state.edu:osuc_pubs:20940

115 http://lsid.tdwg.org/urn:lsid:biosci.ohio-state.edu:osuc_pubs:119

116 http://lsid.tdwg.org/urn:lsid:biosci.ohio-state.edu:osuc_pubs:131

117 http://lsid.tdwg.org/urn:lsid:biosci.ohio-state.edu:osuc_pubs:175

118 http://lsid.tdwg.org/urn:lsid:biosci.ohio-state.edu:osuc_pubs:9560

119 http://lsid.tdwg.org/urn:lsid:biosci.ohio-state.edu:osuc_pubs:235

120 http://lsid.tdwg.org/urn:lsid:biosci.ohio-state.edu:osuc_pubs:409

121 http://lsid.tdwg.org/urn:lsid:biosci.ohio-state.edu:osuc_pubs:404

122 http://lsid.tdwg.org/urn:lsid:biosci.ohio-state.edu:osuc_pubs:21506

123 http://lsid.tdwg.org/urn:lsid:biosci.ohio-state.edu:osuc_pubs:339

124 http://dx.doi.org/doi:10.1111/j.1096-0031.2008.00217.x

125 http://lsid.tdwg.org/urn:lsid:biosci.ohio-state.edu:osuc_pubs:229

126 http://lsid.tdwg.org/urn:lsid:biosci.ohio-state.edu:osuc_pubs:327

127 http://lsid.tdwg.org/urn:lsid:biosci.ohio-state.edu:osuc_pubs:986

128 http://lsid.tdwg.org/urn:lsid:biosci.ohio-state.edu:osuc_pubs:987

129 http://lsid.tdwg.org/urn:lsid:biosci.ohio-state.edu:osuc_pubs:400

130 http://lsid.tdwg.org/urn:lsid:biosci.ohio-state.edu:osuc_pubs:309

131 http://lsid.tdwg.org/urn:lsid:biosci.ohio-state.edu:osuc_pubs:323

132 http://lsid.tdwg.org/urn:lsid:biosci.ohio-state.edu:osuc_pubs:402

133 http://lsid.tdwg.org/urn:lsid:biosci.ohio-state.edu:osuc_pubs:361

134 http://lsid.tdwg.org/urn:lsid:biosci.ohio-state.edu:osuc_pubs:324

135 http://lsid.tdwg.org/urn:lsid:biosci.ohio-state.edu:osuc_pubs:310

136 http://lsid.tdwg.org/urn:lsid:biosci.ohio-state.edu:osuc_pubs:21217

137 http://lsid.tdwg.org/urn:lsid:biosci.ohio-state.edu:osuc_pubs:22163

138 http://lsid.tdwg.org/urn:lsid:biosci.ohio-state.edu:osuc_pubs:9718

139 http://lsid.tdwg.org/urn:lsid:biosci.ohio-state.edu:osuc_pubs:219

140 http://lsid.tdwg.org/urn:lsid:biosci.ohio-state.edu:osuc_pubs:342

141 http://lsid.tdwg.org/urn:lsid:biosci.ohio-state.edu:osuc_pubs:311

142 http://lsid.tdwg.org/urn:lsid:biosci.ohio-state.edu:osuc_pubs:21304

143 http://lsid.tdwg.org/urn:lsid:biosci.ohio-state.edu:osuc_pubs:21300

144 http://lsid.tdwg.org/urn:lsid:biosci.ohio-state.edu:osuc_pubs:240

145 http://lsid.tdwg.org/urn:lsid:biosci.ohio-state.edu:osuc_pubs:21247

146 http://lsid.tdwg.org/urn:lsid:biosci.ohio-state.edu:osuc_pubs:21131

147 http://lsid.tdwg.org/urn:lsid:biosci.ohio-state.edu:osuc_pubs:23583

## Appendix I

Taxonomic records for all records used in the present paper. (doi: 10.3897/zookeys.292.3867.app1). File format: DarwinCore Archive (zip).

## Appendix II

Locality records for all records used in the present paper. (doi: 10.3897/zookeys.292.3867.app2). File format: DarwinCore Archive (zip).

## Appendix III

**Characters. * = used in phylogenetic analysis.**

1. Radicle color

1. same color as scape

2. darker than scape

2. Scape color

1. Yellowish

2. Brown

3. A4 [in females]*

1. broader than long

2. longer than broad

3. as long as broad

4. A5 [in females]*

1. broader than long

2. longer than broad

3. as long as broad

5. Antennal club [in females]

1. segments compact

2. segments not compact

6. Interantennal process

1. not elongate anteriorly

2. elongate anteriorly

7. Median longitudinal elevation in frontal depression

1. absent

2. present

8. Frontal depression*

1. concave

2. flat

9. Frontal depression sculpture

1. without transverse or oblique carinae below submedian carina

2. with 3 or more broadly interrupted transverse carinae

3. with 3–5 complete transverse carinae

4. with 1 complete transverse carina

5. with 2 oblique interrupted carinae

6. crossed by many tiny furrows

7. with 2 complete transverse carinae

10. Submedian carina*

1. strong, formed by a sharp raised carina

2. weak, shallow and rounded or formed by ledge

3. indicated by multiple weak carinae

4. absent

11. Submedian carina medially*

1. without peak

2. with sharp peak

12. Concavity across dorsal part of frontal depression

1. absent

2. present

13. Depression extending ventrally from median ocellus

1. absent

2. present

14. Upper frons*

1. not hood-like

2. hood-like, protruding over pedicel when antenna at rest

15. Malar area near antennal foramen

1. without carina or expansion

2. with oblique tooth-like flange (facial nubbin)

3. with long ruga extending from edge of frontal depression

4. with vertical carina extending from clypeus towards frontal depression

16. Malar area at mouth corner

1. without striae

2. with radiating striae

3. with one carina subparallel to malar sulcus

17. Smooth area along posterior side of malar sulcus

1. absent or not consistently broad

2. present, broad throughout its length

18. Middle genal carina

1. absent

2. present

19. Direction of middle genal carina dorsally

1. parallel to eye margin

2. curving towards genal carina dorsally

3. absent (replace with question mark)

20. Major sculpture of gena anteriorly

1. umbilicate-foveate

2. rugose

3. umbilicate-punctate

21. Major sculpture of gena posteriorly

1. absent

2. umbilicate-foveate

3. rugose

4. umbilicate-punctate

22. Microsculpture of gena antero-ventrally

1. absent

2. granulate

23. Microsculpture of gena postero-ventrally

1. absent

2. granulate

3. punctate

24. Median carina extending posteriorly from hyperoccipital carina*

1. absent

2. present

25. Hyperoccipital carina*

1. complete, continuous with anterior genal carina

2. not indicated medially

3. weakly indicated by rugae medially

26. Lateral connection between hyperoccipital and occipital carinae*

1. absent

2. present as a distinct carina

3. present as a weak elevation

27. Area between vertex and occipital carina

1. umbilicate-foveate

2. with transverse carinae

3. irregularly rugose

4. crenulate

5. umbilicate-punctate

28. Occipital carina medially*

1. uniformly rounded

2. convex, with a sharp median peak

3. flat

4. divided into concave halves, meeting at median peak

5. absent

6. sinuate, concave medial to corners, but without a median peak

7. slightly convex, flatter medially than laterally

29. Dorso-lateral corners of occipital carina*

1. not protruding

2. sharp and protruding

30. Lateral pronotal area

1. without bulge projecting towards anterior pit

2. with slight bulge projecting anteriorly towards anterior pit

31. Epomial corner

1. strong

2. weak

32. Netrion surface anteriorly*

1. not inflexed

2. inflexed

33. Mesoscutum anteriorly

1. steep

2. not steep

34. Mesoscutal median carina

1. present and complete

2. absent or weak and incomplete in places

35. Longitudinal carina between median carina and notauli*

1. absent

2. present

36. Major sculpture of medial mesoscutum anteriorly

1. umbilicate-foveate

2. longitudinally rugose

3. umbilicate-punctate

4. transversely rugose

5. irregularly rugose

37. Major sculpture of medial mesoscutum posteriorly

1. umbilicate-foveate

2. umbilicate-punctate

3. longitudinally rugose

4. transversely rugose

5. irregularly rugose

38. Microsculpture of medial mesoscutum anteriorly

1. absent

2. granulate

39. Microsculpture of medial mesoscutum posteriorly

1. absent

2. granulate

40. Major sculpture of mesoscutellum

1. umbilicate-foveate

2. longitudinally rugose

3. transversely rugose

4. irregularly rugose

5. umbilicate-punctate

6. obliquely rugose

41. Microsculpture of mesoscutellum medially

1. absent

2. granulate

3. punctate

42. Microsculpture of mesoscutellum laterally

1. absent

2. granulate

3. punctate

43. Mesoscutellar apex*

1. convex or straight

2. roundly concave

3. incised

44. Number of carinae crossing speculum above femoral depression*

1. 2

2. 3

3. 4

45. Number of carinae crossing femoral depression

1. 3-5

2. more than 5

46. Mesepimeral sulcus pits

1. 3-5

2. more than 5

47. Setae along anterior limit of femoral depression

1. arising from rows of foveae

2. arising from tiny pits

48. Metascutellum dorsally*

1. concave

2. flat

3. convex

49. Metascutellar sculpture dorsally*

1. smooth or with transverse carinae

2. with scattered rugae

3. foveate

4. granulate

50. Median carina of metascutellum*

1. absent or branched

2. straight, unbranched carina present

51. Metascutellar setae*

1. absent

2. present dorsally

52. Metascutellar apex*

1. deeply emarginate

2. convex or straight

3. weakly emarginate

53. Metapleuron above ventral metapleural area

1. crossed by carinae

2. foveate or rugose

3. smooth

54. Metasomal depression setae*

1. absent

2. present

55. Lateral propodeal carinae antero-medially [in females]

1. strongly diverging

2. weakly diverging

56. Anterior areoles of metasomal depression

1. absent

2. one or more areoles present

57. Anterior longitudinal carinae in metasomal depression

1. absent

2. median carina present

3. pair of submedian carinae present

58. Lateral propodeal areas

1. meeting for only a short distance medially

2. meeting for most of propodeal length as part of a raised structure

3. separated medially

59. Postmarginal vein*

1. present

2. absent

60. Forewing apex [in females]

1. reaching middle of T4

2. reaching apex of T4

3. reaching middle of T5

4. reaching apex of T5

5. reaching apex of T6

6. reaching beyond apex of T6

7. reaching middle of T6

61. T1 midlobe [in females]*

1. with 4 longitudinal carinae

2. with 5 longitudinal carinae

3. with 6 or more longitudinal carinae

4. obscured by other raised sculpture

62. T1 [in females]*

1. without anterior bulge

2. with small rounded anterior bulge, not reaching metascutellum

3. with long anterior bulge, reaching metascutellum

63. T2 [in females]*

1. with straight longitudinal striae or rugae

2. irregularly areolate

3. with strong set of curved striae

4. with long sublateral depressions

64. T6 [in females]

1. broader than long

2. longer than broad

3. as long as broad

65. Apical flange of T6 [in females]

1. exposed apically

2. not exposed apically

66. Metasomal apex [in females]*

1. rounded

2. tapering to a sharp point

67. Major sculpture of T6 [in females]

1. umbilicate-punctate

2. longitudinally striate or rugose

68. Microsculpture of T6 [in females]

1. absent

2. granulate

69. A5 tyloid [in males]

1. carina-like, not expanded

2. expanded, teardrop-shaped or sinuate

70. A11 [in males]

1. longer than broad

2. broader than long

3. as long as broad

71. Median tooth of frontal depression [in males]*

1. absent

2. present

72. Median lobe of T1 [in males]

1. with 3 longitudinal carinae

2. with 4 longitudinal carinae

3. with 5 longitudinal carinae

4. with 6 longitudinal carinae

73. Metasomal apex [in males]*

1. with acuminate lateral corners

2. with no distinct corners

3. with tiny rounded tubercles

4. with rounded but projecting lobe-like corners

## Matrix

Bracalba_cuneata 0003000100000012201200200001

Oxyscelio_aclavae 11000001051000100001003130??

Oxyscelio_acutiventris 11010002041001111002003230??

Oxyscelio_amrichae [02]0000002040010[01]0010200100100

Oxyscelio_anguli [02]000000200001011100101310000

Oxyscelio_angustifrons 0000010005000010000200200000

Oxyscelio_angustinubbin 1100000224100000000201200100

Oxyscelio_arcus 0000000005100010101200000001

Oxyscelio_arvi 11000002000000020001003200??

Oxyscelio_asperi 1[02]00000100000010000100100000

Oxyscelio_aureamediocritas 11000100010000100000001000??

Oxyscelio_bipunctuum 000000000[04]000000000000000011

Oxyscelio_brevidentis 0000010000100010201200000010

Oxyscelio_brevinervis ??000002000000[01]0000100????00

Oxyscelio_caesitas 1[12]010002041001111[01]0200322000

Oxyscelio_capilli 0000000200001000000200100000

Oxyscelio_capitis 0000000200001011100100320000

Oxyscelio_carinatus [02]011000204000000001000101000

Oxyscelio_cavinetrion 1001101103110010000200000000

Oxyscelio_ceylonensis [02]00000000[05]000010101200000001

Oxyscelio_chimaerae 11010002000000000002003100??

Oxyscelio_codae [02]001000204001000000100310000

Oxyscelio_consobrinus 0001000200001000000200100000

Oxyscelio_convergens 110001[01]001000010000200000003

Oxyscelio_cordis 11000012131002101001002000??

Oxyscelio_crassicornis ??00011001000010101200????00

Oxyscelio_crateris 1100000011100000000000100001

Oxyscelio_crebritas 0000000[12]0000[01]000000100100000

Oxyscelio_crustum 1100000000000010101200[13][01]0[01]00

Oxyscelio_cuculli 0000010005000010000200000000

Oxyscelio_cupularis 1100100105100000000[12]003100??

Oxyscelio_cyrtomesos [02]000100225100010000101203103

Oxyscelio_dasymesos 1[12]0100022410000000[01]010200100

Oxyscelio_dasynoton 1[12]11000200000000101010101100

Oxyscelio_dermatoglyphes 1[12]02000200000010100100310002

Oxyscelio_dorsalis ???????????000?1100100?????0

Oxyscelio_doumao 100001000100001010100[01]0000??

Oxyscelio_excavatus ??00000200000000000100????00

Oxyscelio_fistulae ??00000204100010000200????00

Oxyscelio_flabelli 11001002231000111002013100??

Oxyscelio_flavipennis 10010002041100011002003100??

Oxyscelio_flaviventris 00001012000010100002001000??

Oxyscelio_florus 1102000200000011100100320000

Oxyscelio_fodiens 11010001041001121000013130??

Oxyscelio_fossarum 1[12]00000224100011110[12]00313000

Oxyscelio_fossularum 1[02]00000225100000100[12]00[23]13003

Oxyscelio_foveatus 1000000204100011100100320000

Oxyscelio_genae 000010[01]020000000000200100000

Oxyscelio_granorum [12]000010000000010000200000001

Oxyscelio_granuli 1100000220000010100[12]001001??

Oxyscelio_greenacus 11010001041001121002013200??

Oxyscelio_halmaherae ??00000206100000000100????03

Oxyscelio_intermedietas 000000000[01]000010000[02]00000001

Oxyscelio_jaune 00000001040000011001003200??

Oxyscelio_jugi [12]000[01]00204001010000200100100

Oxyscelio_kiefferi ??00000204000010000100????00

Oxyscelio_kramatos 1[12]00000226000010000200000002

Oxyscelio_labis 00011011061000100002001000??

Oxyscelio_lacunae 00010002000000100001011000??

Oxyscelio_latinubbin 11001001051000100001011001??

Oxyscelio_latitudinis 1[12]01000204100[01]1[12]100[12]00320000

Oxyscelio_limae 0000000100001011100101310000

Oxyscelio_longiventris [02]000000204001010000200310000

Oxyscelio_magnus 0000000200100000000100200000

Oxyscelio_marginalis 10110002040000000002001000??

Oxyscelio_mesiodentis 0000010000[01]00010201200000010

Oxyscelio_mollitia 1100000200000010000200310000

Oxyscelio_naraws [12][012]000001041000111[01]0101310000

Oxyscelio_nasolabii 11011002201000100102013100??

Oxyscelio_nodorum 11010002000000000001003100??

Oxyscelio_noduli 11010002000000100001002100??

Oxyscelio_nubbin [02]000010005001010000200000100

Oxyscelio_obsidiani 11010001041000000001003100??

Oxyscelio_ogive 1000101223100010000201[01]000??

Oxyscelio_operimenti 1001000225100102[13]000013200??

Oxyscelio_peludo 1[02]01000224100011101100310100

Oxyscelio_perpensus 11010001251000111002013100??

Oxyscelio_planocarinae 00000102221000100002001000??

Oxyscelio_praecipitis ??00000204000010001200????00

Oxyscelio_reflectens [02]000000100000010000100100001

Oxyscelio_regionis [02]0010001040001111001003100??

Oxyscelio_rugosus ??00000206100000000100????00

Oxyscelio_sinuum 1000100223100001100[12]003200??

Oxyscelio_spinae 1100000013100010000000310101

Oxyscelio_spinosiceps 00[01]1000200000010001000100000

Oxyscelio_striarum 11010002241000111001013220??

Oxyscelio_tecti 11010002141000011[01]0100300000

Oxyscelio_unguis [02]000010000100010101200000000

Oxyscelio_vadorum ??000100000000000002001000??

Oxyscelio_vittae [02]00100020400000000?1001000??

Oxyscelio_zeuctomesos 1[12]01000203101000000201103101

## References

[B1] Austin AD Field SA (1997) The ovipositor system of scelionid and platygastrid wasps (Hymenoptera: Platygastroidea): comparative morphology and phylogenetic implications. Invertebrate Taxonomy 11: 1–87.^114^doi: 10.1071/IT95048

[B2] Baltazar CR (1966) A catalogue of Philippine Hymenoptera (with a bibliography, 1758-1963). Pacific Insects Monographs 8: 1–488.^115^

[B3] Bin F (1981) Definition of female antennal clava based on its plate sensilla in Hymenoptera Scelionidae Telenominae. Redia 64: 245–261.^116^

[B4] Brues CT (1908) Hymenoptera. Fam. Scelionidae. Genera Insectorum, 80: 1–59.^117^

[B5] Dangerfield P Austin A Baker G (2001) Biology, Ecology and Systematics of Australian Scelio, wasp parasitoids of locust and grasshopper eggs. CSIRO Publications, Collingwood, Victoria. 254 pp.^118^

[B6] De Santis L (1980) Catalogo de los himenopteros brasilenos de la serie Parasitica incluyendo Bethyloidea. Editora da Universidade Federal do Parana, Curitiba, Brazil, 395 pp.^119^

[B7] Dodd AP (1920) Notes on the exotic Proctotrupoidea in the British and Oxford University Museums, with descriptions of new genera and species. Transactions of the Entomological Society of London 1919: 321–382.^120^

[B8] Dodd AP (1931) The genus *Oxyscelio* Kieffer, its synonymy and species, with a description of one new genus (Hymenoptera: Proctotrypoidea). Proceedings of the Royal Society of Queensland 42: 71–81.^121^

[B9] Eady RD (1968) Some illustrations of microsculpture in the Hymenoptera. Proceedings of the Royal Entomological Society of London (A) 43: 66–72.^122^ doi: 10.1111/j.1365-3032.1968.tb01029.x

[B10] Galloway ID Austin AD (1984) Revision of the Scelioninae (Hymenoptera: Scelionidae) in Australia. Australian Journal of Zoology Supplementary Series 99: 1–138.^123^

[B11] Goloboff PA Farris JS Nixon KC (2003) T.N.T.–Tree Analysis Using New Technology, version 1.1. Computer software and manual, available at: (http://www.zmuc.dk/public/ phylogeny).

[B12] Goloboff PA Farris JS Nixon KC (2008) TNT: a free program for phylogenetic analysis. Cladistics 24: 774–786.^124^doi: 10.1111/j.1096-0031.2008.00217.x

[B13] Johnson NF (1992) Catalog of world Proctotrupoidea excluding Platygastridae. Memoirs of the American Entomological Institute 51: 1–825.^125^

[B14] Kelner-Pillault S (1958) Catalogue de quelques types d’Hymenopteres provenant de la collection de l’Abbe J. J. Kieffer. Bulletin du Museum National d’Histoire Naturelle 30(2): 146–153.^126^

[B15] Kieffer JJ (1907) Beschreibung neuer Prototrypiden aus Java. (Hym.) Zeitschrift für Systematische Hymenopterologie und Dipterologie 7: 310–313.^127^

[B16] Kieffer JJ (1908) Zwei neue Serphiden aus Java (Hymenoptera). Notes from the Leyden Museum 30: 92–94.^128^

[B17] Kieffer JJ (1910a) Description de nouveaux microhyménoptères du Brésil. Annales de la Société Entomologique de France 78: 287–348.^129^

[B18] Kieffer JJ (1910b) Hymenoptera. Fam. Scelionidae. Addenda et corrigenda. Genera Insectorum 80: 61–112.^130^

[B19] Kieffer JJ (1913a) Proctotrypidae (3e partie). Species des Hymenopteres d’Europe et d’Algerie 11: 161–304.^131^

[B20] Kieffer JJ (1913b) Serphides des Îles Philippines. Insecta 3: 253–462.^132^

[B21] Kieffer JJ (1914) Enumeration des Serphides (Proctotrupides) des Iles Philippines avec description de genres nouveaux et d’especes nouvelles. Philippine Journal of Science (D) 9: 285–311.^133^

[B22] Kieffer JJ (1916) Neue Scelioniden aus den Philippinen-Inseln. Brotéria 14: 58–187.^134^

[B23] Kieffer JJ (1926)*Scelionidae*. Das Tierreich. Vol. 48. Walter de Gruyter & Co., Berlin, 885 pp.^135^

[B24] Kononova SV Fursov VN (2007) A review of the genera *Calotelea*, *Calliscelio*, and *Oxyscelio* (Scelioninae, Scelionidae, Proctotrupoidea) from the Palaearctic fauna. Entomological Review 87: 92–105.^136^doi: 10.1134/S0013873807010101

[B25] Kononova SV Kozlov VN (2008) [Scelionids of the Palearctic (Hymenoptera, Scelionidae). Subfamily Scelioninae]. Tovarishchestvo Nauchnykh Izdanii KMK, Saint Petersburg. 489 pp.^137^

[B26] Lê XH (2000) Egg-parasites of family Scelionidae (Hymenoptera). Fauna of Vietnam, vol. 3. Science and Technics Publishing House, Hanoi. 386 pp.^138^

[B27] Mani MS (1941) Serphoidea. Catalogue of Indian Insects 26: 1–60.^139^

[B28] Masner L (1965) The types of Proctotrupoidea (Hymenoptera) in the British Museum (Natural History) and in the Hope Department of Entomology, Oxford. Bulletin of the British Museum (Natural History) Entomology Supplement 1: 1–154.^140^

[B29] Masner L (1976) Revisionary notes and keys to world genera of Scelionidae (Hymenoptera: Proctotrupoidea). Memoirs of the Entomological Society of Canada97: 1–87.^141^doi: 10.4039/entm10897fv

[B30] Masner L Johnson NF (2007) *Xentor*, a new endemic genus from Fiji (Hymenoptera: Platygastroidea: Scelionidae) and description of three new species. Fiji Arthropods, 9: 11-20.^142^

[B31] Mikó I Vilhelmsen L Johnson NF Masner L Pénzes Z (2007) Skeletomusculature of Scelionidae (Hymenoptera: Platygastroidea): head and mesosoma. Zootaxa 1571, 1–78.^143^

[B32] Muesebeck CFW Walkley LM (1956) Type species of the genera and subgenera of parasitic wasps comprising the superfamily Proctotrupoidea (order Hymenoptera). Proceedings of the United States National Museum 105: 319–419.^144^ doi: 10.5479/si.00963801.3359.319

[B33] Murphy NP Carey D Castro LR Dowton M Austin AD (2007) Phylogeny of the platygastroid wasps (Hymenoptera) based on sequences from the 18S rRNA, 28S rRNA and cytochrome oxidase I genes: implications for evolution of the ovipositor system and host relationships. Biological Journal of the Linnean Society 91: 653–669.^145^doi: 10.1111/j.1095-8312.2007.00825.x

[B34] Rajmohana K (2006) Studies on Proctotrupoidea and Platygastroidea (Hymenoptera: Insecta) of Kerala. Memoirs of the Zoological Survey of India 21(1): 1–153.^146^

[B35] Talamas EJ Masner L Johnson NF (2011) Revision of the *Paridris nephta* species group (Hymenoptera, Platygastroidea, Platygastridae). ZooKeys 133: 49–94.^147^doi: 10.3897/zookeys.133.1613 PMC3208429 22140338

